# A systematic review on malaria and Tuberculosis (TB) vaccine challenges in sub-Saharan African clinical trials

**DOI:** 10.1371/journal.pone.0317233

**Published:** 2025-01-24

**Authors:** Maonezi Abas Hamisi, Nur Ain Mohd Asri, Aini Syahida Mat Yassim, Rapeah Suppian

**Affiliations:** 1 School of Science and Technical Education (CoSTE), Mbeya University of Science and Technology, Mbeya, Tanzania; 2 School of Health Sciences, Universiti Sains Malaysia, Kota Bharu, Kelantan, Malaysia; Fundação Oswaldo Cruz Centro de Pesquisas René Rachou: Fundacao Oswaldo Cruz Instituto Rene Rachou, BRAZIL

## Abstract

**Objective:**

For more than a century, developing novel and effective vaccines against malaria and Tuberculosis (TB) infections has been a challenge. This review sought to investigate the reasons for the slow progress of malaria and TB vaccine candidates in sub-Saharan African clinical trials.

**Methods:**

The systematic review protocol was registered on PROSPERO on July 26, 2023 (CRD42023445166). The research articles related to the immunogenicity, efficacy, or safety of malaria or TB vaccines that were published between January 1, 2012, and August 31, 2023, were searched on three databases: Web of Science (WoS), PubMed, and ClinicalTrials.gov.

**Results:**

A total of 2342 articles were obtained, 50 of which met the inclusion criteria. 28 (56%) articles reported on malaria vaccine attributes, while 22 (44%) articles reported on TB vaccines. In both cases, the major challenges in sub-Saharan African clinical trials were immunogenicity and efficacy, rather than safety.

**Conclusion:**

Factors such as population characteristics, pathogen genetic diversity, vaccine nature, strategy, and formulation were associated with slow progress of the malaria and TB vaccine candidates in sub-Saharan African clinical trials.

## 1. Introduction

Malaria and Tuberculosis (TB) are among the top ten causes of death in low- and middle-income countries, the majority of which are sub-Saharan countries [1]. Malaria is a plasmodium-borne infection that is spread by female Anopheles mosquitoes [2]. *Plasmodium falciparum* species are the dominant cause of human malaria [3]. In 2021, 619,000 deaths were caused by malaria worldwide, 95% of which occurred in sub-Saharan Africa [4]. The favourable environment for *P*. *falciparum* species [5] and healthcare systems for instance, poor hospitals, treatments, and poor drug stewardships contribute to this prevalence [6]. In contrast, human TB is an airborne disease caused by *Mycobacterium tuberculosis* (Mtb). TB spreads through respiratory system encounters active Mtb-containing air droplets [7]. TB infections were the leading cause of human deaths prior to COVID-19 [7]. Human TB causes 10.6 million cases and 1.6 deaths, 90% of which occurred in sub-Saharan Africa [7].

Currently, chemical drugs, as well as vaccines, are used for the control of malaria [8] and TB [7]. However, the development of resistant strains has made drug use inefficient and costly. Vaccines are the most effective options for these diseases, and they can help to prevent the spread of resistance. Despite not being fully certified, the malaria RTS, S/AS01, was recommended for the pilot vaccination of 5- to 17-month-old children living in high-endemic areas [9]. Unlike malaria, BCG is the only certified TB vaccine. Both RTS, S/AS01 [10], and BCG [11] share similar limitations: they provide protection but limited to young age groups. Different groups of malaria vaccine candidates have been tested in sub-Saharan African clinical trials. Some of these include subunit vaccines [12–16], viral-like particle vaccines [17–20], and whole attenuated vaccines [21–23]. Like malaria vaccine candidates, TB vaccine candidates include subunit vaccines [24–33], inactivated vaccines [34], and whole attenuated vaccines [11,35,36]. Nevertheless, the progress of malaria and TB vaccine development in sub-Saharan Africa has been arduous and frequently regarded as sluggish. The aim of this study was to elucidate the barriers that hinder the rapid advancement of efficacious malaria and TB vaccines, with a specific emphasis on immunogenicity, effectiveness, and safety in sub-Saharan Africa.

## 2. Methodology

The review protocol was registered on PROSPERO (CRD42023445166). In brief, this review followed the Preferred Reporting Items for Systematic Reviews and Meta-Analysis (PRISMA) guidelines [37]. The keywords and review questions were formulated based on PICO: Sub-Saharan Africans (population), malaria and TB vaccine candidates (intervention), non-vaccinated, placebo, or any control setting (control), immunogenicity, efficacy, and safety (outcomes). The review question was “What are the challenges that hinder the rapid development of malaria and TB vaccines in sub-Saharan African clinical trials?”.

### 2.1 Article identification

The review began with the identification of keywords and their respective synonyms by the first and second authors (HM and NA, respectively). The keywords and synonyms used to formulate the search strategy were based on the PICO formulation, which includes Sub-Saharan Africans (population), malaria and TB vaccine candidates (intervention), non-vaccinated, placebo, or any control setting (control), immunogenicity, efficacy, and safety (outcomes) as summarized in Table 1. The search was performed between the 1st of August 2023 and the 31st of August 2023 on three databases, Web of Science, PubMed, and ClinicalTrials.gov, with some refinement to meet the inclusion criteria.

**Table 1:** The search strategy applied in this study.

Databases	Filter	String/Option
**Web of Sciences**	Only English, Article type, Sub-Saharan Countries by excluding Libya, Egypt, Algeria, Morocco and Tunisia	TS = (("Tuberculosis*" OR "Malaria*" OR "Vaccine" OR "Vaccine candidate") AND ("immunogenicity” OR "safety" OR "efficacy" OR "protect" OR "immune respons*" OR "CD4+" OR "CD8+" OR "antibody respons*") AND ("Africa" OR "Sub Sahara") NOT ("typhoid" OR "SARS" OR "Covid-19" OR "rotavirus" OR "influenza" OR "ebola" OR "drug" OR "yellow fever"))
**PubMed**	Full text, Randomized Controlled Trial, Humans, English	“(("Malaria") OR ("Tuberculosis")) AND ("vaccin*")) OR ("vaccine candidat*")) AND ("immunogenicity")) OR ("safety")) OR ("efficacy")) OR ("protect")) OR ("immune respons*")) OR ("CD4+ ")) OR ("CD8+")) OR ("antibody respons*")) AND ("Africa")) OR ("Sub Sahara")) NOT ("typhoid")) NOT ("SARS")) NOT ("Covid-19")) NOT ("rotavirus")) NOT ("influenza")) NOT ("ebola")) NOT ("drug")) NOT ("yellow fever")).
**ClinicalTrials.gov**	N/A	ClinicalTrials.gov: Immunogenicity OR safety OR efficacy OR CD4+ OR CD8+ OR protection OR immune response OR Africa OR Sub Sahara | Completed Studies | Studies With Results | malaria OR tuberculosis | vaccine OR vaccine candidate | Studies that accept healthy volunteers | Start date from 01/01/2012 to 08/31/2023

*N/A: Not Available.

### 2.2 Article screening and eligibility

Two authors, HM, and NA screened the articles obtained independently. Malaria or TB vaccine candidate studies that reported immunogenicity, efficacy, safety, or a combination, involved a sub-Saharan population, were randomised clinical trials, published in English between January 1, 2012, and August 31, 2023, were included. Studies not meeting these criteria were excluded. The discrepancies occurred were resolved by involving the third and fourth authors (AS and RS). The duplicate and irrelevant articles were removed using Mendeley software (2.107.0, 2023).

### 2.3 Assessment of study quality

The risk of bias in the eligible studies was analysed by the first author (HM) using the Cochrane risk of bias tool (RoB 2) [37]. The other three co-authors confirmed the findings (NA, AS, and RS). Each randomised controlled trial was rated as “high,” “low,” or “some concerns” for bias in five domains: randomization process, deviation from intervention, missing outcome data, measurement of outcomes, and selection of reported outcomes.

### 2.4 Data extraction and synthesis

Data were extracted and compiled from all eligible articles on the standard data collection table. The first author’s surname, publication year, population, region (country), clinical trial phase, intervention, control, and number of doses were retrieved for the articles. We then performed a narrative synthesis of the literature using our outcome keywords immunogenicity, efficacy, and safety.

## 3. Results

### 3.1 Study selection

**Fig 1:**
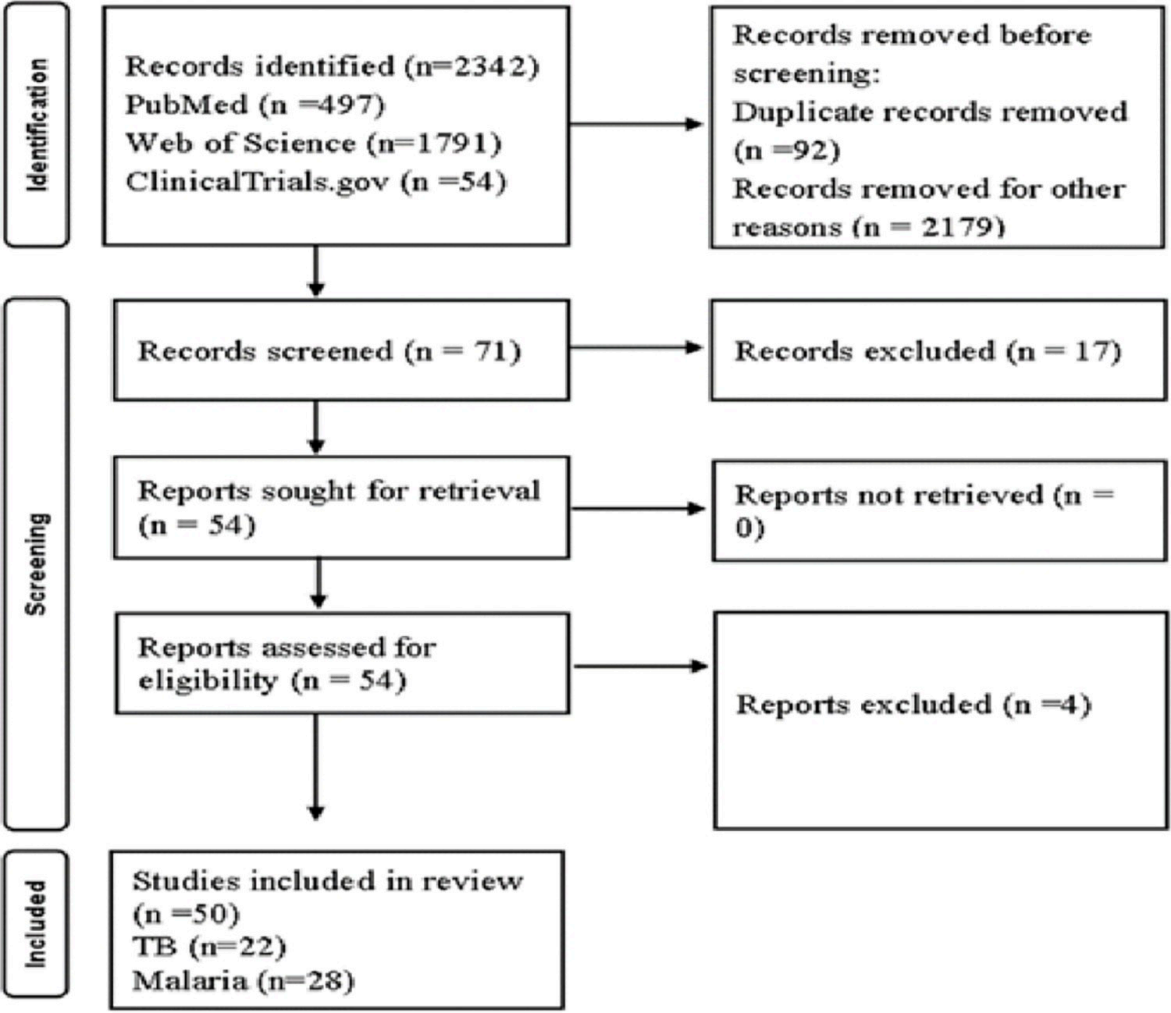
Article screening process based on PRISMA flowchart.

The search yielded 2342 publications S1 Table. 1791 (76.5%) articles were from Web of Sciences, 497 (21.2%) from PubMed, and 54 (2.3%) from ClincalTrials.gov. The ClincalTrials.gov database contained 54 articles from 11 studies (55%) of 20 search projects. To minimise bias, nine (45%) studies had no publications and were excluded from this review. The 2342 published studies were reduced to 92 (3.9%) duplicates and 2179 (93%) irrelevant. 17 (23.9%) studies were removed after screening titles and abstracts of 71 (3.0%). After full-text screening, 4 (7.4%) of 54 (76.1%) studies were removed. Fifty studies were eligible for review; 28 (56%) were malaria vaccines and 22 (44%) were TB vaccines. The flowchart of this study was summarized in Fig 1 and Table 2.

**Table 2:** Characteristics of all eligible studies in this review.

Author	Year	Country	Vaccine	Control	Phase	No of doses	Participants	Main Findings	References
	**Tuberculosis (TB)**								
**Churchyard et al.,**	2015	South Africa	AERAS- 402/A D35. TB-S	Placebo	II	2	Adults	Safe, CD4^+^, CD8^+^, antibodies	[27]
**Ndiaye et al.,**	2015	South Africa & Senegal	MVA 85A	Placebo	II	2	Adults	Safe, no protection, CD8^+^, CD4^+^	[24]
**Meeren et al.,**	2018	Zambia, Kenya, South Africa	M72/ AS10 E	Placebo	II	2	Adults	54% VE, safe	[32]
**Tait et al.,**	2019	Kenya, South Africa, & Zambia	M72/ AS01 E	Placebo	II	2	Adults	Safe, high CD4^+^, 49.7% VE, IgG	[38]
**Tameris et al.,**	2019	South Africa	MTBA V	BCG	I & II	3	Infants and adults	Higher CD4^+^, safe	[11]
**Walsh et al.,**	2016	Kenya	AERA S-402	Placebo	I	2	Adults	Safe, modest CD4^+^ & CD8^+^	[29]
**Tchakoute et al.,**	2014	South Africa	BCG	BCG	II	1	Infants	Higher CD4^+^ in delayed BCG	[39]
**Tameris et al.,**	2015	South Africa	AERA S-402	Placebo	II	3	Infants	Safe, low CD4^+^ and CD8^+^	[40]
**Penn-Nicholson et al.,**	2018	South Africa	ID93+ GLA- SE)	Placebo	I	2	Adults	Th1, IgG, safe	[41]
**Nell et al.,**	2014	South Africa	RUTI	Placebo	II	2	Adults	Safe, T-and humoral responses	[34]
**Lutwama et al.,**	2014	Uganda	BCG	Delayed	-	1	Infants	Higher CD4^+^ & CD8^+^ in early BCG	[42]
**Idoko et al.,**	2014	Gambia	M72/ AS01	Meningitis vaccine	II	2	Infants	Safe, higher CD4^+^ & IgG	[31]
**Nemes et al.,**	2018	South Africa	H4: IC 31& r BCG	Placebo	II	2	Adolescent s	Safe, higher CD4^+^, low VE	[43]
**Suliman et al.,**	2016	South Africa	BCG	Unvaccinated	I	1	Adults	Higher CD4, CD8, γδ T & NK cells	[44]
**Loxton et al.,**	2017	South Africa	VPM1 002	BCG	II	1	Infants	Safe, IL-17 secreting CD8^+^	[36]
**Tameris et al,**	2013	South Africa	MVA 85A	Placebo	IIb	1	Infants	Safe, low CD4^+^, low VE	[45]
**Bekker et al.,**	2020	South Africa	H4: IC 31, H56: I C31 & BCG	Placebo	1b	2	Adolescent s	Safe, higher CD4^+^, low CD8^+^	[30]
**Odutola et al.,**	2012	Gambia	MVA 85A	EPI	I	1	Infants	Safe, IFN-γ	[25]
**Geldenhuys et al.,**	2015	South Africa	BCG	-	I & II	1	Infants and adults	Safe, CD4^+^, CD8^+^	[46]
**Hesseling et al.,**	2015	South Africa	BCG	-	II	1	Infants	IFN-γ	[35]
**Kagina et al.,**	2014	South Africa	AERA S-402	Placebo	I	2	Infants	Safe, High CD4^+^, low CD8^+^	[28]
**Hatherill et al.,**	2014	South Africa	BCG	-	I	1	Adults	Safe	[47]
	**Malaria**								
**Bell et al.,**	2022	Ghana, Malawi, and Gabon	RTS, S/AS01/AS0 1	Placebo	III	3	Children	Waning VE due to transmission intensity	[10]
**Agnandji et al.,**	2014	7 Sub Saharan countries	RTS, S/AS01/AS0 1E	Meningococcol or rabies	III	3	Infants & children	Safe, partial VE	[48]
**Kimani et al.,**	2014	Kenya and Gambia	Chad 63 &MV A ME- TRAP	Placebo	Ib	2	Adults	CD4^+^, CD8^+^	[13]
**Partnership**	2012	7 Sub Saharan countries	RTS, S/AS01 & EPI	Meningococcol vaccine	III	3	Infants	Safe, partial VE, antibodies	[17]
**Dassah et al.,**	2021	Ghana, Uganda, Burkina Faso, and Gabon	GMZ2	Rabies	IIb	3	Children	Safe, low VE	[15]
**Otieno et al.,**	2020	7 Sub- Sahara countries	RTS, S/AS01/AS0 1	Meningococcol or rabies vaccine	III	3	Infants and children	Safe, antibodies	[49]
**Bejon et al.,**	2013	7 Sub Saharan countries	RTS, S/AS01A	Placebo/comparator vaccine	II	3	NA	Variable VE	[50]
**Oneko et al.,**	2021	Kenya	PfSPZ	Placebo	I & II	1	Infants	Low VE, Safe, undetectable T immune cells	[51]
**Bell et al.,**	2020	Malawi	RTS, S/AS01/AS0 1	Meningo coccol or rabies	III	4	Infants and children	Vegetation cover affects VE	[52]
**Ouédraogo et al.,**	2013	Burkina Faso	Ad35. CS.01	Placebo	Ib	3	Adults	Moderate IgG and neutralizing antibodies, safe	[12]
**Dejon-Agobe et al.,**	2019	Gabon	GMZ2	Rabies vaccine	NA	3	Adults	Safe, higher IgG, modest VE	[16]
**Dobaño et al.,**	2019	Tanzania, Burki Faso, and Ghana	RTS, S/AS01/AS0 1E	Comparator vaccine	III	3	Infants and children	Higher IgG associated with VE	[53]
**Mendoza et al.,**	2019	7 Sub Sahar an Africa	RTS, S/AS01	Menongococcol or rabies	III	4	Infants and children	Increased febrile convulsions	[54]
**Datoo et al.,**	2021	Burkina Faso	R21/MM	Rabies vaccine	IIb	3	Children	Safe, anti-NANP IgG, 77% VE	[55]
**Berry et al.,**	2019	Mali	FMP2.1/ AS02A	Rabies vaccine	II	3	Children	High IgG1, IgG2, IgG3, IgG4, no VE	[56]
**Sirima et al.,**	2017	Burkina Faso	(PfAMA1-DiCo)- GLA SE	Placebo	Ia/Ib	3	Adults	IgG, Th1/Th2, safe	[14]
**Moncunill et al.,**	2017	Africa	RTS, S/AS01E	Rabies vaccine	III	3	Children	Polyfunctional CD4^+^ cells	[18]
**Han et al.,**	2017	Malawi	RTS, S/AS01	Meningococcal & rabies vaccines	III	3–4	Infants and children	Seasonal precipitation has no effect on VE	[57]
**Sissoko et al.,**	2017	Mali	PfSPZ	Placebo	I	5	Adults	Safe, significant protection	[22]
**Mensah et al.,**	2016	Senegal	ChAd 63 & MVA ME-TRAP	Rabies vaccine	II	2	Male adults	T-cells, no VE, safe	[58]
**RTS, S partnership**	2015	7 Sub Saharan countries	RTS, S/AS01/AS0 1	Comparator vaccine	III	3	Infants and children	Safe, significant VE, antibodies	[20]
**Thera et al.,**	2016	Mali	PfAMA1- FVO	Tetanus vaccine	I	3	18–55 men and women	Safe, non-durable IgG	[59]
**Ubillos et al.,**	2018	Ghana and Mozambique	RTS, S/AS01E	NA	III	3	Infants and children	Protective IgG (1, 2, 3, &4) & IgM,	[60]
**Neafsey et al.,**	2015	7 Sub- Sahara Africa	RTS, S/AS01	Meningococcal vaccine	III	3	Infants and children	50.3% VE, genetic mismatch reduces protection	[61]
**Gyaase et al.,**	2021	Ghana	RTS, S/AS01	Meningococcal vaccine	III	3	Children	VE depended on SES	[62]
**Jongo et al.,**	2018	Tanzania	PfSPZ	Placebo	I	5	Men adults	Safe, antibodies, high CD4^+^, low CD8^+^, durable VE	[21]
**Chandramohan et al.,**	2021	Burkina Faso	RTS, S/AS01_E_	NA	III	3	Children	VE comparable to chemoprevention, safe	[63]
**Shekalaghe et al.,**	2014	Tanzania	PfSPZ	Placebo	I	2	Adult males	Safe	[23]

*7 Sub Saharan countries mean: Burkina Faso, Gabon, Ghana, Kenya, Malawi, Mozambique, and Tanzania.

*TST means Tubercle skin test.

*VE means vaccine efficacy.

*SES means socioeconomic status.

### 3.2 Quality assessment

**Fig 2:**
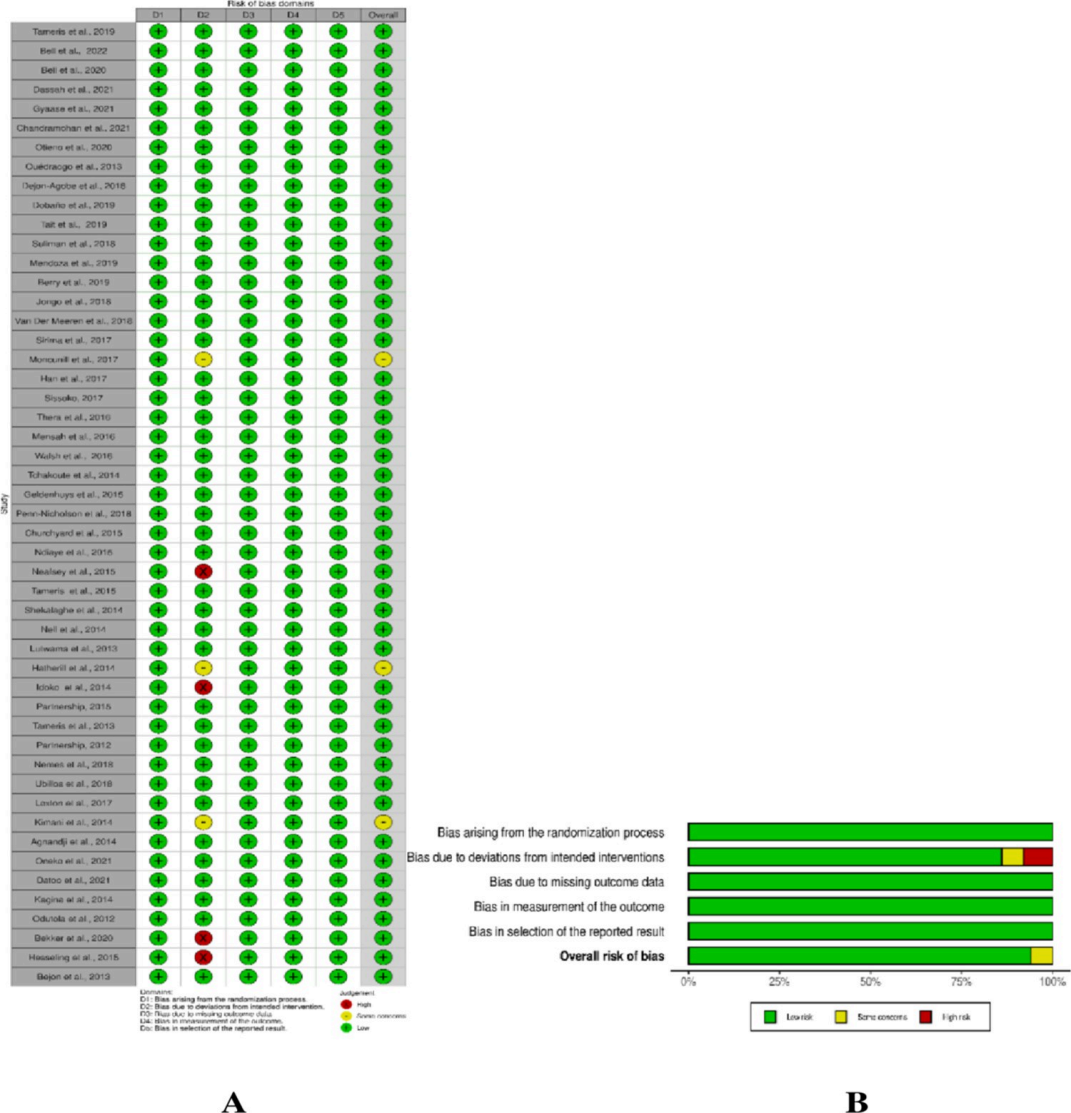
Risk assessment report for all eligible articles. (A) Dot plot showing quality of each article. (B) Overall report summary of all included articles.

The overall quality of all 50 eligible studies was good. 47 (94%) studies received “low risk” overall status for causing bias, 3 (6%) studies received “some concerns” status [13,18,47], and there were no articles with “high risk” overall status Fig 2.

### 3.3 Immunogenicity

In general, this review revealed that most malaria and TB vaccine candidates were immunogenic. However, the immune responses varied in terms of either magnitude, type of immune responses, or durability. For example, most malaria vaccine candidates had seven times the immunogenicity of humoral immune cells compared to CD4+ and CD8+ cells, which appeared only two times (7.14%). Conversely, TB vaccine candidates were more immunogenic to CD4+ T cells (72.7%) than CD8+ T cells (45.50%). However, in most cases, these immune responses were induced simultaneously. For example, CD4+ and CD8+ cells only [24,28,34,36,42,44,46], followed by CD4+, CD8+ cells, and antibodies only [27,29,30,40], and CD4+ cells and antibodies only [31,41]. Furthermore, few vaccine candidates induced only CD4+ cells [11,25,39], and none induced CD8+ cells only. Despite this diversity in both cases, challenges such as insignificant level of immune responses [40], lack of protection efficacy correlation [56], and poor durability [26,35,55] were observed. These findings demonstrate the immunogenic diversity of both malaria and TB vaccine candidates and the need for tailored vaccine design and evaluation.

### 3.4 Efficacy

Like in immunogenicity, efficacy differed based on the respective study. For instance, this review revealed that out of 28 malaria-related studies, only the R21/MM vaccine candidate (3.8%) had the highest vaccine efficacy (VE) of 77% [55], followed by RTS, S/AS01, 50.3% VE [61], 46% VE [48], 45% VE [50], 28% VE [20], 27% VE [48], 12% VE [51], and 18% VE [20], as well as 8% VE [58] in adults. Other malaria-related studies reported vaccine efficacy qualitatively [22,62,63]. On the other hand, the M72/AS01E TB vaccine candidate showed the highest vaccine efficacy, 54.0% VE [32], followed by 49.7% VE [38], 45.4% VE, and 30.5% VE [43]. In addition to the insignificant efficacy shown in both cases, other challenges such as lack of protection correlation [24,45,58] and the tendency of waning over time [15,50,57] were observed. These observations demonstrate that efficacy is not durable and thus requires continued research and development.

### 3.5 Safety

This review revealed that both malaria and TB vaccine candidates had no safety issues that resulted in the termination of the studies. However, adverse effects (AEs) like localized injection site swelling (9, 32.14%) and pains (8, 28.6%), whereas the systemic AEs included headache (12, 42.9%), myalgia, fever, and nausea (8, 28.6%) were reported in different malaria studies. Also in some cases, severe adverse effects (SAEs) such as malaria (8, 28.6%), meningitis, and febrile convulsion (7, 25%) were observed. Unlike in malaria vaccine candidates, injection site pains (5, 22.7%) and systemic abdominal pains (11, 50.0%) were the common AEs associated with TB vaccine candidates. Also, common SAE associated with TB vaccine candidates was found to be gastroenteritis (3, 13.6%) was the only common SAE. These results demonstrate that vaccine candidates’ safety issues are not the big challenges towards achieving the potent vaccines in sub-Saharan clinical trials.

## 4. Discussion

The systematic review of 50 studies highlights immunogenicity and efficacy as major challenges in sub-Saharan African malaria and TB vaccine clinical trials. However, safety should not be ignored in new vaccine development against these diseases since it is influenced by factors like dose [23,33,51,64], vaccine type [43], age group [54], and health status [27]. It is critical to evaluate the factors that contribute to variations in immunogenicity and efficacy during the development of vaccines against malaria and TB, especially in clinical trial settings. Complexities on vaccine candidates’ immunogenicity and efficacy arise because of factors such as population attributes, nature of vaccine candidates, vaccination strategy, and pathogen genetic diversity. Due to these complexities, drawing general conclusions on a particular vaccine candidate becomes hard, hence resulting in slow progress in sub-Saharan clinical trials.

In general, we discovered that population characteristics such as naturally acquired immunity, health status and socioeconomic factors affect greatly the drawing of general conclusions on vaccine candidate performance in clinical trials. Natural acquired immunity was found to influence the vaccine candidates’ performance antagonistically. For example, Kimani et al. showed that vaccine candidate performance was better in naïve adults than semi-immune adults [13]. Non-cytophilic and naturally acquired antibodies correlated with an increase in malaria infections and a reduction in the immunogenicity and efficacy of vaccine candidates [60]. This may explain why malaria is more common in older people [65]. Ideally, older age groups might have more non-cytophilic and naturally acquired antibodies than younger age groups due to the longer exposure time. Contrary to this ideology, many studies show that vaccine candidates’ performance is better in older ages than young ages [15,20,54,57] despite being variable among vaccinees of similar age groups [15,20,50]. These observations agree with those of the study conducted in Brazil that observed that naturally acquired antibodies were protective against parasites [66].

This review also observed that the health and socioeconomic status of the vaccinees played important roles in the vaccine candidates’ immunogenicity and efficacy. In general, HIV infections affected negatively the performance of both malaria and TB vaccine candidates regardless of the age group [24,27,49]. These findings are consistent with a previous study in which the vaccine candidate’s performance was low in comparison to the healthy group [19]. The reduced immune responses in HIV-infected participants might be due to the ability of HIV to cause immune response dysfunction. These differences complicate the drawing of general conclusions for a particular group. However, the effects of the coinfecting infection can be reduced by its respective chemotherapy [19,63]. Also, the socioeconomic status (SES) of the participating population played an important role in the vaccine candidates’ performance. For example, Gyaase et al. discovered that the decrease in vaccine efficacy was associated with a decrease in SES [62]. Since, poor SES may act as the precursor of higher malaria transmission intensity [62]. Studies show that higher infection transmission intensity reduces vaccine candidate performance [50] due to the rebound effect [10]. This condition of heightening naturally acquired immunity increases while vaccine-induced protection diminishes. The decrease in vaccine efficacy due to infection exposure was also reported by Olotu et al. [67]. This is contradictory to the studies that showed better vaccine candidate performance in older age groups than in young groups. Infection transmission intensity can also be facilitated by ecological factors such as vegetation [52] and wet season [57].

The vaccination strategy, such as the number of doses, vaccination timepoints, and routes of administration, influences significantly the immunogenicity and effectiveness of vaccine candidates. This review observed that BCG vaccinations in infants at different time points resulted in mixed observations. For example, vaccination at birth results in more distinct cellular immune responses than vaccination at 6 weeks [42] or vaccination at 14 weeks [35]. However, these differences were not observed when infants were vaccinated at birth and at 8 weeks after delivery [39]. Also, vaccine candidates may have different attributes due to the number of doses and route of administration. This review observed that most studies included employed multiple doses to keep up immunogenicity and efficacy steady [10,17,28,31,52,55]. In some cases, natural acquired immunity played a priming role [33]. However, other studies showed that number of doses had no effects on immunogenicity or efficacy [40,41,57]. The multiple doses strategy is worthy of recovering the diminishing protection. Despite this, the strategy may be an embargo to the accessibility of vaccines in low- or middle-income countries (LMICs), such as Sub-Saharan Africa, due to poverty.

This review revealed that the inferior performance of the vaccine candidates was associated with either poor immunogenicity or a narrow range of immune response inductions [30,43]. The low or missing of some immune responses may result in poor protection since immunogenicity is often related to the vaccine efficacy. Possibility, the nature, and formulations of the vaccine candidates were responsible. For example, CSP-based malaria vaccine candidates induced more anti-NANP antibodies than anti-C-terminal antibodies [53,55]. This might be because the NANP region is more conserved than the C-terminus, which can be reduced by removing antigen parts responsible for the induction of non-neutralizing antibodies [68]. However, the immune response distribution induced by Ad.35.CS.01 was influenced by CSP and Ad35 antigens [12]. Anti-CSP and anti-HB-specific IgG as well as IgM antibodies defined the efficacy of the RTS and S/AS01 vaccine candidates [60]. Despite being different from one endemic region to another, the failure to induce anti-HB antibodies decreased the vaccine candidate efficacy [17]. Also, the lack of PfSPZ vaccine candidate efficacy at 6 months postimmunization was associated with the lack of induction of cellular T immune responses [51]. This shows that multiple immune responses are needed for robust protection. Also, adjuvant formulations contributed to complexity in drawing conclusions. For example, the comparative studies that employed pairs of RTS, S/AS01 and RTS, S/AS02A [50], GMZ2/alum [15] and GMZ2/Alhydrogel/liposomes [16], and AMA1-DiCo GLA-SE and AMA1-DiCo Alhydrogel [14] resulted in different vaccine performance. However, similar differences were not observed during the GMZ2 Alhydorgel and GMZ2 CFA01 studies [16]. This underscores the need for pre-evaluation of adjuvants for conjugation.

Furthermore, pathogen genetic diversity is common in sub-Saharan Africa. This increases allelic mismatches between vaccine antigens and natural pathogens, which may affect vaccine efficacy [61]. The vaccine candidate might be immunogenic, but the immune responses would be non-specific to the natural parasite antigens. Surprisingly, studies show that allelic mismatches are more than 90% in Sub-Saharan Africa. This might be another major reason for the immunogenic vaccine candidates that failed to protect the vaccinees from the infection challenges.

Regardless of the disease, all vaccination studies included in this review employed only parenteral administration routes. This may have limited the induction of immune responses as well as protection efficacy. The mimicking of infection natural route plays an important role in effective protection against such infection. For example, the mucosal MVA85A immunization against TB in the United Kingdom induced strong immunogenicity, which was not observed after being administered through parenteral routes in South Africa [69]. This might be because of the ability of mucosal routes to induce speedy localized and systemic immune responses. Even though the malaria infections do not occur through mucosal surfaces, mucosal immunization against malaria protected the mice [70]. In addition to the immunological point of view, mucosal routes may be more economical than parenteral routes because they do not require trained personnel and needles and hence are appropriate in resource-limited sub-Saharan Africa.

The review acknowledges the missing of some publications as limitations of the review processes. These publications might have increased the scope of our results. However, the possibility to change the conclusion seems to be negligible due to being significantly small. Also, the absence of consistency in observations across studies has been a big challenge. This underscores the need for a comprehensive evaluation of the roles played by administration routes, vaccination timing, dosage, and the existence of confounding variables such as infection exposure, natural acquired immunity, age groups, infection transmission intensity, environments, as well as socioeconomic factors.

Despite this, WHO has recommended two malaria vaccines such as RTS, S/AS01E and R21/Matrix-M for vaccination of young children in sub-Saharan Africa after meeting some of the important WHO preferred product characteristics (PPCs) for malaria vaccines. Such characteristics include being safe, protective throughout the malaria season with the ability to reduce clinical malaria by 90%, and strongly immunogenic [71]. Unlike malaria, none of the novel TB vaccine candidates has been recommended for human vaccination. However, vaccine candidates like M72/AS01E have been shown to meet the PPCs for TB vaccines. In addition to the other two malaria PPCs, a novel TB vaccine must have at least 50% protection efficacy [72]. The vaccine candidate clinical trials show variability in immunogenicity and efficacy, as well as waning tendency. The ineffectiveness of specific vaccine candidates can be attributed to their inadequate immunogenicity, which means they elicit a restricted spectrum of immune responses. This underscores the criticality of refining the process of vaccine development and assessment. The review emphasizes the importance of comprehensive immune responses, which comprise humoral and cellular components, to provide strong protection against malaria and TB. Further investigation is warranted to tackle the identified obstacles, including but not limited to optimizing dosing regimens, enhancing the immunogenicity of vaccines that is specific to certain endemic regions and population groups, and investigating alternative delivery routes such as mucosal administration to enhance vaccine efficacy, particularly in settings with limited resources like sub-Saharan Africa.

## 5. Conclusion

This review revealed that immunogenicity and efficacy are the major challenges for both malaria and TB vaccine candidates. The challenges were orchestrated by population characteristics, vaccination strategies, and pathogen genetic diversity. This review suggests that the continued neglect of these factors could lengthen the journey toward robust vaccines, that mucosal routes may improve vaccine candidate performance, and that the development of endemic region-based vaccines is worthwhile.

## Supporting information

S1 Table List of all articles identified and reasons for exclusion of some articles. (DOCM)

S2 Table Eligible studies, extracted data, extractors, period of data extraction, and reasons for inclusion. (DOCX)

S1 File PRISMA 2020 checklist. (PDF)

S2 File (DOCM)
