# Supplementary material for: A systematic review on malaria and Tuberculosis (TB) vaccine challenges in sub-Saharan African clinical trials
Source: PLoS One. 2025 Jan 24;20(1):e0317233. doi: 10.1371/journal.pone.0317233 (PMC11760592; doi:10.1371/journal.pone.0317233)
Supplement: S1 Table — (DOCM) [file pone.0317233.s001.docm]

S1 Table: List of all articles identified and reasons for exclusion of some articles.

| Sn | Authors | Year | Tittle | Reason for rejection |
| --- | --- | --- | --- | --- |
|  | Granta et al., | 2022 | Impact of seasonal RTS, S/AS01E vaccination plus seasonal malaria chemoprevention on the nutritional status of children in Burkina Faso and Mali | Malnutrition study |
|  | Xu et al | 2012 | Estimation of summary protective efficacy using a frailty mixture model for recurrent event time data | Not related to immunogenicity, efficacy, and safety of TB/Malaria vaccine candidate |
|  | Cairns et al., | 2015 | Analysis of Preventive Interventions for Malaria: Exploring Partial and Complete Protection and Total and Primary Intervention Effects | Not related to immunogenicity, efficacy, and safety of TB/Malaria vaccine candidate |
|  | Warhurst | 2001 | A molecular marker for chloroquine-resistant falciparum malaria. | Publication year and not related to immunogenicity, efficacy, and safety of TB/Malaria vaccine candidate |
|  | Mordmüller et al., | 2017 | Sterile protection against human malaria by chemoattenuated PfSPZ vaccine | Non-sub-Saharan African study |
|  | Bijker et al., | 2015 | Novel approaches to whole sporozoite vaccination against malaria | Not related to immunogenicity, efficacy, and safety of TB/Malaria vaccine candidate |
|  | Swysen et al., | 2011 | Development of standardized laboratory methods and quality processes for a phase III study of the RTS, S/AS01 candidate malaria vaccine | Publication year |
|  | Penny et al., | 2016 | Public health impact and cost-effectiveness of the RTS, S/AS01 malaria vaccine: a systematic comparison of predictions from four mathematical models | Not related to immunogenicity, efficacy, and safety of TB/Malaria vaccine candidate |
|  | Cairns et al., | 2012 | Estimating the potential public health impact of seasonal malaria chemoprevention in African children | Not related to immunogenicity, efficacy, and safety of TB/Malaria vaccine candidate |
|  | Bell et al., | 2022 | Malaria Transmission Intensity Likely Modifies RTS, S/AS01 Efficacy Due to a Rebound Effect in Ghana, Malawi, and Gabon | Included |
|  | Tameris et al., | 2019 | Live-attenuated Mycobacterium tuberculosis vaccine MTBVAC versus BCG in adults and neonates: a randomised controlled, double-blind dose-escalation trial | Included |
|  | Ouédraogo et al., | 2013 | A phase 1b randomized, controlled, double-blinded dosage-escalation trial to evaluate the safety, reactogenicity and immunogenicity of an adenovirus type 35 based circumsporozoite malaria vaccine in burkinabe healthy adults 18 to 45 years of age | Included |
|  | Kimani et al., | 2014 | Translating the Immunogenicity of Prime-boost Immunization With ChAd63 and MVA ME-TRAP From Malaria Naive to Malaria-endemic Populations | Included |
|  | Sirima et al., | 2017 | Safety and immunogenicity of a recombinant Plasmodium falciparum AMA1-DiCo malaria vaccine adjuvanted with GLA-SE or Alhydrogel® in European and African adults: A phase 1a/1b, randomized, double-blind multi-centre trial | Included |
|  | Dassah et al., | 2021 | Extended follow-up of children in a phase2b trial of the GMZ2 malaria vaccine | Included |
|  | Dejon-Agobe et al., | 2019 | Controlled Human Malaria Infection of Healthy Adults with Lifelong Malaria Exposure to Assess Safety, Immunogenicity, and Efficacy of the Asexual Blood Stage Malaria Vaccine Candidate GMZ2 | Included, duplicate |
|  | Partnerships | 2012 | A phase 3 trial of RTS, S/AS01 malaria vaccine in African infants | Included, duplicate |
|  | Moncunill et al., | 2017 | RTS,S/AS01E Malaria Vaccine Induces Memory and Polyfunctional T Cell Responses in a Pediatric African Phase III Trial | Included |
|  | Ndiaye et al., | 2016 | Safety of Seasonal Malaria Chemoprevention (SMC) with Sulfadoxine-Pyrimethamine plus Amodiaquine when Delivered to Children under 10 Years of Age by District Health Services in Senegal: Results from a Stepped-Wedge Cluster Randomized Trial | Not related to immunogenicity, efficacy, and safety of TB/Malaria vaccine candidate |
|  | RTS, S partnership | 2015 | Efficacy and safety of RTS, S/AS01 malaria vaccine with or without a booster dose in infants and children in Africa: final results of a phase 3, individually randomised, controlled trial | Duplicate, included |
|  | Jongo et al., | 2018 | Safety, immunogenicity, and protective efficacy against controlled human malaria infection of plasmodium falciparum sporozoite vaccine in Tanzanian adults | Included |
|  | Sissoko et al., | 2017 | Safety and efficacy of PfSPZ Vaccine against Plasmodium falciparum via direct venous inoculation in healthy malaria-exposed adults in Mali: a randomised, double-blind phase 1 trial | Included |
|  | Shekalaghe et al.,f | 2014 | Controlled human malaria infection of Tanzanians by intradermal injection of aseptic, purified, cryopreserved plasmodium falciparum sporozoites | Included |
|  | Ndiaye et al., | 2015 | Safety, immunogenicity, and efficacy of the candidate tuberculosis vaccine MVA85A in healthy adults infected with HIV-1: a randomised, placebo-controlled, phase 2 trial | Included, duplicate |
|  | Odutola et al., | 2012 | A new TB vaccine, MVA85A, induces durable antigen-specific responses 14 months after vaccination in African infants | Duplicate, included |
|  | Regules et al., | 2016 | Fractional Third and Fourth Dose of RTS, S/AS01 Malaria Candidate Vaccine: A Phase 2a Controlled Human Malaria Parasite Infection and Immunogenicity Study | Non-sub-Saharan African study |
|  | Churchyard et al., | 2015 | The safety and immunogenicity of an adenovirus type 35-vectored TB vaccine in HIV-infected, BCG-vaccinated adults with CD4(+) T cell counts >350 cells/mm^3^ | Included, duplicate |
|  | Kagina et al., | 2014 | The novel tuberculosis vaccine, AERAS-402, is safe in healthy infants previously vaccinated with BCG, and induces dose-dependent CD4 and CD8T cell responses | Duplicate, included |
|  | Walsh et al., | 2016 | Adenovirus type 35-vectored tuberculosis vaccine has an acceptable safety and tolerability profile in healthy, BCG-vaccinated, QuantiFERON (R)-TB Gold (+) Kenyan adults without evidence of tuberculosis | Duplicate, included |
|  | Bekker et al., | 2020 | A phase lb randomized study of the safety and immunological responses to vaccination with H4:IC31, H56:IC31, and BCG revaccination in Mycobacterium tuberculosis-uninfected adolescents in Cape Town, South Africa | Included |
|  | Idoko et al., | 2014 | Safety and immunogenicity of the M72/AS01 candidate tuberculosis vaccine when given as a booster to BCG in Gambian infants: an open label randomized controlled trial | Duplicate, included |
|  | Meeren et al., | 2018 | Phase 2b Controlled Trial of M72/AS01E Vaccine to Prevent Tuberculosis | Included |
|  | Dahan et al., | 2012 | Comparison of trabeculectomy and Ex-PRESS implantation in fellow eyes of the same patient: a prospective, randomised study. | Not related to immunogenicity, efficacy, and safety of TB/Malaria vaccine candidate |
|  | Nell et al., | 2014 | Safety, tolerability, and immunogenicity of the novel antituberculous vaccine RUTI: Randomized, placebo-controlled phase II clinical trial in patients with latent tuberculosis infection | Duplicate, included |
|  | Hesseling et al., | 2015 | Immunogenicity of BCG in HIV-exposed and non-exposed infants following routine birth or delayed vaccination | Duplicate, included |
|  | Loxton et al., | 2017 | Safety and Immunogenicity of the Recombinant Mycobacterium bovis BCG Vaccine VPM1002 in HIV-Unexposed Newborn Infants in South Africa | Duplicate, included |
|  | Mihret et al., | 2012 | Plasma Level of IL-4 Differs in Patients Infected with Different Modern  Lineages of M. tuberculosis | Not related to immunogenicity, efficacy, and safety of TB/Malaria vaccine candidate |
|  | Tait et al., | 2019 | Final Analysis of a Trial of M72/AS01 E Vaccine to Prevent Tuberculosis | Included, duplicate |
|  | Tchakoute et al., | 2014 | Delaying BCG vaccination until 8 weeks of age results in robust BCG-specific T-cell responses in HIV-exposed infants | Included |
|  | Tameris et al., | 2015 | A double-blind, randomised, placebo-controlled, dose-finding trial of the novel tuberculosis vaccine AERAS-402, an adenovirus-vectored fusion protein, in healthy, BCG-vaccinated infants | Duplicate, included |
|  | Penn-Nicholson et al., | 2018 | Safety and immunogenicity of the novel tuberculosis vaccine ID93 + GLA-SE in BCG-vaccinated healthy adults in South Africa: a randomised, double-blind, placebo-controlled phase 1 trial | Duplicate, included |
|  | Lutwama et al., | 2014 | Distinct T-Cell Responses When BCG Vaccination Is Delayed from Birth to 6 Weeks of Age in Ugandan Infants | Duplicate, included |
|  | Nemes et al., | 2018 | Prevention of M. tuberculosis Infection with H4:IC31 Vaccine or BCG Revaccination | Included |
|  | Suliman et al., | 2016 | Bacillus Calmette-Guerin (BCG) Revaccination of Adults with Latent Mycobacterium tuberculosis Infection Induces Long-Lived BCG-Reactive NK  Cell Responses | Duplicate, included |
|  | Tameris et al., | 2013 | Safety and efficacy of MVA85A, a new tuberculosis vaccine, in infants previously vaccinated with BCG: A randomised, placebo-controlled phase 2b trial | Included |
|  | Geldenhuys et al., | 2015 | A randomized clinical trial in adults and newborns in South Africa to compare the safety and immunogenicity of bacille Calmette-Guérin (BCG) vaccine administration via a disposable-syringe jet injector to conventional technique with needle and syringe | Duplicate, included |
|  | Hatherill et al., | 2014 | Safety and reactogenicity of BCG revaccination with isoniazid pretreatment in TST positive adults | Duplicate, included |
|  | Agnandji et al., | 2014 | Efficacy and Safety of the RTS, S/AS01 Malaria Vaccine during 18 Months after Vaccination: A Phase 3 Randomized, Controlled Trial in Children and Young Infants at 11 African Sites | Duplicate, included |
|  | Otieno et al., | 2020 | Safety and immunogenicity of the RTS, S/AS01 malaria vaccine in infants and children identified as HIV-infected during a randomized trial in sub-Saharan Africa | Duplicate, included |
|  | Bejon et al., | 2013 | Efficacy of RTS,S malaria vaccines: individual-participant pooled analysis of phase 2 data | Included |
|  | Oneko et al., | 2021 | Safety, immunogenicity and efficacy of PfSPZ Vaccine against malaria in infants in western Kenya: a double-blind, randomized, placebo-controlled phase 2 trial | Included |
|  | Bell et al. | 2020 | Environmental modifiers of RTS, S/AS01 malaria vaccine efficacy in Lilongwe, Malawi | Duplicate, included |
|  | Dobaño et al., | 2019 | Concentration and avidity of antibodies to different circumsporozoite epitopes correlate with RTS,S/AS01E malaria vaccine efficacy | Included |
|  | Mendoza et al., | 2019 | Safety profile of the RTS, S/AS01 malaria vaccine in infants and children: additional data from a phase III randomized controlled trial in sub-Saharan Africa | Duplicate, included |
|  | Datoo et al., | 2019 | Efficacy of a low-dose candidate malaria vaccine, R21 in adjuvant Matrix-M, with seasonal administration to children in Burkina Faso: a randomised controlled trial | Included |
|  | Berry et al., | 2019 | Immunoglobulin G subclass and antibody avidity responses in Malian children immunized with Plasmodium falciparum apical membrane antigen 1 vaccine candidate FMP2.1/AS02(A) | Included |
|  | O’leary et al., | 2012 | Moderation and mediation of an effective HIV risk-Reduction intervention for south African adolescents | Not related to immunogenicity, efficacy, and safety of TB/Malaria vaccine candidate |
|  | Mensah et al., | 2016 | Safety, immunogenicity and efficacy of prime-Boost vaccination with chad63 and mva encoding me-trap against plasmodium falciparum infection in adults in senegal | Included |
|  | Thera et al., | 2016 | Phase 1 randomized controlled trial to evaluate the safety and immunogenicity of recombinant Pichia pastoris-expressed Plasmodium falciparum apical membrane antigen 1 (PfAMA1-FVO [25-545]) in healthy Malian adults in Bandiagara | Included |
|  | Buldeo et al., | 2012 | Pulmonary Immune-Compartment-Specific Interferon Gamma Responses in  HIV-Infected Individuals with Active Tuberculosis (TB) in an Area of  High TB Prevalence | Not related to immunogenicity, efficacy, and safety of TB/Malaria vaccine candidate |
|  | Ubillos et al., | 2018 | Baseline exposure, antibody subclass, and hepatitis B response differentially affect malaria protective immunity following RTS,S/AS01E vaccination in African children | Included |
|  | Neafsey et al., | 2015 | Genetic Diversity and Protective Efficacy of the RTS, S/AS01 Malaria Vaccine | Duplicate, included |
|  | Gyaase et al., | 2021 | Potential effect modification of RTS, S/AS01 malaria vaccine efficacy by household socio-economic status | Included, duplicate |
|  | Chandramohan et al., | 2020 | Seasonal Malaria Vaccination with or without Seasonal Malaria Chemoprevention | Included, duplicate |
|  | Dicko et al., | 2011 | Intermittent preventive treatment of malaria provides substantial protection against malaria in children already protected by an insecticide-treated bed net in Mali: a randomised, double-blind, placebo-controlled trial | Publication year and not related to immunogenicity, efficacy, and safety of TB/Malaria vaccine candidate |
|  | Lin et al., | 2012 | CD4 T Cell Depletion Exacerbates Acute Mycobacterium Tuberculosis While  Reactivation of Latent Infection Is Dependent on Severity of Tissue Depletion in Cynomolgus Macaques | Not related to immunogenicity, efficacy, and safety of TB/Malaria vaccine candidate |
|  | Wilson et al., | 2011 | A systematic review and meta-analysis of the efficacy and safety of intermittent preventive treatment of malaria in children (IPTc) | Not related to immunogenicity, efficacy, and safety of TB/Malaria vaccine candidate and publication year |
|  | Konaté et al., | 2011 | Intermittent preventive treatment of malaria provides substantial protection against malaria in children already protected by an insecticide-treated bed net in Burkina Faso: a randomised, double-blind, placebo-controlled trial | Not related to immunogenicity, efficacy, and safety of TB/Malaria vaccine candidate and publication year |
|  | Mohamed and Fourie | 2012 | Clinical trials of TB vaccines: Harmonization and cooperation | Not related to immunogenicity, efficacy, and safety of TB/Malaria vaccine candidate |
|  | Limmahakhun et al., | 2012 | Treatment outcomes of patients co-infected with tuberculosis and HIV at Chiang Mai University Hospital, Thailand | Not related to immunogenicity, efficacy, and safety of TB/Malaria vaccine candidate, non-sub-Saharan study |
|  | White et al., | 2015 | Immunogenicity of the RTS, S/AS01 malaria vaccine and implications for duration of vaccine efficacy: secondary analysis of data from a phase 3 randomised controlled trial | Not related to immunogenicity, efficacy, and safety of TB/Malaria vaccine candidate |
|  | Cohen et al., | 2010 | From the circumsporozoite protein to the RTS, S/AS candidate vaccine | Not related to immunogenicity, efficacy, and safety of TB/Malaria vaccine candidate and publication year |
|  | Sagawa et al., | 2023 | Safety and immunogenicity of a thermostable ID93 + GLA-SE tuberculosis vaccine candidate in healthy adults | Non-sub–Saharan African study |
|  | Luabeya et al., | 2015 | First-in-human trial of the post-exposure tuberculosis vaccine H56:IC31 in Mycobacterium tuberculosis infected and non-infected healthy adults | Non-randomised clinical trial |
|  | Mulenga et al., | 2015 | The Role of Clinical Symptoms in the Diagnosis of Intrathoracic Tuberculosis in Young Children | Not related to immunogenicity, efficacy, and safety of TB/Malaria vaccine candidate |
|  | Waggie et al., | 2012 | Randomized trial of type 1 and type 3 oral monovalent poliovirus vaccines in Newborns in Africa | Non-malaria/ TB clinical study |
|  | Müller et al., | 2019 | Cytomegalovirus infection is a risk factor for tuberculosis disease in infants | Not related to immunogenicity, efficacy, and safety of TB/Malaria vaccine candidate |
|  | Tameris et al., | 2013 | Lessons learnt from the first efficacy trial of a new infant tuberculosis vaccine since BCG | Not related to immunogenicity, efficacy, and safety of TB/Malaria vaccine candida |
|  | Luabeya et al., | 2015 | Risk of Disease After Isoniazid Preventive Therapy for Mycobacterium tuberculosis Exposure in Young HIV-uninfected Children | Not related to immunogenicity, efficacy, and safety of TB/Malaria vaccine candidate |
|  | Bunyasi et al., | 2015 | Evaluation of Xpert® MTB/RIF Assay in Induced Sputum and Gastric Lavage Samples from Young Children with Suspected Tuberculosis from the MVA85A TB Vaccine Trial | Not related to immunogenicity, efficacy, and safety of TB/Malaria vaccine candidate |
|  | Rose et al., | 2012 | Linezolid-containing regimens for the treatment of drug-resistant tuberculosis in South African children | Not related to immunogenicity, efficacy, and safety of TB/Malaria vaccine candidate |
|  | Beksinska et al., | 2012 | Practice makes perfect: Reduction in female condom failures and user problems with short-term experience in a randomized trial | Not related to immunogenicity, efficacy, and safety of TB/Malaria vaccine candidate |
|  | Matsumiya et al., | 2014 | Inflammatory and myeloid-associated gene expression before and one day after infant vaccination with MVA85A correlates with induction of a T cell response | Not related to immunogenicity, efficacy, and safety of TB/Malaria vaccine candidate |
|  | Farrance et al., | 2011 | A plant-produced Pfs230 vaccine candidate blocks transmission of Plasmodium falciparum | Pre-clinical study and publication year |
|  | MacDonald et al., | 2016 | Structural and Immunological Characterization of Recombinant 6-Cysteine Domains of the Plasmodium falciparum Sexual Stage Protein Pfs230 | Not related to immunogenicity, efficacy, and safety of TB/Malaria vaccine candidate |
|  | Wu et al., | 2006 | Sustained high-titer antibody responses induced by conjugating a malarial vaccine candidate to outer-membrane protein complex | Not related to immunogenicity, efficacy, and safety of TB/Malaria vaccine candidate |
|  | Tachibana et al., | 2011 | N-terminal prodomain of Pfs230 synthesized using a cell-free system is sufficient to induce complement-dependent malaria transmission-blocking activity | Publication year, and non-clinical study |
|  | Diallo et al., | 2008 | Evaluation and optimization of membrane feeding compared to direct feeding as an assay for infectivity | Non-clinical study and publication year |
|  | Thera et al., | 2010 | Safety and immunogenicity of an AMA1 malaria vaccine in Malian children: results of a phase 1 randomized controlled trial | Publication year |
|  | Valéa et al., | 2018 | Immune response to the hepatitis B antigen in the RTS, S/AS01 malaria vaccine, and co-administration with pneumococcal conjugate and rotavirus vaccines in African children: A randomized controlled trial | Non-TB/malaria vaccine objectives |
|  | Chichester et al., | 2018 | Safety and immunogenicity of a plant-produced Pfs25 virus-like particle as a transmission blocking vaccine against malaria: A Phase 1 dose-escalation study in healthy adults | Non-sub-Saharan study |
|  | Van den Berg et al., | 2018 | Adjuvant-Associated Peripheral Blood mRNA Profiles and Kinetics Induced by the Adjuvanted Recombinant Protein Candidate Tuberculosis Vaccine M72/AS01 in Bacillus Calmette-Guérin-Vaccinated Adults | Non-sub-Saharan Africa study |
|  | Aspinall et al., | 2012 | Lot-to-lot consistency study of the fully liquid pentavalent DTwP-HepB-Hib vaccine Quinvaxem® demonstrating clinical equivalence, suitability of the vaccine as a booster and concomitant administration with measles vaccine | Not related to immunogenicity, efficacy, and safety of TB/Malaria vaccine candidate |
|  | Aide et al., | 2010 | Safety, immunogenicity, and duration of protection of the RTS, S/AS02(D) malaria vaccine: one year follow-up of a randomized controlled phase I/IIb trial | Publication year |
|  | Aponte et al., | 2007 | Safety of the RTS, S/AS02D candidate malaria vaccine in infants living in a highly endemic area of Mozambique: a double blind randomised controlled phase I/IIb trial | Publication year |
|  | Angov et al., | 2003 | Development and pre-clinical analysis of a Plasmodium falciparum Merozoite Surface Protein-142 malaria vaccine | Publication year and non-clinical study |
|  | Ockenhouse et al., | 2006 | Phase I safety and immunogenicity trial of FMP1/AS02A, a Plasmodium falciparum MSP-1 asexual blood stage vaccine | Publication year and non-sub-Saharan Africa |
|  | Stoute | 2007 | Phase 1 randomized double-blind safety and immunogenicity trial of Plasmodium falciparum malaria merozoite surface protein FMP1 vaccine, adjuvanted with AS02A, in adults in western Kenya | Publication year |
|  | Ophorst et al., | 2006 | Immunogenicity and protection of a recombinant human adenovirus serotype 35-based malaria vaccine against Plasmodium yoelii in mice | Pre-clinical study and publication year |
|  | Rodrigues et al., | 1998 | Efficient induction of protective anti-malaria immunity by recombinant adenovirus | Pre-clinical study and publication year |
|  | Sedegah et al., | 2016 | Vaccine Strain-Specificity of Protective HLA-Restricted Class 1 P. falciparum Epitopes | Non-sub-Saharan Africa study |
|  | Sedegah et al., | 2015 | Controlled Human Malaria Infection (CHMI) differentially affects cell-mediated and antibody responses to CSP and AMA1 induced by adenovirus vaccines with and without DNA-priming | Non-sub-Saharan Africa study |
|  | Li et al., | 2007 | Viral vectors for malaria vaccine development | Publication year and not related to immunogenicity, efficacy, and safety of TB/Malaria vaccine candidate |
|  | Tamminga et al., | 2011 | Adenovirus-5-vectored P. falciparum vaccine expressing CSP and AMA1. Part B: safety, immunogenicity, and protective efficacy of the CSP component | Publication year |
|  | Moon et al., | 2020 | A Phase IIa Controlled Human Malaria Infection and Immunogenicity Study of RTS, S/AS01E and RTS, S/AS01B Delayed Fractional Dose Regimens in Malaria-Naive Adults | Non-sub-Saharan African study |
|  | Jewkes et al., | 2012 | Motivations for, and perceptions and experiences of participating in, a cluster randomised controlled trial of a HIV-behavioural intervention in rural South Africa | Not related to immunogenicity, efficacy, and safety of TB/Malaria vaccine candidate |
|  | Aguirar et al., | 2015 | Discovery of Novel Plasmodium falciparum Pre-Erythrocytic Antigens for Vaccine Development | Not related to immunogenicity, efficacy, and safety of TB/Malaria vaccine candidate |
|  | Sedegah et al., | 2014 | Sterile immunity to malaria after DNA prime/adenovirus boost immunization is associated with effector memory CD8+T cells targeting AMA1 class I epitopes | Non-sub-Saharan Africa study |
|  | Tamminga et al., | 2013 | Human adenovirus 5-vectored Plasmodium falciparum NMRC-M3V-Ad-PfCA vaccine encoding CSP and AMA1 is safe, well-tolerated and immunogenic but does not protect against controlled human malaria infection | Non-sub-Saharan Africa study |
|  | Abdulla et al., | 2008 | Safety and immunogenicity of RTS, S/AS02D malaria vaccine in infants | Publication year |
|  | Du Plessis et al., | 2012 | In vitro activity of Pheroid vesicles containing antibiotics against Plasmodium falciparum | Not related to immunogenicity, efficacy, and safety of TB/Malaria vaccine candidate |
|  | Cardona et al., | 2005 | Immunotherapy with fragmented Mycobacterium tuberculosis cells increases the effectiveness of chemotherapy against a chronical infection in a murine model of tuberculosis | Not related to immunogenicity, efficacy, and safety of TB/Malaria vaccine candidate and publication year |
|  | Seedat et al., | 2012 | Developing the evidence-base for Safe Communities: A multi-level, partly randomised, controlled trial | Not related to immunogenicity, efficacy, and safety of TB/Malaria vaccine candidate |
|  | Gil et al., | 2008 | Enhanced gamma interferon responses of mouse spleen cells following immunotherapy for tuberculosis relapse | Not related to immunogenicity, efficacy, and safety of TB/Malaria vaccine candidate and publication year |
|  | Guirado et al., | 2008 | Induction of a specific strong polyantigenic cellular immune response after short-term chemotherapy controls bacillary reactivation in murine and guinea pig experimental models of tuberculosis | Publication year and non-clinical study |
|  | Vilaplana et al., | 2008 | The tuberculin skin test increases the responses measured by T cell interferon-gamma release assays | Not related to immunogenicity, efficacy, and safety of TB/Malaria vaccine candidate and publication year |
|  | Cardona | 2006 | RUTI: a new chance to shorten the treatment of latent tuberculosis infection | Not related to immunogenicity, efficacy, and safety of TB/Malaria vaccine candidate and publication year |
|  | Cardona and Amat | 2006 | Origin and development of RUTI, a new therapeutic vaccine against Mycobacterium tuberculosis infection | Not related to immunogenicity, efficacy, and safety of TB/Malaria vaccine candidate and publication year |
|  | Moncunill et al., | 2022 | Transcriptional correlates of malaria in RTS, S/AS01-vaccinated African children: a matched case-control study | Non-randomized study |
|  | Ward et al., | 2018 | The Ethics of End-of-Trial Obligations in a Pediatric Malaria Vaccine Trial: The Perspectives of Stakeholders from Ghana and Tanzania | Not related to immunogenicity, efficacy, and safety of TB/Malaria vaccine candidate |
|  | Vandoolaeghe  And Schuerman | 2016 | The RTS, S/AS01 malaria vaccine in children 5 to 17 months of age at first vaccination | Not related to immunogenicity, efficacy, and safety of TB/Malaria vaccine candidate |
|  | Swysen et al., | 2011 | Development of standardized laboratory methods and quality processes for a phase III study of the RTS, S/AS01 candidate malaria vaccine | Not related to immunogenicity, efficacy, and safety of TB/Malaria vaccine candidate and publication year |
|  | Leach et al., | 2011 | Design of a phase III multicenter trial to evaluate the efficacy of the RTS, S/AS01 malaria vaccine in children across diverse transmission settings in Africa | Publication year and not related to immunogenicity, efficacy, and safety of TB/Malaria vaccine candidate |
|  | Lievens et al., | 2011 | Statistical methodology for the evaluation of vaccine efficacy in a phase III multi-centre trial of the RTS, S/AS01 malaria vaccine in African children | Publication year and not related to immunogenicity, efficacy, and safety of TB/Malaria vaccine candidate |
|  | Vekemans et al., | 2011 | Assessment of severe malaria in a multicenter, phase III, RTS, S/AS01 malaria candidate vaccine trial: case definition, standardization of data collection and patient care | Not related to immunogenicity, efficacy, and safety of TB/Malaria vaccine candidate, publication year |
|  | Ward et al., | 2018 | The Ethics of Health Care Delivery in a Pediatric Malaria Vaccine Trial: The Perspectives of Stakeholders from Ghana and Tanzania | Not related to immunogenicity, efficacy, and safety of TB/Malaria vaccine candidate |
|  | Ward et al., | 2018 | Defining Health Research for Development: The perspective of stakeholders from an international health research partnership in Ghana and Tanzania | Not related to immunogenicity, efficacy, and safety of TB/Malaria vaccine candidate, |
|  | Sauboin et al., | 2015 | Potential public health impact of RTS, S malaria candidate vaccine in sub-Saharan Africa: a modelling study | Not related to immunogenicity, efficacy, and safety of TB/Malaria vaccine candidate |
|  | Angwenyi et al., | 2014 | Complex realities: community engagement for a paediatric randomized controlled malaria vaccine trial in Kilifi, Kenya | Not related to immunogenicity, efficacy, and safety of TB/Malaria vaccine candidate |
|  | Dutta et al., | 2002 | Purification, characterization, and immunogenicity of the refolded ectodomain of the Plasmodium falciparum apical membrane antigen 1 expressed in Escherichia coli | Publication year and not related to immunogenicity, efficacy, and safety of TB/Malaria vaccine candidate |
|  | Polhemus et al., | 2007 | Phase I dose escalation safety and immunogenicity trial of Plasmodium falciparum apical membrane protein (AMA-1) FMP2.1, adjuvanted with AS02A, in malaria-naïve adults at the Walter Reed Army Institute of Research | Publication year |
|  | Scriba et al., | 2012 | A Phase IIa Trial of the New Tuberculosis Vaccine, MVA85A, in HIV-and/or Mycobacterium tuberculosis-infected Adults | Non-randomised study |
|  | Ritz et al., | 2012 | The Influence of Bacille Calmette-Guerin Vaccine Strain on the Immune Response against Tuberculosis: A Randomized Trial | Non-sub-Saharan Africa study |
|  | Kassa et al., | 2012 | Analysis of Immune Responses against a Wide Range of Mycobacterium tuberculosis Antigens in Patients with Active Pulmonary Tuberculosis | Not related to immunogenicity, efficacy, and safety of TB/Malaria vaccine candidate |
|  | Thera et al., | 2011 | A field trial to assess a blood-stage malaria vaccine | Publication year |
|  | Hill et al., | 1992 | Molecular analysis of the association of HLA-B53 and resistance to severe malaria | Publication year |
|  | Agnandji et al., | 2011 | First results of phase 3 trial of RTS, S/AS01 malaria vaccine in African children | Publication year |
|  | Hill et al., | 1991 | Common west African HLA antigens are associated with protection from severe malaria | Publication year |
|  | Von Reyn et al., | 2012 | New vaccines for the prevention of tuberculosis in human immunodeficiency virus infection | Not related to immunogenicity, efficacy, and safety of TB/Malaria vaccine candidate |
|  | McConkeys et al., | 2003 | Enhanced T-cell immunogenicity of plasmid DNA vaccines boosted by recombinant modified vaccinia virus Ankara in humans | Publication year |
|  | Ewer et al., | 2013 | Protective CD8+ T-cell immunity to human malaria induced by chimpanzee adenovirus-MVA immunisation | Non-sub-Saharan African study |
|  | Webster et al., | 2005 | Enhanced T cell-mediated protection against malaria in human challenges by using the recombinant poxviruses FP9 and modified vaccinia virus Ankara | Publication year |
|  | Nussenzweig et al., | 1967 | Protective immunity produced by the injection of x-irradiated sporozoites of plasmodium berghei | Publication time |
|  | Regules et al., | 2016 | Fractional Third and Fourth Dose of RTS, S/AS01 Malaria Candidate Vaccine: A Phase 2a Controlled Human Malaria Parasite Infection and Immunogenicity Study | Non-randomised clinical trial |
|  | Oyen et al., | 2017 | Structural basis for antibody recognition of the NANP repeats in Plasmodium falciparum circumsporozoite protein | Not related to immunogenicity, efficacy, and safety of TB/Malaria vaccine candidate |
|  | Rantala et al., | 2010 | Comparison of real-time PCR and microscopy for malaria parasite detection in Malawian pregnant women | Publication year |
|  | Osman et al., | 2010 | Informed decision-making before changing to RDT: a comparison of microscopy, rapid diagnostic test and molecular techniques for the diagnosis and identification of malaria parasites in Kassala, eastern Sudan | Publication year |
|  | Lawn et al., | 2012 | Characteristics and Early Outcomes of Patients with Xpert MTB/RIF-Negative Pulmonary Tuberculosis Diagnosed During Screening Before Antiretroviral Therapy | Not related to immunogenicity, efficacy, and safety of TB/Malaria vaccine candidate |
|  | Ogwang et al., | 2013 | Safety and immunogenicity of heterologous prime-boost immunisation with Plasmodium falciparum malaria candidate vaccines, ChAd63 ME-TRAP and MVA ME-TRAP, in healthy Gambian and Kenyan adults | Non-randomised study |
|  | O'Hara et al., | 2012 | Clinical assessment of a recombinant simian adenovirus ChAd63: a potent new vaccine vector | Not related to immunogenicity, efficacy, and safety of TB/Malaria vaccine candidate |
|  | Keating et al., | 2005 | Durable human memory T cells quantifiable by cultured enzyme-linked immunospot assays are induced by heterologous prime boost immunization and correlate with protection against malaria | Publication year |
|  | Bejon et al., | 2006 | Immunogenicity of the candidate malaria vaccines FP9 and modified vaccinia virus Ankara encoding the pre-erythrocytic antigen ME-TRAP in 1–6-year-old children in a malaria endemic area | Publication year |
|  | Takuva et al., | 2012 | Antiretroviral therapy initiation during tuberculosis treatment and HIV-RNA and CD4 T-lymphocyte responses | Not related to immunogenicity, efficacy, and safety of TB/Malaria vaccine candidate |
|  | Miller et al., | 2012 | Pharmacokinetic profiles of artesunate following multiple intravenous doses of 2, 4, and 8 mg/kg in healthy volunteers: Phase 1b study | Not related to immunogenicity, efficacy, and safety of TB/Malaria vaccine candidate |
|  | Dunachie et al., | 2006 | A DNA prime-modified vaccinia virus Ankara boost vaccine encoding thrombospondin-related adhesion protein but not circumsporozoite protein partially protects healthy malaria-naive adults against Plasmodium falciparum sporozoite challenge | Publication year |
|  | Bejon et al., | 2006 | Safety profile of the viral vectors of attenuated fowl pox strain FP9 and modified vaccinia virus Ankara recombinant for either of 2 preerythrocytic malaria antigens, ME-TRAP or the circumsporozoite protein, in children and adults in Kenya | Publication year |
|  | Heffron et al., | 2012 | Use of hormonal contraceptives and risk of HIV-1 transmission: A prospective cohort study | Not related to immunogenicity, efficacy, and safety of TB/Malaria vaccine candidate |
|  | Roestenberg et al., | 2009 | Protection against a malaria challenge by sporozoite inoculation | Publication year and non-sub-Saharan African study |
|  | Owusu-ofori et al., | 2012 | Impact of Inconsistent Policies for Transfusion-Transmitted Malaria on Clinical Practice in Ghana | Not related to immunogenicity, efficacy, and safety of TB/Malaria vaccine candidate |
|  | Diacon et al., | 2012 | Phase II Dose-Ranging Trial of the Early Bactericidal Activity of PA-824 | Not related to immunogenicity, efficacy, and safety of TB/Malaria vaccine candidate |
|  | Bejon et al., | 2005 | Calculation of liver-to-blood inocula, parasite growth rates, and preerythrocytic vaccine efficacy, from serial quantitative polymerase chain reaction studies of volunteers challenged with malaria sporozoites | Publication year and not related to immunogenicity, efficacy, and safety of TB/Malaria vaccine candidate |
|  | Bejon et al., | 2006 | A phase 2b randomised trial of the candidate malaria vaccines FP9 ME-TRAP and MVA ME-TRAP among children in Kenya | Publication year |
|  | Imoukhuede, et al., | 2006 | Safety and immunogenicity of the malaria candidate vaccines FP9 CS and MVA CS in adult Gambian men | Publication year |
|  | Moorthy et al., | 2004 | A randomised, double-blind, controlled vaccine efficacy trial of DNA/MVA ME-TRAP against malaria infection in Gambian adults | Publication year |
|  | Olotu et al., | 2011 | Efficacy of RTS, S/AS01E malaria vaccine and exploratory analysis on anti-circumsporozoite antibody titres and protection in children aged 5-17 months in Kenya and Tanzania: a randomised controlled trial | Publication year |
|  | Sachs et al., | 2002 | The economic and social burden of malaria | Publication year and not related to immunogenicity, efficacy, and safety of TB/Malaria vaccine candidate |
|  | Das and Horton | 2002 | Malaria elimination: worthy, challenging, and just possible | Publication year and not related to immunogenicity, efficacy, and safety of TB/Malaria vaccine candidate |
|  | White | 2004 | Antimalarial drug resistance | Not related to immunogenicity, efficacy, and safety of TB/Malaria vaccine candidate and publication year |
|  | Alonso et al., | 2004 | Efficacy of the RTS, S/AS02A vaccine against Plasmodium falciparum infection and disease in young African children: Randomised controlled trial | Publication year |
|  | Baird | 2010 | Eliminating malaria--all of them | Not related to immunogenicity, efficacy, and safety of TB/Malaria vaccine candidate and publication year |
|  | Muehlenbachs et al., | 2012 | Artemether-lumefantrine to treat malaria in pregnancy is associated with reduced placental haemozoin deposition compared to quinine in a randomized controlled trial | Not related to immunogenicity, efficacy, and safety of TB/Malaria vaccine candidate |
|  | Todrysk et al., | 2008 | Correlation of memory T cell responses against TRAP with protection from clinical malaria, and CD4 CD25 high T cells with susceptibility in Kenyans | Publication year |
|  | Khusmith et al., | 1994 | Complete protection against Plasmodium yoelii by adoptive transfer of a CD8+ cytotoxic T-cell clone recognizing sporozoite surface protein 2 | Not related to immunogenicity, efficacy, and safety of TB/Malaria vaccine candidate and publication year |
|  | Reece et al., | 2004 | A CD4(+) T-cell immune response to a conserved epitope in the circumsporozoite protein correlates with protection from natural Plasmodium falciparum infection and disease | Publication year and not related to immunogenicity, efficacy, and safety of TB/Malaria vaccine candidate |
|  | Romero et al., | 1989 | Cloned cytotoxic T cells recognize an epitope in the circumsporozoite protein and protect against malaria | Not related to immunogenicity, efficacy, and safety of TB/Malaria vaccine candidate and publication year |
|  | Schofield et al., | 1987 | Gamma interferon, CD8+ T cells and antibodies required for immunity to malaria sporozoites | Publication year and not related to immunogenicity, efficacy, and safety of TB/Malaria vaccine candidate |
|  | Querec et al., | 2009 | Systems biology approach predicts immunogenicity of the yellow fever vaccine in humans | Not related to immunogenicity, efficacy, and safety of TB/Malaria vaccine candidate and publication year |
|  | Tiano et al., | 2014 | Malaria incidence in children in South-West Burkina Faso: comparison of active and passive case detection methods | Not related to immunogenicity, efficacy, and safety of TB/Malaria vaccine candidate |
|  | Bedell et al., | 2012 | High Prevalence of Tuberculosis and Serious Bloodstream Infections in Ambulatory Individuals Presenting for Antiretroviral Therapy in Malawi | Not related to immunogenicity, efficacy, and safety of TB/Malaria vaccine candidate |
|  | Mash et al., | 2012 | Effectiveness of a group diabetes education programme in underserved communities in South Africa: pragmatic cluster randomized control trial | Not related to immunogenicity, efficacy, and safety of TB/Malaria vaccine candidate |
|  | Pérez et al., | 2001 | An essential role for phoP in Mycobacterium tuberculosis virulence | Not related to immunogenicity, efficacy, and safety of TB/Malaria vaccine candidate and publication year |
|  | Martin et al., | 2006 | The live Mycobacterium tuberculosis phoP mutant strain is more attenuated than BCG and confers protective immunity against tuberculosis in mice and guinea pigs | Publication year, pre-clinical study |
|  | Gonzalo-Asensio et al., | 2008 | The live Mycobacterium tuberculosis phoP mutant strain is more attenuated than BCG and confers protective immunity against tuberculosis in mice and guinea pigs | Publication year and preclinical study |
|  | Turan et al., | 2012 | The Study of HIV and Antenatal Care Integration in Pregnancy in Kenya: Design, Methods, and Baseline Results of a Cluster-Randomized Controlled Trial | Not related to immunogenicity, efficacy, and safety of TB/Malaria vaccine candidate |
|  | Verreck etal., | 2009 | MVA.85A boosting of BCG and an attenuated, phoP deficient M. tuberculosis vaccine both show protective efficacy against tuberculosis in rhesus macaques | Publication year and pre-clinical study |
|  | Spertini et al., | 2015 | Safety of human immunisation with a live-attenuated Mycobacterium tuberculosis vaccine: a randomised, double-blind, controlled phase I trial | Non-sub-Saharan African study |
|  | Polhemus et al., | 2009 | Evaluation of RTS, S/AS02A and RTS, S/AS01B in adults in a high malaria transmission area | Publication year |
|  | Aide et al., | 2011 | Four-year immunogenicity of the RTS, S/AS02(A) malaria vaccine in Mozambican children during a phase IIb trial | Publication year |
|  | Sacarlal et al., | 2009 | Long-term safety and efficacy of the RTS, S/AS02A malaria vaccine in Mozambican children | Publication year |
|  | Matthews et al., | 2012 | HIV-1 infection alters CD4+memory T-cell phenotype at the site of disease in extrapulmonary tuberculosis | Not related to immunogenicity, efficacy, and safety of TB/Malaria vaccine candidate |
|  | Lusingu et al., | 2010 | Safety of the malaria vaccine candidate, RTS, S/AS01E in 5- to 17-month-old Kenyan and Tanzanian Children | Publication year |
|  | Lang et al., | 2012 | Approaching the community about screening children for a multicentre malaria vaccine trial | Not related to immunogenicity, efficacy, and safety of TB/Malaria vaccine candidate |
|  | Bejon et al., | 2008 | Efficacy of RTS, S/AS01E vaccine against malaria in children 5 to 17 months of age | Publication year |
|  | Warimwe et al., | 2013 | Peripheral blood monocyte-to-lymphocyte ratio at study enrollment predicts efficacy of the RTS, S malaria vaccine: analysis of pooled phase II clinical trial data | Not related to immunogenicity, efficacy, and safety of TB/Malaria vaccine candidate |
|  | Ockenhouse et al., | 2006 | Phase I safety and immunogenicity trial of FMP1/AS02A, a Plasmodium falciparum MSP-1 asexual blood stage vaccine | Publication year |
|  | Asante et al., | 2011 | Safety and efficacy of the RTS, S/AS01E candidate malaria vaccine given with expanded-programme-on-immunisation vaccines: 19-month follow-up of a randomised, open-label, phase 2 trial | Publication year |
|  | Agnandji et al., | 2010 | Evaluation of the safety and immunogenicity of the RTS, S/AS01E malaria candidate vaccine when integrated in the expanded program of immunization | Publication year |
|  | Leroux-Roels et al., | 2013 | Improved CD4⁺ T cell responses to Mycobacterium tuberculosis in PPD-negative adults by M72/AS01 as compared to the M72/AS02 and Mtb72F/AS02 tuberculosis candidate vaccine formulations: a randomized trial | Non-sub-Saharan African study |
|  | Owusu-Agyei, et al., | 2009 | Randomized controlled trial of RTS, S/AS02D and RTS, S/AS01E malaria candidate vaccines given according to different schedules in Ghanaian children | Publication year |
|  | Ansong et al., | 2011 | T cell responses to the RTS, S/AS01(E) and RTS, S/AS02(D) malaria candidate vaccines administered according to different schedules to Ghanaian children | Publication year |
|  | Ockenhouse et al., | 2015 | Ad35.CS.01-RTS, S/AS01 Heterologous Prime Boost Vaccine Efficacy against Sporozoite Challenge in Healthy Malaria-Naïve Adults | Non-sub-Saharan African study |
|  | Salkeld et al., | 2022 | Repeat controlled human malaria infection of healthy UK adults with blood-stage Plasmodium falciparum: Safety and parasite growth dynamics | Non-sub-Saharan African study |
|  | Minassian et al., | 2021 | Reduced blood-stage malaria growth and immune correlates in humans following RH5 vaccination | Non-sub-Saharan study |
|  | Willcox et al., | 2021 | Antibodies from malaria-exposed Malians generally interact additively or synergistically with human vaccine-induced RH5 antibodies | Non-randomized study |
|  | Nielsen et al., | 2021 | Protein/AS01B vaccination elicits stronger, more Th2-skewed antigen-specific human T follicular helper cell responses than heterologous viral vectors | Non-sub-Saharan study |
|  | Thacher et al., | 2014 | Safety and immunogenicity of the M72/AS01 candidate tuberculosis vaccine in HIV-infected adults on combination antiretroviral therapy: a phase I/II, randomized trial | Non-sub-Saharan study |
|  | Quintana et al., | 2020 | Measuring Naturally Acquired Phagocytosis-Inducing Antibodies to Plasmodium falciparum Parasites by a Flow Cytometry-Based Assay | Not related to immunogenicity, efficacy, and safety of TB/Malaria vaccine candidate |
|  | Snetselaar et al., | 2017 | Eave tubes for malaria control in Africa: prototyping and evaluation against Anopheles gambiae s.s. and Anopheles arabiensis under semi-field  conditions in western Kenya | Not related to immunogenicity, efficacy, and safety of TB/Malaria vaccine candidate |
|  | Bekker et al., | 2014 | Pharmacokinetics of Isoniazid in Low-Birthweight and Premature Infants | Not related to immunogenicity, efficacy, and safety of TB/Malaria vaccine candidate |
|  | Edwards et al., | 2012 | MCP1 SNPs and Pulmonary Tuberculosis in Cohorts from West Africa, the USA and Argentina: Lack of Association or Epistasis with IL12B  Polymorphisms | Not related to immunogenicity, efficacy, and safety of TB/Malaria vaccine candidate |
|  | Gopal et al., | 2013 | S100A8/A9 Proteins Mediate Neutrophilic Inflammation and Lung Pathology  during Tuberculosis | Not related to immunogenicity, efficacy, and safety of TB/Malaria vaccine candidate |
|  | Mwanga et al., | 2019 | Evaluation of an ultraviolet LED trap for catching Anopheles and Culex mosquitoes in south-eastern Tanzania | Not related to immunogenicity, efficacy, and safety of TB/Malaria vaccine candidate |
|  | Nathavitharana et al., | 2021 | ``If I've got latent TB, I would like to get rid of it'': Derivation of the CARD (Constraints, Actions, Risks, and Desires) Framework informed by South African healthcare worker perspectives on latent tuberculosis treatment | Not related to immunogenicity, efficacy, and safety of TB/Malaria vaccine candidate |
|  | Masalu et al., | 2020 | Creating mosquito-free outdoor spaces using transfluthrin-treated chairs and ribbons | Not related to immunogenicity, efficacy, and safety of TB/Malaria vaccine candidate |
|  | Govella et al., | 2016 | An improved mosquito electrocuting trap that safely reproduces epidemiologically relevant metrics of mosquito human-feeding behaviours as determined by human landing catch | Not related to immunogenicity, efficacy, and safety of TB/Malaria vaccine candidate |
|  | Ubben and Poll | 2013 | MMV in partnership: the Eurartesim (R) experience | Not related to immunogenicity, efficacy, and safety of TB/Malaria vaccine candidate |
|  | Awine et al., | 2020 | Accounting for regional transmission variability and the impact of malaria control interventions in Ghana: a population level mathematical modelling approach | Not related to immunogenicity, efficacy, and safety of TB/Malaria vaccine candidate |
|  | Fongnikin et al., | 2022 | Pirikool (R) 300 CS, a new long-lasting capsule suspension formulation of the organophosphate insecticide pirimiphos-methyl for indoor residual spraying against pyrethroid-resistant malaria vectors | Not related to immunogenicity, efficacy, and safety of TB/Malaria vaccine candidate |
|  | Rangaka et al., | 2012 | Interferon release does not add discriminatory value to smear-negative HIV-tuberculosis algorithms | Not related to immunogenicity, efficacy, and safety of TB/Malaria vaccine candidate |
|  | Bernard et al., | 2016 | Mycobacterium ulcerans Mouse Model Refinement for Pre-Clinical Profiling of Vaccine Candidates | Not related to immunogenicity, efficacy, and safety of TB/Malaria vaccine candidate |
|  | Brazeau et al., | 2019 | Pooled Deep Sequencing of Drug Resistance Loci from Plasmodium falciparum Parasites across Ethiopia | Not related to immunogenicity, efficacy, and safety of TB/Malaria vaccine candidate |
|  | Kauta et al., | 2019 | Spodoptera frugiperda Smith (Lepidoptera: Noctuidae) in Cameroon: Case study on its distribution, damage, pesticide use, genetic differentiation, and host plants | Not related to immunogenicity, efficacy, and safety of TB/Malaria vaccine candidate |
|  | Musa et al. | 2015 | Incidence of tuberculosis and immunological profile of TB/HIVco-infected patients in Nigeria | Not related to immunogenicity, efficacy, and safety of TB/Malaria vaccine candidate |
|  | Waisberg et al., | 2012 | Testing in Mice the Hypothesis That Melanin Is Protective in Malaria Infections | Not related to immunogenicity, efficacy, and safety of TB/Malaria vaccine candidate |
|  | de Sousa et al., | 2012 | Pharmacovigilance of Malaria Intermittent Preventive Treatment in Infants Coupled with Routine Immunizations in 6 African Countries | Non- vaccination study |
|  | Nguetse et al., | 2017 | Molecular markers of anti-malarial drug resistance in Central, West and East African children with severe malaria | Not related to immunogenicity, efficacy, and safety of TB/Malaria vaccine candidate |
|  | Matrevi et al., | 2022 | Novel pfk13 polymorphisms in Plasmodium falciparum population in Ghana | Not related to immunogenicity, efficacy, and safety of TB/Malaria vaccine candidate |
|  | Fowkes et al., | 2016 | Immunity to malaria in an era of declining malaria transmission | Not related to immunogenicity, efficacy, and safety of TB/Malaria vaccine candidate |
|  | Waliis | 2016 | Mathematical Models of Tuberculosis Reactivation and Relapse | Not related to immunogenicity, efficacy, and safety of TB/Malaria vaccine candidate |
|  | Das et al., | 2021 | A novel nano-anti-malarial induces redox damage and elicits cytokine  response to the parasite | Not related to immunogenicity, efficacy, and safety of TB/Malaria vaccine candidate |
|  | Ghilardi et al., | 2020 | How useful are malaria risk maps at the country level? Perceptions of decision-makers in Kenya, Malawi, and the Democratic Republic of Congo | Not related to immunogenicity, efficacy, and safety of TB/Malaria vaccine candidate |
|  | Arora and shrama | 2019 | Global scenario of counterfeit antimalarials: A potential threat | Not related to immunogenicity, efficacy, and safety of TB/Malaria vaccine candidate |
|  | Ssengooba et al., | 2015 | High Genotypic Discordance of Concurrent Mycobacterium tuberculosis Isolates from Sputum and Blood of HIV-Infected Individuals | Not related to immunogenicity, efficacy, and safety of TB/Malaria vaccine candidate |
|  | Moukoko et al., | 2019 | K-13 propeller gene polymorphisms isolated between 2014 and 2017 from Cameroonian Plasmodium falciparum malaria patients | Non-vaccination sturdy |
|  | Mbuba et al., | 2023 | Multi-country evaluation of the durability of pyrethroid plus piperonyl-butoxide insecticide-treated nets: study protocol | Not related to immunogenicity, efficacy, and safety of TB/Malaria vaccine candidate |
|  | Chegou etal., | 2012 | Potential of Host Markers Produced by Infection Phase-Dependent Antigen-Stimulated Cells for the Diagnosis of Tuberculosis in a Highly Endemic Area | Not related to immunogenicity, efficacy, and safety of TB/Malaria vaccine candidate |
|  | de Kock et al., | 2014 | Pharmacokinetics of para-Aminosalicylic Acid in HIV-Uninfected and HIV-Coinfected Tuberculosis Patients Receiving Antiretroviral Therapy, Managed for Multidrug-Resistant and Extensively Drug-Resistant Tuberculosis | Not related to immunogenicity, efficacy, and safety of TB/Malaria vaccine candidate |
|  | Kalloli et al., | 2021 | Aggregation state of Mycobacterium tuberculosis impacts host immunity and augments pulmonary disease pathology | Not related to immunogenicity, efficacy, and safety of TB/Malaria vaccine candidate |
|  | Maziarz et al., | 2017 | Age and geographic patterns of Plasmodium falciparum malaria infection in a representative sample of children living in Burkitt lymphoma-endemic areas of northern Uganda | Not related to immunogenicity, efficacy, and safety of TB/Malaria vaccine candidate |
|  | Moyo et al., | 2012 | Tuberculosis case finding for vaccine trials in young children in high-incidence settings: a randomised trial | Not related to immunogenicity, efficacy, and safety of TB/Malaria vaccine candidate |
|  | Talundzic et al., | 2012 | Sequence Polymorphism, Segmental Recombination and Toggling Amino Acid Residues within the DBL3X Domain of the VAR2CSA Placental Malaria Antigen | Not related to immunogenicity, efficacy, and safety of TB/Malaria vaccine candidate |
|  | Nayebare et al., | 2020 | Associations between Malaria-Preventive Regimens and Plasmodium falciparum Drug Resistance-Mediating Polymorphisms in Ugandan Pregnant  Women | Not related to immunogenicity, efficacy, and safety of TB/Malaria vaccine candidate |
|  | Bonizzoni et al., | 2015 | RNA-seq analyses of changes in the Anopheles gambiae transcriptome associated with resistance to pyrethroids in Kenya: identification of candidate-resistance genes and candidate-resistance SNPs | Not related to immunogenicity, efficacy, and safety of TB/Malaria vaccine candidate |
|  | Palomo et al., | 2013 | Type I interferons contribute to experimental cerebral malaria development in response to sporozoite or blood-stage Plasmodium berghei ANKA | Not related to immunogenicity, efficacy, and safety of TB/Malaria vaccine candidate |
|  | Erlank et al., | 2022 | Standard Membrane Feeding Assay for the Detection of Plasmodiumfalciparum Infection in Anopheles Mosquito Vectors | Not related to immunogenicity, efficacy, and safety of TB/Malaria vaccine candidate |
|  | Edessa et al., | 2015 | A Description of Mortality Associated with IPT plus ART Compared to ART Alone among HIV-Infected Individuals in Addis Ababa, Ethiopia: A Cohort Study | Not related to immunogenicity, efficacy, and safety of TB/Malaria vaccine candidate |
|  | Bentley et al., | 2012 | The Genome of Mycobacterium Africanum West African 2 Reveals a Lineage-Specific Locus and Genome Erosion Common to the M. tuberculosis Complex | Not related to immunogenicity, efficacy, and safety of TB/Malaria vaccine candidate |
|  | Adedokun et al., | 2020 | Individual and contextual correlates of mosquito net use among women in Nigeria | Not related to immunogenicity, efficacy, and safety of TB/Malaria vaccine candidate |
|  | Ferguson et al., | 2020 | Cost-effectiveness of one month of daily isoniazid and rifapentine versus three months of weekly isoniazid and rifapentine for prevention of tuberculosis among people receiving antiretroviral therapy in Uganda | Not related to immunogenicity, efficacy, and safety of TB/Malaria vaccine candidate |
|  | Henostroza et al., | 2016 | High prevalence of tuberculosis in newly enrolled HIV patients in Zambia: need for enhanced screening approach | Not related to immunogenicity, efficacy, and safety of TB/Malaria vaccine candidate |
|  | Jensenius et al., | 2016 | Multidrug-resistant tuberculosis in Norway: a nationwide study, 1995-2014 | Not related to immunogenicity, efficacy, and safety of TB/Malaria vaccine candidate |
|  | van Lettow et al., | 2012 | Six-Month Mortality among HIV-Infected Adults Presenting for Antiretroviral Therapy with Unexplained Weight Loss, Chronic Fever or Chronic Diarrhea in Malawi | Not related to immunogenicity, efficacy, and safety of TB/Malaria vaccine candidate |
|  | Chigutsa et al., | 2012 | Population Pharmacokinetics and Pharmacodynamics of Ofloxacin in South African Patients with Multidrug-Resistant Tuberculosis | Not related to immunogenicity, efficacy, and safety of TB/Malaria vaccine candidate |
|  | Griesel et al., | 2019 | Abdominal Ultrasound for the Diagnosis of Tuberculosis Among Humans Immunodeficiency Virus-Positive Inpatients with World Health Organization Danger Signs | Not related to immunogenicity, efficacy, and safety of TB/Malaria vaccine candidate |
|  | Londono-Renteria et al., | 2015 | An. gambiae gSG6-P1 evaluation as a proxy for human-vector contact in the Americas: a pilot study | Not related to immunogenicity, efficacy, and safety of TB/Malaria vaccine candidate |
|  | Ajibola et al., | 2021 | In silico characterisation of putative Plasmodium falciparum vaccine candidates in African malaria populations | Not related to immunogenicity, efficacy, and safety of TB/Malaria vaccine candidate |
|  | Leisching et al., | 2017 | RNAseq reveals hypervirulence-specific host responses to M-tuberculosis infection | Not related to immunogenicity, efficacy, and safety of TB/Malaria vaccine candidate |
|  | Kasirye et al., | 2017 | Effect of antiretroviral therapy on malaria incidence in HIV-infected Ugandan adults | Not related to immunogenicity, efficacy, and safety of TB/Malaria vaccine candidate |
|  | Korenromp et al., | 2016 | Malaria intervention scale-up in Africa: effectiveness predictions for health programme planning tools, based on dynamic transmission modelling | Not related to immunogenicity, efficacy, and safety of TB/Malaria vaccine candidate |
|  | Dye | 2013 | Making wider use of the world's most widely used vaccine: Bacille Calmette-Guerin revaccination reconsidered | Not related to immunogenicity, efficacy, and safety of TB/Malaria vaccine candidate |
|  | Mitchell et al., | 2012 | Prospective Monitoring Reveals Dynamic Levels of T Cell Immunity to Mycobacterium Tuberculosis in HIV Infected Individuals | Not related to immunogenicity, efficacy, and safety of TB/Malaria vaccine candidate |
|  | Golumbeanu et al., | 2022 | Leveraging mathematical models of disease dynamics and machine learning to improve development of novel malaria interventions | Not related to immunogenicity, efficacy, and safety of TB/Malaria vaccine candidate |
|  | Maiga et al., | 2014 | Glucose-6-phosphate dehydrogenase polymorphisms and susceptibility to mild malaria in Dogon and Fulani, Mali | Not related to immunogenicity, efficacy, and safety of TB/Malaria vaccine candidate |
|  | Campbell et al., | 2021 | BCG Vaccine Protection Against Mycobacterium Tuberculosis Infection by  Level of Exposure in The Gambia | Not related to immunogenicity, efficacy, and safety of TB/Malaria vaccine candidate |
|  | Braack et al., | 2015 | Biting behaviour of African malaria vectors: 1. where do the main vector species bite on the human body? | Not related to immunogenicity, efficacy, and safety of TB/Malaria vaccine candidate |
|  | Mzinza et al., | 2015 | Kinetics of Mycobacterium tuberculosis-specific IFN-gamma responses and sputum bacillary clearance in HIV-infected adults during treatment of pulmonary tuberculosis | Not related to immunogenicity, efficacy, and safety of TB/Malaria vaccine candidate |
|  | Tanabe et al., | 2012 | Geographic differentiation of polymorphism in the Plasmodium falciparum malaria vaccine candidate gene SERA5 | Not related to immunogenicity, efficacy, and safety of TB/Malaria vaccine candidate |
|  | Aberese-Ako et al., | 2021 | An ethnographic study of how health system, socio-cultural and individual factors influence uptake of intermittent preventive treatment of malaria in pregnancy with sulfadoxine-pyrimethamine in a Ghanaian context | Not related to immunogenicity, efficacy, and safety of TB/Malaria vaccine candidate |
|  | Krogstad et al., | 2015 | Molecular incidence and clearance of Plasmodium falciparum infection | Not related to immunogenicity, efficacy, and safety of TB/Malaria vaccine candidate |
|  | Ba et al., | 2016 | Widespread distribution of Plasmodium vivax malaria in Mauritania on the interface of the Maghreb and West Africa | Not related to immunogenicity, efficacy, and safety of TB/Malaria vaccine candidate |
|  | Chen et al., | 2021 | Radiological and functional evidence of the bronchial spread of tuberculosis: an observational analysis | Not related to immunogenicity, efficacy, and safety of TB/Malaria vaccine candidate |
|  | Swanson et al., | 2023 | Antigen-specific B cells direct T follicular-like helper cells into lymphoid follicles to mediate Mycobacterium tuberculosis control | Not related to immunogenicity, efficacy, and safety of TB/Malaria vaccine candidate |
|  | Duffy et al., | 2019 | Multinomial modelling of TB/HIV co-infection yields a robust predictive signature and generates hypotheses about the HIV plus TB plus disease state | Not related to immunogenicity, efficacy, and safety of TB/Malaria vaccine candidate |
|  | Maiga et al., | 2012 | No Evidence of Delayed Parasite Clearance after Oral Artesunate Treatment of Uncomplicated Falciparum Malaria in Mali | Not related to immunogenicity, efficacy, and safety of TB/Malaria vaccine candidate |
|  | du Plessis et al., | 2016 | The Functional Response of B Cells to Antigenic Stimulation: A Preliminary Report of Latent Tuberculosis | Not related to immunogenicity, efficacy, and safety of TB/Malaria vaccine candidate |
|  | Matowo et al., | 2022 | Expression of pyrethroid metabolizing P450 enzymes characterizes highly resistant Anopheles vector species targeted by successful deployment of PBO-treated bednets in Tanzania | Not related to immunogenicity, efficacy, and safety of TB/Malaria vaccine candidate |
|  | Richie et al., | 2015 | Progress with Plasmodium falciparum sporozoite (PfSPZ)-based malaria vaccines | Not related to immunogenicity, efficacy, and safety of TB/Malaria vaccine candidate |
|  | Diendere et al., | 2015 | Clinical outcomes and mortality associated factors in patients infected with HIV receiving a presumptive anti-tuberculosis treatment in a tertiary level hospital in Burkina Faso | Not related to immunogenicity, efficacy, and safety of TB/Malaria vaccine candidate |
|  | Guindo et al., | 2012 | Promoting Good Clinical Laboratory Practices and Laboratory Accreditation to Support Clinical Trials in Sub-Saharan Africa | Not related to immunogenicity, efficacy, and safety of TB/Malaria vaccine candidate |
|  | Asidi et al., | 2012 | Loss of Household Protection from Use of Insecticide-Treated Nets against Pyrethroid-Resistant Mosquitoes, Benin | Not related to immunogenicity, efficacy, and safety of TB/Malaria vaccine candidate |
|  | Zumla et al., | 2012 | Drug-Resistant Tuberculosis-Current Dilemmas, Unanswered Questions, Challenges, and Priority Needs | Not related to immunogenicity, efficacy, and safety of TB/Malaria vaccine candidate |
|  | Cox | 2012 | Wind-Driven Roof Turbines: A Novel Way to Improve Ventilation for TB Infection Control in Health Facilities | Not related to immunogenicity, efficacy, and safety of TB/Malaria vaccine candidate |
|  | Kirenga et al., | 2013 | Tuberculin skin test conversion among HIV patients on antiretroviral therapy in Uganda | Not related to immunogenicity, efficacy, and safety of TB/Malaria vaccine candidate |
|  | MacCord | 2017 | A Malaria Ecology Index Predicted Spatial and Temporal Variation of Malaria Burden and Efficacy of Antimalarial Interventions Based on African Serological Data | Not related to immunogenicity, efficacy, and safety of TB/Malaria vaccine candidate |
|  | Augusto et al., | 2020 | First trimester use of artemisinin-based combination therapy and the risk of low birth weight and small for gestational age | Not related to immunogenicity, efficacy, and safety of TB/Malaria vaccine candidate |
|  | Nolan et al., | 2013 | Elevated IP-10 and IL-6 from bronchoalveolar lavage cells are biomarkers of non-cavitary tuberculosis | Not related to immunogenicity, efficacy, and safety of TB/Malaria vaccine candidate |
|  | McCarthy et al., | 2015 | High incidence of latent tuberculous infection among South African health workers: an urgent call for action | Not related to immunogenicity, efficacy, and safety of TB/Malaria vaccine candidate |
|  | Sridhara et al., | 2022 | Differential detection of IgM and IgG antibodies to chimeric antigens in bovine tuberculosis | Not related to immunogenicity, efficacy, and safety of TB/Malaria vaccine candidate |
|  | Cavanaugh et al., | 2016 | Comparative Yield of Different Diagnostic Tests for Tuberculosis among People Living with HIV in Western Kenya | Not related to immunogenicity, efficacy, and safety of TB/Malaria vaccine candidate |
|  | Bradley et al., | 2017 | Insecticide-treated nets provide protection against malaria to children in an area of insecticide resistance in Southern Benin | Not related to immunogenicity, efficacy, and safety of TB/Malaria vaccine candidate |
|  | Zhou et al., | 2021 | An Adaptive Intervention Trial Design for Finding the Optimal Integrated Strategies for Malaria Control and Elimination in Africa: A Model Simulation Study | Not related to immunogenicity, efficacy, and safety of TB/Malaria vaccine candidate |
|  | Akanbi et al., | 2013 | Tuberculosis After One Year of Combination Antiretroviral Therapy in Nigeria: A Retrospective Cohort Study | Not related to immunogenicity, efficacy, and safety of TB/Malaria vaccine candidate |
|  | Agarwal et al., | 2020 | Foam Cells ControlMycobacterium tuberculosisInfection | Not related to immunogenicity, efficacy, and safety of TB/Malaria vaccine candidate |
|  | Omondi et al., | 2019 | Gametocyte clearance in children, from western Kenya, with uncomplicated  Plasmodium falciparum malaria after artemether-lumefantrine or dihydroartemisinin-piperaquine treatment | Not related to immunogenicity, efficacy, and safety of TB/Malaria vaccine candidate |
|  | Liu et al., | 2014 | African origin of the malaria parasite Plasmodium vivax | Not related to immunogenicity, efficacy, and safety of TB/Malaria vaccine candidate |
|  | Stein et al., | 2021 | Methylome-wide Analysis Reveals Epigenetic Marks Associated with Resistance to Tuberculosis in Human Immunodeficiency Virus-Infected Individuals from East Africa | Not related to immunogenicity, efficacy, and safety of TB/Malaria vaccine candidate |
|  | Cai et al., | 2021 | Host immunity increases Mycobacterium tuberculosis reliance on cytochrome bd oxidase | Not related to immunogenicity, efficacy, and safety of TB/Malaria vaccine candidate |
|  | Ezenyi et al., | 2016 | Approaches, Challenges and Prospects of Antimalarial Drug Discovery from Plant Sources | Not related to immunogenicity, efficacy, and safety of TB/Malaria vaccine candidate |
|  | Drain et al., | 2016 | Rapid Urine LAM Testing Improves Diagnosis of Expectorated Smear-Negative Pulmonary Tuberculosis in an HIV-endemic Region | Not related to immunogenicity, efficacy, and safety of TB/Malaria vaccine candidate |
|  | Bayili et al., | 2019 | Experimental hut evaluation of DawaPlus 3.0 LN and DawaPlus 4.0 LN treated with deltamethrin and PBO against free-flying populations of Anopheles gambiae s.l. in Vallee du Kou, Burkina Faso | Not related to immunogenicity, efficacy, and safety of TB/Malaria vaccine candidate |
|  | Heslop et al., | 2016 | Changes in Host Cytokine Patterns of TB Patients with Different Bacterial Loads Detected Using S-16 rRNA Analysis | Not related to immunogenicity, efficacy, and safety of TB/Malaria vaccine candidate |
|  | Oelofse et al., | 2021 | Pretomanid with bedaquiline and linezolid for drug-resistant TB: a comparison of prospective cohorts | Not related to immunogenicity, efficacy, and safety of TB/Malaria vaccine candidate |
|  | Yaro et al., | 2021 | Risk factors associated with house entry of malaria vectors in an area of Burkina Faso with high, persistent malaria transmission and high insecticide resistance | Not related to immunogenicity, efficacy, and safety of TB/Malaria vaccine candidate |
|  | Osborne et al., | 2021 | Characterizing the genomic variation and population dynamics of Plasmodium falciparum malaria parasites in and around Lake Victoria, Kenya | Not related to immunogenicity, efficacy, and safety of TB/Malaria vaccine candidate |
|  | Kerkhoff et al., | 2014 | Prognostic Value of a Quantitative Analysis of Lipoarabinomannan in Urine from Patients with HIV-Associated Tuberculosis | Not related to immunogenicity, efficacy, and safety of TB/Malaria vaccine candidate |
|  | Maskus et al., | 2016 | Characterization of a novel inhibitory human monoclonal antibody directed against Plasmodium falciparum Apical Membrane Antigen 1 | Not related to immunogenicity, efficacy, and safety of TB/Malaria vaccine candidate |
|  | Warimwe et al., | 2013 | The Ratio of Monocytes to Lymphocytes in Peripheral Blood Correlates with Increased Susceptibility to Clinical Malaria in Kenyan Children | Not related to immunogenicity, efficacy, and safety of TB/Malaria vaccine candidate |
|  | Steketee et al., | 2020 | World Malaria Day 2021: Commemorating 15 Years of Contribution by the  United States President's Malaria Initiative | Not related to immunogenicity, efficacy, and safety of TB/Malaria vaccine candidate |
|  | Leroy et al., | 2019 | African isolates show a high proportion of multiple copies of the Plasmodium falciparum plasmepsin-2 gene, a piperaquine resistance marker | Not related to immunogenicity, efficacy, and safety of TB/Malaria vaccine candidate |
|  | Smithuis et al., | 2013 | The effect of insecticide-treated bed nets on the incidence and prevalence of malaria in children in an area of unstable seasonal transmission in western Myanmar | Not related to immunogenicity, efficacy, and safety of TB/Malaria vaccine candidate |
|  | Nyarko et al., | 2020 | Investigating a Plasmodium falciparum erythrocyte invasion phenotype switch at the whole transcriptome level | Not related to immunogenicity, efficacy, and safety of TB/Malaria vaccine candidate |
|  | Van Ginderdeuren et al., | 2021 | High conversion of tuberculin skin tests during the first year of antiretroviral treatment among South African adults in primary care | Not related to immunogenicity, efficacy, and safety of TB/Malaria vaccine candidate |
|  | Hoshide et al., | 2014 | Geographical Differences Associated with Single-Nucleotide Polymorphisms  (SNPs) in Nine Gene Targets among Resistant Clinical Isolates of Mycobacterium tuberculosis | Not related to immunogenicity, efficacy, and safety of TB/Malaria vaccine candidate |
|  | Hoffman et al | 2014 | Cotrimoxazole Prophylaxis and Tuberculosis Risk among People Living with HIV | Not related to immunogenicity, efficacy, and safety of TB/Malaria vaccine candidate |
|  | Traore et al., | 2021 | Laboratory and field evaluation of MAIA(R), an ointment containing N, N-diethyl-3-methylbenzamide (DEET) against mosquitoes in Burkina Faso | Not related to immunogenicity, efficacy, and safety of TB/Malaria vaccine candidate |
|  | Hughes et al., | 2020 | Anopheles gambiae populations from Burkina Faso show minimal delayed mortality after exposure to insecticide-treated nets | Not related to immunogenicity, efficacy, and safety of TB/Malaria vaccine candidate |
|  | Goussard et al., | 2015 | Decompression of Enlarged Mediastinal Lymph Nodes Due to Mycobacterium  Tuberculosis Causing Severe Airway Obstruction in Children | Not related to immunogenicity, efficacy, and safety of TB/Malaria vaccine candidate |
|  | Nyberg et al., | 2020 | Population Pharmacokinetics and Dosing of Ethionamide in Children with Tuberculosis | Not related to immunogenicity, efficacy, and safety of TB/Malaria vaccine candidate |
|  | Beogo et al., | 2016 | Malaria related care-seeking-behaviour and expenditures in urban settings: A household survey in Ouagadougou, Burkina Faso | Not related to immunogenicity, efficacy, and safety of TB/Malaria vaccine candidate |
|  | Kafy et al., | 2017 | Impact of insecticide resistance in Anopheles arabiensis on malaria incidence and prevalence in Sudan and the costs of mitigation | Not related to immunogenicity, efficacy, and safety of TB/Malaria vaccine candidate |
|  | Manirakiza et al., | 2021 | Cotrimoxazole versus sulfadoxine-pyrimethamine for intermittent preventive treatment of malaria in HIV-infected pregnant women in Bangui, Central African Republic: A pragmatic randomised controlled trial | Not related to immunogenicity, efficacy, and safety of TB/Malaria vaccine candidate |
|  | Gcebe et al., | 2017 | Mycobacterium malmesburyense sp nov., a non-tuberculous species of the genus Mycobacterium revealed by multiple gene sequence characterization | Not related to immunogenicity, efficacy, and safety of TB/Malaria vaccine candidate |
|  | Baumann et al., | 2015 | A Subgroup of Latently Mycobacterium tuberculosis Infected Individuals Is Characterized by Consistently Elevated IgA Responses to Several Mycobacterial Antigens | Not related to immunogenicity, efficacy, and safety of TB/Malaria vaccine candidate |
|  | Van Rie et al., | 2013 | Diagnostic accuracy and effectiveness of the Xpert MTB/RIF assay for the diagnosis of HIV-associated lymph node tuberculosis | Not related to immunogenicity, efficacy, and safety of TB/Malaria vaccine candidate |
|  | Cock et al., | 2017 | Molecular methods to detect Spodoptera frugiperda in Ghana, and implications for monitoring the spread of invasive species in developing countries | Not related to immunogenicity, efficacy, and safety of TB/Malaria vaccine candidate |
|  | Omer et al., | 2021 | Impact of placental malaria on maternal, placental, and fetal cord responses and its role in pregnancy outcomes in women from Blue Nile State, Sudan | Not related to immunogenicity, efficacy, and safety of TB/Malaria vaccine candidate |
|  | Chatio | 2019 | Community acceptability of Seasonal Malaria Chemoprevention of morbidity  and mortality in young children: A qualitative study in the Upper West Region of Ghana | Not related to immunogenicity, efficacy, and safety of TB/Malaria vaccine candidate |
|  | Taylor et al., | 2013 | Plasmodium falciparum sulfadoxine resistance is geographically and genetically clustered within the DR Congo | Not related to immunogenicity, efficacy, and safety of TB/Malaria vaccine candidate |
|  | Debalke et al., | 2020 | Stability of the effect of silencing fibronectin type III domain-protein 1 (FN3D1) gene on Anopheles arabiensis reared under different breeding site conditions | Not related to immunogenicity, efficacy, and safety of TB/Malaria vaccine candidate |
|  | Andriessen etal., | 2015 | Electrostatic coating enhances bioavailability of insecticides and breaks pyrethroid resistance in mosquitoes | Not related to immunogenicity, efficacy, and safety of TB/Malaria vaccine candidate |
|  | Quao et al., |  | Leprosy | Not related to immunogenicity, efficacy, and safety of TB/Malaria vaccine candidate |
|  | Dhingra et al., | 2019 | Global Spread of Mutant PfCRT and Its Pleiotropic Impact on Plasmodium falciparum Multidrug Resistance and Fitness | Not related to immunogenicity, efficacy, and safety of TB/Malaria vaccine candidate |
|  | Lun et al., | 2013 | Pharmacokinetic and In Vivo Efficacy Studies of the Mycobactin Biosynthesis Inhibitor Salicyl-AMS in Mice | Not related to immunogenicity, efficacy, and safety of TB/Malaria vaccine candidate |
|  | Hast et al., | 2021 | The Impact of Three Years of Targeted Indoor Residual Spraying with Pirimiphos-Methyl on Household Vector Abundance in a High Malaria Transmission Area of Northern Zambia | Not related to immunogenicity, efficacy, and safety of TB/Malaria vaccine candidate |
|  | Davenport | 2016 | Reduced Parasite Burden in Children with Falciparum Malaria and Bacteremia Coinfections: Role of Mediators of Inflammation | Not related to immunogenicity, efficacy, and safety of TB/Malaria vaccine candidate |
|  | Smith et al., | 2021 | Optimized interferon-gamma release assays for detection of Mycobacterium bovis infection in African buffaloes (Syncerus caffer) | Not related to immunogenicity, efficacy, and safety of TB/Malaria vaccine candidate |
|  | Cox et al., | 2017 | World Health Organization recommendations for multidrug-resistant tuberculosis: should different standards be applied? | Not related to immunogenicity, efficacy, and safety of TB/Malaria vaccine candidate |
|  | Atchade et al., | 2013 | Is a Plasmodium lactate dehydrogenase (pLDH) enzyme-linked immunosorbent  (ELISA)-based assay a valid tool for detecting risky malaria blood donations in Africa? | Not related to immunogenicity, efficacy, and safety of TB/Malaria vaccine candidate |
|  | Mpogoro et al., | 2014 | Uptake of intermittent preventive treatment with sulphadoxine-pyrimethamine for malaria during pregnancy and pregnancy outcomes: a cross-sectional study in Geita district, North-Western Tanzania | Not related to immunogenicity, efficacy, and safety of TB/Malaria vaccine candidate |
|  | Hemingway et al., | 2017 | Childhood tuberculosis is associated with decreased abundance of T cell gene transcripts and impaired T cell function | Not related to immunogenicity, efficacy, and safety of TB/Malaria vaccine candidate |
|  | Amegashie, et al., | 2020 | Population genetic analysis of the Plasmodium falciparum circumsporozoite protein in two distinct ecological regions in Ghana | Not related to immunogenicity, efficacy, and safety of TB/Malaria vaccine candidate |
|  | Arinaitwe et al., | 2013 | Intermittent Preventive Therapy with Sulfadoxine-Pyrimethamine for Malaria in Pregnancy: A Cross-Sectional Study from Tororo, Uganda | Not related to immunogenicity, efficacy, and safety of TB/Malaria vaccine candidate |
|  | Qi et al., | 2015 | Evaluation of the Efficiency of the Sample Inactivation Reagent in the Abbott RealTime MTB Assay for Inactivation of Mycobacterium tuberculosis | Not related to immunogenicity, efficacy, and safety of TB/Malaria vaccine candidate |
|  | Oxborough et al., | 2019 | Susceptibility testing of Anopheles malaria vectors with the neonicotinoid insecticide clothianidin; results from 16 African countries, in preparation for indoor residual spraying with new insecticide formulations | Not related to immunogenicity, efficacy, and safety of TB/Malaria vaccine candidate |
|  | Pho et al., | 2012 | The Cost-Effectiveness of Tuberculosis Preventive Therapy for HIV-Infected Individuals in Southern India: A Trial-Based Analysis | Not related to immunogenicity, efficacy, and safety of TB/Malaria vaccine candidate |
|  | Kiware et al., | 2012 | Simplified Models of Vector Control Impact upon Malaria Transmission by Zoophagic Mosquitoes | Not related to immunogenicity, efficacy, and safety of TB/Malaria vaccine candidate |
|  | Ochomo et al., | 2017 | Insecticide-Treated Nets and Protection against Insecticide-Resistant Malaria Vectors in Western Kenya | Not related to immunogenicity, efficacy, and safety of TB/Malaria vaccine candidate |
|  | Limwagu et al., | 2019 | Using a miniaturized double-net trap (DN-Mini) to assess relationships between indoor-outdoor biting preferences and physiological ages of two malaria vectors, Anopheles arabiensis and Anopheles funestus | Not related to immunogenicity, efficacy, and safety of TB/Malaria vaccine candidate |
|  | Odhiambo et al., | 2019 | Correlation Between Malaria-Specific Antibody Profiles and Responses to Artemisinin Combination Therapy for Treatment of Uncomplicated Malaria in Western Kenya | Not related to immunogenicity, efficacy, and safety of TB/Malaria vaccine candidate |
|  | Koele et al., | 2022 | Optimized Loading Dose Strategies for Bedaquiline When Restarting Interrupted Drug-Resistant Tuberculosis Treatment | Not related to immunogenicity, efficacy, and safety of TB/Malaria vaccine candidate |
|  | Baguma et al., | 2017 | Application of a whole blood mycobacterial growth inhibition assay to study immunity against Mycobacterium tuberculosis in a high tuberculosis burden population | Not related to immunogenicity, efficacy, and safety of TB/Malaria vaccine candidate |
|  | Longwe et al., | 2017 | The Effect of Daily Co-Trimoxazole Prophylaxis on Natural Development of Antibody-Mediated Immunity against P. falciparum Malaria Infection in HIV-Exposed Uninfected Malawian Children | Not related to immunogenicity, efficacy, and safety of TB/Malaria vaccine candidate |
|  | Zahouli et al., | 2023 | Small-scale field evaluation of PermaNet((R)) Dual (a long-lasting net coated with a mixture of chlorfenapyr and deltamethrin) against pyrethroid-resistant Anopheles gambiae mosquitoes from Tiassale, Cote d'Ivoire | Not related to immunogenicity, efficacy, and safety of TB/Malaria vaccine candidate |
|  | Huerga et al., | 2022 | Safety and Effectiveness Outcomes From a 14-Country Cohort of Patients With Multi-Drug-Resistant Tuberculosis Treated Concomitantly With Bedaquiline, Delamanid, and Other Second-Line Drugs | Not related to immunogenicity, efficacy, and safety of TB/Malaria vaccine candidate |
|  | Scriba et al., | 2017 | Human Immunology of Tuberculosis | Not related to immunogenicity, efficacy, and safety of TB/Malaria vaccine candidate |
|  | Kitau et al., | 2014 | Laboratory and experimental hut evaluation of a long-lasting insecticide treated blanket for protection against mosquitoes | Not related to immunogenicity, efficacy, and safety of TB/Malaria vaccine candidate |
|  | Kleinnijenhuis et al., | 2014 | Long-Lasting Effects of BCG Vaccination on Both Heterologous Th1/Th17 Responses and Innate Trained Immunity | Non-randomised study |
|  | Guler et al., | 2019 | Batf2 differentially regulates tissue immunopathology in Type 1 and Type 2 diseases | Not related to immunogenicity, efficacy, and safety of TB/Malaria vaccine candidate |
|  | Briset et al., | 2022 | Non-traumatic coma in young children in Benin are viral and bacterial infections gaining ground on cerebral malaria? | Not related to immunogenicity, efficacy, and safety of TB/Malaria vaccine candidate |
|  | Akano et al., | 2019 | Clinical illness and outcomes in Nigerian children with persistent early appearing anaemia following initiation of artemisinin-based combination treatments of uncomplicated falciparum malaria | Not related to immunogenicity, efficacy, and safety of TB/Malaria vaccine candidate |
|  | Knoll et al., | 2022 | In Silico Drug Discovery Strategies Identified ADMET Properties of Decoquinate RMB041 and Its Potential Drug Targets against Mycobacterium tuberculosis | Not related to immunogenicity, efficacy, and safety of TB/Malaria vaccine candidate |
|  | Kimuda et al., | 2017 | Humoral Responses to Rv1733c, Rv0081, Rv1735c, and Rv1737c DosR Regulon-Encoded Proteins of Mycobacterium tuberculosis in Individuals with Latent Tuberculosis Infection | Not related to immunogenicity, efficacy, and safety of TB/Malaria vaccine candidate |
|  | Chirehwa et al., | 2023 | Optimizing Moxifloxacin Dose in MDR-TB Participants with or without Efavirenz Coadministration Using Population Pharmacokinetic Modeling | Not related to immunogenicity, efficacy, and safety of TB/Malaria vaccine candidate |
|  | Omar et al., | 2021 | Host Blood Gene Signatures Can Detect the Progression to Severe and Cerebral Malaria | Not related to immunogenicity, efficacy, and safety of TB/Malaria vaccine candidate |
|  | Yamana et al., | 2013 | Linking environmental variability to village-scale malaria transmission using a simple immunity model | Not related to immunogenicity, efficacy, and safety of TB/Malaria vaccine candidate |
|  | Cohen et al., | 2023 | A severe case of Plasmodium falciparum malaria imported by a French traveler from Cameroon to French Guiana despite regular intake of Artemisia annua herbal tea | Not related to immunogenicity, efficacy, and safety of TB/Malaria vaccine candidate |
|  | Sangoro et al., | 2020 | Evaluation of personal protection afforded by repellent-treated sandals against mosquito bites in south-eastern Tanzania | Not related to immunogenicity, efficacy, and safety of TB/Malaria vaccine candidate |
|  | Muvunyi and masaisa | 2012 | Diagnosis of Smear-Negative Pulmonary Tuberculosis in Low-Income Countries: Current Evidence in Sub-Saharan Africa with Special Focus on HIV Infection or AIDS | Not related to immunogenicity, efficacy, and safety of TB/Malaria vaccine candidate |
|  | Kotze et al., | 2021 | Establishment of a Patient-Derived, Magnetic Levitation-Based, Three-Dimensional Spheroid Granuloma Model for Human Tuberculosis | Not related to immunogenicity, efficacy, and safety of TB/Malaria vaccine candidate |
|  | Omondi et al., | 2021 | Antibody Responses to Crude Gametocyte Extract Predict Plasmodium falciparum Gametocyte Carriage in Kenya | Not related to immunogenicity, efficacy, and safety of TB/Malaria vaccine candidate |
|  | Gallant et al., | 2021 | PPE38-Secretion-Dependent Proteins of M. tuberculosis Alter NF-kB Signalling and Inflammatory Responses in Macrophages | Not related to immunogenicity, efficacy, and safety of TB/Malaria vaccine candidate |
|  | Rangaka et al., | 2012 | Effect of Antiretroviral Therapy on the Diagnostic Accuracy of Symptom Screening for Intensified Tuberculosis Case Finding in a South African HIV Clinic | Not related to immunogenicity, efficacy, and safety of TB/Malaria vaccine candidate |
|  | Matondo et al., | 2014 | High levels of sulphadoxine-pyrimethamine resistance Pfdhfr-Pfdhps quintuple mutations: a cross-sectional survey of six regions in Tanzania | Not related to immunogenicity, efficacy, and safety of TB/Malaria vaccine candidate |
|  | Cheallaigh et al., | 2013 | Interferon Gamma Release Assays for the Diagnosis of Latent TB Infection in HIV-Infected Individuals in a Low TB Burden Country | Not related to immunogenicity, efficacy, and safety of TB/Malaria vaccine candidate |
|  | Nhamoyebonde et al., | 2014 | Biological Differences Between the Sexes and Susceptibility to Tuberculosis | Not related to immunogenicity, efficacy, and safety of TB/Malaria vaccine candidate |
|  | Foulon et al., | 2020 | Mycolactone toxin induces an inflammatory response by targeting the IL-1 beta pathway: Mechanistic insight into Buruli ulcer pathophysiology | Not related to immunogenicity, efficacy, and safety of TB/Malaria vaccine candidate |
|  | Baird et al., | 2012 | Effect of a cash transfer programme for schooling on prevalence of HIV and herpes simplex type 2 in Malawi: A cluster randomised trial | Not related to immunogenicity, efficacy, and safety of TB/Malaria vaccine candidate |
|  | Zaharie et al., | 2020 | The immunological architecture of granulomatous inflammation in central nervous system tuberculosis | Not related to immunogenicity, efficacy, and safety of TB/Malaria vaccine candidate |
|  | Assogba et al., | 2014 | Phenotypic effects of concomitant insensitive acetylcholinesterase (ace-1(R)) and knockdown resistance (kdr(R)) in Anopheles gambiae: a hindrance for insecticide resistance management for malaria vector control | Not related to immunogenicity, efficacy, and safety of TB/Malaria vaccine candidate |
|  | Court et al., | 2019 | Effect of tablet crushing on drug exposure in the treatment of multidrug-resistant tuberculosis | Not related to immunogenicity, efficacy, and safety of TB/Malaria vaccine candidate |
|  | Nansera et al., | 2012 | Mortality and loss to follow-up among tuberculosis and HIV co-infected patients in rural southwestern Uganda | Not related to immunogenicity, efficacy, and safety of TB/Malaria vaccine candidate |
|  | Graustein et al., | 2017 | The SIGLEC14 null allele is associated with Mycobacterium tuberculosis and BCG-induced clinical and immunologic outcomes | Not related to immunogenicity, efficacy, and safety of TB/Malaria vaccine candidate |
|  | Shey et al., | 2023 | Mycobacterial-specific secretion of cytokines and chemokines in healthcare workers with apparent resistance to infection with Mycobacterium tuberculosis | Not related to immunogenicity, efficacy, and safety of TB/Malaria vaccine candidate |
|  | Holstad et al., | 2012 | Motivational groups support adherence to antiretroviral therapy and use of risk reduction behaviors in HIV positive Nigerian women: a pilot study. | Not related to immunogenicity, efficacy, and safety of TB/Malaria vaccine candidate |
|  | Swaminathan et al., | 2012 | Antigen detection as a point-of-care test for TB: the case of lipoarabinomannan | Not related to immunogenicity, efficacy, and safety of TB/Malaria vaccine candidate |
|  | Malima et al., | 2013 | Evaluation of the long-lasting insecticidal net Interceptor LN: laboratory and experimental hut studies against anopheline and culicine mosquitoes in northeastern Tanzania | Not related to immunogenicity, efficacy, and safety of TB/Malaria vaccine candidate |
|  | Olayanju et al., | 2020 | A regimen containing bedaquiline and delamanid compared to bedaquiline in patients with drug-resistant tuberculosis | Not related to immunogenicity, efficacy, and safety of TB/Malaria vaccine candidate |
|  | Shey et al., | 2012 | Optimization of a whole blood intracellular cytokine assay for measuring innate cell responses to mycobacteria | Not related to immunogenicity, efficacy, and safety of TB/Malaria vaccine candidate |
|  | Wasserman et al.., | 2021 | Plasma Pharmacokinetics of High-Dose Oral versus Intravenous Rifampicin in Patients with Tuberculous Meningitis: a Randomized Controlled Trial | Not related to immunogenicity, efficacy, and safety of TB/Malaria vaccine candidate |
|  | Ottenhoff et al., | 2012 | Genome-Wide Expression Profiling Identifies Type 1 Interferon Response Pathways in Active Tuberculosis | Not related to immunogenicity, efficacy, and safety of TB/Malaria vaccine candidate |
|  | Seo et al., | 2014 | Cost-effectiveness analysis of vaccinating children in Malawi with RTS, S vaccines in comparison with long-lasting insecticide-treated nets | Not related to immunogenicity, efficacy, and safety of TB/Malaria vaccine candidate |
|  | Badolo et al., | 2014 | Laboratory evaluation of Fendona 6SC (R) treated bednets and Interceptor (R) long-lasting nets against Anopheles gambiae s.l. in Burkina Faso | Not related to immunogenicity, efficacy, and safety of TB/Malaria vaccine candidate |
|  | Kobayashi et al., | 2019 | Distinct Antibody Signatures Associated with Different Malaria Transmission Intensities in Zambia and Zimbabwe | Not related to immunogenicity, efficacy, and safety of TB/Malaria vaccine candidate |
|  | Mvelase et al., | 2019 | rpoB Mutations Causing Discordant Rifampicin Susceptibility in Mycobacterium tuberculosis: Retrospective Analysis of Prevalence, Phenotypic, Genotypic, and Treatment Outcomes | Not related to immunogenicity, efficacy, and safety of TB/Malaria vaccine candidate |
|  | Deshpande et al., | 2016 | Azithromycin Dose to Maximize Efficacy and Suppress Acquired Drug Resistance in Pulmonary Mycobacterium avium Disease | Not related to immunogenicity, efficacy, and safety of TB/Malaria vaccine candidate |
|  | Hounkonnou et al., | 2021 | Suboptimal Intermittent Preventive Treatment in Pregnancy (IPTp) is Associated with an Increased Risk of Submicroscopic Plasmodium falciparum Infection in Pregnant Women: A Prospective Cohort Study in Benin | Not related to immunogenicity, efficacy, and safety of TB/Malaria vaccine candidate |
|  | Mbye et al., | 2022 | Plasmodium falciparum merozoite invasion ligands, linked antimalarial resistance loci and ex vivo responses to antimalarials in The Gambia | Not related to immunogenicity, efficacy, and safety of TB/Malaria vaccine candidate |
|  | Ronacher et al., | 2019 | Distinct serum biosignatures are associated with different tuberculosis treatment outcomes | Not related to immunogenicity, efficacy, and safety of TB/Malaria vaccine candidate |
|  | Fack et al., | 2022 | The impact of a change in infant BCG vaccination policy on adolescent TB incidence rates: A South African population-level cohort study | Not related to immunogenicity, efficacy, and safety of TB/Malaria vaccine candidate |
|  | Pretorious et al., | 2014 | Using the TIME model in Spectrum to estimate tuberculosis-HIV incidence and mortality | Not related to immunogenicity, efficacy, and safety of TB/Malaria vaccine candidate |
|  | Beshir et al., | 221 | Persistent Submicroscopic Plasmodium falciparum Parasitemia 72 Hours after Treatment with Artemether-Lumefantrine Predicts 42-Day Treatment Failure in Mali and Burkina Faso | Not related to immunogenicity, efficacy, and safety of TB/Malaria vaccine candidate |
|  | Ndlandla et al., | 2016 | Standardization of natural mycolic acid antigen composition and production for use in biomarker antibody detection to diagnose active tuberculosis | Not related to immunogenicity, efficacy, and safety of TB/Malaria vaccine candidate |
|  | Hughes et al., | 2020 | Quantifying late-stage host-seeking behaviour of Anopheles gambiae at the insecticidal net interface using a baited-box bioassay | Not related to immunogenicity, efficacy, and safety of TB/Malaria vaccine candidate |
|  | O et al., | 2015 | Rapid and Specific Drug Quality Testing Assay for Artemisinin and Its Derivatives Using a Luminescent Reaction and Novel Microfluidic Technology | Not related to immunogenicity, efficacy, and safety of TB/Malaria vaccine candidate |
|  | Nagu et al., | 2017 | Tuberculosis among the elderly in Tanzania: disease presentation and initial response to treatment | Not related to immunogenicity, efficacy, and safety of TB/Malaria vaccine candidate |
|  | Pettit et al., | 2019 | Directly observed therapy and risk of unfavourable tuberculosis treatment outcomes among an international cohort of people living with HIV in low- and middle-income countries | Not related to immunogenicity, efficacy, and safety of TB/Malaria vaccine candidate |
|  | Chigutsa et al., | 2013 | A Time-to-Event Pharmacodynamic Model Describing Treatment Response in  Patients with Pulmonary Tuberculosis Using Days to Positivity in Automated Liquid Mycobacterial Culture | Not related to immunogenicity, efficacy, and safety of TB/Malaria vaccine candidate |
|  | Shanley et al., | 2013 | Characterization of W-Beijing isolates of Mycobacterium tuberculosis from the Western Cape | Not related to immunogenicity, efficacy, and safety of TB/Malaria vaccine candidate |
|  | Frigati et al., | 2021 | Tuberculosis infection and disease in South African adolescents with perinatally acquired HIV on antiretroviral therapy: a cohort study | Not related to immunogenicity, efficacy, and safety of TB/Malaria vaccine candidate |
|  | Porciani et al., | 2017 | Influence of pyrethroid-treated bed net on host seeking behavior of Anopheles gambiae s. s. carrying the kdr allele | Not related to immunogenicity, efficacy, and safety of TB/Malaria vaccine candidate |
|  | E Lima et al., | 2013 | Linkage to Care and Treatment for TB and HIV among People Newly Diagnosed with TB or HIV-Associated TB at a Large, Inner-City South African Hospital | Not related to immunogenicity, efficacy, and safety of TB/Malaria vaccine candidate |
|  | Dadzie et al., | 2017 | Evaluation of piperonyl butoxide in enhancing the efficacy of pyrethroid  insecticides against resistant Anopheles gambiae s.l. in Ghana | Not related to immunogenicity, efficacy, and safety of TB/Malaria vaccine candidate |
|  | Schewo et al., | 2020 | Risk practices for bovine tuberculosis transmission to cattle and livestock farming communities living at wildlife-livestock-human interface in northern KwaZulu Natal, South Africa | Not related to immunogenicity, efficacy, and safety of TB/Malaria vaccine candidate |
|  | Marlin et al., | 2014 | The Only African Wild Tobacco, Nicotiana africana: Alkaloid Content and the Effect of Herbivory | Not related to immunogenicity, efficacy, and safety of TB/Malaria vaccine candidate |
|  | Peters et al., | 2016 | Identification of Quantitative Proteomic Differences between Mycobacterium tuberculosis Lineages with Altered Virulence | Not related to immunogenicity, efficacy, and safety of TB/Malaria vaccine candidate |
|  | Accrombessi et al., | 2019 | Effects of Malaria in the First Trimester of Pregnancy on Poor Maternal and Birth Outcomes in Benin | Not related to immunogenicity, efficacy, and safety of TB/Malaria vaccine candidate |
|  | Audibert et al., | 2021 | Perception of Malaria Chemoprevention Interventions in Infants and Children in Eight Sub-Saharan African Countries: An End User Perspective Study | Not related to immunogenicity, efficacy, and safety of TB/Malaria vaccine candidate |
|  | Sternberg et al., | 2016 | Eave tubes for malaria control in Africa: initial development and semi-field evaluations in Tanzania | Not related to immunogenicity, efficacy, and safety of TB/Malaria vaccine candidate |
|  | Davids et al., | 2021 | The Frequency and Effect of Granulocytic Myeloid-Derived Suppressor Cells on Mycobacterial Survival in Patients with Tuberculosis: A Preliminary Report | Not related to immunogenicity, efficacy, and safety of TB/Malaria vaccine candidate |
|  | Scriba et al., | 2017 | Sequential inflammatory processes define human progression from M. tuberculosis infection to tuberculosis disease | Not related to immunogenicity, efficacy, and safety of TB/Malaria vaccine candidate |
|  | Kavishe et al., | 2016 | Molecular monitoring of Plasmodium falciparum super-resistance to sulfadoxine-pyrimethamine in Tanzania | Not related to immunogenicity, efficacy, and safety of TB/Malaria vaccine candidate |
|  | Ali et al., | 2020 | Allelic frequencies of mutants of the Plasmodium falciparum, quinoline and folate metabolizing genes in the west region of Cameroon | Not related to immunogenicity, efficacy, and safety of TB/Malaria vaccine candidate |
|  | DeAtley et al., | 2021 | The child ecosystem and childhood pulmonary tuberculosis: A South African perspective | Not related to immunogenicity, efficacy, and safety of TB/Malaria vaccine candidate |
|  | Anoi et al., | 2020 | Global Repertoire of Human Antibodies Against Plasmodium falciparum RIFINs, SURFINs, and STEVORs in a Malaria Exposed Population | Not related to immunogenicity, efficacy, and safety of TB/Malaria vaccine candidate |
|  | van der Heijden et al., | 2016 | Field application of immunoassays for the detection of Mycobacterium bovis infection in the African buffalo (Syncerus caffer) | Not related to immunogenicity, efficacy, and safety of TB/Malaria vaccine candidate |
|  | Tindana et al., | 2022 | Ethical considerations in deploying triple artemisinin-based combination therapies for malaria: An analysis of stakeholders' perspectives in Burkina Faso and Nigeria | Not related to immunogenicity, efficacy, and safety of TB/Malaria vaccine candidate |
|  | Parihar et al., | 2021 | IL-4-Responsive B Cells Are Detrimental During Chronic Tuberculosis Infection in Mice | Not related to immunogenicity, efficacy, and safety of TB/Malaria vaccine candidate |
|  | Anyalechi et al., | 2022 | Tuberculosis prevalence, incidence, and prevention in a south African cohort of children living with HIV | Not related to immunogenicity, efficacy, and safety of TB/Malaria vaccine candidate |
|  | Canepa et al | 2016 | Molecular Analysis of Pfs47-Mediated Plasmodium Evasion of Mosquito Immunity | Not related to immunogenicity, efficacy, and safety of TB/Malaria vaccine candidate |
|  | Ruberto et al., | 2014 | Knowledge, attitudes, and practices of malaria control among communities from the health district of Forecariah in the Republic of Guinea, West Africa | Not related to immunogenicity, efficacy, and safety of TB/Malaria vaccine candidate |
|  | Brietb et al., | 2015 | Applications and limitations of Centers for Disease Control and Prevention miniature light traps for measuring biting densities of African malaria vector populations: a pooled analysis of 13 comparisons with human landing catches | Not related to immunogenicity, efficacy, and safety of TB/Malaria vaccine candidate |
|  | Heinrich et al., | 2015 | Early phase evaluation of SQ109 alone and in combination with rifampicin in pulmonary TB patients | Not related to immunogenicity, efficacy, and safety of TB/Malaria vaccine candidate |
|  | Aniweh et al., | 2020 | Analysis of Plasmodium falciparum Rh2b deletion polymorphism across different transmission areas | Not related to immunogenicity, efficacy, and safety of TB/Malaria vaccine candidate |
|  | Koladjo et al., | 2022 | Malaria in the First Trimester of Pregnancy and Fetal Growth: Results from a Beninese Preconceptional Cohort | Not related to immunogenicity, efficacy, and safety of TB/Malaria vaccine candidate |
|  | Den boon et al., | 2022 | WHO target product profiles for TB preventive treatment | Not related to immunogenicity, efficacy, and safety of TB/Malaria vaccine candidate |
|  | Tinto et al., | 2014 | Ex vivo anti-malarial drugs sensitivity profile of Plasmodium falciparum field isolates from Burkina Faso five years after the national policy change | Not related to immunogenicity, efficacy, and safety of TB/Malaria vaccine candidate |
|  | Abiodun et al., | 2016 | Modelling the influence of temperature and rainfall on the population dynamics of Anopheles arabiensis | Not related to immunogenicity, efficacy, and safety of TB/Malaria vaccine candidate |
|  | Khaireh et al., | 2013 | Population genetics analysis during the elimination process of Plasmodium falciparum in Djibouti | Not related to immunogenicity, efficacy, and safety of TB/Malaria vaccine candidate |
|  | Tchicaya et al., | 2014 | Micro-encapsulated pirimiphos-methyl shows high insecticidal efficacy and long residual activity against pyrethroid-resistant malaria vectors in central Cote d'Ivoire | Not related to immunogenicity, efficacy, and safety of TB/Malaria vaccine candidate |
|  | Mvubu et al., | 2016 | Canonical pathways, networks, and transcriptional factor regulation by clinical strains of Mycobacterium tuberculosis in pulmonary alveolar epithelial cells | Not related to immunogenicity, efficacy, and safety of TB/Malaria vaccine candidate |
|  | Yeebiyo et al., | 2016 | Short persistence of bendiocarb sprayed on pervious walls and its implication for the indoor residual spray program in Ethiopia | Not related to immunogenicity, efficacy, and safety of TB/Malaria vaccine candidate |
|  | Hill et al., | 2015 | Access and Use of Interventions to Prevent and Treat Malaria among Pregnant Women in Kenya and Mali: A Qualitative Study | Not related to immunogenicity, efficacy, and safety of TB/Malaria vaccine candidate |
|  | Sarfo et al., | 2013 | Microbiological, Histological, Immunological, and Toxin Response to Antibiotic Treatment in the Mouse Model of Mycobacterium ulcerans Disease | Not related to immunogenicity, efficacy, and safety of TB/Malaria vaccine candidate |
|  | Le Roex et al., | 2017 | Toll-like receptor (TLR) diversity influences mycobacterial growth in African buffalo | Not related to immunogenicity, efficacy, and safety of TB/Malaria vaccine candidate |
|  | Murray et al., | 2020 | Barrier bednets target malaria vectors and expand the range of usable insecticides | Not related to immunogenicity, efficacy, and safety of TB/Malaria vaccine candidate |
|  | Leisching et al., | 2016 | The Host Response to a Clinical MDR Mycobacterial Strain Cultured in a Detergent-Free Environment: A Global Transcriptomics Approach | Not related to immunogenicity, efficacy, and safety of TB/Malaria vaccine candidate |
|  | Pepin et al., | 2022 | In the footsteps of Albert Calmette: an ecological study of TB, leprosy, and potential exposure to wild-type Mycobacterium bovis | Not related to immunogenicity, efficacy, and safety of TB/Malaria vaccine candidate |
|  | Rathmes et al., | 2020 | Global estimation of anti-malarial drug effectiveness for the treatment of uncomplicated Plasmodium falciparum malaria 1991-2019 | Not related to immunogenicity, efficacy, and safety of TB/Malaria vaccine candidate |
|  | Mungwira et al., | 2022 | High burden of malaria among Malawian adults on antiretroviral therapy after discontinuing prophylaxis | Not related to immunogenicity, efficacy, and safety of TB/Malaria vaccine candidate |
|  | Buxton et al., | 2020 | Spatial Anopheles arabiensis (Diptera: Culicidae) insecticide resistance patterns across malaria-endemic regions of Botswana | Not related to immunogenicity, efficacy, and safety of TB/Malaria vaccine candidate |
|  | Parhboo et al., | 2022 | Persistence of Mycobacterium tuberculosis in response to infection burden and host-induced stressors | Not related to immunogenicity, efficacy, and safety of TB/Malaria vaccine candidate |
|  | Jahnmatz et al., | 2021 | Memory B-Cell Responses Against Merozoite Antigens After Acute Plasmodium Falciparum Malaria, Assessed Over One Year Using a Novel Multiplexed FluoroSpot Assay | Not related to immunogenicity, efficacy, and safety of TB/Malaria vaccine candidate |
|  | Phyo | 2017 | Challenges to replace ACT as first-line drug | Not related to immunogenicity, efficacy, and safety of TB/Malaria vaccine candidate |
|  | Chanda | 2016 | Exploiting the Potential of Integrated Vector Management for Combating Malaria in Africa | Not related to immunogenicity, efficacy, and safety of TB/Malaria vaccine candidate |
|  | Fenner et al., | 2012 | Tuberculosis in HIV-Negative and HIV-Infected Patients in a Low-Incidence Country: Clinical Characteristics and Treatment Outcomes | Not related to immunogenicity, efficacy, and safety of TB/Malaria vaccine candidate |
|  | Molina-cruz | 2014 | The remarkable journey of adaptation of the Plasmodium falciparum malaria parasite to New World anopheline mosquitoes | Not related to immunogenicity, efficacy, and safety of TB/Malaria vaccine candidate |
|  | Balkema et al., | 2014 | Tuberculosis in the intensive care unit: a prospective observational study | Not related to immunogenicity, efficacy, and safety of TB/Malaria vaccine candidate |
|  | Fofana et al., | 2022 | Differential Incidence of Malaria in Neighboring Villages in a High-Transmission Setting of Southern Mali | Not related to immunogenicity, efficacy, and safety of TB/Malaria vaccine candidate |
|  | Moiroux et al., | 2017 | Remote Effect of Insecticide-Treated Nets and the Personal Protection against Malaria Mosquito Bites | Not related to immunogenicity, efficacy, and safety of TB/Malaria vaccine candidate |
|  | Mugyenyi et al., | 2017 | Declining Malaria Transmission Differentially Impacts the Maintenance of Humoral Immunity to Plasmodium falciparum in Children | Not related to immunogenicity, efficacy, and safety of TB/Malaria vaccine candidate |
|  | Cresswell et al., | 2021 | High-Dose Oral and Intravenous Rifampicin for the Treatment of Tuberculous Meningitis in Predominantly Human Immunodeficiency Virus (HIV)-Positive Ugandan Adults: A Phase II Open-Label Randomized Controlled Trial | Not related to immunogenicity, efficacy, and safety of TB/Malaria vaccine candidate |
|  | Medina-lara et al., | 2014 | Stated preferences for anti-malarial drug characteristics in Zomba, a malaria endemic area of Malawi | Not related to immunogenicity, efficacy, and safety of TB/Malaria vaccine candidate |
|  | D'Alessandro et al., | 2012 | Malaria in infants aged less than six months - is it an area of unmet medical need? | Not related to immunogenicity, efficacy, and safety of TB/Malaria vaccine candidate |
|  | Herman et al., | 2019 | The differential impact of HIV and antiretroviral therapy on gender-specific tuberculosis rates | Not related to immunogenicity, efficacy, and safety of TB/Malaria vaccine candidate |
|  | Rasmussen et al., | 2022 | Current and emerging strategies to combat antimalarial resistance | Not related to immunogenicity, efficacy, and safety of TB/Malaria vaccine candidate |
|  | Chang et al., | 2015 | Tuberculosis Incidence and Risk Factors Among Human Immunodeficiency Virus (HIV)-Infected Adults Receiving Antiretroviral Therapy in a Large HIV Program in Nigeria | Not related to immunogenicity, efficacy, and safety of TB/Malaria vaccine candidate |
|  | Ngninpogni et al., | 2021 | Insights into factors sustaining persistence of high malaria transmission in forested areas of sub-Saharan Africa: the case of Mvoua, South Cameroon | Not related to immunogenicity, efficacy, and safety of TB/Malaria vaccine candidate |
|  | Bunyasi et al., | 2017 | Impact of isoniazid preventive therapy on the evaluation of long-term effectiveness of infant MVA85A vaccination | Secondary study |
|  | Reuter et al., | 2017 | The devil we know is the use of injectable agents for the treatment of MDR-TB justified? | Not related to immunogenicity, efficacy, and safety of TB/Malaria vaccine candidate |
|  | Kimuda et al ., | 2017 | Use of QuantiFERON (R)-TB Gold in-tube culture supernatants for measurement of antibody responses | Not related to immunogenicity, efficacy, and safety of TB/Malaria vaccine candidate |
|  | Koffi et al., | 2015 | Efficacy of Olyset (R) Duo, a permethrin and pyriproxyfen mixture net against wild pyrethroid-resistant Anopheles gambiae s.s. from Cote d'Ivoire: an experimental hut trial | Not related to immunogenicity, efficacy, and safety of TB/Malaria vaccine candidate |
|  | Sei et al., | 2019 | Opsonic monoclonal antibodies enhance phagocytic killing activity and clearance of Mycobacterium tuberculosis from blood in a quantitative qPCR mouse model | Not related to immunogenicity, efficacy, and safety of TB/Malaria vaccine candidate |
|  | Fenner et al., | 2013 | Tuberculosis in Antiretroviral Treatment Programs in Lower Income Countries: Availability and Use of Diagnostics and Screening | Not related to immunogenicity, efficacy, and safety of TB/Malaria vaccine candidate |
|  | Mupfumi et al., | 2019 | High incidence of tuberculosis in the first year of antiretroviral therapy in the Botswana National antiretroviral therapy programme between 2011 and 2015 | Not related to immunogenicity, efficacy, and safety of TB/Malaria vaccine candidate |
|  | Pollard et al., | 2019 | Human exposure to Anopheles farauti bites in the Solomon Islands is not associated with IgG antibody response to the gSG6 salivary protein of Anopheles gambiae | Not related to immunogenicity, efficacy, and safety of TB/Malaria vaccine candidate |
|  | Chen et al., | 2023 | B cells promote granulomatous inflammation during chronic Mycobacterium tuberculosis infection in mice | Not related to immunogenicity, efficacy, and safety of TB/Malaria vaccine candidate |
|  | Menard et al., | 2016 | Insight into k13-propeller gene polymorphism and ex vivo DHA-response profiles from Cameroonian isolates | Not related to immunogenicity, efficacy, and safety of TB/Malaria vaccine candidate |
|  | Peter et al., | 2012 | The Diagnostic Accuracy of Urine-Based Xpert MTB/RIF in HIV-Infected Hospitalized Patients Who Are Smear-Negative or Aputum Scarce | Not related to immunogenicity, efficacy, and safety of TB/Malaria vaccine candidate |
|  | Esmail et al., | 2020 | An Optimal Diagnostic Strategy for Tuberculosis in Hospitalized HIV-Infected Patients Using GeneXpert MTB/RIF and Alere Determine TB LAM Ag | Not related to immunogenicity, efficacy, and safety of TB/Malaria vaccine candidate |
|  | Cimperman et al., | 2023 | Cerebral Malaria Is Regulated by Host-Mediated Changes in Plasmodium Gene Expression | Not related to immunogenicity, efficacy, and safety of TB/Malaria vaccine candidate |
|  | Mueller et al., | 2014 | An Experimental Model to Study Tuberculosis-Malaria Coinfection upon Natural Transmission of Mycobacterium tuberculosis and Plasmodium berghei | Not related to immunogenicity, efficacy, and safety of TB/Malaria vaccine candidate |
|  | Hughes et al., | 2019 | Adverse events among people on delamanid for rifampicin-resistant tuberculosis in a high HIV prevalence setting | Not related to immunogenicity, efficacy, and safety of TB/Malaria vaccine candidate |
|  | Fischinger et al., | 2021 | A Mycobacterium tuberculosis Specific IgG3 Signature of Recurrent Tuberculosis | Not related to immunogenicity, efficacy, and safety of TB/Malaria vaccine candidate |
|  | Grant et al., | 2020 | Algorithm-guided empirical tuberculosis treatment for people with advanced HIV (TB Fast Track): an open-label, cluster-randomised trial | Not related to immunogenicity, efficacy, and safety of TB/Malaria vaccine candidate |
|  | Zurcher et al., | 2019 | Diagnosis and clinical outcomes of extrapulmonary tuberculosis in  antiretroviral therapy programmes in low- and middle-income countries: a multicohort study | Not related to immunogenicity, efficacy, and safety of TB/Malaria vaccine candidate |
|  | Wasserman et al., | 2022 | Linezolid toxicity in patients with drug-resistant tuberculosis: a prospective cohort study | Not related to immunogenicity, efficacy, and safety of TB/Malaria vaccine candidate |
|  | Naido et al., | 2016 | Comparing laboratory costs of smear/culture and Xpert (R) MTB/RIF-based tuberculosis diagnostic algorithms | Not related to immunogenicity, efficacy, and safety of TB/Malaria vaccine candidate |
|  | Brouwer et al., | 2013 | A case report of transfusion-transmitted Plasmodium malariae from an asymptomatic non-immune traveller | Not related to immunogenicity, efficacy, and safety of TB/Malaria vaccine candidate |
|  | Theron et al., | 2012 | Correlation of Mycobacterium Tuberculosis Specific and Non-Specific Quantitative Th1 T-Cell Responses with Bacillary Load in a High Burden Setting | Not related to immunogenicity, efficacy, and safety of TB/Malaria vaccine candidate |
|  | Zawedde-mayenje et al., | 2019 | Anti-retroviral therapy scale-up and its impact on sex-stratified tuberculosis notification trends in Uganda | Not related to immunogenicity, efficacy, and safety of TB/Malaria vaccine candidate |
|  | Ndo et al., | 2016 | High susceptibility of wild Anopheles funestus to infection with natural Plasmodium falciparum gametocytes using membrane feeding assays | Not related to immunogenicity, efficacy, and safety of TB/Malaria vaccine candidate |
|  | Gideon et al., | 2012 | Bioinformatic and Empirical Analysis of Novel Hypoxia-Inducible Targets of the Human Antituberculosis T Cell Response | Not related to immunogenicity, efficacy, and safety of TB/Malaria vaccine candidate |
|  | Tsegaye et al., | 2022 | Alteration of Endocrine Hormones and Antibody Responses in Different Spectrum of Tuberculosis Disease | Not related to immunogenicity, efficacy, and safety of TB/Malaria vaccine candidate |
|  | Schousboe et al., | 2014 | Global and local genetic diversity at two microsatellite loci in Plasmodium vivax parasites from Asia, Africa, and South America | Not related to immunogenicity, efficacy, and safety of TB/Malaria vaccine candidate |
|  | Christinet et al., | 2014 | Impact of Human Immunodeficiency Virus on the Severity of Buruli Ulcer Disease: Results of a Retrospective Study in Cameroon | Not related to immunogenicity, efficacy, and safety of TB/Malaria vaccine candidate |
|  | Ajayi | 2013 | Possible artemisinin-based combination therapy-resistant malaria in Nigeria: a report of three cases | Not related to immunogenicity, efficacy, and safety of TB/Malaria vaccine candidate |
|  | Watts et al., | 2021 | Rethinking the economic costs of hospitalization for malaria: accounting for the comorbidities of malaria patients in western Kenya | Not related to immunogenicity, efficacy, and safety of TB/Malaria vaccine candidate |
|  | Cuevas et al., | 2012 | Evaluation of Tuberculosis Diagnostics in Children: 2. Methodological Issues for Conducting and Reporting Research Evaluations of Tuberculosis Diagnostics for Intrathoracic Tuberculosis in Children. Consensus From an Expert Panel(a) | Not related to immunogenicity, efficacy, and safety of TB/Malaria vaccine candidate |
|  | Pengpid et al., | 2012 | Screening and brief intervention for alcohol problems in Dr George Mukhari Hospital out-patients in Gauteng, South Africa: A single-blinded randomized controlled trial protocol | Not related to immunogenicity, efficacy, and safety of TB/Malaria vaccine candidate |
|  | Migiliori et al., | 2012 | European Union Standards for Tuberculosis Care | Not related to immunogenicity, efficacy, and safety of TB/Malaria vaccine candidate |
|  | Jansson et al., | 2021 | Real-time dispersal of malaria vectors in rural Africa monitored with lidar | Not related to immunogenicity, efficacy, and safety of TB/Malaria vaccine candidate |
|  | Walker et al., | 2012 | Doxycycline and HIV Infection Suppress Tuberculosis-induced Matrix Metalloproteinases | Not related to immunogenicity, efficacy, and safety of TB/Malaria vaccine candidate |
|  | Auma et al., | 2013 | Malaria is an uncommon cause of adult sepsis in south-western Uganda | Not related to immunogenicity, efficacy, and safety of TB/Malaria vaccine candidate |
|  | mcNeney et al., | 2012 | Tuberculosis Diagnostics and Biomarkers: Needs, Challenges, Recent Advances, and Opportunities | Not related to immunogenicity, efficacy, and safety of TB/Malaria vaccine candidate |
|  | Anyorigiya et al., | 2021 | Loss of Toll-like receptor 7 alters cytokine production and protects against experimental cerebral malaria | Not related to immunogenicity, efficacy, and safety of TB/Malaria vaccine candidate |
|  | Baccarela et al., | 2014 | Loss of Toll-like receptor 7 alters cytokine production and protects against experimental cerebral malaria | Not related to immunogenicity, efficacy, and safety of TB/Malaria vaccine candidate |
|  | Ajileye et al., | 2017 | Some Synonymous and Nonsynonymous gyrA Mutations in Mycobacterium tuberculosis Lead to Systematic False-Positive Fluoroquinolone Resistance Results with the Hain GenoType MTBDRsl Assays | Not related to immunogenicity, efficacy, and safety of TB/Malaria vaccine candidate |
|  | Yukich et al., | 2022 | Incremental cost and cost-effectiveness of the addition of indoor residual spraying with pirimiphos-methyl in sub-Saharan Africa versus standard malaria control: results of data collection and analysis in theNext Generation Indoor Residual Sprays (NgenIRS) project, an economic evaluation | Not related to immunogenicity, efficacy, and safety of TB/Malaria vaccine candidate |
|  | Huis et al., | 2012 | The efficacy of a brief intervention to reduce alcohol misuse in patients with HIV in South Africa: Study protocol for a randomized controlled trial | Not related to immunogenicity, efficacy, and safety of TB/Malaria vaccine candidate |
|  | Mollel et al., | 2020 | Effect of tuberculosis infection on mortality of HIV-infected patients in Northern Tanzania | Not related to immunogenicity, efficacy, and safety of TB/Malaria vaccine candidate |
|  | Nambunga et al., | 2021 | Wild populations of malaria vectors can mate both inside and outside human dwellings | Not related to immunogenicity, efficacy, and safety of TB/Malaria vaccine candidate |
|  | Dooley et al., | 2023 | Assessing Pretomanid for Tuberculosis (APT), a Randomized Phase 2 Trial of Pretomanid-Containing Regimens for Drug-Sensitive Tuberculosis 12-Week Results | Not related to immunogenicity, efficacy, and safety of TB/Malaria vaccine candidate |
|  | Penny et al., | 2015 | The time-course of protection of the RTS, S vaccine against malaria infections and clinical disease | Not related to immunogenicity, efficacy, and safety of TB/Malaria vaccine candidate |
|  | Ebohon et al., | 2019 | Therapeutic failure after regimen with artemether-lumefantrine combination therapy: a report of three cases in Benin City, Nigeria | Not related to immunogenicity, efficacy, and safety of TB/Malaria vaccine candidate |
|  | Shekalaghe et al., | 2020 | Optimal timing of primaquine to reduce Plasmodium falciparum gametocyte carriage when co-administered with artemether-lumefantrine | Not related to immunogenicity, efficacy, and safety of TB/Malaria vaccine candidate |
|  | Yerlikaya et al., | 2022 | A Dual, Systematic Approach to Malaria Diagnostic Biomarker Discovery | Not related to immunogenicity, efficacy, and safety of TB/Malaria vaccine candidate |
|  | Teneza-Mora et al., | 2015 | A malaria vaccine for travelers and military personnel: Requirements and top candidates | Not related to immunogenicity, efficacy, and safety of TB/Malaria vaccine candidate |
|  | Oni et al., | 2012 | Risk Factors Associated with Indeterminate Gamma Interferon Responses in the Assessment of Latent Tuberculosis Infection in a High-Incidence Environment | Not related to immunogenicity, efficacy, and safety of TB/Malaria vaccine candidate |
|  | Achar et al., | 2017 | Off-Label Use of Bedaquiline in Children and Adolescents with Multidrug-Resistant Tuberculosis | Not related to immunogenicity, efficacy, and safety of TB/Malaria vaccine candidate |
|  | Pandie et al., | 2016 | The diagnostic accuracy of pericardial and urinary lipoarabinomannan (LAM) assays in patients with suspected tuberculous pericarditis | Not related to immunogenicity, efficacy, and safety of TB/Malaria vaccine candidate |
|  | Madhvi et al., | 2022 | Comparison of Cytokines Expression from Human Monocyte-Derived Macrophages Infected with Different Species of Mycobacteria | Not related to immunogenicity, efficacy, and safety of TB/Malaria vaccine candidate |
|  | Fauconnier et al., | 2012 | IL-12R beta 2 Is Essential for the Development of Experimental Cerebral Malaria | Not related to immunogenicity, efficacy, and safety of TB/Malaria vaccine candidate |
|  | Che et al., | 2015 | Chemokines responses to Plasmodium falciparum malaria and co-infections among rural Cameroonians | Not related to immunogenicity, efficacy, and safety of TB/Malaria vaccine candidate |
|  | Slater et al., | 2015 | Assessing the impact of next-generation rapid diagnostic tests on Plasmodium falciparum malaria elimination strategies | Not related to immunogenicity, efficacy, and safety of TB/Malaria vaccine candidate |
|  | Beckwith et al., | 2021 | Causes and Outcomes of Admission and Investigation of Tuberculosis in Adults with Advanced HIV in South African Hospitals: Data from the TB Fast Track Trial | Not related to immunogenicity, efficacy, and safety of TB/Malaria vaccine candidate |
|  | Monrie et al., | 2021 | Methods and indicators for measuring patterns of human exposure to malaria vectors | Not related to immunogenicity, efficacy, and safety of TB/Malaria vaccine candidate |
|  | Kay et al., | 2019 | Evaluation of the QuantiFERON-Tuberculosis Gold Plus Assay in Children with Tuberculosis Disease or Following Household Exposure to Tuberculosis | Not related to immunogenicity, efficacy, and safety of TB/Malaria vaccine candidate |
|  | Migiliori et al., | 2022 | Clinical standards for the diagnosis, treatment, and prevention of TB infection | Not related to immunogenicity, efficacy, and safety of TB/Malaria vaccine candidate |
|  | Morahan et al., | 2020 | Human Aurora kinase inhibitor Hesperadin reveals epistatic interaction between Plasmodium falciparum PfArk1 and PfNek1 kinases | Not related to immunogenicity, efficacy, and safety of TB/Malaria vaccine candidate |
|  | Tenforde et al., | 2015 | C-Reactive Protein (CRP), Interferon Gamma-Inducible Protein 10 (IP-10), and Lipopolysaccharide (LPS) Are Associated with Risk of Tuberculosis after Initiation of Antiretroviral Therapy in Resource-Limited Settings | Not related to immunogenicity, efficacy, and safety of TB/Malaria vaccine candidate |
|  | Moodley et al., | 2022 | Reverse vaccinology approach to design a multi-epitope vaccine construct based on the Mycobacterium tuberculosis biomarker PE_PGRS17 | Not related to immunogenicity, efficacy, and safety of TB/Malaria vaccine candidate |
|  | Denz et al., | 2021 | Predicting the impact of outdoor vector control interventions on malaria transmission intensity from semi-field studies | Not related to immunogenicity, efficacy, and safety of TB/Malaria vaccine candidate |
|  | Jones et al., | 2022 | Lyl1-deficiency promotes inflammatory responses and increases mycobacterial burden in response to Mycobacterium tuberculosis infection in mice | Not related to immunogenicity, efficacy, and safety of TB/Malaria vaccine candidate |
|  | Ludlow et al., | 2012 | HIV-1 Inhibits Phagocytosis and Inflammatory Cytokine Responses of Human Monocyte-Derived Macrophages to P-falciparum Infected Erythrocytes | Not related to immunogenicity, efficacy, and safety of TB/Malaria vaccine candidate |
|  | Cherif et al., | 2016 | Distribution of Fc gamma R gene polymorphisms among two sympatric populations in Mali: differing allele frequencies, associations with malariometric indices and implications for genetic susceptibility to malaria | Not related to immunogenicity, efficacy, and safety of TB/Malaria vaccine candidate |
|  | Garcia et al., | 2023 | Identifying individual, household, and environmental risk factors for malaria infection on Bioko Island to inform interventions | Not related to immunogenicity, efficacy, and safety of TB/Malaria vaccine candidate |
|  | Vekemans et al., | 2021 | Meeting report: WHO consultation on malaria vaccine development, Geneva, 15-16 July 2019 | Not related to immunogenicity, efficacy, and safety of TB/Malaria vaccine candidate |
|  | Krakauer et al., | 2019 | Palliative care and symptom relief for people affected by multidrug-resistant tuberculosis | Not related to immunogenicity, efficacy, and safety of TB/Malaria vaccine candidate |
|  | Esmail et al., | 2022 | An All-Oral 6-Month Regimen for Multidrug-Resistant Tuberculosis A Multicenter, Randomized Controlled Clinical Trial (the NExT Study) | Not related to immunogenicity, efficacy, and safety of TB/Malaria vaccine candidate |
|  | Fanello et al., | 2021 | Pharmacokinetic Study of Rectal Artesunate in Children with Severe Malaria in Africa | Not related to immunogenicity, efficacy, and safety of TB/Malaria vaccine candidate |
|  | Monroe et al., | 2020 | Patterns of human exposure to malaria vectors in Zanzibar and implications for malaria elimination efforts | Not related to immunogenicity, efficacy, and safety of TB/Malaria vaccine candidate |
|  | Kibret et al., | 2012 | How does an Ethiopian dam increase malaria? Entomological determinants around the Koka reservoir | Not related to immunogenicity, efficacy, and safety of TB/Malaria vaccine candidate |
|  | Ugboaja et al., | 2017 | Efficacy of intermittent preventive treatment and insecticide treated nets on malaria parasitaemia in pregnancy among Igbo women in southeastern Nigeria | Not related to immunogenicity, efficacy, and safety of TB/Malaria vaccine candidate |
|  | Mueller et al., | 2012 | Natural Transmission of Plasmodium berghei Exacerbates Chronic Tuberculosis in an Experimental Co-Infection Model | Not related to immunogenicity, efficacy, and safety of TB/Malaria vaccine candidate |
|  | Bradley et al., | 2015 | Rapid antibiotic-resistance predictions from genome sequence data for Staphylococcus aureus and Mycobacterium tuberculosis | Not related to immunogenicity, efficacy, and safety of TB/Malaria vaccine candidate |
|  | Tetteh et al., | 2021 | Acute Phase Responses Vary Between Children of HbAS and HbAA Genotypes During Plasmodium falciparum Infection | Not related to immunogenicity, efficacy, and safety of TB/Malaria vaccine candidate |
|  | Moodliar et al., | 2021 | Bedaquiline for multidrug-resistant TB in paediatric patients | Not related to immunogenicity, efficacy, and safety of TB/Malaria vaccine candidate |
|  | Gbedande et al., | 2017 | Clinical development of a VAR2CSA-based placental malaria vaccine PAMVAC: Quantifying vaccine antigen-specific memory B & T cell activity in Beninese primigravidae | Not related to immunogenicity, efficacy, and safety of TB/Malaria vaccine candidate |
|  | Frankel et al., | 2016 | Different effects of BCG strains - A natural experiment evaluating the impact of the Danish and the Russian BCG strains on morbidity and scar formation in Guinea-Bissau | Not related to immunogenicity, efficacy, and safety of TB/Malaria vaccine candidate |
|  | Waite et al., | 2016 | Eave tubes for malaria control in Africa: a modelling assessment of potential impact on transmission | Not related to immunogenicity, efficacy, and safety of TB/Malaria vaccine candidate |
|  | Andrews et al., | 2012 | The cost-effectiveness of routine tuberculosis screening with Xpert MTB/RIF prior to initiation of antiretroviral therapy: a model-based analysis | Not related to immunogenicity, efficacy, and safety of TB/Malaria vaccine candidate |
|  | Fatima et al., | 2012 | Estimating tuberculosis burden and case detection in Pakistan | Not related to immunogenicity, efficacy, and safety of TB/Malaria vaccine candidate |
|  | Sarathy et al., | 2023 | A Novel Tool to Identify Bactericidal Compounds against Vulnerable Targets in Drug-Tolerant M. tuberculosis found in Caseum | Not related to immunogenicity, efficacy, and safety of TB/Malaria vaccine candidate |
|  | O'Flaherty et al., | 2022 | Anti-Gametocyte Antigen Humoral Immunity and Gametocytemia During Treatment of Uncomplicated Falciparum Malaria: A Multi-National Study | Not related to immunogenicity, efficacy, and safety of TB/Malaria vaccine candidate |
|  | Cordel et al., | 2013 | Atovaquone-proguanil in the treatment of imported uncomplicated Plasmodium falciparum malaria: a prospective observational study of 553 cases | Not related to immunogenicity, efficacy, and safety of TB/Malaria vaccine candidate |
|  | Watson-jones et al., | 2012 | Reasons for Receiving or Not Receiving HPV Vaccination in Primary Schoolgirls in Tanzania: A Case Control Study | Not related to immunogenicity, efficacy, and safety of TB/Malaria vaccine candidate |
|  | Ezeamama et al., | 2012 | HIV Infection and the Incidence of Malaria Among HIV-Exposed Children from Tanzania | Not related to immunogenicity, efficacy, and safety of TB/Malaria vaccine candidate |
|  | Latka et al., | 2012 | Pregnancy incidence and correlates during the HVTN 503 phambili HIV vaccine trial conducted among south african women | Not related to immunogenicity, efficacy, and safety of TB/Malaria vaccine candidate |
|  | Wecker et al., | 2012 | Phase I safety and immunogenicity evaluations of an alphavirus replicon HIV-1 subtype C gag vaccine in healthy HIV-1-uninfected adults | Non-TB/malaria vaccination study, |
|  | Apinjol et al., | 2022 | Intermittent preventive treatment with Sulphadoxine-Pyrimethamine (IPTp-SP) is associated with protection against sub-microscopic P. falciparum infection in pregnant women during the low transmission dry season in southwestern Cameroon: A Semi-longitudinal study | Not related to immunogenicity, efficacy, and safety of TB/Malaria vaccine candidate and |
|  | Kobune et al., | 2012 | Symbiotic Fungal Flora in Leaf Galls Induced by Illiciomyia yukawai (Diptera: Cecidomyiidae) and in Its Mycangia | Not related to immunogenicity, efficacy, and safety of TB/Malaria vaccine candidate |
|  | Ursing et al., | 2014 | Chloroquine Is Grossly Under Dosed in Young Children with Malaria: Implications for Drug Resistance | Not related to immunogenicity, efficacy, and safety of TB/Malaria vaccine candidate |
|  | Reid et al., | 2012 | Implementation of tuberculosis infection control measures at HIV care and treatment sites in sub-Saharan Africa | Not related to immunogenicity, efficacy, and safety of TB/Malaria vaccine candidate |
|  | Mouhamadou et al., | 2019 | Evidence of insecticide resistance selection in wild Anopheles coluzzii mosquitoes due to agricultural pesticide use | Not related to immunogenicity, efficacy, and safety of TB/Malaria vaccine candidate |
|  | Baraka et al., | 2015 | A Bundle of Services Increased Ascertainment of Tuberculosis among HIV-Infected Individuals Enrolled in a HIV Cohort in Rural Sub-Saharan Africa | Not related to immunogenicity, efficacy, and safety of TB/Malaria vaccine candidate |
|  | Lawn et al., | 2012 | Clinical significance of lipoarabinomannan detection in urine using a low-cost point-of-care diagnostic assay for HIV-associated tuberculosis | Not related to immunogenicity, efficacy, and safety of TB/Malaria vaccine candidate |
|  | Opiyo et al., | 2021 | Sub-lethal aquatic doses of pyriproxyfen may increase pyrethroid resistance in malaria mosquitoes | Not related to immunogenicity, efficacy, and safety of TB/Malaria vaccine candidate |
|  | Msellem et al., | 2020 | Increased Sensitivity of Plasmodium falciparum to Artesunate/Amodiaquine Despite 14 Years as First-Line Malaria Treatment, Zanzibar | Not related to immunogenicity, efficacy, and safety of TB/Malaria vaccine candidate |
|  | Du Bruyn et al., | 2021 | Inflammatory profile of patients with tuberculosis with or without HIV-1 co-infection: a prospective cohort study and immunological network analysis | Not related to immunogenicity, efficacy, and safety of TB/Malaria vaccine candidate |
|  | Drame et al., | 2013 | Evaluation of the Effectiveness of Malaria Vector Control Measures in Urban Settings of Dakar by a Specific Anopheles Salivary Biomarker | Not related to immunogenicity, efficacy, and safety of TB/Malaria vaccine candidate |
|  | Wang et al., | 2015 | Population dynamics and associated factors of cereal aphids and armyworms under global change | Not related to immunogenicity, efficacy, and safety of TB/Malaria vaccine candidate |
|  | Ochomo et al., | 2013 | The efficacy of long-lasting nets with declining physical integrity may be compromised in areas with high levels of pyrethroid resistance | Not related to immunogenicity, efficacy, and safety of TB/Malaria vaccine candidate |
|  | Dabire et al., | 2014 | Distribution and Frequency of kdr Mutations within Anopheles gambiae s.I. Populations and First Report of the Ace. 1G119S Mutation in Anopheles arabiensis from Burkina Faso (West Africa) | Not related to immunogenicity, efficacy, and safety of TB/Malaria vaccine candidate |
|  | Lee et al., | 2012 | Identification of Optimal Epitopes for Plasmodium falciparum Rapid Diagnostic Tests That Target Histidine-Rich Proteins 2 and 3 | Not related to immunogenicity, efficacy, and safety of TB/Malaria vaccine candidate |
|  | Lumsden et al., | 2012 | Evaluation of immune responses to a Plasmodium vivax CSP-based recombinant protein vaccine candidate in combination with second-generation adjuvants in mice | Not related to immunogenicity, efficacy, and safety of TB/Malaria vaccine candidate |
|  | Bourke et al., | 2022 | Comparison of total immunoglobulin G antibody responses to different protein fragments of Plasmodium vivax Reticulocyte binding protein 2b | Not related to immunogenicity, efficacy, and safety of TB/Malaria vaccine candidate |
|  | Sanou et al., | 2021 | Insecticide resistance and behavioural adaptation as a response to long-lasting insecticidal net deployment in malaria vectors in the Cascades region of Burkina Faso | Not related to immunogenicity, efficacy, and safety of TB/Malaria vaccine candidate |
|  | Brunner et al., | 2019 | The potential of pregnant women as a sentinel population for malaria surveillance | Not related to immunogenicity, efficacy, and safety of TB/Malaria vaccine candidate |
|  | Genton | 2012 | Malaria Prevention in Travelers | Not related to immunogenicity, efficacy, and safety of TB/Malaria vaccine candidate |
|  | Obboh et al., | 2020 | Large Variations in Malaria Parasite Carriage by Afebrile School Children Living in Nearby Communities in the Central Region of Ghana | Not related to immunogenicity, efficacy, and safety of TB/Malaria vaccine candidate |
|  | Roos et al., | 2019 | Cytokine gene expression assay as a diagnostic tool for detection of Mycobacterium bovis infection in warthogs (Phacochoerus africanus) | Not related to immunogenicity, efficacy, and safety of TB/Malaria vaccine candidate |
|  | Guindo et al., | 2021 | Improved BioGents (R) Sentinel trap with heat (BGSH) for outdoor collections of Anopheline species in Burkina Faso and Mali, West Africa | Not related to immunogenicity, efficacy, and safety of TB/Malaria vaccine candidate |
|  | Whitfield et al., | 2019 | Comparative Performance of Genomic Methods for the Detection of Pyrazinamide Resistance and Heteroresistance in Mycobacterium tuberculosis | Not related to immunogenicity, efficacy, and safety of TB/Malaria vaccine candidate |
|  | Hamasur et al., | 2015 | A Sensitive Urinary Lipoarabinomannan Test for Tuberculosis | Not related to immunogenicity, efficacy, and safety of TB/Malaria vaccine candidate |
|  | Abdelwahab et al., | 2021 | Linezolid Population Pharmacokinetics in South African Adults with Drug-Resistant Tuberculosis | Not related to immunogenicity, efficacy, and safety of TB/Malaria vaccine candidate |
|  | Dagg et al., | 2019 | Evaluation of toxicity of clothianidin (neonicotinoid) and chlorfenapyr (pyrrole) insecticides and cross-resistance to other public health insecticides in Anopheles arabiensis from Ethiopia | Not related to immunogenicity, efficacy, and safety of TB/Malaria vaccine candidate |
|  | Agossa et al., | 2014 | Laboratory and field evaluation of the impact of washings on the effectiveness of LifeNet (R), Olyset (R) and PermaNet (R) 2.0 in two areas, where there is a high level of resistance of Anopheles gambiae to pyrethroids, Benin, West Africa | Not related to immunogenicity, efficacy, and safety of TB/Malaria vaccine candidate |
|  | Ochwedo et al., | 2022 | Signatures of selection and drivers for novel mutation on transmission-blocking vaccine candidate Pfs25 gene in western Kenya | Not related to immunogenicity, efficacy, and safety of TB/Malaria vaccine candidate |
|  | Wombo et al., | 2023 | Knowledge, attitudes, and practices of mothers regarding childhood malaria in southeastern Gabon | Not related to immunogenicity, efficacy, and safety of TB/Malaria vaccine candidate |
|  | Tsenova et al., | 2020 | Inoculum size and traits of the infecting clinical strain define the protection level against Mycobacterium tuberculosis infection in a rabbit model | Not related to immunogenicity, efficacy, and safety of TB/Malaria vaccine candidate |
|  | Kerkoff et al., | 2012 | Systematic Review of TST Responses in People Living with HIV in Under-Resourced Settings: Implications for Isoniazid Preventive Therapy | Not related to immunogenicity, efficacy, and safety of TB/Malaria vaccine candidate |
|  | Ntivuruguzwa et al., | 2022 | Prevalence of bovine tuberculosis and characterization of the members of the Mycobacterium tuberculosis complex from slaughtered cattle in Rwanda | Not related to immunogenicity, efficacy, and safety of TB/Malaria vaccine candidate |
|  | Manjurano et al., | 2012 | Candidate Human Genetic Polymorphisms and Severe Malaria in a Tanzanian Population | Not related to immunogenicity, efficacy, and safety of TB/Malaria vaccine candidate |
|  | Jensen et al., | 2015 | Heterologous Immunological Effects of Early BCG Vaccination in Low-Birth-Weight Infants in Guinea-Bissau: A Randomized-controlled Trial | Not related to immunogenicity, efficacy, and safety of TB/Malaria vaccine candidate |
|  | Van Der Walt et al., | 2021 | Treatment Outcomes and Adverse Drug Effects of Ethambutol, Cycloserine, and Terizidone for the Treatment of Multidrug-Resistant Tuberculosis in South Africa | Not related to immunogenicity, efficacy, and safety of TB/Malaria vaccine candidate |
|  | Deshpande et al., | 2016 | A Faropenem, Linezolid, and Moxifloxacin Regimen for Both Drug-Susceptible and Multidrug-Resistant Tuberculosis in Children: FLAME Path on the Milky Way | Not related to immunogenicity, efficacy, and safety of TB/Malaria vaccine candidate |
|  | Lachenal et al., | 2020 | Setting up pharmacovigilance based on available endTB Project data for bedaquiline | Not related to immunogenicity, efficacy, and safety of TB/Malaria vaccine candidate |
|  | Sy et al., | 2021 | Genomic investigation of atypical malaria cases in Kanel, northern Senegal | Not related to immunogenicity, efficacy, and safety of TB/Malaria vaccine candidate |
|  | Hodgson et al., | 2015 | Lessons learnt from the first controlled human malaria infection study conducted in Nairobi, Kenya | Not related to immunogenicity, efficacy, and safety of TB/Malaria vaccine candidate |
|  | Aregawi et al., | 2017 | Effect of anti-malarial interventions on trends of malaria cases, hospital admissions and deaths, 2005-2015, Ghana | Not related to immunogenicity, efficacy, and safety of TB/Malaria vaccine candidate |
|  | Lalani et al., | 2015 | Substandard Antimalarials Available in Afghanistan: A Case for Assessing the Quality of Drugs in Resource Poor Settings | Not related to immunogenicity, efficacy, and safety of TB/Malaria vaccine candidate |
|  | Okumu et al., | 2021 | Key Characteristics of Residual Malaria Transmission in Two Districts in South-Eastern Tanzania Implications for Improved Control | Not related to immunogenicity, efficacy, and safety of TB/Malaria vaccine candidate |
|  | Landier et al., | 2015 | Seasonal Patterns of Buruli Ulcer Incidence, Central Africa, 2002-2012 | Not related to immunogenicity, efficacy, and safety of TB/Malaria vaccine candidate |
|  | Pooran et al., | 2019 | IL-4 subverts mycobacterial containment in Mycobacterium tuberculosis-infected human macrophages | Not related to immunogenicity, efficacy, and safety of TB/Malaria vaccine candidate |
|  | Moreira-Teixeira et al., | 2020 | Mouse transcriptome reveals potential signatures of protection and pathogenesis in human tuberculosis | Not related to immunogenicity, efficacy, and safety of TB/Malaria vaccine candidate |
|  | Ibara-Okabande et al., | 2012 | Reduction of multiplicity of infections but no change in msp2 genetic diversity in Plasmodium falciparum isolates from Congolese children after introduction of artemisinin-combination therapy | Not related to immunogenicity, efficacy, and safety of TB/Malaria vaccine candidate |
|  | Fullman et al., | 2013 | Nets, spray or both? The effectiveness of insecticide-treated nets and indoor residual spraying in reducing malaria morbidity and child mortality in sub-Saharan Africa | Not related to immunogenicity, efficacy, and safety of TB/Malaria vaccine candidate |
|  | Musasia et al., | 2022 | Phagocytosis of Plasmodium falciparum ring-stage parasites predicts protection against malaria | Not related to immunogenicity, efficacy, and safety of TB/Malaria vaccine candidate |
|  | Vinnemeier et al., | 2017 | Response to fever and utilization of standby emergency treatment (SBET) for malaria in travellers to Southeast Asia: a questionnaire-based cohort study | Not related to immunogenicity, efficacy, and safety of TB/Malaria vaccine candidate |
|  | Beyene et al., | 2017 | Concurrent Plasmodium infection, anemia and their correlates among newly diagnosed people living with HIV/AIDS in Northern Ethiopia | Not related to immunogenicity, efficacy, and safety of TB/Malaria vaccine candidate |
|  | Falade et al., | 2019 | Consequences of restricting antimalarial drugs to rapid diagnostic test-positive febrile children in south-west Nigeria | Not related to immunogenicity, efficacy, and safety of TB/Malaria vaccine candidate |
|  | Allen et al., | 2013 | Evaluating harm associated with anti-malarial drugs: a survey of methods used by clinical researchers to elicit, assess and record participant-reported adverse events and related data | Not related to immunogenicity, efficacy, and safety of TB/Malaria vaccine candidate |
|  | Desai et al., | 2015 | Impact of Sulfadoxine-Pyrimethamine Resistance on Effectiveness of Intermittent Preventive Therapy for Malaria in Pregnancy at Clearing Infections and Preventing Low Birth Weight | Not related to immunogenicity, efficacy, and safety of TB/Malaria vaccine candidate |
|  | Hughes et al., | 2020 | Piperaquine Exposure Is Altered by Pregnancy, HIV, and Nutritional Status in Ugandan Women | Not related to immunogenicity, efficacy, and safety of TB/Malaria vaccine candidate |
|  | Lewis et al., | 2012 | ``Proof-Of-Concept'' Evaluation of an Automated Sputum Smear Microscopy System for Tuberculosis Diagnosis | Not related to immunogenicity, efficacy, and safety of TB/Malaria vaccine candidate |
|  | Buffen et al., | 2014 | Autophagy Controls BCG-Induced Trained Immunity and the Response to Intravesical BCG Therapy for Bladder Cancer | Not related to immunogenicity, efficacy, and safety of TB/Malaria vaccine candidate |
|  | Ng’ang’a et al., | 2020 | Evaluating effectiveness of screening house eaves as a potential intervention for reducing indoor vector densities and malaria prevalence in Nyabondo, western Kenya | Not related to immunogenicity, efficacy, and safety of TB/Malaria vaccine candidate |
|  | Cliff et al., | 2016 | Excessive Cytolytic Responses Predict Tuberculosis Relapse After Apparently Successful Treatment | Not related to immunogenicity, efficacy, and safety of TB/Malaria vaccine candidate |
|  | Nagot et al., | 2012 | Lopinavir/Ritonavir versus Lamivudine peri-exposure prophylaxis to prevent HIV-1 transmission by breastfeeding: the PROMISE-PEP trial Protocol ANRS 12174 | Not related to immunogenicity, efficacy, and safety of TB/Malaria vaccine candidate |
|  | Kilian et al., | 2015 | Field durability of the same type of long-lasting insecticidal net varies between regions in Nigeria due to differences in household behaviour and living conditions | Not related to immunogenicity, efficacy, and safety of TB/Malaria vaccine candidate |
|  | Akanbi et al., | 2017 | Evaluation of gene xpert for routine diagnosis of HIV-associated tuberculosis in Nigeria: A prospective cohort study | Not related to immunogenicity, efficacy, and safety of TB/Malaria vaccine candidate |
|  | McEvoy et al., | 2012 | Comparative Analysis of Mycobacterium tuberculosis pe and ppe GenesReveals High Sequence Variation and an Apparent Absence of Selective Constraints | Not related to immunogenicity, efficacy, and safety of TB/Malaria vaccine candidate |
|  | Massuse et al., | 2016 | Durability of Olyset campaign nets distributed between 2009 and 2011 in eight districts of Tanzania | Not related to immunogenicity, efficacy, and safety of TB/Malaria vaccine candidate |
|  | Vanden et al., | 2013 | The Cough Cylinder: a tool to study measures against airborne spread of (myco-) bacteria | Not related to immunogenicity, efficacy, and safety of TB/Malaria vaccine candidate |
|  | Keita et al., | 2021 | Multiple Resistance Mechanisms to Pyrethroids Insecticides in Anopheles gambiae sensu lato Population from Mali, West Africa | Not related to immunogenicity, efficacy, and safety of TB/Malaria vaccine candidate |
|  | Peter et al., | 2012 | Diagnosis of TB: state of the art | Not related to immunogenicity, efficacy, and safety of TB/Malaria vaccine candidate |
|  | Stoney et al., | 2012 | Malaria Prevention Strategies: Adherence among Boston Area Travelers Visiting Malaria-Endemic Countries | Not related to immunogenicity, efficacy, and safety of TB/Malaria vaccine candidate |
|  | Nana et al., | 2023 | Intermittent preventive treatment with Sulfadoxine pyrimethamine for malaria: a global overview and challenges affecting optimal drug uptake in pregnant women | Not related to immunogenicity, efficacy, and safety of TB/Malaria vaccine candidate |
|  | Fukuda et al., | 2021 | Ex vivo susceptibility of Plasmodium falciparum to antimalarial drugs in Northern Uganda | Not related to immunogenicity, efficacy, and safety of TB/Malaria vaccine candidate |
|  | Takem et al., | 2013 | Detecting Foci of Malaria Transmission with School Surveys: A Pilot Study in the Gambia | Not related to immunogenicity, efficacy, and safety of TB/Malaria vaccine candidate |
|  | Barone et al., | 2012 | Non-inferiority of short-term urethral catheterization following fistula repair surgery: Study protocol for a randomized controlled trial | Not related to immunogenicity, efficacy, and safety of TB/Malaria vaccine candidate |
|  | Mthiyane et al., | 2019 | Urine lipoarabinomannan (LAM) and antimicrobial usage in seriously ill HIV-infected patients with sputum smear-negative pulmonary tuberculosis | Not related to immunogenicity, efficacy, and safety of TB/Malaria vaccine candidate |
|  | Gallant et al., | 2016 | Glutamate Dehydrogenase Is Required by Mycobacterium bovis BCG for Resistance to Cellular Stress | Not related to immunogenicity, efficacy, and safety of TB/Malaria vaccine candidate |
|  | Marais et al., | 2012 | Prevention of TB in areas of high incidence | Not related to immunogenicity, efficacy, and safety of TB/Malaria vaccine candidate |
|  | Uyoga et al., | 2019 | The indirect health effects of malaria estimated from health advantages of the sickle cell trait | Not related to immunogenicity, efficacy, and safety of TB/Malaria vaccine candidate |
|  | Meintjes et al., | 2012 | Corticosteroid-modulated Immune Activation in the Tuberculosis Immune Reconstitution Inflammatory Syndrome | Not related to immunogenicity, efficacy, and safety of TB/Malaria vaccine candidate |
|  | Chengalroyen et al., | 2016 | Detection and Quantification of Differentially Culturable Tubercle Bacteria in Sputum from Patients with Tuberculosis | Not related to immunogenicity, efficacy, and safety of TB/Malaria vaccine candidate |
|  | Meldau et al., | 2019 | Same-Day Tools, Including Xpert Ultra and IRISA-TB, for Rapid Diagnosis of Pleural Tuberculosis: a Prospective Observational Study | Not related to immunogenicity, efficacy, and safety of TB/Malaria vaccine candidate |
|  | Sanders et al., | 2014 | Antimalarial Efficacy of Hydroxyethylapoquinine (SN-119) and Its Derivatives | Not related to immunogenicity, efficacy, and safety of TB/Malaria vaccine candidate |
|  | Madongo et al | 2015 | Amikacin Concentrations Predictive of Ototoxicity in Multidrug-Resistant Tuberculosis Patients | Not related to immunogenicity, efficacy, and safety of TB/Malaria vaccine candidate |
|  | Kassam et al., | 2016 | Assets and challenges facing caregivers when managing malaria in young children in rural Uganda | Not related to immunogenicity, efficacy, and safety of TB/Malaria vaccine candidate |
|  | Onyamboko et al., | 2020 | A Randomized Controlled Trial of Three- versus Five-Day Artemether-Lumefantrine Regimens for Treatment of Uncomplicated Plasmodium falciparum Malaria in Pregnancy in Africa | Not related to immunogenicity, efficacy, and safety of TB/Malaria vaccine candidate |
|  | Manabe et al., | 2016 | Prevention of Early Mortality by Presumptive Tuberculosis Therapy Study: An Open Label, Randomized Controlled Trial | Not related to immunogenicity, efficacy, and safety of TB/Malaria vaccine candidate |
|  | Skerry et al., | 2012 | Adjunctive TNF Inhibition with Standard Treatment Enhances Bacterial Clearance in a Murine Model of Necrotic TB Granulomas | Not related to immunogenicity, efficacy, and safety of TB/Malaria vaccine candidate |
|  | Lukwa et al., | 2019 | Effect of malaria on productivity in a workplace: the case of a banana plantation in Zimbabwe | Not related to immunogenicity, efficacy, and safety of TB/Malaria vaccine candidate |
|  | Padayatchi et al., | 2014 | Improved survival in multidrug-resistant tuberculosis patients receiving integrated tuberculosis and antiretroviral treatment in the SAPiT Trial | Not related to immunogenicity, efficacy, and safety of TB/Malaria vaccine candidate |
|  | Kanoi et al., | 2017 | Antibody profiles to wheat germ cell-free system synthesized Plasmodium falciparum proteins correlate with protection from symptomatic malaria in Uganda | Not related to immunogenicity, efficacy, and safety of TB/Malaria vaccine candidate |
|  | Rusell et al., | 2015 | Determinants of Bed Net Use in Southeast Nigeria following Mass Distribution of LLINs: Implications for Social Behavior Change Interventions | Not related to immunogenicity, efficacy, and safety of TB/Malaria vaccine candidate |
|  | Exavery et al., | 2014 | Factors affecting uptake of optimal doses of sulphadoxine-pyrimethamine for intermittent preventive treatment of malaria in pregnancy in six districts of Tanzania | Not related to immunogenicity, efficacy, and safety of TB/Malaria vaccine candidate |
|  | Hamed et al., | 2012 | Coartem (R): a decade of patient-centric malaria management | Not related to immunogenicity, efficacy, and safety of TB/Malaria vaccine candidate |
|  | Ahmed et al., | 2022 | Mycobacterium tuberculosis senses host Interferon-gamma via the membrane protein MmpL10 | Not related to immunogenicity, efficacy, and safety of TB/Malaria vaccine candidate |
|  | Rumisha et al., | 2013 | Assessing seasonal variations and age patterns in mortality during the first year of life in Tanzania | Not related to immunogenicity, efficacy, and safety of TB/Malaria vaccine candidate |
|  | Bourigautt et al., | 2014 | Relative contribution of IL-1 alpha, IL-1 beta and TNF to the host response to Mycobacterium tuberculosis and attenuated M. bovis BCG | Not related to immunogenicity, efficacy, and safety of TB/Malaria vaccine candidate |
|  | Paul et al., | 2016 | Prevalence of Plasmodium falciparum transmission reducing immunity among primary school children in a malaria moderate transmission region in Zimbabwe | Not related to immunogenicity, efficacy, and safety of TB/Malaria vaccine candidate |
|  | Adu-gyasi et al., | 2013 | Prevalence of clinically captured and confirmed malaria among HIVs seropositve clinic attendants in five hospitals in Ghana | Not related to immunogenicity, efficacy, and safety of TB/Malaria vaccine candidate |
|  | Idris et al., | 2017 | Serological measures to assess the efficacy of malaria control programme on Ambae Island, Vanuatu | Not related to immunogenicity, efficacy, and safety of TB/Malaria vaccine candidate |
|  | Torrens et al., | 2016 | Effectiveness of a conditional cash transfer programme on TB cure rate: a retrospective cohort study in Brazil | Not related to immunogenicity, efficacy, and safety of TB/Malaria vaccine candidate |
|  | Nguetse et al., | 2016 | Glucose-6-phosphate dehydrogenase deficiency and reduced haemoglobin levels in African children with severe malaria | Not related to immunogenicity, efficacy, and safety of TB/Malaria vaccine candidate |
|  | Turner et al., | 2021 | Dendritic cell responses to Plasmodium falciparum in a malaria-endemic setting | Not related to immunogenicity, efficacy, and safety of TB/Malaria vaccine candidate |
|  | Sarazaz-Austin et al., | 2020 | Isoniazid Preventive Therapy and Pregnancy Outcomes in Women Living With  Human Immunodeficiency Virus in the Tshepiso Cohort | Not related to immunogenicity, efficacy, and safety of TB/Malaria vaccine candidate |
|  | He | 2014 | Development of a colloidal gold-based lateral flow dipstick immunoassay for rapid qualitative and semi-quantitative analysis of artesunate and dihydroartemisinin | Not related to immunogenicity, efficacy, and safety of TB/Malaria vaccine candidate |
|  | Arama et al., | 2015 | Genetic Resistance to Malaria Is Associated with Greater Enhancement of Immunoglobulin (Ig)M Than IgG Responses to a Broad Array of Plasmodium  falciparum Antigens | Not related to immunogenicity, efficacy, and safety of TB/Malaria vaccine candidate |
|  | Goncalves et al., | 2016 | Sickle haemoglobin, haemoglobin C and malaria mortality feedback | Not related to immunogenicity, efficacy, and safety of TB/Malaria vaccine candidate |
|  | Mcllleron et al., | 2012 | Reduced Antituberculosis Drug Concentrations in HIV-Infected Patients Who Are Men or Have Low Weight: Implications for International Dosing Guidelines | Not related to immunogenicity, efficacy, and safety of TB/Malaria vaccine candidate |
|  | Ayele et al., | 2012 | Isoniazid Prophylactic Therapy for the Prevention of Tuberculosis in HIV Infected Adults: A Systematic Review and Meta-Analysis of Randomized Trials | Not related to immunogenicity, efficacy, and safety of TB/Malaria vaccine candidate |
|  | Gupta et al., | 2020 | Feasibility of Identifying Household Contacts of Rifampin-and Multidrug-resistant Tuberculosis Cases at High Risk of Progression to Tuberculosis Disease | Not related to immunogenicity, efficacy, and safety of TB/Malaria vaccine candidate |
|  | Smithson et al., | 2015 | Impact of Malaria Control on Mortality and Anemia among Tanzanian Children Less than Five Years of Age, 1999-2010 | Not related to immunogenicity, efficacy, and safety of TB/Malaria vaccine candidate |
|  | Pwalia et al., | 2019 | High insecticide resistance intensity of Anopheles gambiae (s.l.) and low efficacy of pyrethroid LLINs in Accra, Ghana | Not related to immunogenicity, efficacy, and safety of TB/Malaria vaccine candidate |
|  | Jansensen et al., | 2017 | Hemostatic Changes Associated with Increased Mortality Rates in Hospitalized Patients With HIV-Associated Tuberculosis: Prospective Cohort Study | Not related to immunogenicity, efficacy, and safety of TB/Malaria vaccine candidate |
|  | Roordink et al., | 2021 | The TB vaccine development pathway - An innovative approach to accelerating global TB vaccine development | Not related to immunogenicity, efficacy, and safety of TB/Malaria vaccine candidate |
|  | Stepniewska et al., | 2022 | Efficacy of Single-Dose Primaquine with Artemisinin Combination Therapy on Plasmodium falciparum Gametocytes and Transmission: An Individual Patient Meta-Analysis | Not related to immunogenicity, efficacy, and safety of TB/Malaria vaccine candidate |
|  | Aleke et al., | 2022 | Cluster-randomized controlled trial of intermittent preventive treatment in infancy using sulfadoxine-pyrimethamine (SP-IPTi): a pilot study in Nigeria | Not related to immunogenicity, efficacy, and safety of TB/Malaria vaccine candidate |
|  | Moore et al., | 2020 | Immunoglobulin profile and B-cell frequencies are altered with changes in the cellular microenvironment independent of the stimulation conditions | Not related to immunogenicity, efficacy, and safety of TB/Malaria vaccine candidate |
|  | Elbadry et al., | 2017 | Submicroscopic malaria infections in pregnant women from six departments in Haiti | Not related to immunogenicity, efficacy, and safety of TB/Malaria vaccine candidate |
|  | Moguche et al., | 2017 | Antigen Availability Shapes T Cell Differentiation and Function during Tuberculosis | Not related to immunogenicity, efficacy, and safety of TB/Malaria vaccine candidate |
|  | Ngufor et al., | 2022 | Comparative efficacy of two pyrethroid-piperonyl butoxide nets (Olyset Plus, and PermaNet 3.0) against pyrethroid resistant malaria vectors: a non-inferiority assessment | Not related to immunogenicity, efficacy, and safety of TB/Malaria vaccine candidate |
|  | Timire et al., | 2021 | Catastrophic costs among tuberculosis-affected households in Zimbabwe: a national health facility-based survey | Not related to immunogenicity, efficacy, and safety of TB/Malaria vaccine candidate |
|  | Deshpande et al., | 2019 | Minocycline Immunomodulates via Sonic Hedgehog Signaling and Apoptosis  and Has Direct Potency Against Drug-Resistant Tuberculosis | Not related to immunogenicity, efficacy, and safety of TB/Malaria vaccine candidate |
|  | Oduro et al., | 2013 | Seroepidemiological and parasitological evaluation of the heterogeneity of malaria infection in the Gambia | Not related to immunogenicity, efficacy, and safety of TB/Malaria vaccine candidate |
|  | Akech et al., | 2020 | The Clinical Profile of Severe Pediatric Malaria in an Area Targeted for Routine RTS, S/AS01 Malaria Vaccination in Western Kenya | Not related to immunogenicity, efficacy, and safety of TB/Malaria vaccine candidate |
|  | Ali et al., | 2014 | Host candidate gene polymorphisms and associated clearance of P. falciparum amodiaquine and fansidar resistance mutants in children less than 5 years in Cameroon | Not related to immunogenicity, efficacy, and safety of TB/Malaria vaccine candidate |
|  | Panodou et al., | 2012 | Decreased proportions of indoor feeding and endophily in Anopheles gambiae s.l. populations following the indoor residual spraying and insecticide-treated net interventions in Benin (West Africa) | Not related to immunogenicity, efficacy, and safety of TB/Malaria vaccine candidate |
|  | Fowler et al., | 2022 | Epidemiological cut-off values for a 96-well broth microdilution plate for high-throughput research antibiotic susceptibility testing of M. tuberculosis | Not related to immunogenicity, efficacy, and safety of TB/Malaria vaccine candidate |
|  | Maiswelo et al., | 2017 | Sustained High Cure Rate of Artemether-Lumefantrine against Uncomplicated Plasmodium falciparum Malaria after 8 Years of Its Wide-Scale Use in Bagamoyo District, Tanzania | Not related to immunogenicity, efficacy, and safety of TB/Malaria vaccine candidate |
|  | Tweed et al., | 2019 | Bedaquiline, moxifloxacin, pretomanid, and pyrazinamide during the first 8 weeks of treatment of patients with drug-susceptible or drug-resistant pulmonary tuberculosis: a multicentre, open-label, partially randomised, phase 2b trial | Not related to immunogenicity, efficacy, and safety of TB/Malaria vaccine candidate |
|  | Mbuba et al., | 2021 | Single blinded semi-field evaluation of MAiA((R)) topical repellent ointment compared to unformulated 20% DEET against Anopheles gambiae,Anopheles arabiensis and Aedes aegypti in Tanzaniam | Not related to immunogenicity, efficacy, and safety of TB/Malaria vaccine candidate |
|  | Okumu et al., | 2013 | Comparative field evaluation of combinations of long-lasting insecticide treated nets and indoor residual spraying, relative to either method alone, for malaria prevention in an area where the main vector is Anopheles arabiensis | Not related to immunogenicity, efficacy, and safety of TB/Malaria vaccine candidate |
|  | Guiguemde et al., | 2013 | Treatment of Murine Cerebral Malaria by Artemisone in Combination with Conventional Antimalarial Drugs: Antiplasmodial Effects and Immune Responses | Not related to immunogenicity, efficacy, and safety of TB/Malaria vaccine candidate |
|  | Albrecht et al., | 2020 | Rosettes integrity protects Plasmodium vivax of being phagocytized | Not related to immunogenicity, efficacy, and safety of TB/Malaria vaccine candidate |
|  | Garcia-prats et al., | 2019 | Pharmacokinetics, Safety, and Dosing of Novel Pediatric Levofloxacin Dispersible Tablets in Children with Multidrug-Resistant Tuberculosis Exposure | Not related to immunogenicity, efficacy, and safety of TB/Malaria vaccine candidate |
|  | Valea et al., | 2012 | An analysis of timing and frequency of malaria infection during pregnancy in relation to the risk of low birth weight, anaemia, and perinatal mortality in Burkina Faso | Not related to immunogenicity, efficacy, and safety of TB/Malaria vaccine candidate |
|  | Agamah et al., | 2021 | Network-driven analysis of human-Plasmodium falciparum interactome: processes for malaria drug discovery and extracting in silico targets | Not related to immunogenicity, efficacy, and safety of TB/Malaria vaccine candidate |
|  | Menon et al., | 2016 | Cost and Predictors of Care-Seeking Behaviors among Caregivers of Febrile Children-Uganda, 2009 | Not related to immunogenicity, efficacy, and safety of TB/Malaria vaccine candidate |
|  | Albuquerque, et al., | 2014 | Infection control in households of drug-resistant tuberculosis patients co-infected with HIV in Mumbai, India | Not related to immunogenicity, efficacy, and safety of TB/Malaria vaccine candidate |
|  | Jonhson et al | 2014 | Effect of Isoniazid Therapy for Latent TB Infection on QuantiFERON-TB Gold In-Tube Responses in Adults with Positive Tuberculin Skin Test Results in a High TB Incidence Area | Not related to immunogenicity, efficacy, and safety of TB/Malaria vaccine candidate |
|  | De plessis et al., | 2016 | Phenotypic analysis of peripheral B cell populations during Mycobacterium tuberculosis infection and disease | Not related to immunogenicity, efficacy, and safety of TB/Malaria vaccine candidate |
|  | Mathad et al., | 2022 | Pharmacokinetics and Safety of 3 Months of Weekly Rifapentine and Isoniazid for Tuberculosis Prevention in Pregnant Women | Not related to immunogenicity, efficacy, and safety of TB/Malaria vaccine candidate |
|  | Lai et al., | 2019 | Changing epidemiology and challenges of malaria in China towards elimination | Not related to immunogenicity, efficacy, and safety of TB/Malaria vaccine candidate |
|  | Kurbatova et al., | 2015 | Sputum culture conversion as a prognostic marker for end-of-treatment outcome in patients with multidrug-resistant tuberculosis: a secondary analysis of data from two observational cohort studies | Not related to immunogenicity, efficacy, and safety of TB/Malaria vaccine candidate |
|  | Dechavanne et al., | 2016 | Acquisition of natural humoral immunity to P. falciparum in early life in Benin: impact of clinical, environmental and host factors | Not related to immunogenicity, efficacy, and safety of TB/Malaria vaccine candidate |
|  | Hobbs et al., | 2017 | Trimethoprim-Sulfamethoxazole Prophylaxis During Live Malaria Sporozoite  Immunization Induces Long-Lived, Homologous, and Heterologous Protective  Immunity Against Sporozoite Challenge | Not related to immunogenicity, efficacy, and safety of TB/Malaria vaccine candidate |
|  | Beng et al., | 2015 | Bioefficacy of a long-lasting insecticide impregnated net against Anopheles maculatus theobald and culex quinquefasciatus say | Not related to immunogenicity, efficacy, and safety of TB/Malaria vaccine candidate |
|  | Nuwa et al. | 2023 | A non-randomized controlled trial to assess the protective effect of SMC in the context of high parasite resistance in Uganda | Not related to immunogenicity, efficacy, and safety of TB/Malaria vaccine candidate |
|  | Orlov et al., | 2012 | P. falciparum Enhances HIV Replication in an Experimental Malaria Challenge System | Not related to immunogenicity, efficacy, and safety of TB/Malaria vaccine candidate |
|  | Knight et al., | 2019 | Cost of managing severe cutaneous adverse drug reactions to first line tuberculosis therapy in South Africa | Not related to immunogenicity, efficacy, and safety of TB/Malaria vaccine candidate |
|  | Musiba et al., | 2022 | Outdoor biting and pyrethroid resistance as potential drivers of persistent malaria transmission in Zanzibar | Not related to immunogenicity, efficacy, and safety of TB/Malaria vaccine candidate |
|  | Fletcher et al., | 2016 | T-cell activation is an immune correlate of risk in BCG vaccinated infants | Case study |
|  | Dheda et al., | 2019 | Spatial Network Mapping of Pulmonary Multidrug-Resistant Tuberculosis Cavities Using RNA Sequencing | Not related to immunogenicity, efficacy, and safety of TB/Malaria vaccine candidate |
|  | Martins et al., | 2020 | Malaria Risk and Prevention in Asian Migrants to Angola | Not related to immunogenicity, efficacy, and safety of TB/Malaria vaccine candidate |
|  | Benzekri et al., | 2019 | Nutrition support for HIV-TB co-infected adults in Senegal, West Africa: A randomized pilot implementation study | Not related to immunogenicity, efficacy, and safety of TB/Malaria vaccine candidate |
|  | Sangoro et al., | 2014 | A cluster-randomized controlled trial to assess the effectiveness of using 15% DEET topical repellent with long-lasting insecticidal nets (LLINs) compared to a placebo lotion on malaria transmission | Not related to immunogenicity, efficacy, and safety of TB/Malaria vaccine candidate |
|  | Guelbeogo et al., | 2014 | Behavioural divergence of sympatric Anopheles funestus populations in Burkina Faso | Not related to immunogenicity, efficacy, and safety of TB/Malaria vaccine candidate |
|  | Finda et al., | 2020 | Opinions of key stakeholders on alternative interventions for malaria control and elimination in Tanzania | Not related to immunogenicity, efficacy, and safety of TB/Malaria vaccine candidate |
|  | Santema et al., | 2012 | Early infection dynamics after experimental challenge with Mycobacterium avium subspecies paratuberculosis in calves reveal limited calf-to-calf transmission and no impact of Hsp70 vaccination | Pre-clinical study |
|  | Guo et al., | 2017 | Quality Testing of Artemisinin-Based Antimalarial Drugs in Myanmar | Not related to immunogenicity, efficacy, and safety of TB/Malaria vaccine candidate |
|  | Converse et al., | 2015 | Efficacy of Rifampin Plus Clofazimine in a Murine Model of Mycobacterium ulcerans Disease | Not related to immunogenicity, efficacy, and safety of TB/Malaria vaccine candidate |
|  | Wang et al., | 2014 | Role of casual contacts in the recent transmission of tuberculosis in settings with high disease burden | Not related to immunogenicity, efficacy, and safety of TB/Malaria vaccine candidate |
|  | Waheed et al., | 2017 | Infection control in hospitals managing drug-resistant tuberculosis in Pakistan: how are we doing? | Not related to immunogenicity, efficacy, and safety of TB/Malaria vaccine candidate |
|  | Moh | 2017 | Screening for active tuberculosis before isoniazid preventive therapy among HIV-infected West African adults | Not related to immunogenicity, efficacy, and safety of TB/Malaria vaccine candidate |
|  | Teinhardt et al., | 2014 | Quality of Malaria Case Management in Malawi: Results from a Nationally Representative Health Facility Survey | Not related to immunogenicity, efficacy, and safety of TB/Malaria vaccine candidate |
|  | Borisov et al., | 2017 | Effectiveness and safety of bedaquiline-containing regimens in the treatment of MDR- and XDR-TB: a multicentre study | Not related to immunogenicity, efficacy, and safety of TB/Malaria vaccine candidate |
|  | Maas et al., | 2012 | Development of a lion-specific interferon-gamma assay | Not related to immunogenicity, efficacy, and safety of TB/Malaria vaccine candidate |
|  | Hart et al., | 2016 | The Regulation of Inherently Autoreactive VH4-34-Expressing B Cells in Individuals Living in a Malaria-Endemic Area of West Africa | Not related to immunogenicity, efficacy, and safety of TB/Malaria vaccine candidate |
|  | Tan et al., | 2020 | Barriers and facilitators of tuberculosis infection prevention and control in low- and middle-income countries from the perspective of healthcare workers: A systematic review | Not related to immunogenicity, efficacy, and safety of TB/Malaria vaccine candidate |
|  | Kynast-Wolf et al., | 2012 | ITN protection, MSP1 antibody levels and malaria episodes in young children of rural Burkina Faso | Not related to immunogenicity, efficacy, and safety of TB/Malaria vaccine candidate |
|  | Apinjol et al., | 2014 | Association of candidate gene polymorphisms and TGF-beta/IL-10 levels with malaria in three regions of Cameroon: a case-control study | Not related to immunogenicity, efficacy, and safety of TB/Malaria vaccine candidate |
|  | Haenigger et al., | 2020 | Sexual communication of Spodoptera frugiperda from West Africa: Adaptation of an invasive species and implications for pest management | Not related to immunogenicity, efficacy, and safety of TB/Malaria vaccine candidate |
|  | Lopez-perez et al., | 2021 | PfEMP1-Specific Immunoglobulin G Reactivity Among Beninese Pregnant Women with Sickle Cell Trait | Not related to immunogenicity, efficacy, and safety of TB/Malaria vaccine candidate |
|  | Ranaivomanana et al., | 2021 | Responses in HIV-Negative Pregnant Women Exposed to Tuberculosis | Not related to immunogenicity, efficacy, and safety of TB/Malaria vaccine candidate |
|  | Mejia et al., | 2013 | Physical condition of Olyset (R) nets after five years of utilization in rural western Kenya | Not related to immunogenicity, efficacy, and safety of TB/Malaria vaccine candidate |
|  | Messenger et al., | 2014 | Installation of insecticide-treated durable wall lining: evaluation of attachment materials and product durability under field conditions | Not related to immunogenicity, efficacy, and safety of TB/Malaria vaccine candidate |
|  | N’Guessan et al., | 2014 | Mosquito Nets Treated with a Mixture of Chlorfenapyr and Alphacypermethrin Control Pyrethroid Resistant Anopheles gambiae and Culex quinquefasciatus Mosquitoes in West Africa | Not related to immunogenicity, efficacy, and safety of TB/Malaria vaccine candidate |
|  | Botwe et al., | 2021 | Determinants of the varied profiles of Plasmodium falciparum infections among infants living in Kintampo, Ghana | Not related to immunogenicity, efficacy, and safety of TB/Malaria vaccine candidate |
|  | Parsons et al., | 2012 | Development of a diagnostic gene expression assay for tuberculosis and its use under field conditions in African buffaloes (Syncerus caffer) | Not related to immunogenicity, efficacy, and safety of TB/Malaria vaccine candidate |
|  | Li et al., | 2012 | Identification of a specific region of Plasmodium falciparum EBL-1 that binds to host receptor glycophorin B and inhibits merozoite invasion in human red blood cells | Not related to immunogenicity, efficacy, and safety of TB/Malaria vaccine candidate |
|  | Idoko et al., | 2014 | The impact of pre-existing antibody on subsequent immune responses to meningococcal A-containing vaccines | Non-TB/malaria study |
|  | Rustomjee et al., | 2012 | Key issues in the clinical development and implementation of TB vaccines in South Africa | Not related to immunogenicity, efficacy, and safety of TB/Malaria vaccine candidate |
|  | Bojang et al., | 2001 | Efficacy of RTS, S/AS02 malaria vaccine against Plasmodium falciparum infection in semi-immune adult men in The Gambia: a randomised trial | Publication year |
|  | Pichyangkul et al., | 2004 | Pre-clinical evaluation of the malaria vaccine candidate P. falciparum MSP1(42) formulated with novel adjuvants or with alum | Pre-clinical study, publication year |
|  | Reece et al., | 2004 | A CD4(+) T-cell immune response to a conserved epitope in the circumsporozoite protein correlates with protection from natural Plasmodium falciparum infection and disease | Not related to immunogenicity, efficacy, and safety of TB/Malaria vaccine candidate |
|  | Webster et al., | 2005 | Enhanced T cell-mediated protection against malaria in human challenges by using the recombinant poxviruses FP9 and modified vaccinia virus Ankara | Not related to immunogenicity, efficacy, and safety of TB/Malaria vaccine candidate |
|  | Vardas et al., | 2012 | Indicators of therapeutic effect in FIT-06, a Phase II trial of a DNA vaccine, GTU®-multi-HIVB, in untreated HIV-1 infected subjects | Not related to immunogenicity, efficacy, and safety of TB/Malaria vaccine candidate |
|  | Kassa et al., | 2012 | Incidence of tuberculosis and early mortality in a large cohort of HIV infected patients receiving antiretroviral therapy in a tertiary hospital in Addis Ababa, Ethiopia | Not related to immunogenicity, efficacy, and safety of TB/Malaria vaccine candidate |
|  | Mitchell et al., | 2012 | Identification and validation of a gene causing cross-resistance between insecticide classes in Anopheles gambiae from Ghana | Not related to immunogenicity, efficacy, and safety of TB/Malaria vaccine candidate |
|  | Pensi et al., | 2012 | Simultaneous HAART improves survival in children coinfected with HIV and  TB | Not related to immunogenicity, efficacy, and safety of TB/Malaria vaccine candidate |
|  | Davies et al., | 2012 | Designing Adverse Event Forms for Real-World Reporting: Participatory Research in Uganda | Not related to immunogenicity, efficacy, and safety of TB/Malaria vaccine candidate |
|  | Zeeshan et al., | 2012 | Genetic Variation in the Plasmodium falciparum Circumsporozoite Protein in India and Its Relevance to RTS, S Malaria Vaccine | Not related to immunogenicity, efficacy, and safety of TB/Malaria vaccine candidate |
|  | Moiroux et al., | 2012 | Changes in Anopheles funestus Biting Behavior Following Universal Coverage of Long-Lasting Insecticidal Nets in Benin | Not related to immunogenicity, efficacy, and safety of TB/Malaria vaccine candidate |
|  | Vordermeier et al., | 2012 | Conserved Immune Recognition Hierarchy of Mycobacterial PE/PPE Proteins during Infection in Natural Hosts | Not related to immunogenicity, efficacy, and safety of TB/Malaria vaccine candidate |
|  | Luabaye et al., | 2012 | Diagnostic Features Associated with Culture of Mycobacterium tuberculosis Among Young Children in a Vaccine Trial Setting | Not related to immunogenicity, efficacy, and safety of TB/Malaria vaccine candidate |
|  | Diacon et al., | 2012 | Randomized Pilot Trial of Eight Weeks of Bedaquiline (TMC207) Treatment for Multidrug-Resistant Tuberculosis: Long-Term Outcome, Tolerability, and Effect on Emergence of Drug Resistance | Not related to immunogenicity, efficacy, and safety of TB/Malaria vaccine candidate |
|  | Kitau et al., | 2012 | Species Shifts in the Anopheles gambiae Complex: Do LLINs Successfully Control Anopheles arabiensis? | Not related to immunogenicity, efficacy, and safety of TB/Malaria vaccine candidate |
|  | Riou et al., | 2012 | Effect of Standard Tuberculosis Treatment on Plasma Cytokine Levels in Patients with Active Pulmonary Tuberculosis | Not related to immunogenicity, efficacy, and safety of TB/Malaria vaccine candidate |
|  | Farley et al., | 2012 | A national infection control evaluation of drug-resistant tuberculosis hospitals in South Africa | Not related to immunogenicity, efficacy, and safety of TB/Malaria vaccine candidate |
|  | Hanifa et al., | 2012 | Tuberculosis among adults starting antiretroviral therapy in South Africa: the need for routine case finding | Not related to immunogenicity, efficacy, and safety of TB/Malaria vaccine candidate |
|  | Naidoo et al., | 2012 | Tuberculosis infection control in primary health clinics in eThekwini, KwaZulu-Natal, South Africa | Not related to immunogenicity, efficacy, and safety of TB/Malaria vaccine candidate |
|  | Messenger et al., | 2012 | Multicentre studies of insecticide-treated durable wall lining in Africa and South-East Asia: entomological efficacy and household acceptability during one year of field use | Not related to immunogenicity, efficacy, and safety of TB/Malaria vaccine candidate |
|  | Chapman et al., | 2012 | Priming with a Recombinant Pantothenate Auxotroph of Mycobacterium bovis  BCG and Boosting with MVA Elicits HIV-1 Gag Specific CD8(+) T Cells | Not related to immunogenicity, efficacy, and safety of TB/Malaria vaccine candidate |
|  | Thind et al., | 2012 | An evaluation of `Ribolola': a household tuberculosis contact tracing programme in Northwest Province, South Africa | Not related to immunogenicity, efficacy, and safety of TB/Malaria vaccine candidate |
|  | Loxton et al., | 2012 | Heparin-Binding Hemagglutinin Induces IFN-gamma (+) IL-2(+) IL-17(+) Multifunctional CD4(+) T Cells during Latent but Not Active Tuberculosis Disease | Not related to immunogenicity, efficacy, and safety of TB/Malaria vaccine candidate |
|  | Froberg et al., | 2012 | Decreased prevalence of Plasmodium falciparum resistance markers to amodiaquine despite its wide scale use as ACT partner drug in Zanzibar | Non-vaccination |
|  | macMullin et al., | 2012 | Host immune response in returning travellers infected with malaria | Not related to immunogenicity, efficacy, and safety of TB/Malaria vaccine candidate |
|  | Karema et al., | 2012 | Trends in malaria cases, hospital admissions and deaths following scale-up of anti-malarial interventions, 2000-2010, Rwanda | Not related to immunogenicity, efficacy, and safety of TB/Malaria vaccine candidate |
|  | Zwang et al., | 2012 | Comparing changes in haematologic parameters occurring in patients included in randomized controlled trials of artesunate-amodiaquine vs single and combination treatments of uncomplicated falciparum in sub-Saharan Africa | Not related to immunogenicity, efficacy, and safety of TB/Malaria vaccine candidate |
|  | Likwela et al., | 2012 | Sulfadoxine-pyrimethamine resistance and intermittent preventive treatment during pregnancy: a retrospective analysis of birth weight data in the Democratic Republic of Congo (DRC) | Not related to immunogenicity, efficacy, and safety of TB/Malaria vaccine candidate |
|  | khatib et al., | 2012 | Routine delivery of artemisinin-based combination treatment at fixed health facilities reduces malaria prevalence in Tanzania: an observational study | Not related to immunogenicity, efficacy, and safety of TB/Malaria vaccine candidate |
|  | Adu et al., | 2012 | Fc Gamma Receptor IIIB (Fc gamma RIIIB) Polymorphisms Are Associated with Clinical Malaria in Ghanaian Children | Not related to immunogenicity, efficacy, and safety of TB/Malaria vaccine candidate |
|  | Padayatchi et al., | 2012 | Lessons from a randomised clinical trial for multidrug-resistant tuberculosis | Not related to immunogenicity, efficacy, and safety of TB/Malaria vaccine candidate |
|  | Doumbia et al., | 2012 | Improving malaria control in West Africa: Interruption of transmission as a paradigm shift | Not related to immunogenicity, efficacy, and safety of TB/Malaria vaccine candidate |
|  | Hermans et al., | 2012 | Unrecognised tuberculosis at antiretroviral therapy initiation is associated with lower CD4+T cell recovery | Not related to immunogenicity, efficacy, and safety of TB/Malaria vaccine candidate |
|  | Emeth et al., | 2012 | Peripheral T Cell Cytokine Responses for Diagnosis of Active Tuberculosis | Not related to immunogenicity, efficacy, and safety of TB/Malaria vaccine candidate |
|  | Kerschberger et al., | 2012 | The Effect of Complete Integration of HIV and TB Services on Time to Initiation of Antiretroviral Therapy: A Before-After Study | Not related to immunogenicity, efficacy, and safety of TB/Malaria vaccine candidate |
|  | Ritz et al., | 2012 | A Comparative Analysis of Polyfunctional T Cells and Secreted Cytokines Induced by Bacille Calmette-Guerin Immunisation in Children and Adults | Non-sub-Saharan African study |
|  | Peter et al., | 2012 | Diagnostic accuracy of a urine lipoarabinomannan strip-test for TB detection in HIV-infected hospitalised patients | Not related to immunogenicity, efficacy, and safety of TB/Malaria vaccine candidate |
|  | Badolo et al., | 2012 | Development of an allele-specific, loop-mediated, isothermal amplification method (AS-LAMP) to detect the L1014F kdr-w mutation in Anopheles gambiae s. l. | Not related to immunogenicity, efficacy, and safety of TB/Malaria vaccine candidate |
|  | Yadava et al., | 2012 | Cross-Species Immunity Following Immunization with a Circumsporozoite Protein-Based Vaccine for Malaria | Pre-clinical study |
|  | Zwang et al., | 2012 | Clinical tolerability of artesunate-amodiaquine versus comparator treatments for uncomplicated falciparum malaria: an individual patient analysis of eight randomized controlled trials in sub-Saharan Africa | Not related to immunogenicity, efficacy, and safety of TB/Malaria vaccine candidate |
|  | Wurt et al., | 2012 | Early treatment failure during treatment of Plasmodium falciparum malaria with atovaquone-proguanil in the Republic of Côte d’Ivoire | Not related to immunogenicity, efficacy, and safety of TB/Malaria vaccine candidate |
|  | Maharaj et al., | 2012 | Screening of selected ethnomedicinal plants from South Africa for larvicidal activity against the mosquito Anopheles arabiensis | Not related to immunogenicity, efficacy, and safety of TB/Malaria vaccine candidate |
|  | Hom et al., | 2012 | Drug-Resistant Tuberculosis among HIV-Infected Patients Starting Antiretroviral Therapy in Durban, South Africa | Not related to immunogenicity, efficacy, and safety of TB/Malaria vaccine candidate |
|  | Vaughan-Williams, et al., | 2012 | Assessment of the therapeutic efficacy of artemether-lumefantrine in the treatment of uncomplicated Plasmodium falciparum malaria in northern KwaZulu-Natal: an observational cohort study | Not related to immunogenicity, efficacy, and safety of TB/Malaria vaccine candidate |
|  | Choveaux et al., | 2012 | A Plasmodium falciparum copper-binding membrane protein with copper transport motifs | Not related to immunogenicity, efficacy, and safety of TB/Malaria vaccine candidate |
|  | Fancony et al | 2012 | Various pfcrt and pfmdr1 Genotypes of Plasmodium falciparum Cocirculate with P. malariae, P. ovale spp., and P. vivax in Northern Angola | Non vaccination study |
|  | Korenromp | 2012 | Lives saved from malaria prevention in Africa-evidence to sustain cost-effective gains | Not related to immunogenicity, efficacy, and safety of TB/Malaria vaccine candidate |
|  | Shenoi et al., | 2012 | Survival from XDR-TB Is Associated with Modifiable Clinical Characteristics in Rural South Africa | Not related to immunogenicity, efficacy, and safety of TB/Malaria vaccine candidate |
|  | Crump et al., | 2012 | Bacteremic Disseminated Tuberculosis in Sub-Saharan Africa: A Prospective Cohort Study | Not related to immunogenicity, efficacy, and safety of TB/Malaria vaccine candidate |
|  | Jagannathan et al., | 2012 | Increasing incidence of malaria in children despite insecticide-treated bed nets and prompt anti-malarial therapy in Tororo, Uganda | Not related to immunogenicity, efficacy, and safety of TB/Malaria vaccine candidate |
|  | Portugal et al., | 2012 | B cell analysis of ethnic groups in Mali with differential susceptibility to malaria | Not related to immunogenicity, efficacy, and safety of TB/Malaria vaccine candidate |
|  | Brust et al., | 2012 | Integrated, home-based treatment for MDR-TB and HIV in rural South Africa: an alternate model of care | Not related to immunogenicity, efficacy, and safety of TB/Malaria vaccine candidate |
|  | Timmann et al., | 2012 | Genome-wide association study indicates two novel resistance loci for severe malaria | Not related to immunogenicity, efficacy, and safety of TB/Malaria vaccine candidate |
|  | Ablordey et al., | 2012 | Detection of Mycobacterium ulcerans by the Loop Mediated Isothermal Amplification Method | Not related to immunogenicity, efficacy, and safety of TB/Malaria vaccine candidate |
|  | Janson et al., | 2012 | Costs and process of in-patient tuberculosis management at a central academic hospital, Cape Town, South Africa | Not related to immunogenicity, efficacy, and safety of TB/Malaria vaccine candidate |
|  | Badolo et al., | 2012 | Experimental hut evaluation of Fendona 6SC (R)-treated bednets and Interceptor (R) long-lasting nets against Anopheles gambiae s.l. in Burkina Faso | Not related to immunogenicity, efficacy, and safety of TB/Malaria vaccine candidate |
|  | Sarkar et al., | 2012 | Modern Lineages of Mycobacterium tuberculosis Exhibit Lineage-Specific Patterns of Growth and Cytokine Induction in Human Monocyte-Derived Macrophages | Not related to immunogenicity, efficacy, and safety of TB/Malaria vaccine candidate |
|  | Komazawa et al., | 2012 | Are Long-Lasting Insecticidal Nets Effective for Preventing Childhood Deaths among Non-Net Users? A Community-Based Cohort Study in Western Kenya | Not related to immunogenicity, efficacy, and safety of TB/Malaria vaccine candidate |
|  | Krause et al., | 2012 | alpha-Thalassemia Impairs the Cytoadherence of Plasmodium falciparum- Infected Erythrocytes | Not related to immunogenicity, efficacy, and safety of TB/Malaria vaccine candidate |
|  | Andreani et al., | 2012 | An In vitro Co-infection Model to Study Plasmodium falciparum-HIV-1 Interactions in Human Primary Monocyte-derived Immune Cells | Not related to immunogenicity, efficacy, and safety of TB/Malaria vaccine candidate |
|  | Shipro et al., | 2012 | Community-based Targeted Case Finding for Tuberculosis and HIV in Household Contacts of Patients with Tuberculosis in South Africa | Not related to immunogenicity, efficacy, and safety of TB/Malaria vaccine candidate |
|  | Maia et al., | 2012 | The Effect of Deltamethrin-treated Net Fencing around Cattle Enclosures on Outdoor-biting Mosquitoes in Kumasi, Ghana | Not related to immunogenicity, efficacy, and safety of TB/Malaria vaccine candidate |
|  | Bliven-Sizemore et al., | 2012 | Effect of HIV infection on tolerability and bacteriologic outcomes of tuberculosis treatment | Not related to immunogenicity, efficacy, and safety of TB/Malaria vaccine candidate |
|  | Oni et al., | 2012 | Smoking, BCG and Employment and the Risk of Tuberculosis Infection in HIV-Infected Persons in South Africa | Not related to immunogenicity, efficacy, and safety of TB/Malaria vaccine candidate |
|  | Ambele et al., | 2012 | Neutral lipids associated with haemozoin mediate efficient and rapid beta-haematin formation at physiological pH, temperature, and ionic composition | Not related to immunogenicity, efficacy, and safety of TB/Malaria vaccine candidate |
|  | Faye et al., | 2012 | Multicentre study evaluating the non-inferiority of the new paediatric formulation of artesunate/amodiaquine versus artemether/lumefantrine for the management of uncomplicated Plasmodium falciparum malaria in children in Cameroon, Ivory Coast and Senegal | Not related to immunogenicity, efficacy, and safety of TB/Malaria vaccine candidate |
|  | Gupta et al., | 2012 | Tuberculosis Incidence Rates during 8 Years of Follow-Up of an Antiretroviral Treatment Cohort in South Africa: Comparison with Rates in the Community | Not related to immunogenicity, efficacy, and safety of TB/Malaria vaccine candidate |
|  | Kleinnijenhuis et al., | 2012 | Bacille Calmette-Guerin induces NOD2-dependent nonspecific protection from reinfection via epigenetic reprogramming of monocytes | Not related to immunogenicity, efficacy, and safety of TB/Malaria vaccine candidate |
|  | Cottrell et al., | 2012 | Modeling the Influence of Local Environmental Factors on Malaria Transmission in Benin and Its Implications for Cohort Study | Not related to immunogenicity, efficacy, and safety of TB/Malaria vaccine candidate |
|  | Drame et al., | 2012 | IgG responses to the gSG6-P1 salivary peptide for evaluating human exposure to Anopheles bites in urban areas of Dakar region, Senegal | Not related to immunogenicity, efficacy, and safety of TB/Malaria vaccine candidate |
|  | Skolimowska et al., | 2012 | Altered Ratio of IFN-gamma/IL-10 in Patients with Drug Resistant Mycobacterium tuberculosis and HIV-Tuberculosis Immune Reconstitution Inflammatory Syndrome | Not related to immunogenicity, efficacy, and safety of TB/Malaria vaccine candidate |
|  | Briet et al. | 2012 | Importance of factors determining the effective lifetime of a mass, long-lasting, insecticidal net distribution: a sensitivity analysis | Not related to immunogenicity, efficacy, and safety of TB/Malaria vaccine candidate |
|  | Gandhi et al., | 2012 | Risk factors for mortality among MDR- and XDR-TB patients in a high HIV prevalence setting | Not related to immunogenicity, efficacy, and safety of TB/Malaria vaccine candidate |
|  | Temu et al., | 2012 | High Prevalence of Malaria in Zambezia, Mozambique: The Protective Effect of IRS versus Increased Risks Due to Pig-Keeping and House Construction | Not related to immunogenicity, efficacy, and safety of TB/Malaria vaccine candidate |
|  | Bingham et all., | 2012 | Community perceptions of malaria and vaccines in two districts of Mozambique | Not related to immunogenicity, efficacy, and safety of TB/Malaria vaccine candidate |
|  | Hsiang et al., | 2012 | Surveillance for Malaria Elimination in Swaziland: A National Cross-Sectional Study Using Pooled PCR and Serology | Not related to immunogenicity, efficacy, and safety of TB/Malaria vaccine candidate |
|  | Mesfin et al., | 2012 | Predictors of antiretroviral treatment-associated tuberculosis in Ethiopia: a nested case-control study | Not related to immunogenicity, efficacy, and safety of TB/Malaria vaccine candidate |
|  | Kaul et al., | 2012 | An Important Role of Prostanoid Receptor EP2 in Host Resistance to Mycobacterium tuberculosis Infection in Mice | Not related to immunogenicity, efficacy, and safety of TB/Malaria vaccine candidate |
|  | Dharmadhikari et al., | 2012 | Surgical Face Masks Worn by Patients with Multidrug-Resistant Tuberculosis Impact on Infectivity of Air on a Hospital Ward | Not related to immunogenicity, efficacy, and safety of TB/Malaria vaccine candidate |
|  | Okello et al., | 2012 | Local perceptions of intermittent screening and treatment for malaria in school children on the south coast of Kenya | Not related to immunogenicity, efficacy, and safety of TB/Malaria vaccine candidate |
|  | Lapadula et al., | 2012 | Unmasking tuberculosis in the era of antiretroviral treatment | Not related to immunogenicity, efficacy, and safety of TB/Malaria vaccine candidate |
|  | Seyoum et al., | 2012 | Human exposure to anopheline mosquitoes occurs primarily indoors, even for users of insecticide-treated nets in Luangwa Valley, South-east Zambia | Not related to immunogenicity, efficacy, and safety of TB/Malaria vaccine candidate |
|  | Dabire et al., | 2012 | Population dynamics of Anopheles gambiae s.l. in Bobo-Dioulasso city: bionomics, infection rate and susceptibility to insecticides | Not related to immunogenicity, efficacy, and safety of TB/Malaria vaccine candidate |
|  | Temu et al., | 2012 | Pyrethroid Resistance in Anopheles gambiae, in Bomi County, Liberia, Compromises Malaria Vector Control | Not related to immunogenicity, efficacy, and safety of TB/Malaria vaccine candidate |
|  | Sonnenberg et al., | 2012 | Quantifying errors in the estimation of tuberculosis mortality in a population of South African miners | Not related to immunogenicity, efficacy, and safety of TB/Malaria vaccine candidate |
|  | Brasseur et al., | 2012 | Anti-malarial drug safety information obtained through routine monitoring in a rural district of South-Western Senegal | Not related to immunogenicity, efficacy, and safety of TB/Malaria vaccine candidate |
|  | Louwagie et al., | 2012 | Missed opportunities for accessing HIV care among Tshwane tuberculosis patients under different models of care | Not related to immunogenicity, efficacy, and safety of TB/Malaria vaccine candidate |
|  | Hatherill et al., | 2012 | Consensus Statement on Diagnostic End Points for Infant Tuberculosis Vaccine Trials | Not related to immunogenicity, efficacy, and safety of TB/Malaria vaccine candidate |
|  | Geldenhuys et al., | 2012 | Safety and tolerability of sputum induction in adolescents and adults with suspected pulmonary tuberculosis | Not related to immunogenicity, efficacy, and safety of TB/Malaria vaccine candidate |
|  | Orlov et al., | 2012 | P. falciparum Enhances HIV Replication in an Experimental Malaria Challenge System | Not related to immunogenicity, efficacy, and safety of TB/Malaria vaccine candidate |
|  | Bogart et al., | 2013 | Let's Talk! A South African worksite-based HIV prevention parenting program | Not related to immunogenicity, efficacy, and safety of TB/Malaria vaccine candidate |
|  | Annane et al., | 2013 | Effects of fluid resuscitation with colloids vs crystalloids on mortality in critically ill patients presenting with hypovolemic shock - The CRISTAL randomized trial | Not related to immunogenicity, efficacy, and safety of TB/Malaria vaccine candidate |
|  | Pengpid et al., | 2013 | Screening and brief interventions for hazardous and harmful alcohol use among university students in South Africa: Results from a randomized controlled trial | Non- vaccination study |
|  | Kintu et al., | 2013 | Feasibility and safety of ALVAC-HIV vCP1521 vaccine in HIV-exposed infants in uganda: Results from the first HIV vaccine trial in infants in Africa | Non-TB/malaria study |
|  | Wingood et al., | 2013 | Efficacy of SISTA South Africa on sexual behavior and relationship control among isiXhosa women in South Africa: Results of a randomized-controlled trial | Not related to immunogenicity, efficacy, and safety of TB/Malaria vaccine candidate |
|  | Mehta et al., | 2013 | The long-term efficacy of medical male circumcision against HIV acquisition | Not related to immunogenicity, efficacy, and safety of TB/Malaria vaccine candidate |
|  | Paintain et al., | 2013 | Sustaining Fragile Gains: The Need to Maintain Coverage with Long-Lasting Insecticidal Nets for Malaria Control and Likely Implications of Not Doing So | Not related to immunogenicity, efficacy, and safety of TB/Malaria vaccine candidate |
|  | Sudfeld et al., | 2013 | Effect of multivitamin supplementation on measles vaccine response among HIV-exposed uninfected Tanzanian infants | Not related to immunogenicity, efficacy, and safety of TB/Malaria vaccine candidate |
|  | Dintwe et al., | 2013 | Heterologous vaccination against human tuberculosis modulates antigen-specific CD4(+) T-cell function | Not related to immunogenicity, efficacy, and safety of TB/Malaria vaccine candidate |
|  | Andries et al., | 2013 | High Rate of Hypothyroidism in Multidrug-Resistant Tuberculosis Patients Co-Infected with HIV in Mumbai, India | Not related to immunogenicity, efficacy, and safety of TB/Malaria vaccine candidate |
|  | Isaakidis et al., | 2013 | Poor Outcomes in a Cohort of HIV-Infected Adolescents Undergoing Treatment for Multidrug-Resistant Tuberculosis in Mumbai, India | Not related to immunogenicity, efficacy, and safety of TB/Malaria vaccine candidate |
|  | du Plessis et al., | 2013 | Increased Frequency of Myeloid-derived Suppressor Cells during Active Tuberculosis and after Recent Mycobacterium tuberculosis Infection Suppresses T-Cell Function | Not related to immunogenicity, efficacy, and safety of TB/Malaria vaccine candidate |
|  | Gupta et al., | 2013 | Acceleration of Tuberculosis Treatment by Adjunctive Therapy with Verapamil as an Efflux Inhibitor | Not related to immunogenicity, efficacy, and safety of TB/Malaria vaccine candidate |
|  | Laureillard et al., | 2013 | Paradoxical tuberculosis-associated immune reconstitution inflammatory syndrome after early initiation of antiretroviral therapy in a  randomized clinical trial | Not related to immunogenicity, efficacy, and safety of TB/Malaria vaccine candidate |
|  | Kløverpris et al., | 2013 | Non-Immunogenicity of Overlapping Gag Peptides Pulsed on Autologous Cells after Vaccination of HIV Infected Individuals | Not related to immunogenicity, efficacy, and safety of TB/Malaria vaccine candidate |
|  | Beksinska et al., | 2013 | Performance and safety of the second-generation female condom (FC2) versus the Woman's, the VA worn-of-women, and the Cupid female condoms: A randomised controlled non-inferiority crossover trial | Not related to immunogenicity, efficacy, and safety of TB/Malaria vaccine candidate |
|  | Agbor et al., | 2014 | Factors Associated with Death during Tuberculosis Treatment of Patients Co-Infected with HIV at the Yaounde Central Hospital, Cameroon: An 8-Year Hospital-Based Retrospective Cohort Study (2006-2013) | Not related to immunogenicity, efficacy, and safety of TB/Malaria vaccine candidate |
|  | Kanza et al., | 2013 | Pyrethroid, DDT and malathion resistance in the malaria vector Anopheles gambiae from the Democratic Republic of Congo | Not related to immunogenicity, efficacy, and safety of TB/Malaria vaccine candidate |
|  | Apinjohr et al., | 2013 | Association of Cytokine and Toll-Like Receptor Gene Polymorphisms with Severe Malaria in Three Regions of Cameroon | Not related to immunogenicity, efficacy, and safety of TB/Malaria vaccine candidate |
|  | Gehre et al., | 2013 | Immunogenic Mycobacterium africanum Strains Associated with Ongoing Transmission in The Gambia | Not related to immunogenicity, efficacy, and safety of TB/Malaria vaccine candidate |
|  | Allie et al., | 2013 | Prominent role for T cell-derived Tumour Necrosis Factor for sustained control of Mycobacterium tuberculosis infection | Not related to immunogenicity, efficacy, and safety of TB/Malaria vaccine candidate |
|  | Khatib et al., | 2013 | Access to artemisinin-based anti-malarial treatment and its related factors in rural Tanzania | Not related to immunogenicity, efficacy, and safety of TB/Malaria vaccine candidate |
|  | Tadokera et al., | 2013 | Role of the Interleukin 10 Family of Cytokines in Patients with Immune Reconstitution Inflammatory Syndrome Associated with HIV Infection and Tuberculosis | Not related to immunogenicity, efficacy, and safety of TB/Malaria vaccine candidate |
|  | Liheluka et al., | 2013 | Community perceptions on the secondary health benefits established by malaria vaccine trials (RTS, S phase 2 and phase 3) at the Korogwe site in Northeastern Tanzania | Not related to immunogenicity, efficacy, and safety of TB/Malaria vaccine candidate |
|  | Stockdale et al., | 2013 | Initiation of antiretroviral therapy in HIV-infected tuberculosis patients in rural Kenya: an observational study | Not related to immunogenicity, efficacy, and safety of TB/Malaria vaccine candidate |
|  | Fenner et al., | 2013 | Tuberculosis and the risk of opportunistic infections and cancers in HIV-infected patients starting ART in Southern Africa | Not related to immunogenicity, efficacy, and safety of TB/Malaria vaccine candidate |
|  | Rowland et al., | 2013 | A New Long-Lasting Indoor Residual Formulation of the Organophosphate Insecticide Pirimiphos Methyl for Prolonged Control of Pyrethroid-Resistant Mosquitoes: An Experimental Hut Trial in Benin | Not related to immunogenicity, efficacy, and safety of TB/Malaria vaccine candidate |
|  | Mpagama et al., | 2013 | Diagnosis and Interim Treatment Outcomes from the First Cohort of Multidrug-Resistant Tuberculosis Patients in Tanzania | Not related to immunogenicity, efficacy, and safety of TB/Malaria vaccine candidate |
|  | Flegg et al., | 2013 | Trends in Antimalarial Drug Use in Africa | Not related to immunogenicity, efficacy, and safety of TB/Malaria vaccine candidate |
|  | Lo et al., | 2013 | Prevalence of molecular markers of drug resistance in an area of seasonal malaria chemoprevention in children in Senegal | Not related to immunogenicity, efficacy, and safety of TB/Malaria vaccine candidate |
|  | Mangoka et al., | 2013 | Experience and challenges from clinical trials with malaria vaccines in Africa | Not related to immunogenicity, efficacy, and safety of TB/Malaria vaccine candidate |
|  | Ndounga et al., | 2013 | Artesunate-amodiaquine efficacy in Congolese children with acute uncomplicated falciparum malaria in Brazzaville | Not related to immunogenicity, efficacy, and safety of TB/Malaria vaccine candidate |
|  | Payne et al., | 2013 | Plasmodium index, prevention, and control of malaria in Dschang municipality, Cameroon | Not related to immunogenicity, efficacy, and safety of TB/Malaria vaccine candidate |
|  | Charle et al., | 2013 | Artesunate/Amodiaquine Malaria Treatment for Equatorial Guinea (Central Africa) | Not related to immunogenicity, efficacy, and safety of TB/Malaria vaccine candidate |
|  | Dugassa et al., | 2013 | Development of a Gravid Trap for Collecting Live Malaria Vectors Anopheles gambiae s.l. | Not related to immunogenicity, efficacy, and safety of TB/Malaria vaccine candidate |
|  | Gupta et al., | 2013 | Prevalent and Incident Tuberculosis Are Independent Risk Factors for Mortality among Patients Accessing Antiretroviral Therapy in South Africa | Not related to immunogenicity, efficacy, and safety of TB/Malaria vaccine candidate |
|  | Pascual et al., | 2013 | In vitro susceptibility to quinine and microsatellite variations of the Plasmodium falciparum Na+/H+ exchanger transporter (Pfnhe-1) gene in 393 isolates from Dakar, Senegal | Not related to immunogenicity, efficacy, and safety of TB/Malaria vaccine candidate |
|  | Bauman et al., | 2013 | Serodiagnostic markers for the prediction of the outcome of intensive phase tuberculosis therapy | Not related to immunogenicity, efficacy, and safety of TB/Malaria vaccine candidate |
|  | Tanabe et al., | 2013 | Within-population genetic diversity of Plasmodium falciparum vaccine candidate antigens reveals geographic distance from a Central sub-Saharan African origin | Not related to immunogenicity, efficacy, and safety of TB/Malaria vaccine candidate |
|  | Bratschi et al., | 2013 | Geographic Distribution, Age Pattern and Sites of Lesions in a Cohort of Buruli Ulcer Patients from the Mape Basin of Cameroon | Not related to immunogenicity, efficacy, and safety of TB/Malaria vaccine candidate |
|  | Maheu-Giroux et al., | 2013 | Impact of Community-Based Larviciding on the Prevalence of Malaria Infection in Dar es Salaam, Tanzania | Not related to immunogenicity, efficacy, and safety of TB/Malaria vaccine candidate |
|  | Otwombe et al., | 2013 | Predictors of delay in the diagnosis and treatment of suspected tuberculosis in HIV co-infected patients in South Africa | Non-vaccination et al., |
|  | Molina-Cruz et al., | 2013 | The Human Malaria Parasite Pfs47 Gene Mediates Evasion of the Mosquito Immune System | Not related to immunogenicity, efficacy, and safety of TB/Malaria vaccine candidate |
|  | Brust et al., | 2013 | Chest Radiograph Findings and Time to Culture Conversion in Patients with Multidrug-Resistant Tuberculosis and HIV in Tugela Ferry, South Africa | Not related to immunogenicity, efficacy, and safety of TB/Malaria vaccine candidate |
|  | Ouedraogo et al., | 2013 | Malaria Morbidity in High and Seasonal Malaria Transmission Area of Burkina Faso | Not related to immunogenicity, efficacy, and safety of TB/Malaria vaccine candidate |
|  | Vliegenthart-Jongbloed et al., | 2013 | Severity of imported malaria: protective effect of taking malaria chemoprophylaxis | Not related to immunogenicity, efficacy, and safety of TB/Malaria vaccine candidate |
|  | Semple et al., | 2013 | Regulatory T Cells Attenuate Mycobacterial Stasis in Alveolar and Blood-derived Macrophages from Patients with Tuberculosis | Not related to immunogenicity, efficacy, and safety of TB/Malaria vaccine candidate |
|  | Schramm et al., | 2013 | Efficacy of artesunate-amodiaquine and artemether-lumefantrine fixed-dose combinations for the treatment of uncomplicated Plasmodium falciparum malaria among children aged six to 59 months in Nimba County, Liberia: an open label randomized non-inferiority trial | Not related to immunogenicity, efficacy, and safety of TB/Malaria vaccine candidate |
|  | Ebonwu et al., | 2013 | Low treatment initiation rates among multidrug-resistant tuberculosis patients in Gauteng, South Africa, 2011 | Not related to immunogenicity, efficacy, and safety of TB/Malaria vaccine candidate |
|  | Lyra et al., | 2013 | Incidence and Risk Factors for Tuberculosis in People Living with HIV: Cohort from HIV Referral Health Centers in Recife, Brazil | Not related to immunogenicity, efficacy, and safety of TB/Malaria vaccine candidate |
|  | Sun et al., | 2013 | Cost utility of lateral-flow urine lipoarabinomannan for tuberculosis diagnosis in HIV-infected African adults | Not related to immunogenicity, efficacy, and safety of TB/Malaria vaccine candidate |
|  | Rulisa et al., | 2013 | Malaria Prevalence, Spatial Clustering and Risk Factors in a Low Endemic Area of Eastern Rwanda: A Cross-Sectional Study | Not related to immunogenicity, efficacy, and safety of TB/Malaria vaccine candidate |
|  | Diacon et al., | 2013 | Randomized Dose-Ranging Study of the 14-Day Early Bactericidal Activity of Bedaquiline (TMC207) in Patients with Sputum Microscopy Smear-Positive Pulmonary Tuberculosis | Not related to immunogenicity, efficacy, and safety of TB/Malaria vaccine candidate |
|  | Goovaerts et al., | 2013 | LPS-Binding Protein and IL-6 Mark Paradoxical Tuberculosis Immune Reconstitution Inflammatory Syndrome in HIV Patients | Not related to immunogenicity, efficacy, and safety of TB/Malaria vaccine candidate |
|  | Lawn et al., | 2013 | Diagnostic and prognostic value of serum C-reactive protein for screening for HIV-associated tuberculosis | Non-vaccination et al., |
|  | Gehre et al., | 2013 | Deciphering the Growth Behaviour of Mycobacterium africanum | Not related to immunogenicity, efficacy, and safety of TB/Malaria vaccine candidate |
|  | Mbarare et al., | 2013 | Dose-response tests and semi-field evaluation of lethal and sub-lethal effects of slow release pyriproxyfen granules (Sumilarv (R) 0.5G) for the control of the malaria vectors Anopheles gambiae sensu lato | Not related to immunogenicity, efficacy, and safety of TB/Malaria vaccine candidate |
|  | Adams et al., | 2013 | The Effect of Anti-Rosetting Agents against Malaria Parasites under Physiological Flow Conditions | Not related to immunogenicity, efficacy, and safety of TB/Malaria vaccine candidate |
|  | Moussiliou et al., | 2013 | High rates of parasite recrudescence following intermittent preventive treatment with sulphadoxine-pyrimethamine during pregnancy in Benin | Not related to immunogenicity, efficacy, and safety of TB/Malaria vaccine candidate |
|  | Littrell et al., | 2013 | Case investigation and reactive case detection for malaria elimination in northern Senegal | Not related to immunogenicity, efficacy, and safety of TB/Malaria vaccine candidate |
|  | Tran et al., | 2013 | Modulation of the complement system in monocytes contributes to tuberculosis-associated immune reconstitution inflammatory syndrome | Not related to immunogenicity, efficacy, and safety of TB/Malaria vaccine candidate |
|  | Singh et al., | 2013 | Dynamics of Forest Malaria Transmission in Balaghat District, Madhya Pradesh, India | Not related to immunogenicity, efficacy, and safety of TB/Malaria vaccine candidate |
|  | Raz et al., | 2013 | Molecular Characterization of the Carboxypeptidase B1 of Anopheles stephensi and Its Evaluation as a Target for Transmission-Blocking Vaccines | Non-vaccination et al., |
|  | Namountougou etal., | 2013 | First report of the L1014S kdr mutation in wild populations of Anopheles gambiae M and S molecular forms in Burkina Faso (West Africa) | Not related to immunogenicity, efficacy, and safety of TB/Malaria vaccine candidate |
|  | Verma et al., | 2013 | Detection of histidine rich protein & lactate dehydrogenase of Plasmodium falciparum in malaria patients by sandwich ELISA using in-house reagents | Not related to immunogenicity, efficacy, and safety of TB/Malaria vaccine candidate |
|  | Verma et al., | 2013 | Malaria vaccine can prevent millions of deaths in the world | Not related to immunogenicity, efficacy, and safety of TB/Malaria vaccine candidate |
|  | Luntamo et al., | 2013 | The effect of antenatal monthly sulphadoxinepyrimethamine, alone or with  azithromycin, on foetal and neonatal growth faltering in Malawi: a randomised controlled trial | Not related to immunogenicity, efficacy, and safety of TB/Malaria vaccine candidate |
|  | van Huijsduijnen et al., | 2013 | Anticancer Properties of Distinct Antimalarial Drug Classes | Not related to immunogenicity, efficacy, and safety of TB/Malaria vaccine candidate |
|  | Lee et al., | 2013 | Taxonomic Status of the Bemisia tabaci Complex (Hemiptera: Aleyrodidae)  and Reassessment of the Number of Its Constituent Species | Not related to immunogenicity, efficacy, and safety of TB/Malaria vaccine candidate |
|  | Wong et al., | 2013 | Standardizing operational vector sampling techniques for measuring malaria transmission intensity: evaluation of six mosquito collection methods in western Kenya | Not related to immunogenicity, efficacy, and safety of TB/Malaria vaccine candidate |
|  | Swindells et al., | 2013 | Screening for pulmonary tuberculosis in HIV-infected individuals: AIDS Clinical Trials Group Protocol A5253 | Not related to immunogenicity, efficacy, and safety of TB/Malaria vaccine candidate |
|  | Marais et al., | 2013 | Frequency, Severity, and Prediction of Tuberculous Meningitis Immune Reconstitution Inflammatory Syndrome | Non-vaccination et al., |
|  | Skinner et al., | 2013 | It's hard work, but it's worth it: the task of keeping children adherent to isoniazid preventive therapy | Not related to immunogenicity, efficacy, and safety of TB/Malaria vaccine candidate |
|  | Adukpo et al., | 2013 | High Plasma Levels of Soluble Intercellular Adhesion Molecule (ICAM)-1 Are Associated with Cerebral Malaria | Not related to immunogenicity, efficacy, and safety of TB/Malaria vaccine candidate |
|  | Sutherland et al., | 2013 | Analysis of Host Responses to Mycobacterium tuberculosis Antigens in a Multi-Site Study of Subjects with Different TB and HIV Infection States in Sub-Saharan Africa | Not related to immunogenicity, efficacy, and safety of TB/Malaria vaccine candidate |
|  | Hanrahan et al., | 2013 | Time to Treatment and Patient Outcomes among TB Suspects Screened by a Single Point-of-Care Xpert MTB/RIF at a Primary Care Clinic in Johannesburg, South Africa | Not related to immunogenicity, efficacy, and safety of TB/Malaria vaccine candidate |
|  | Pennetier et al., | 2013 | Efficacy of Olyset (R) Plus, a New Long-Lasting Insecticidal Net Incorporating Permethrin and Piperonil-Butoxide against Multi-Resistant Malaria Vectors | Not related to immunogenicity, efficacy, and safety of TB/Malaria vaccine candidate |
|  | Musoke et al.,, | 2013 | Integrated approach to malaria prevention at household level in rural communities in Uganda: experiences from a pilot project | Not related to immunogenicity, efficacy, and safety of TB/Malaria vaccine candidate |
|  | Menard et al., | 2013 | Whole Genome Sequencing of Field Isolates Reveals a Common Duplication of the Duffy Binding Protein Gene in Malagasy Plasmodium vivax Strains | Not related to immunogenicity, efficacy, and safety of TB/Malaria vaccine candidate |
|  | Van Der Plas et al., | 2013 | Complications of Antiretroviral Therapy Initiation in Hospitalised Patients with HIV-Associated Tuberculosis | Not related to immunogenicity, efficacy, and safety of TB/Malaria vaccine candidate |
|  | Littrell et al., | 2013 | Documenting malaria case management coverage in Zambia: a systems effectiveness approach | Not related to immunogenicity, efficacy, and safety of TB/Malaria vaccine candidate |
|  | Kleynhans et al., | 2013 | The Contraceptive Depot Medroxyprogesterone Acetate Impairs Mycobacterial Control and Inhibits Cytokine Secretion in Mice Infected with Mycobacterium tuberculosis | Not related to immunogenicity, efficacy, and safety of TB/Malaria vaccine candidate |
|  | Santema et al., | 2013 | Postexposure Subunit Vaccination against Chronic Enteric Mycobacterial Infection in a Natural Host | Non-vaccination et al., |
|  | Arifin et al., | 2013 | Examining the impact of larval source management and insecticide-treated nets using a spatial agent-based model of Anopheles gambiae and a landscape generator tool | Not related to immunogenicity, efficacy, and safety of TB/Malaria vaccine candidate |
|  | Cliff et al., | 2013 | Distinct Phases of Blood Gene Expression Pattern Through Tuberculosis Treatment Reflect Modulation of the Humoral Immune Response | Not related to immunogenicity, efficacy, and safety of TB/Malaria vaccine candidate |
|  | Migliori et al., | 2013 | Drug resistance beyond extensively drug-resistant tuberculosis: individual patient data meta-analysis | Not related to immunogenicity, efficacy, and safety of TB/Malaria vaccine candidate |
|  | Vial et al., | 2013 | CRIMALDDI: platform technologies and novel anti-malarial drug targets | Not related to immunogenicity, efficacy, and safety of TB/Malaria vaccine candidate |
|  | Baum et al., |  | Protein Microarray Analysis of Antibody Responses to Plasmodium falciparum in Western Kenyan Highland Sites with Differing Transmission Levels | Not related to immunogenicity, efficacy, and safety of TB/Malaria vaccine candidate |
|  | Lewis et al., |  | Eligibility for Isoniazid Preventive Therapy in South African Gold Mines | Non-vaccination et al., |
|  | Chihota et al., | 2013 | Spectrum of non-tuberculous mycobacteria identified using standard biochemical testing vs. 16S sequencing | Not related to immunogenicity, efficacy, and safety of TB/Malaria vaccine candidate |
|  | Gideon et al., | 2013 | Impairment of IFN-Gamma Response to Synthetic Peptides of Mycobacterium  tuberculosis in a 7-Day Whole Blood Assay | Not related to immunogenicity, efficacy, and safety of TB/Malaria vaccine candidate |
|  | Soares et al., | 2013 | Longitudinal Changes in CD4(+) T-Cell Memory Responses Induced by BCG  Vaccination of Newborns | Not related to immunogenicity, efficacy, and safety of TB/Malaria vaccine candidate |
|  | Juliao et al., | 2013 | Importation of chloroquine-resistant Plasmodium falciparum by Guatemalan peacekeepers returning from the Democratic Republic of the Congo | Not related to immunogenicity, efficacy, and safety of TB/Malaria vaccine candidate |
|  | Kalk et al., | 2013 | Paradoxical Mycobacterium tuberculosis Meningitis Immune Reconstitution Inflammatory Syndrome in an HIV-infected Child | Non-blind randomised study |
|  | Duparc et al., | 2013 | Safety and efficacy of pyronaridine-artesunate in uncomplicated acute malaria: an integrated analysis of individual patient data from six randomized clinical trials | Not related to immunogenicity, efficacy, and safety of TB/Malaria vaccine candidate |
|  | Auld et al., | 2013 | Incidence and Determinants of Tuberculosis among Adults Initiating Antiretroviral Therapy - Mozambique, 2004-2008 | Not related to immunogenicity, efficacy, and safety of TB/Malaria vaccine candidate |
|  | Oxborough et al., | 2013 | ITN Mixtures of Chlorfenapyr (Pyrrole) and Alphacypermethrin (Pyrethroid) for Control of Pyrethroid Resistant Anopheles arabiensis and Culex quinquefasciatus | Not related to immunogenicity, efficacy, and safety of TB/Malaria vaccine candidate |
|  | Kerkoff et al., | 2013 | Blood Neutrophil Counts in HIV-Infected Patients with PulmonaryTuberculosis: Association with Sputum Mycobacterial Load | Not related to immunogenicity, efficacy, and safety of TB/Malaria vaccine candidate |
|  | Alout et al., | 2013 | Insecticide Resistance Alleles Affect Vector Competence of Anopheles gambiae s.s. for Plasmodium falciparum Field Isolates | Not related to immunogenicity, efficacy, and safety of TB/Malaria vaccine candidate |
|  | Kumar et al., | 2013 | Circulating Biomarkers of Pulmonary and Extrapulmonary Tuberculosis in Children | Not related to immunogenicity, efficacy, and safety of TB/Malaria vaccine candidate |
|  | Jacob et al., | 2013 | Mycobacterium tuberculosis Bacteremia in a Cohort of HIV-Infected Patients Hospitalized with Severe Sepsis in Uganda-High Frequency, Low Clinical Sand Derivation of a Clinical Prediction Score | Not related to immunogenicity, efficacy, and safety of TB/Malaria vaccine candidate |
|  | Quashie et al., | 2013 | A SYBR Green 1-based in vitro test of susceptibility of Ghanaian Plasmodium falciparum clinical isolates to a panel of anti-malarial drugs | Not related to immunogenicity, efficacy, and safety of TB/Malaria vaccine candidate |
|  | Peter et al., | 2013 | Can Point-of-Care Urine LAM Strip Testing for Tuberculosis Add Value to Clinical Decision Making in Hospitalised HIV-Infected Persons? | Not related to immunogenicity, efficacy, and safety of TB/Malaria vaccine candidate |
|  | Nolan et al., | 2013 | Increased Production of IL-4 and IL-12p40 from Bronchoalveolar Lavage Cells Are Biomarkers of Mycobacterium tuberculosis in the Sputum | Not related to immunogenicity, efficacy, and safety of TB/Malaria vaccine candidate |
|  | Axelsson-Robertson et al., | 2013 | A Broad Profile of Co-Dominant Epitopes Shapes the Peripheral Mycobacterium tuberculosis Specific CD8+T-Cell Immune Response in South  African Patients with Active Tuberculosis | Non-blind randomised study |
|  | Le Roux | 2013 | Safety of long-term isoniazid preventive therapy in children with HIV: a comparison of two dosing schedules | Not related to immunogenicity, efficacy, and safety of TB/Malaria vaccine candidate |
|  | Ravimohan et al., | 2013 | Early Immunologic Failure is Associated with Early Mortality Among Advanced HIV-Infected Adults Initiating Antiretroviral Therapy with Active Tuberculosis | Not related to immunogenicity, efficacy, and safety of TB/Malaria vaccine candidate |
|  | Mharakurwa et al., | 2013 | Changes in the burden of malaria following scale up of malaria control interventions in Mutasa District, Zimbabwe | Not related to immunogenicity, efficacy, and safety of TB/Malaria vaccine candidate |
|  | Abuogi et al., | 2013 | Impact of expanded antiretroviral use on incidence and prevalence of tuberculosis in children with HIV in Kenya | Not related to immunogenicity, efficacy, and safety of TB/Malaria vaccine candidate |
|  | Rozot et al., | 2013 | Mycobacterium tuberculosis-specific CD8(+) T cells are functionally and phenotypically different between latent infection and active disease | Not related to immunogenicity, efficacy, and safety of TB/Malaria vaccine candidate |
|  | Haji et al., | 2013 | Challenges for malaria elimination in Zanzibar: pyrethroid resistance in malaria vectors and poor performance of long-lasting insecticide nets | Not related to immunogenicity, efficacy, and safety of TB/Malaria vaccine candidate |
|  | Gupta et al., | 2013 | Impact of human immunodeficiency virus and CD4 count on tuberculosis diagnosis: analysis of city-wide data from Cape Town, South Africa | Not related to immunogenicity, efficacy, and safety of TB/Malaria vaccine candidate |
|  | Boissiere et al., | 2013 | Application of a qPCR Assay in the Investigation of Susceptibility to Malaria Infection of the M and S Molecular Forms of An. gambiae s.s. in Cameroon | Not related to immunogenicity, efficacy, and safety of TB/Malaria vaccine candidate |
|  | Wieten et al., | 2013 | Towards improved uptake of malaria chemoprophylaxis among West African travellers: identification of behavioural determinants | Not related to immunogenicity, efficacy, and safety of TB/Malaria vaccine candidate |
|  | Bryant et al., | 2013 | Whole-genome sequencing to establish relapse or re-infection with Mycobacterium tuberculosis: a retrospective observational study | Not related to immunogenicity, efficacy, and safety of TB/Malaria vaccine candidate |
|  | Dodd et al., | 2013 | Predicting the Long-Term Impact of Antiretroviral Therapy Scale-Up on Population Incidence of Tuberculosis | Not related to immunogenicity, efficacy, and safety of TB/Malaria vaccine candidate |
|  | Wilson et al., | 2013 | Skewing of the CD4(+) T-Cell Pool Toward Monofunctional Antigen-Specific  Responses in Patients with Immune Reconstitution Inflammatory Syndrome in The Gambia | Not related to immunogenicity, efficacy, and safety of TB/Malaria vaccine candidate |
|  | Day et al., | 2013 | Induction and Regulation of T-Cell Immunity by the Novel Tuberculosis Vaccine M72/AS01 in South African Adults | Non-blind randomised study |
|  | Moon et al., | 2013 | Adaptation of the genetically tractable malaria pathogen Plasmodium knowlesi to continuous culture in human erythrocytes | Not related to immunogenicity, efficacy, and safety of TB/Malaria vaccine candidate |
|  | Prugnolle et al., | 2013 | Diversity, host switching and evolution of Plasmodium vivax infecting African great apes | Not related to immunogenicity, efficacy, and safety of TB/Malaria vaccine candidate |
|  | Essandoh et al., | 2013 | Acetylcholinesterase (Ace-1) target site mutation 119S is strongly diagnostic of carbamate and organophosphate resistance in Anopheles gambiae s.s. and Anopheles coluzzii across southern Ghana | Not related to immunogenicity, efficacy, and safety of TB/Malaria vaccine candidate |
|  | Sakamoto et al., | 2013 | Mycobacterial Trehalose Dimycolate Reprograms Macrophage Global Gene Expression and Activates Matrix Metalloproteinases | Not related to immunogenicity, efficacy, and safety of TB/Malaria vaccine candidate |
|  | Belderok et al., | 2013 | Adherence to Chemoprophylaxis and Plasmodium falciparum Anti-Circumsporozoite Seroconversion in a Prospective Cohort Study of Dutch Short-Term Travelers | Not related to immunogenicity, efficacy, and safety of TB/Malaria vaccine candidate |
|  | Grode et al., | 2013 | Safety and immunogenicity of the recombinant BCG vaccine VPM1002 in a phase 1 open label randomized clinical trial | Non-sub-Saharan African study |
|  | Madhi et al., | 2013 | Immunogenicity following the first and second doses of 7-valent pneumococcal conjugate vaccine in HIV-infected and -uninfected infants | Not related to immunogenicity, efficacy, and safety of TB/Malaria vaccine candidate |
|  | Motswagole et al., |  | The efficacy of micronutrient-fortified sorghum meal in improving the immune status of HIV-positive adults | Not related to immunogenicity, efficacy, and safety of TB/Malaria vaccine candidate |
|  | Bork et al., | 2013 | Infant feeding modes and determinants among HIV-1-infected African women in the kesho bora study | Not related to immunogenicity, efficacy, and safety of TB/Malaria vaccine candidate |
|  | Watson-Jones et al., | 2013 | High prevalence and incidence of human papillomavirus in a cohort of Healthy young African female subjects | Not related to immunogenicity, efficacy, and safety of TB/Malaria vaccine candidate |
|  | Thirlwall et al., | 2013 | Treatment of child anxiety disorders via guided parent-delivered cognitive-behavioural therapy: Randomised controlled trial | Not related to immunogenicity, efficacy, and safety of TB/Malaria vaccine candidate |
|  | Anita et al., | 2013 | A randomized controlled pilot study of an HIV risk-reduction intervention for sub-Saharan African University Students | Non-TB/malaria study |
|  | Okello et al., | 2013 | Challenges for consent and community engagement in the conduct of cluster randomized trial among school children in low-income settings: Experiences from Kenya | Not related to immunogenicity, efficacy, and safety of TB/Malaria vaccine candidate |
|  | Jones et al., | 2013 | The dynamics of pyrethroid resistance in Anopheles arabiensis from Zanzibar and an assessment of the underlying genetic basis | Not related to immunogenicity, efficacy, and safety of TB/Malaria vaccine candidate |
|  | Baumgartner et al., | 2013 | Overweight impairs efficacy of iron supplementation in iron-deficient South African children: A randomized controlled intervention |  |
|  | Todd et al., | 2013 | When enough is enough: How the decision was made to stop the FEAST trial: Data and safety monitoring in an African trial of Fluid Expansion as Supportive Therapy (FEAST) for critically ill children | Not related to immunogenicity, efficacy, and safety of TB/Malaria vaccine candidate |
|  | Walson et al., | 2013 | Evaluation of impact of long-lasting insecticide-treated bed nets and point-of-use water filters on HIV-1 disease progression in Kenya | Not related to immunogenicity, efficacy, and safety of TB/Malaria vaccine candidate |
|  | Sow et al., | 2013 | Safety and immunogenicity of human papillomavirus-16/18 AS04-adjuvanted vaccine: A randomized trial in 10-25-year-old HIV-seronegative African girls and young women | Non-TB/malaria study |
|  | Jarvis et al., | 2013 | The phenotype of the cryptococcus-specific CD4+ memory T-cell response is associated with disease severity and outcome in HIV-associated cryptococcal meningitis | Non-TB/malaria study |
|  | Wang et al., | 2019 | Monitoring and evaluation of intervals from onset of fever to diagnosis before ``1-3-7'' approach in malaria elimination: a retrospective study in Shanxi Province, China from 2013 to 2018 | Not related to immunogenicity, efficacy, and safety of TB/Malaria vaccine candidate |
|  | Kulkarni et al., | 2013 | Safety and immunogenicity of a new purified vero cell rabies vaccine (PVRV) administered by intramuscular and intradermal routes in healthy volunteers | Not related to immunogenicity, efficacy, and safety of TB/Malaria vaccine candidate |
|  | Abdul-Ghani et al., | 2013 | Mutant Plasmodium falciparum chloroquine resistance transporter in Hodeidah, Yemen: Association with parasitologic indices and treatment-seeking behaviors | Not related to immunogenicity, efficacy, and safety of TB/Malaria vaccine candidate |
|  | Vrieling et al., | 2013 | Hsp70 vaccination-induced primary immune responses in efferent lymph of the draining lymph node | Not related to immunogenicity, efficacy, and safety of TB/Malaria vaccine candidate |
|  | Danaviah et al., | 2013 | Immunohistological characterization of spinal TB granulomas from HIV-negative and -positive patients | Not related to immunogenicity, efficacy, and safety of TB/Malaria vaccine candidate |
|  | Naidoo | 2013 | A randomized placebo-controlled pilot study of Cat saliva 9cH and Histaminum 9cH in cat allergic adults | Pre-clinical study |
|  |  |  |  |  |
|  | Denny et al., | 2013 | Safety and immunogenicity of the HPV-16/18 AS04-adjuvanted vaccine in HIV-positive women in South Africa: A partially blind randomised placebo-controlled study | Non-TB/malaria vaccination study |
|  | Mahomed et al., | 2013 | Screening for TB in high school adolescents in a high burden setting in South Africa | Not related to immunogenicity, efficacy, and safety of TB/Malaria vaccine candidate |
|  | Gendrot et al., | 2020 | Baseline and multinormal distribution of ex vivo susceptibilities of Plasmodium falciparum to methylene blue in Africa, 2013-18 | Not related to immunogenicity, efficacy, and safety of TB/Malaria vaccine candidate |
|  | Roman et al., | 2013 | Therapeutic vaccination using cationic liposome-adjuvanted HIV type 1 peptides representing HLA-supertype-restricted subdominant t cell epitopes: Safety, immunogenicity, and feasibility in guinea-Bissau | Non-TB/malaria vaccination study |
|  | Tejedor-Garavito et al., | 2010 | Travel patterns and demographic characteristics of malaria cases in Swaziland, 2010-2014 | Not related to immunogenicity, efficacy, and safety of TB/Malaria vaccine candidate |
|  | Gonzalo-Asensio et al., | 2008 | PhoP: a missing piece in the intricate puzzle of Mycobacterium tuberculosis virulence | Not related to immunogenicity, efficacy, and safety of TB/Malaria vaccine candidate |
|  | Imoukhuede et al., | 2006 | Safety and immunogenicity of the malaria candidate vaccines FP9 CS and MVA CS in adult Gambian men | Publication year |
|  | Keating et al., | 2005 | Durable human memory T cells quantifiable by cultured enzyme-linked immunospot assays are induced by heterologous prime boost immunization and correlate with protection against malaria | Publication year |
|  | Goosen et al., | 2014 | The evaluation of candidate biomarkers of cell-mediated immunity for the diagnosis of Mycobacterium bovis infection in African buffaloes (Syncerus caffer) | Not related to immunogenicity, efficacy, and safety of TB/Malaria vaccine candidate |
|  | Hyde et al., | 2014 | A Randomised Controlled Trial of complete denture impression materials | Not related to immunogenicity, efficacy, and safety of TB/Malaria vaccine candidate |
|  | Li et al., | 2014 | K13-propeller gene polymorphisms of Plasmodium falciparum and the therapeutic effect of artesunate among migrant workers returning to Guangxi, China (2014-2017) | Not related to immunogenicity, efficacy, and safety of TB/Malaria vaccine candidate |
|  | Hill et al., | 2014 | Influence of compression garments on recovery after marathon running | Not related to immunogenicity, efficacy, and safety of TB/Malaria vaccine candidate |
|  | Maman et al., | 2014 | NIMH projects accept (HPTN 043): Results from in-depth interviews with a longitudinal cohort of community members | Not related to immunogenicity, efficacy, and safety of TB/Malaria vaccine candidate |
|  | Pengpid et al., | 2014 | Efficacy of a church-based lifestyle intervention programme to control high normal blood pressure and/or high normal blood glucose in church members: A randomized controlled trial in Pretoria, South Africa | Not related to immunogenicity, efficacy, and safety of TB/Malaria vaccine candidate |
|  | Chetty et al., | 2014 | Tuberculosis distorts the inhibitory impact of interleukin-10 in HIV infection | Not related to immunogenicity, efficacy, and safety of TB/Malaria vaccine candidate |
|  | Nkonki et al., | 2014 | Costs of promoting exclusive breastfeeding at community level in three sites in South Africa | Not related to immunogenicity, efficacy, and safety of TB/Malaria vaccine candidate |
|  | Essone et al., | 2014 | Bifunctional T-Cell-Derived Cytokines for the Diagnosis of Tuberculosis and Treatment Monitoring | Not related to immunogenicity, efficacy, and safety of TB/Malaria vaccine candidate |
|  | Pretorius et al., | 2014 | The potential effects of changing HIV treatment policy on tuberculosis outcomes in South Africa: results from three tuberculosis-HIV transmission models | Not related to immunogenicity, efficacy, and safety of TB/Malaria vaccine candidate |
|  | Maman et al., | 2014 | Efficacy of enhanced HIV counseling for risk reduction during pregnancy and in the postpartum period: A randomized controlled trial | Not related to immunogenicity, efficacy, and safety of TB/Malaria vaccine candidate |
|  | Mash et al., | 2014 | Effectiveness of a group diabetes education programme in under-served communities in South Africa: A pragmatic cluster randomized controlled trial | Not related to immunogenicity, efficacy, and safety of TB/Malaria vaccine candidate |
|  | Eloff et al., | 2014 | A randomized clinical trial of an intervention to promote resilience in young children of HIV-positive mothers in South Africa | Not related to immunogenicity, efficacy, and safety of TB/Malaria vaccine candidate |
|  | Eloff et al., | 2014 | A randomized clinical trial of an intervention to promote resilience in young children of HIV-positive mothers in South Africa | Not related to immunogenicity, efficacy, and safety of TB/Malaria vaccine candidate |
|  | Scott et al., | 2014 | Timing of bacterial carriage sampling in vaccine trials: A modelling study | Not related to immunogenicity, efficacy, and safety of TB/Malaria vaccine candidate |
|  | Tefit et al., | 2014 | Efficacy of ABX196, a new NKT agonist, in prophylactic human vaccination | Non-TB/malaria vaccination study |
|  | Armistead et al., | 2014 | Preliminary results from a family-based HIV prevention intervention for South African youth | Non-TB/malaria study |
|  | Sarnquist et al., | 2014 | Rape prevention through empowerment of adolescent girls | Not related to immunogenicity, efficacy, and safety of TB/Malaria vaccine candidate |
|  | Harris et al., | 2014 | Process of assay selection and optimization for the study of case and control samples from a phase IIb efficacy trial of a candidate tuberculosis vaccine, MVA85A | Secondary analysis |
|  | Hopkins et al., | 2014 | Predictors of HVTN 503 MRK-AD5 HIV-1 gag/pol/nef vaccine induced immune responses | Non-TB/malaria vaccination study |
|  | mcDonald et al., | 2014 | A double blind randomized controlled trial in neonates to determine the effect of vitamin A supplementation on immune responses: The Gambia protocol | Not related to immunogenicity, efficacy, and safety of TB/Malaria vaccine candidate |
|  | Duvignaud et al., | 2014 | Incidence of malaria-related fever and morbidity due to Plasmodium falciparum among HIV1-infected pregnant women: A prospective cohort study in South Benin | Not related to immunogenicity, efficacy, and safety of TB/Malaria vaccine candidate |
|  | Gray et al., | 2014 | Recombinant adenovirus type 5 HIV gag/pol/nef vaccine in South Africa: Unblinded, long-term follow-up of the phase 2b HVTN 503/Phambili study | Non-TB/malaria study |
|  | Olupot-Olupot et al., | 2014 | Phase II trial of standard versus increased transfusion volume in Ugandan children with acute severe anemia | Non-TB/malaria study |
|  | Buchbinder et al., | 2014 | HIV pre-exposure prophylaxis in men who have sex with men and transgender women: A secondary analysis of a phase 3 randomised controlled efficacy trial | Non-TB/malaria study |
|  | Ho et al., | 2015 | Implementation workshop of WHO guidelines on evaluation of Malaria vaccines: Current regulatory concepts and issues related to vaccine quality, Pretoria, South Africa 07 Nov 2014 | Not related to immunogenicity, efficacy, and safety of TB/Malaria vaccine candidate |
|  | Mantell et al., | 2014 | Pregnancy intent among a sample of recently diagnosed HIV-positive women and men practicing unprotected sex in Cape Town, South Africa | Non-malaria/TB study |
|  | Bain | 2014 | "New beginnings" in South African shelters for the homeless: Piloting of a group psychotherapy intervention for high-risk mother-infant dyads | Non-malaria/TB study |
|  | Zhang et al., | 2014 | Parameters for Sample Size Estimation from a Group-Randomized HIV Prevention Trial in HIV Clinics in Sub-Saharan Africa | Non-malaria/TB study |
|  | Jemmott et al., | 2014 | 'Let Us Protect Our Future' a culturally congruent evidenced-based HIV/STD risk-reduction intervention for young South African adolescents | Non-malaria/TB study |
|  | Lazarus et al., | 2014 | Uptake of genital mucosal sampling in HVTN 097, a Phase 1b HIV vaccine trial in South Africa | Non-malaria/TB study |
|  | Ward et al., | 2014 | Screening and brief intervention for substance misuse: Does it reduce aggression and HIV-related risk behaviours? | Non-malaria/TB study |
|  | Atukunda et al., | 2014 | Sublingual Misoprostol versus Intramuscular Oxytocin for Prevention of Postpartum Hemorrhage in Uganda: A Double-Blind Randomized Non-Inferiority Trial | Non-TB/malaria study |
|  | Jemmott et al., | 2014 | Cluster-randomized controlled trial of an HIV/sexually transmitted infection risk-reduction intervention for South African men | Non-TB/malaria study |
|  | Heffron et al., | 2014 | A prospective study of the effect of pregnancy on CD4 counts and plasma HIV-1 RNA concentrations of antiretroviral-naive HIV-1-infected women | Non-TB/malaria study |
|  | Barrera et al., | 2014 | Keywords to recruit spanish- and english-speaking participants: Evidence from an online postpartum depression randomized controlled trial | Non-TB/malaria study |
|  | Gonzalez et al., | 2014 | Intermittent Preventive Treatment of Malaria in Pregnancy with Mefloquine in HIV-Negative Women: A Multicentre Randomized Controlled Trial | Not related to immunogenicity, efficacy, and safety of TB/Malaria vaccine candidate |
|  | Louwagie et al., | 2014 | Efficacy of brief motivational interviewing on smoking cessation at tuberculosis clinics in Tshwane, South Africa: A randomized controlled trial | Non-TB/malaria study |
|  | Jafari et al., | 2014 | Efficacy of inactivated poliovirus vaccine in India | Non-malaria/TB study |
|  | Tarantino et al., | 2014 | Safety-related moderators of a parent-based HIV prevention intervention in south Africa | Non-malaria/TB study |
|  | Kaleebu, et al., | 2014 | Immunogenicity of ALVAC-HIV vCP1521 in Infants of HIV-1-Infected Women in Uganda (HPTN 027): The First Pediatric HIV Vaccine Trial in Africa | Non-malaria/TB study |
|  | Fowler et al., | 2014 | Efficacy and safety of an extended nevirapine regimen in infants of breastfeeding mothers with HIV-1 infection for prevention of HIV-1 transmission (HPTN 046): 18-month results of a randomized, double-blind, placebo-controlled trial | Non-TB/malaria study |
|  | Brown et al., | 2014 | Impact of malaria and helminth infections on immunogenicity of the human papillomavirus-16/18 AS04-adjuvanted vaccine in Tanzania | Not related to immunogenicity, efficacy, and safety of TB/Malaria vaccine candidate |
|  | Kotlyar et al., | 2014 | Spleen volume and clinical disease manifestations of severe Plasmodium falciparum malaria in African children | Not related to immunogenicity, efficacy, and safety of TB/Malaria vaccine candidate |
|  | Auld et al., | 2014 | Tuberculosis in human immunodeficiency virus-infected children starting antiretroviral therapy in Cote d'Ivoire | Not related to immunogenicity, efficacy, and safety of TB/Malaria vaccine candidate |
|  | Siev et al., | 2014 | Antibodies against Mycobacterial Proteins as Biomarkers for HIV-Associated Smear-Negative Tuberculosis | Not related to immunogenicity, efficacy, and safety of TB/Malaria vaccine candidate |
|  | Coulibaly et al., | 2014 | Stable malaria incidence despite scaling up control strategies in a malaria vaccine-testing site in Mali | Not related to immunogenicity, efficacy, and safety of TB/Malaria vaccine candidate |
|  | Salie et al., | 2014 | Associations Between Human Leukocyte Antigen Class I Variants and the Mycobacterium tuberculosis Subtypes Causing Disease | Not related to immunogenicity, efficacy, and safety of TB/Malaria vaccine candidate |
|  | Planting et al., | 2014 | Safety and efficacy of induced sputum in young children hospitalised with suspected pulmonary tuberculosis | Not related to immunogenicity, efficacy, and safety of TB/Malaria vaccine candidate |
|  | Manabe et al., | 2014 | Point-of-Care Lateral Flow Assays for Tuberculosis and Cryptococcal Antigenuria Predict Death in HIV Infected Adults in Uganda | Not related to immunogenicity, efficacy, and safety of TB/Malaria vaccine candidate |
|  | Kyeyune et al., | 2014 | The interaction between malaria and human immunodeficiency virus infection in severely anaemic Malawian children: prospective longitudinal study | Not related to immunogenicity, efficacy, and safety of TB/Malaria vaccine candidate |
|  | Pfeil et al., | 2014 | Dihydroartemisinin-Piperaquine vs. Artemether-Lumefantrine for First-Line Treatment of Uncomplicated Malaria in African Children: A Cost-Effectiveness Analysis | Not related to immunogenicity, efficacy, and safety of TB/Malaria vaccine candidate |
|  | Kirenga et al., | 2014 | Treatment Outcomes of New Tuberculosis Patients Hospitalized in Kampala, Uganda: A Prospective Cohort Study | Not related to immunogenicity, efficacy, and safety of TB/Malaria vaccine candidate |
|  | Sirugo et al., | 2014 | Short Report: G6PD A- Deficiency and Severe Malaria in The Gambia: Heterozygote Advantage and Possible Homozygote Disadvantage | Not related to immunogenicity, efficacy, and safety of TB/Malaria vaccine candidate |
|  | Oladimeji et al., | 2014 | Intensive-Phase Treatment Outcomes among Hospitalized Multidrug-Resistant Tuberculosis Patients: Results from a Nationwide Cohort in Nigeria | Not related to immunogenicity, efficacy, and safety of TB/Malaria vaccine candidate |
|  | Nambozi et al., | 2014 | Defining the malaria burden in Nchelenge District, northern Zambia using the World Health Organization malaria indicators survey | Not related to immunogenicity, efficacy, and safety of TB/Malaria vaccine candidate |
|  | Mbeye et al., | 2014 | Cotrimoxazole prophylactic treatment prevents malaria in children in sub-Saharan Africa: systematic review and meta-analysis | Not related to immunogenicity, efficacy, and safety of TB/Malaria vaccine candidate |
|  | Okell et al., | 2014 | Contrasting benefits of different artemisinin combination therapies as first-line malaria treatments using model-based cost-effectiveness analysis | Not related to immunogenicity, efficacy, and safety of TB/Malaria vaccine candidate |
|  | Sutherland et al., | 2014 | Differential gene expression of activating Fc gamma receptor classifies active tuberculosis regardless of human immunodeficiency virus status or ethnicity | Not related to immunogenicity, efficacy, and safety of TB/Malaria vaccine candidate |
|  | Teixeria et al., | 2014 | Immunogenicity of a Prime-Boost Vaccine Containing the Circumsporozoite Proteins of Plasmodium vivax in Rodents | Pre-clinical study |
|  | jagannathan | 2014 | IFN gamma/IL-10 Co-producing Cells Dominate the CD4 Response to Malaria in Highly Exposed Children | Not related to immunogenicity, efficacy, and safety of TB/Malaria vaccine candidate |
|  | Tameris et al., | 2014 | The Candidate TB Vaccine, MVA85A, Induces Highly Durable Th1 Responses | Descriptive study |
|  | Gresty et al., | 2014 | Genetic mutations in pfcrt and pfmdr1 at the time of artemisinin combination therapy introduction in South Pacific islands of Vanuatu and Solomon Islands | Not related to immunogenicity, efficacy, and safety of TB/Malaria vaccine candidate |
|  | Coulibaly et al., | 2014 | Parasite clearance following treatment with sulphadoxine-pyrimethamine for intermittent preventive treatment in Burkina-Faso and Mali: 42-day in vivo follow-up study | Not related to immunogenicity, efficacy, and safety of TB/Malaria vaccine candidate |
|  | Adepititi et al., | 2014 | In vivo antimalarial evaluation of MAMA decoction on Plasmodium berghei in mice | Not related to immunogenicity, efficacy, and safety of TB/Malaria vaccine candidate |
|  | Cumming et al., | 2014 | The Physiology and Genetics of Oxidative Stress in Mycobacteria | Not related to immunogenicity, efficacy, and safety of TB/Malaria vaccine candidate |
|  | Xu | 2014 | Traditional Chinese Medicines Against Malaria | Not related to immunogenicity, efficacy, and safety of TB/Malaria vaccine candidate |
|  | Mosha et al., | 2014 | Hot spot or not: a comparison of spatial statistical methods to predict prospective malaria infections | Not related to immunogenicity, efficacy, and safety of TB/Malaria vaccine candidate |
|  | Day et al., | 2014 | Patients with Tuberculosis Disease Have Mycobacterium tuberculosis-Specific CD8 T Cells with a Pro-Apoptotic Phenotype and Impaired Proliferative Capacity, Which Is Not Restored following Treatment | Not related to immunogenicity, efficacy, and safety of TB/Malaria vaccine candidate |
|  | Link-Gelles et al., | 2014 | Tuberculosis Immune Reconstitution Inflammatory Syndrome in Children Initiating Antiretroviral Therapy for HIV Infection: A Systematic Literature Review | Not related to immunogenicity, efficacy, and safety of TB/Malaria vaccine candidate |
|  | Shey et al., | 2014 | Maturation of Innate Responses to Mycobacteria over the First Nine Months of Life | Not related to immunogenicity, efficacy, and safety of TB/Malaria vaccine candidate |
|  | Drain et al., | 2014 | Diagnostic accuracy and clinical role of rapid C-reactive protein testing in HIV-infected individuals with presumed tuberculosis in South Africa | Not related to immunogenicity, efficacy, and safety of TB/Malaria vaccine candidate |
|  | Sagara et al., | 2014 | Delayed anemia assessment in patients treated with oral artemisinin derivatives for uncomplicated malaria: a pooled analysis of clinical trials data from Mali | Not related to immunogenicity, efficacy, and safety of TB/Malaria vaccine candidate |
|  | Djenontin et al., | 2014 | Field Efficacy of Vectobac GR as a Mosquito Larvicide for the Control of Anopheline and Culicine Mosquitoes in Natural Habitats in Benin, West Africa | Not related to immunogenicity, efficacy, and safety of TB/Malaria vaccine candidate |
|  | Dambach et al., | 2014 | Efficacy of Bacillus thuringiensis var. israelensis against malaria mosquitoes in northwestern Burkina Faso | Not related to immunogenicity, efficacy, and safety of TB/Malaria vaccine candidate |
|  | Hasse et al., | 2014 | Co-Trimoxazole Prophylaxis Is Associated with Reduced Risk of Incident Tuberculosis in Participants in the Swiss HIV Cohort Study | Not related to immunogenicity, efficacy, and safety of TB/Malaria vaccine candidate |
|  | Antonio-Nkondjio et al., | 2014 | Exposure to disinfectants (soap or hydrogen peroxide) increases tolerance to permethrin in Anopheles gambiae populations from the city of Yaounde, Cameroon | Not related to immunogenicity, efficacy, and safety of TB/Malaria vaccine candidate |
|  | Aikpon et al., | 2014 | Good performances but short-lasting efficacy of Actellic 50 EC Indoor Residual Spraying (IRS) on malaria transmission in Benin, West Africa | Not related to immunogenicity, efficacy, and safety of TB/Malaria vaccine candidate |
|  | Mori et al., | 2014 | Cost-effectiveness of dihydroartemisinin-piperaquine compared with artemether-lumefantrine for treating uncomplicated malaria in children at a district hospital in Tanzania | Not related to immunogenicity, efficacy, and safety of TB/Malaria vaccine candidate |
|  | Toe et al., | 2014 | Increased Pyrethroid Resistance in Malaria Vectors and Decreased Bed Net Effectiveness, Burkina Faso | Not related to immunogenicity, efficacy, and safety of TB/Malaria vaccine candidate |
|  | Manyando et al., | 2014 | Rapid diagnostic tests for malaria and health workers' adherence to test results at health facilities in Zambia | Not related to immunogenicity, efficacy, and safety of TB/Malaria vaccine candidate |
|  | Danel et al., | 2014 | Quantiferon-TB Gold: Performance for Ruling out Active Tuberculosis in HIV-Infected Adults with High CD4 Count in Cote d'Ivoire, West Africa | Not related to immunogenicity, efficacy, and safety of TB/Malaria vaccine candidate |
|  | Abdi et al., | 2014 | Measuring Soluble ICAM-1 in African Populations | Not related to immunogenicity, efficacy, and safety of TB/Malaria vaccine candidate |
|  | Toure et al., | 2014 | Coverage and efficacy of intermittent preventive treatment with sulphadoxine pyrimethamine against malaria in pregnancy in Cote d'Ivoire five years after its implementation | Not related to immunogenicity, efficacy, and safety of TB/Malaria vaccine candidate |
|  | Andrade et al., | 2014 | Mycobacterial Antigen Driven Activation of CD14(++) CD16(-) Monocytes Is  a Predictor of Tuberculosis-Associated Immune Reconstitution Inflammatory Syndrome | Not related to immunogenicity, efficacy, and safety of TB/Malaria vaccine candidate |
|  | Parihar et al., | 2014 | Statin Therapy Reduces the Mycobacterium tuberculosis Burden in Human Macrophages and in Mice by Enhancing Autophagy and Phagosome Maturation | Not related to immunogenicity, efficacy, and safety of TB/Malaria vaccine candidate |
|  | Zvada et al., | 2014 | Moxifloxacin Population Pharmacokinetics and Model-Based Comparison of Efficacy between Moxifloxacin and Ofloxacin in African Patients | Not related to immunogenicity, efficacy, and safety of TB/Malaria vaccine candidate |
|  | Dharmadhikari et al., | 2014 | Rapid impact of effective treatment on transmission of multidrug-resistant tuberculosis | Not related to immunogenicity, efficacy, and safety of TB/Malaria vaccine candidate |
|  | Marcy et al., | 2014 | Causes and Determinants of Mortality in HIV-Infected Adults with Tuberculosis: An Analysis from the CAMELIA ANRS 1295-CIPRA KH001  Randomized Trial | Not related to immunogenicity, efficacy, and safety of TB/Malaria vaccine candidate |
|  | Hanrahan et al., | 2014 | Xpert MTB/RIF as a Measure of Sputum Bacillary Burden Variation by HIV Status and Immunosuppression | Not related to immunogenicity, efficacy, and safety of TB/Malaria vaccine candidate |
|  | Gupta et al., | 2014 | Cost-effectiveness of the Three I's for HIV/TB and ART to prevent TB among people living with HIV | Not related to immunogenicity, efficacy, and safety of TB/Malaria vaccine candidate |
|  | Aguiar et al., | 2014 | Plasmodium vivax and Plasmodium falciparum ex vivo susceptibility to anti-malarials and gene characterization in Rondonia, West Amazon, Brazil | Not related to immunogenicity, efficacy, and safety of TB/Malaria vaccine candidate |
|  | Riou et al., | 2014 | A Subset of Circulating Blood Mycobacteria-Specific CD4 T Cells Can Predict the Time to Mycobacterium tuberculosis Sputum Culture Conversion | Not related to immunogenicity, efficacy, and safety of TB/Malaria vaccine candidate |
|  | Chapman et al., | 2014 | The Use of Directed Evolution to Create a Stable and Immunogenic Recombinant BCG Expressing a Modified HIV-1 Gag Antigen | Not related to immunogenicity, efficacy, and safety of TB/Malaria vaccine candidate |
|  | Nkye et al., | 2014 | Insecticide resistance mechanisms associated with different environments in the malaria vector Anopheles gambiae: a case study in Tanzania | Not related to immunogenicity, efficacy, and safety of TB/Malaria vaccine candidate |
|  | Fairlie et al., | 2014 | Microbiological investigation for tuberculosis among HIV-infected children in Soweto, South Africa | Not related to immunogenicity, efficacy, and safety of TB/Malaria vaccine candidate |
|  | Doderer-lang et al., | 2014 | The ears of the African elephant: unexpected high seroprevalence of Plasmodium ovale and Plasmodium malariae in healthy populations in Western Africa | Not related to immunogenicity, efficacy, and safety of TB/Malaria vaccine candidate |
|  | Navarro et al., | 2014 | Metal-chloroquine derivatives as possible anti-malarial drugs: evaluation of anti-malarial activity and mode of action | Not related to immunogenicity, efficacy, and safety of TB/Malaria vaccine candidate |
|  | Aichelburg et al., | 2014 | The association of smoking with IGRA and TST results in HIV-1-infected  subjects | Not related to immunogenicity, efficacy, and safety of TB/Malaria vaccine candidate |
|  | Mangesho et al., | 2014 | ``Every drug goes to treat its own disease`` - a qualitative study of perceptions and experiences of taking anti-retrovirals concomitantly with anti-malarials among those affected by HIV and malaria in Tanzania | Not related to immunogenicity, efficacy, and safety of TB/Malaria vaccine candidate |
|  | Cox et al., | 2014 | Community-based treatment of drug-resistant tuberculosis in Khayelitsha, South Africa | Not related to immunogenicity, efficacy, and safety of TB/Malaria vaccine candidate |
|  | Ngufor et al., | 2014 | Combining organophosphate-treated wall linings and long-lasting insecticidal nets fail to provide additional control over long-lasting insecticidal nets alone against multiple insecticide-resistant Anopheles gambiae in Cote d'Ivoire: an experimental hut trial | Not related to immunogenicity, efficacy, and safety of TB/Malaria vaccine candidate |
|  | Wallis | 2014 | Corticosteroid Effects on Sputum Culture in Pulmonary Tuberculosis: A Meta-Regression Analysis | Not related to immunogenicity, efficacy, and safety of TB/Malaria vaccine candidate |
|  | Arnott et al., | 2014 | Distinct patterns of diversity, population structure and evolution in the AMA1 genes of sympatric Plasmodium falciparum and Plasmodium vivax populations of Papua New Guinea from an area of similarly high transmission | Not related to immunogenicity, efficacy, and safety of TB/Malaria vaccine candidate |
|  | Furin et al., | 2014 | `I'm fed up': experiences of prior anti-tuberculosis treatment in patients with drug-resistant tuberculosis and HIV | Not related to immunogenicity, efficacy, and safety of TB/Malaria vaccine candidate |
|  | Aurin et al., | 2014 | Molecular Approaches for Detection of the Multi-Drug-Resistant Tuberculosis (MDR-TB) in Bangladesh | Not related to immunogenicity, efficacy, and safety of TB/Malaria vaccine candidate |
|  | Badejo et al., | 2014 | Interaction between rifampicin, amodiaquine and artemether in mice infected with chloroquine resistant Plasmodium berghei | Not related to immunogenicity, efficacy, and safety of TB/Malaria vaccine candidate |
|  | Barogui et al., | 2014 | Contribution of the Community Health Volunteers in the Control of Buruli Ulcer in Benin | Non-TB/malaria study |
|  | BedU-Addo et al., | 2014 | Reduced prevalence of placental malaria in primiparae with blood group O | Not related to immunogenicity, efficacy, and safety of TB/Malaria vaccine candidate |
|  | Marais et al., | 2014 | Neutrophil-Associated Central Nervous System Inflammation in Tuberculous Meningitis Immune Reconstitution Inflammatory Syndrome | Not related to immunogenicity, efficacy, and safety of TB/Malaria vaccine candidate |
|  | Ditkowsky et al., | 2014 | Potential Cost-Effectiveness of a New Infant Tuberculosis Vaccine in South Africa - Implications for Clinical Trials: A Decision Analysis | Not related to immunogenicity, efficacy, and safety of TB/Malaria vaccine candidate |
|  | Sauerzopf et al., | 2014 | In vitro growth of Plasmodium falciparum in neonatal blood | Not related to immunogenicity, efficacy, and safety of TB/Malaria vaccine candidate |
|  | Kawada et al., | 2014 | A Small-Scale Field Trial of Pyriproxyfen-Impregnated Bed Nets against Pyrethroid-Resistant Anopheles gambiae s.s. in Western Kenya | Not related to immunogenicity, efficacy, and safety of TB/Malaria vaccine candidate |
|  | Larsen et al., | 2014 | Community Coverage with Insecticide-Treated Mosquito Nets and Observed Associations with All-Cause Child Mortality and Malaria Parasite Infections | Not related to immunogenicity, efficacy, and safety of TB/Malaria vaccine candidate |
|  | Kawada et al., | 2014 | Preventive effect of permethrin-impregnated long-lasting insecticidal nets on the blood feeding of three major pyrethroid-resistant malaria vectors in western Kenya | Not related to immunogenicity, efficacy, and safety of TB/Malaria vaccine candidate |
|  | Sagara et al., | 2014 | Efficacy and safety of a combination of azithromycin and chloroquine for the treatment of uncomplicated Plasmodium falciparum malaria in two multi-country randomised clinical trials in African adults | Not related to immunogenicity, efficacy, and safety of TB/Malaria vaccine candidate |
|  | Sangoro et al., | 2014 | Feasibility of repellent use in a context of increasing outdoor transmission: a qualitative study in rural Tanzania | Not related to immunogenicity, efficacy, and safety of TB/Malaria vaccine candidate |
|  | Klement et al., | 2014 | Effectiveness of Co-trimoxazole to Prevent Plasmodium falciparum Malaria in HIV-Positive Pregnant Women in Sub-Saharan Africa: An Open-Label, Randomized Controlled Trial | Not related to immunogenicity, efficacy, and safety of TB/Malaria vaccine candidate |
|  | Kaneko et al., | 2014 | Characteristic Age Distribution of Plasmodium vivax Infections after Malaria Elimination on Aneityum Island, Vanuatu | Not related to immunogenicity, efficacy, and safety of TB/Malaria vaccine candidate |
|  | Zhao et al., | 2014 | PfRON3 is an erythrocyte-binding protein and a potential blood-stage vaccine candidate antigen |  |
|  | Phillips et al., | 2014 | Clinical and Bacteriological Efficacy of Rifampin-Streptomycin Combination for Two Weeks followed by Rifampin and Clarithromycin for Six Weeks for Treatment of Mycobacterium ulcerans Disease | Not related to immunogenicity, efficacy, and safety of TB/Malaria vaccine candidate |
|  | Sekandi et al., | 2014 | Yield of undetected tuberculosis and human immunodeficiency virus coinfection from active case finding in urban Uganda | Not related to immunogenicity, efficacy, and safety of TB/Malaria vaccine candidate |
|  | Gray et al., | 2014 | Isoniazid preventive therapy in HIV-infected children on antiretroviral therapy: a pilot study | Not related to immunogenicity, efficacy, and safety of TB/Malaria vaccine candidate |
|  | Leow et al., | 2014 | Production and characterization of specific monoclonal antibodies binding the Plasmodium falciparum diagnostic biomarker, histidine-rich protein 2 | Not related to immunogenicity, efficacy, and safety of TB/Malaria vaccine candidate |
|  | Rumisha et al., | 2014 | Relationship between child survival and malaria transmission: an analysis of the malaria transmission intensity and mortality burden across Africa (MTIMBA) project data in Rufiji demographic surveillance system, Tanzania | Not related to immunogenicity, efficacy, and safety of TB/Malaria vaccine candidate |
|  | Agossa et al., | 2014 | Efficacy of various insecticides recommended for indoor residual spraying: pirimiphos methyl, potential alternative to bendiocarb for pyrethroid resistance management in Benin, West Africa | Not related to immunogenicity, efficacy, and safety of TB/Malaria vaccine candidate |
|  | Bandeiras et al., | 2014 | Modeling Malaria Infection and Immunity against Variant Surface Antigens in Principe Island, West Africa | Not related to immunogenicity, efficacy, and safety of TB/Malaria vaccine candidate |
|  | Kufa et al., | 2014 | Incidence of HIV-Associated Tuberculosis among Individuals Taking Combination Antiretroviral Therapy: A Systematic Review and Meta-Analysis | Not related to immunogenicity, efficacy, and safety of TB/Malaria vaccine candidate |
|  | Alvarez-Uria et al., | 2014 | Incidence and mortality of tuberculosis before and after initiation of antiretroviral therapy: an HIV cohort study in India | Not related to immunogenicity, efficacy, and safety of TB/Malaria vaccine candidate |
|  | Fru-Cho et al., | 2014 | Molecular typing reveals substantial Plasmodium vivax infection in asymptomatic adults in a rural area of Cameroon | Not related to immunogenicity, efficacy, and safety of TB/Malaria vaccine candidate |
|  | Ogutu et al., | 2014 | Fixed dose artesunate amodiaquine - a phase IIb, randomized comparative trial with non-fixed artesunate amodiaquine | Not related to immunogenicity, efficacy, and safety of TB/Malaria vaccine candidate |
|  | Sikalaa et al., | 2014 | A cost-effective, community-based, mosquito-trapping scheme that captures spatial and temporal heterogeneities of malaria transmission in rural Zambia | Not related to immunogenicity, efficacy, and safety of TB/Malaria vaccine candidate |
|  | Peter et al., | 2014 | Sputum induction to aid diagnosis of smear-negative or sputum-scarce tuberculosis in adults in HIV-endemic settings | Not related to immunogenicity, efficacy, and safety of TB/Malaria vaccine candidate |
|  | Rahmna et al., | 2014 | Mycobacterium tuberculosis Subverts the TLR-2-MyD88 Pathway to Facilitate Its Translocation into the Cytosol | Not related to immunogenicity, efficacy, and safety of TB/Malaria vaccine candidate |
|  | Grobler et al., | 2014 | Assessment of the Induction of Dormant Ring Stages in Plasmodium falciparum Parasites by Artemisone and Artemisone Entrapped in Pheroid Vesicles In Vitro | Not related to immunogenicity, efficacy, and safety of TB/Malaria vaccine candidate |
|  | Telisinghe et al., | 2014 | High Tuberculosis Prevalence in a South African Prison: The Need for Routine Tuberculosis Screening | Not related to immunogenicity, efficacy, and safety of TB/Malaria vaccine candidate |
|  | Naranbhai et al., | 2014 | Ratio of Monocytes to Lymphocytes in Peripheral Blood Identifies Adults at Risk of Incident Tuberculosis Among HIV-Infected Adults Initiating Antiretroviral Therapy | Not related to immunogenicity, efficacy, and safety of TB/Malaria vaccine candidate |
|  | Ritcher et al., | 2014 | Economic Support to Patients in HIV and TB Grants in Rounds 7 and 10 from the Global Fund to Fight AIDS, Tuberculosis and Malaria | Not related to immunogenicity, efficacy, and safety of TB/Malaria vaccine candidate |
|  | Wu et al., | 2014 | Particle-based platforms for malaria vaccines | Not related to immunogenicity, efficacy, and safety of TB/Malaria vaccine candidate |
|  | Chimbatata et al., | 2017 | Post-2015, why delay to seek healthcare? Perceptions and field experiences from TB healthcare providers in northern Malawi: a qualitative study | Not related to immunogenicity, efficacy, and safety of TB/Malaria vaccine candidate |
|  | Mueller et al., | 2015 | Development of vaccines for Plasmodium vivax malaria | Not related to immunogenicity, efficacy, and safety of TB/Malaria vaccine candidate |
|  | Carpenter et al., | 2015 | A side-by-side comparison of T cell reactivity to fifty-nine Mycobacterium tuberculosis antigens in diverse populations from five continents | Not related to immunogenicity, efficacy, and safety of TB/Malaria vaccine candidate |
|  | Mothe et al., | 2015 | The TB-specific CD4(+) T cell immune repertoire in both cynomolgus and rhesus macaques largely overlap with humans | Not related to immunogenicity, efficacy, and safety of TB/Malaria vaccine candidate |
|  | Megnekou et al., | 2015 | Placental malaria and modulation of immune and hormonal responses in Cameroonian women | Not related to immunogenicity, efficacy, and safety of TB/Malaria vaccine candidate |
|  | Jaffer et al., | 2015 | Evaluating the sterilizing effect of pyriproxyfen treated mosquito nets against Anopheles gambiae at different blood-feeding intervals | Not related to immunogenicity, efficacy, and safety of TB/Malaria vaccine candidate |
|  | Richie et al., | 2015 | Design of a study to determine the impact of insecticide resistance on malaria vector control: a multi-country investigation | Not related to immunogenicity, efficacy, and safety of TB/Malaria vaccine candidate |
|  | Purbhoo et al., | 2015 | Efficacy of full-fat milk and diluted lemon juice in reducing infra-cardiac activity of 99mTc sestamibi during myocardial perfusion imaging | Not related to immunogenicity, efficacy, and safety of TB/Malaria vaccine candidate |
|  | Etheredge et al., | 2015 | Iron supplementation in iron-replete and nonanemic pregnant women in Tanzania: A randomized clinical trial | Not related to immunogenicity, efficacy, and safety of TB/Malaria vaccine candidate |
|  | Getahun et al., | 2015 | Managennent of latent Mycobacterium tuberculosis infection: WHO guidelines for Low tuberculosis burden countries | Not related to immunogenicity, efficacy, and safety of TB/Malaria vaccine candidate |
|  | Farnert et al., | 2015 | Duration of residency in a non-endemic area and risk of severe malaria in African immigrants | Not related to immunogenicity, efficacy, and safety of TB/Malaria vaccine candidate |
|  | Du toit et al., | 2015 | Randomized Trial of Peanut Consumption in Infants at Risk for Peanut Allergy | Not related to immunogenicity, efficacy, and safety of TB/Malaria vaccine candidate |
|  | Browne et al., | 2015 | Uterine Tonus Assessment by Midwives versus Patient self-assessment in the active management of the third stage of labor (UTAMP): Study protocol for a randomized controlled trial | Not related to immunogenicity, efficacy, and safety of TB/Malaria vaccine candidate |
|  | Madhi et al., | 2015 | Longitudinal study on Streptococcus pneumoniae, Haemophilus influenzae and Staphylococcus aureus nasopharyngeal colonization in HIV-infected and -uninfected infants vaccinated with pneumococcal conjugate vaccine | Not related to immunogenicity, efficacy, and safety of TB/Malaria vaccine candidate |
|  | Beksinska, et al., | 2015 | A randomized noninferiority crossover-controlled trial of the functional performance and safety of new female condoms: An evaluation of the Velvet, Cupid2, and FC2 | Not related to immunogenicity, efficacy, and safety of TB/Malaria vaccine candidate |
|  | Cutland et al., | 2015 | Lessons learnt from enrolment and follow up of pregnant women and their infants in clinical trials in South Africa, a low-middle income country | Not related to immunogenicity, efficacy, and safety of TB/Malaria vaccine candidate |
|  | Ratanasuwan et al., | 2015 | Peru-15 (Choleragarde®), a live attenuated oral cholera vaccine, is safe and immunogenic in human immunodeficiency virus (HIV)-seropositive adults in Thailand | Not related to immunogenicity, efficacy, and safety of TB/Malaria vaccine candidate |
|  | Achieng et al., | 2015 | Temporal trends in prevalence of Plasmodium falciparum molecular markers selected for by artemether-lumefantrine treatment in pre-ACT and post-ACT parasites in western Kenya | Not related to immunogenicity, efficacy, and safety of TB/Malaria vaccine candidate |
|  | Wilson et al., | 2015 | The C-Type Lectin Receptor CLECSF8/CLEC4D Is a Key Component of Anti-Mycobacterial Immunity | Not related to immunogenicity, efficacy, and safety of TB/Malaria vaccine candidate |
|  | Lawal et al., | 2015 | Effectiveness of a structured circuit class therapy model in stroke rehabilitation: A protocol for a randomised controlled trial | Not related to immunogenicity, efficacy, and safety of TB/Malaria vaccine candidate |
|  | Maughan-Brown et al., | 2015 | What Do People Actually Learn from Public Health Campaigns? Incorrect Inferences About Male Circumcision and Female HIV Infection Risk Among Men and Women in Malawi | Non-TB/malaria study |
|  | Ntozini et al., | 2015 | Using geographic information systems and spatial analysis methods to assess household water access and sanitation coverage in the SHINE Trial | Non-TB/malaria study |
|  | Tonguet-Papucci, et al., | 2015 | The MAM'Out project: A randomized controlled trial to assess multiannual and seasonal cash transfers for the prevention of acute malnutrition in children under 36 months in Burkina Faso | Non-TB/malaria study |
|  | Mantell et al., | 2015 | Promoting Female Condom Use Among Female University Students in KwaZulu-Natal, South Africa: Results of a Randomized Behavioral Trial | Non-TB/malaria study |
|  | Thomson et al., | 2015 | Laparoscopic versus open surgery for complicated appendicitis: a randomized controlled trial to prove safety | Non-TB/malaria study |
|  | Tiemersma et al., | 2015 | Delayed cord clamping in South African neonates with expected low birthweight: A randomised controlled trial | Non-TB/malaria study |
|  | Perez et al., | 2015 | Massage therapy improves the development of HIV-exposed infants living in a low socio-economic, peri-urban community of South Africa | Non-TB/malaria study |
|  | Kashuba et al., | 2015 | Genital Tenofovir Concentrations Correlate with Protection Against HIV Infection in the CAPRISA 004 Trial: Importance of Adherence for Microbicide Effectiveness | Non-TB/malaria study |
|  | Mavhu et al., | 2015 | Implementation and Operational Research: A Randomized Noninferiority Trial of AccuCirc Device Versus Mogen Clamp for Early Infant Male Circumcision in Zimbabwe | Non-TB/malaria study |
|  | Sagnon et al., | 2015 | To assess whether addition of pyriproxyfen to long-lasting insecticidal mosquito nets increases their durability compared to standard long-lasting insecticidal mosquito nets: Study protocol for a randomised controlled trial | Not related to immunogenicity, efficacy, and safety of TB/Malaria vaccine candidate |
|  | Sagnon et al., | 2015 | To assess whether addition of pyriproxyfen to long-lasting insecticidal mosquito nets increases their durability compared to standard long-lasting insecticidal mosquito nets: Study protocol for a randomised controlled trial | Not related to immunogenicity, efficacy, and safety of TB/Malaria vaccine candidate |
|  | Trehan et al., | 2015 | Common beans and cowpeas as complementary foods to reduce environmental enteric dysfunction and stunting in Malawian children: Study protocol for two randomized controlled trials | Not related to immunogenicity, efficacy, and safety of TB/Malaria vaccine candidate |
|  | Jemmott et al., | 2015 | Mediation of effects of a theory-based behavioral intervention on self-reported physical activity in South African men | Non-TB/malaria study |
|  | Lo et al., | 2015 | Managing Cancer and Living Meaningfully: Study protocol for a randomized controlled trial | Non-TB/malaria study |
|  | Palanee-Phillips et al., | 2015 | Characteristics of women enrolled into a randomized clinical trial of dapivirine vaginal ring for HIV-1 prevention | Non-TB/malaria study |
|  | Soliman et al., | 2015 | Baseline cardiovascular risk in the INSIGHT Strategic Timing of Antiretroviral Treatment (START) trial | Non-TB/malaria study |
|  | Siedner et al., | 2015 | Know your audience: Predictors of success for a patient-centered texting app to augment linkage to HIV care in rural Uganda | Non-TB/malaria study |
|  | Louwagie et al., | 2015 | Predictors of tobacco smoking abstinence among tuberculosis patients in South Africa | Non-TB/malaria study |
|  | Moodie et al., | 2015 | Continued follow-up of Phambili phase 2b randomized HIV-1 vaccine trial participants support increased HIV-1 acquisition among vaccinated men | Non-TB/malaria study |
|  | Magaret et al., | 2015 | Effect of Condom Use on Per-act HSV-2 Transmission Risk in HIV-1, HSV-2-discordant Couples | Non-TB/malaria study |
|  | Giuliano et al., | 2015 | High HIV, HPV, and STI prevalence among young Western Cape, South African women: EVRI HIV prevention preparedness trial | Non-TB/malaria study |
|  | Alhassan et al., | 2015 | Effect of community engagement interventions on patient safety and risk reduction efforts in primary health facilities: Evidence from Ghana | Non-TB/malaria study |
|  | Garrett et al., | 2015 | HIV disease progression in seroconvertors from the CAPRISA 004 tenofovir gel pre-exposure prophylaxis trial | Non-TB/malaria study |
|  | Pattacini et al., | 2015 | Antiretroviral pre-exposure prophylaxis does not enhance immune responses to HIV in exposed but uninfected persons | Non-TB/malaria study |
|  | Diallo et al., | 2015 | Antibody persistence at 1 and 4 years following a single dose of MenAfriVac or Quadrivalent polysaccharide vaccine in healthy subjects aged 2-29 years | Non-TB/malaria study |
|  | Ruff et al., | 2015 | Time course of safety and efficacy of aflibercept in combination with FOLFIRI in patients with metastatic colorectal cancer who progressed on previous oxaliplatin-based therapy | Non-TB/malaria study |
|  | Omosa-Manyonyi et al., | 2015 | A phase I double blind, placebo-controlled, randomized study of the safety and immunogenicity of an adjuvanted HIV-1 Gag-Pol-Nef fusion protein and adenovirus 35 Gag-RT-Int-Nef vaccine in healthy HIV-uninfected African adults | Non-TB/malaria study |
|  | Pengpid et al., | 2015 | Screening and concurrent brief intervention of conjoint hazardous or harmful alcohol and tobacco use in hospital out-patients in Thailand: A randomized controlled trial | Non-TB/malaria study |
|  | Apter et al., | 2015 | Efficacy of human papillomavirus 16 and 18 (HPV-16/18) AS04-adjuvanted vaccine against cervical infection and precancer in young women: Final event-driven analysis of the randomized, double-blind PATRICIA trial | Non-TB/malaria study |
|  | Agnandji et al., | 2015 | Clinical development of RTS, S/AS malaria vaccine: a systematic review of clinical Phase I-III trials | Not related to immunogenicity, efficacy, and safety of TB/Malaria vaccine candidate |
|  | Esterhuyse et al., | 2015 | Epigenetics and Proteomics Join Transcriptomics in the Quest for Tuberculosis Biomarkers | Not related to immunogenicity, efficacy, and safety of TB/Malaria vaccine candidate |
|  | Ewing et al., | 2015 | Perceptions and utilization of the anti-malarials artemether-lumefantrine and dihydroartemisininpiperaquine in young children in the Chikhwawa District of Malawi: a mixed methods study | Not related to immunogenicity, efficacy, and safety of TB/Malaria vaccine candidate |
|  | Denti et al., | 2015 | Pharmacokinetics of Isoniazid, Pyrazinamide, and Ethambutol in Newly Diagnosed Pulmonary TB Patients in Tanzania | Not related to immunogenicity, efficacy, and safety of TB/Malaria vaccine candidate |
|  | Kwenda et al., | 2015 | Molecular characterisation of clinical and environmental isolates of Mycobacterium kansasii isolates from South African gold mines | Not related to immunogenicity, efficacy, and safety of TB/Malaria vaccine candidate |
|  | Amaokwa et al., | 2015 | Risk Factors for Developing Active Tuberculosis After the Treatment of Latent Tuberculosis in Adults Infected with Human Immunodeficiency Virus | Not related to immunogenicity, efficacy, and safety of TB/Malaria vaccine candidate |
|  | Drame et al., | 2015 | Specific antibodies to Anopheles gSG6-P1 salivary peptide to assess early childhood exposure to malaria vector bites | Not related to immunogenicity, efficacy, and safety of TB/Malaria vaccine candidate |
|  | Kagina et al., | 2015 | Qualification of a whole blood intracellular cytokine staining assay to measure mycobacteria-specific CD4 and CD8 T cell immunity by flow cytometry | Not related to immunogenicity, efficacy, and safety of TB/Malaria vaccine candidate |
|  | Cabrera-Mora et al., | 2015 | Induction of Multifunctional Broadly Reactive T Cell Responses by a Plasmodium vivax Circumsporozoite Protein Recombinant Chimera | Pre-clinical study |
|  | Braun et al., | 2015 | Lack of effect of intermittent preventive treatment for malaria in pregnancy and intense drug resistance in western Uganda | Not related to immunogenicity, efficacy, and safety of TB/Malaria vaccine candidate |
|  | Orlov et al., | 2015 | Presence of Plasmodium falciparum DNA in Plasma Does Not Predict Clinical Malaria in an HIV-1 Infected Population | Not related to immunogenicity, efficacy, and safety of TB/Malaria vaccine candidate |
|  | Chetty et al., | 2015 | Co-Infection with Mycobacterium tuberculosis Impairs HIV-Specific CD8+and CD4+T Cell Functionality | Not related to immunogenicity, efficacy, and safety of TB/Malaria vaccine candidate |
|  | Kalokhe et al., | 2015 | Impaired Degranulation and Proliferative Capacity of Mycobacterium tuberculosis-Specific CD8(+) T Cells in HIV-Infected Individuals With Latent Tuberculosis | Not related to immunogenicity, efficacy, and safety of TB/Malaria vaccine candidate |
|  | Edwards et al., | 2015 | Coinfection with Blood-Stage Plasmodium Promotes Systemic Type I Interferon Production during Pneumovirus Infection but Impairs Inflammation and Viral Control in the Lung | Not related to immunogenicity, efficacy, and safety of TB/Malaria vaccine candidate |
|  | Oxborough et al., | 2015 | The activity of the pyrrole insecticide chlorfenapyr in mosquito bioassay: towards a more rational testing and screening of non-neurotoxic insecticides for malaria vector control | Not related to immunogenicity, efficacy, and safety of TB/Malaria vaccine candidate |
|  | Grynberg et al., | 2015 | Artemether-Lumefantrine Compared to Atovaquone-Proguanil as a Treatment for Uncomplicated Plasmodium falciparum Malaria in Travelers | Not related to immunogenicity, efficacy, and safety of TB/Malaria vaccine candidate |
|  | Amogne et al., | 2015 | Efficacy and Safety of Antiretroviral Therapy Initiated One Week after Tuberculosis Therapy in Patients with CD4 Counts < 200 Cells/mu L: TB-HAART Study, a Randomized Clinical Trial | Not related to immunogenicity, efficacy, and safety of TB/Malaria vaccine candidate |
|  | Mita | 2015 | Patterns and dynamics of genetic diversity in Plasmodium falciparum: What past human migrations tell us about malaria | Not related to immunogenicity, efficacy, and safety of TB/Malaria vaccine candidate |
|  | Wotodjo et al., | 2015 | Malaria Risk Factors in Dielmo, A Senegalese Malaria-Endemic Village, Between October and November of 2013: A Case-Control Study | Not related to immunogenicity, efficacy, and safety of TB/Malaria vaccine candidate |
|  | Shelton et al., | 2015 | Genetic determinants of anti-malarial acquired immunity in a large multi-centre study | Not related to immunogenicity, efficacy, and safety of TB/Malaria vaccine candidate |
|  | Nunes-Elves et al., | 2015 | Human and Murine Clonal CD8+T Cell Expansions Arise during Tuberculosis  Because of TCR Selection | Not related to immunogenicity, efficacy, and safety of TB/Malaria vaccine candidate |
|  | Tagbor et al., | 2015 | A Non-Inferiority, Individually Randomized Trial of Intermittent Screening and Treatment versus Intermittent Preventive Treatment in the Control of Malaria in Pregnancy | Not related to immunogenicity, efficacy, and safety of TB/Malaria vaccine candidate |
|  | Gadalla et al., | 2015 | Prevalence of Plasmodium falciparum anti-malarial resistance-associated polymorphisms in pfcrt, pfmdr1 and pfnhe1 in Muheza, Tanzania, prior to introduction of artemisinin combination therapy | Not related to immunogenicity, efficacy, and safety of TB/Malaria vaccine candidate |
|  | Tay et al., | 2015 | The prevalence of malaria among HIV seropositive individuals and the impact of the co-infection on their hemoglobin levels | Not related to immunogenicity, efficacy, and safety of TB/Malaria vaccine candidate |
|  | Chandra et al., | 2015 | Comparison of azithromycin plus chloroquine versus artemether-lumefantrine for the treatment of uncomplicated Plasmodium falciparum malaria in children in Africa: a randomized, open-label study | Not related to immunogenicity, efficacy, and safety of TB/Malaria vaccine candidate |
|  | Boeree et al., | 2015 | A Dose-Ranging Trial to Optimize the Dose of Rifampin in the Treatment of Tuberculosis | Not related to immunogenicity, efficacy, and safety of TB/Malaria vaccine candidate |
|  | Visser et al., | 2015 | Host Immune Response to Tuberculous Meningitis | Not related to immunogenicity, efficacy, and safety of TB/Malaria vaccine candidate |
|  | Ngane et al., | 2015 | Molecular epidemiology of drug-resistant Plasmodium falciparum in Benguela province, Angola | Not related to immunogenicity, efficacy, and safety of TB/Malaria vaccine candidate |
|  | Nwabor | 2015 | Anopheline Mosquitoes and the Malaria Scourge | Not related to immunogenicity, efficacy, and safety of TB/Malaria vaccine candidate |
|  | Dow et al., | 2015 | Summary of anti-malarial prophylactic efficacy of tafenoquine from three placebo-controlled studies of residents of malaria-endemic countries | Not related to immunogenicity, efficacy, and safety of TB/Malaria vaccine candidate |
|  | Oxborough et al., | 2015 | A new class of insecticide for malaria vector control: evaluation of mosquito nets treated singly with indoxacarb (oxadiazine) or with a pyrethroid mixture against Anopheles gambiae and Culex quinquefasciatus | Not related to immunogenicity, efficacy, and safety of TB/Malaria vaccine candidate |
|  | McCarthy et al., | 2015 | Calibration of an intrahost malaria model and parameter ensemble evaluation of a pre-erythrocytic vaccine | Not related to immunogenicity, efficacy, and safety of TB/Malaria vaccine candidate |
|  | Coulibaly et al., | 2015 | Efficacy and Safety of Triple Combination Therapy with Artesunate-Amodiaquine-Methylene Blue for Falciparum Malaria in Children: A Randomized Controlled Trial in Burkina Faso | Not related to immunogenicity, efficacy, and safety of TB/Malaria vaccine candidate |
|  | Riou et al., | 2015 | Restoration of CD4(+) Responses to Copathogens in HIV-Infected Individuals on Antiretroviral Therapy Is Dependent on T Cell Memory Phenotype | Not related to immunogenicity, efficacy, and safety of TB/Malaria vaccine candidate |
|  | Schats et al., | 2015 | Heterologous Protection against Malaria after Immunization with Plasmodium falciparum Sporozoites | Non-sub-Saharan African study |
|  | Kapisi et al., | 2015 | Efficacy and safety of artemether-lumefantrine for the treatment of uncomplicated malaria in the setting of three different chemopreventive regimens | Not related to immunogenicity, efficacy, and safety of TB/Malaria vaccine candidate |
|  | Abay et al., | 2015 | Efficacy and pharmacokinetic evaluation of a novel anti-malarial compound (NP046) in a mouse model | Not related to immunogenicity, efficacy, and safety of TB/Malaria vaccine candidate |
|  | Gupta et al., | 2015 | Does antiretroviral therapy reduce HIV-associated tuberculosis incidence to background rates? A national observational cohort study from England, Wales, and Northern Ireland | Not related to immunogenicity, efficacy, and safety of TB/Malaria vaccine candidate |
|  | Okoyo et al., | 2015 | Comparing insecticide-treated bed net use to Plasmodium falciparum infection among schoolchildren living near Lake Victoria, Kenya | Not related to immunogenicity, efficacy, and safety of TB/Malaria vaccine candidate |
|  | Golub et al., | 2015 | Long-term Protection from Isoniazid Preventive Therapy for Tuberculosis in HIV-Infected Patients in a Medium-Burden Tuberculosis Setting: The TB/HIV in Rio (THRio) Study | Not related to immunogenicity, efficacy, and safety of TB/Malaria vaccine candidate |
|  | Mohr et al., | 2015 | Programmatic treatment outcomes in HIV-infected and uninfected drug-resistant TB patients in Khayelitsha, South Africa | Not related to immunogenicity, efficacy, and safety of TB/Malaria vaccine candidate |
|  | Przybylska et al., | 2015 | Detection of the Quarantine Species Thrips palmi by Loop-Mediated Isothermal Amplification | Non-TB/malaria study |
|  | Peters et al., | 2015 | Integration of TB-HIV services at an ANC facility in Frances Baard District, Northern Cape, South Africa | Not related to immunogenicity, efficacy, and safety of TB/Malaria vaccine candidate |
|  | Ndjeka et al., | 2015 | Treatment of drug-resistant tuberculosis with bedaquiline in a high HIV prevalence setting: an interim cohort analysis | Not related to immunogenicity, efficacy, and safety of TB/Malaria vaccine candidate |
|  | Torrado et al., | 2015 | Interleukin 27R regulates CD4(+) T cell phenotype and impacts protective immunity during Mycobacterium tuberculosis infection | Not related to immunogenicity, efficacy, and safety of TB/Malaria vaccine candidate |
|  | Quattarra et al., | 2015 | Polymorphisms in the K13-Propeller Gene in Artemisinin-Susceptible Plasmodium falciparum Parasites from Bougoula-Hameau and Bandiagara, Mali | Not related to immunogenicity, efficacy, and safety of TB/Malaria vaccine candidate |
|  | Dambuza et al., | 2015 | Antiplasmodial activity, in vivo pharmacokinetics, and anti-malarial efficacy evaluation of hydroxypyridinone hybrids in a mouse model | Not related to immunogenicity, efficacy, and safety of TB/Malaria vaccine candidate |
|  | Murray et al., | 2015 | Accelerating clinical drug development for children with tuberculosis | Not related to immunogenicity, efficacy, and safety of TB/Malaria vaccine candidate |
|  | Wanjala et al., | 2015 | Insecticidal decay effects of long-lasting insecticide nets and indoor residual spraying on Anopheles gambiae and Anopheles arabiensis in Western Kenya | Not related to immunogenicity, efficacy, and safety of TB/Malaria vaccine candidate |
|  | Apinjoh et al., | 2015 | Determinants of Infant Susceptibility to Malaria During the First Year of Life in Southwestern Cameroon | Not related to immunogenicity, efficacy, and safety of TB/Malaria vaccine candidate |
|  | Kruger et al., | 2015 | Acceptability and effectiveness of a monofilament, polyethylene insecticide-treated wall lining for malaria control after six months in dwellings in Vhembe District, Limpopo Province, South Africa | Not related to immunogenicity, efficacy, and safety of TB/Malaria vaccine candidate |
|  | Bachmann et al., | 2015 | Effect of antiretroviral treatment on the risk of tuberculosis during South Africa's programme expansion | Not related to immunogenicity, efficacy, and safety of TB/Malaria vaccine candidate |
|  | Guler et al., | 2015 | IL-4R alpha-Dependent Alternative Activation of Macrophages Is Not Decisive for Mycobacterium tuberculosis Pathology and Bacterial Burden in Mice | Not related to immunogenicity, efficacy, and safety of TB/Malaria vaccine candidate |
|  | Francisco et al., | 2015 | TNF-dependent regulation and activation of innate immune cells are essential for host protection against cerebral tuberculosis | Not related to immunogenicity, efficacy, and safety of TB/Malaria vaccine candidate |
|  | Dawson et al., | 2015 | Two-stage activity-safety study of daily rifapentine during intensive phase treatment of pulmonary tuberculosis | Not related to immunogenicity, efficacy, and safety of TB/Malaria vaccine candidate |
|  | Siame et al., | 2015 | High prevalence of dhfr and dhps molecular markers in Plasmodium falciparum in pregnant women of Nchelenge district, Northern Zambia | Not related to immunogenicity, efficacy, and safety of TB/Malaria vaccine candidate |
|  | Daniels et al., | 2015 | Time to ART Initiation among Patients Treated for Rifampicin-Resistant Tuberculosis in Khayelitsha, South Africa: Impact on Mortality and Treatment Success | Not related to immunogenicity, efficacy, and safety of TB/Malaria vaccine candidate |
|  | Thee et al., | 2015 | Pharmacokinetics and Safety of Moxifloxacin in Children with Multidrug-Resistant Tuberculosis | Not related to immunogenicity, efficacy, and safety of TB/Malaria vaccine candidate |
|  | Van Oosterhout et al., | 2015 | Pharmacokinetics of Antituberculosis Drugs in HIV-Positive and HIV-Negative Adults in Malawi | Not related to immunogenicity, efficacy, and safety of TB/Malaria vaccine candidate |
|  | Sicuri et al., | 2015 | Economic Evaluation of an Alternative Drug to Sulfadoxine-Pyrimethamine as Intermittent Preventive Treatment of Malaria in Pregnancy | Not related to immunogenicity, efficacy, and safety of TB/Malaria vaccine candidate |
|  | Akono et al., | 2015 | Impact of vegetable crop agriculture on anopheline agressivity and malaria transmission in urban and less urbanized settings of the South region of Cameroon | Not related to immunogenicity, efficacy, and safety of TB/Malaria vaccine candidate |
|  | Abokyi et al., | 2015 | Use of Antimalarial in the Management of Fever during a Community Survey in the Kintampo Districts of Ghana | Not related to immunogenicity, efficacy, and safety of TB/Malaria vaccine candidate |
|  | Gordon et al., | 2015 | Inhibiting the Mammalian Target of Rapamycin Blocks the Development of Experimental Cerebral Malaria | Not related to immunogenicity, efficacy, and safety of TB/Malaria vaccine candidate |
|  | Barnard et al., | 2015 | The utility of Xpert MTB/RIF performed on bronchial washings obtained in patients with suspected pulmonary tuberculosis in a high prevalence setting | Not related to immunogenicity, efficacy, and safety of TB/Malaria vaccine candidate |
|  | Chico et al., | 2015 | Global Call to Action: maximize the public health impact of intermittent preventive treatment of malaria in pregnancy in sub-Saharan Africa | Not related to immunogenicity, efficacy, and safety of TB/Malaria vaccine candidate |
|  | Mathema et al., | 2015 | Molecular Epidemiology of Mycobacterium tuberculosis among South African  Gold Miners | Not related to immunogenicity, efficacy, and safety of TB/Malaria vaccine candidate |
|  | Andrejak et al., | 2015 | Characterization of Mouse Models of Mycobacterium avium Complex Infection and Evaluation of Drug Combinations | Not related to immunogenicity, efficacy, and safety of TB/Malaria vaccine candidate |
|  | Henao-Tamayo et al., | 2015 | The Efficacy of the BCG Vaccine against Newly Emerging Clinical Strains of Mycobacterium tuberculosis | Not related to immunogenicity, efficacy, and safety of TB/Malaria vaccine candidate |
|  | Hermans et al., | 2015 | Temporal trends in TB notification rates during ART scale-up in Cape Town: an ecological analysis | Not related to immunogenicity, efficacy, and safety of TB/Malaria vaccine candidate |
|  | Mangano et al., | 2015 | Novel Insights into the Protective Role of Hemoglobin S and C Against Plasmodium falciparum Parasitemia | Not related to immunogenicity, efficacy, and safety of TB/Malaria vaccine candidate |
|  | Mbah et al., | 2015 | Comparing the Impact of Artemisinin-Based Combination Therapies on Malaria Transmission in Sub-Saharan Africa | Not related to immunogenicity, efficacy, and safety of TB/Malaria vaccine candidate |
|  | Gilbert et al., | 2015 | Integrating Community-Based Interventions to Reverse the Convergent TB/HIV Epidemics in Rural South Africa | Not related to immunogenicity, efficacy, and safety of TB/Malaria vaccine candidate |
|  | Maka et al., | 2015 | A randomized trial of the efficacy of artesunate and three quinine regimens in the treatment of severe malaria in children at the Ebolowa Regional Hospital, Cameroon | Not related to immunogenicity, efficacy, and safety of TB/Malaria vaccine candidate |
|  | Adelman et al., | 2015 | Intensified tuberculosis case finding among HIV-infected persons using a WHO symptom screen and Xpert (R) MTB/RIF | Not related to immunogenicity, efficacy, and safety of TB/Malaria vaccine candidate |
|  | Ilin et al., | 2015 | Complete Genome Sequence of Multidrug-Resistant Clinical Isolate Mycobacterium tuberculosis 187.0, Used to Study the Effect of Drug Susceptibility Reversion by the New Medicinal Drug FS-1 | Not related to immunogenicity, efficacy, and safety of TB/Malaria vaccine candidate |
|  | Subbian et al., | 2015 | Lesion-Specific Immune Response in Granulomas of Patients with Pulmonary Tuberculosis: A Pilot Study | Not related to immunogenicity, efficacy, and safety of TB/Malaria vaccine candidate |
|  | Van Griensven et al., | 2015 | Implementation of isoniazid preventive therapy in an HIV clinic in Cambodia: high rates of discontinuation when combined with antiretroviral therapy | Not related to immunogenicity, efficacy, and safety of TB/Malaria vaccine candidate |
|  | Rullas et al., | 2015 | Combinations of beta-Lactam Antibiotics Currently in Clinical Trials Are Efficacious in a DHP-I-Deficient Mouse Model of Tuberculosis Infection | Not related to immunogenicity, efficacy, and safety of TB/Malaria vaccine candidate |
|  | Lai et al., | 2015 | HIV-tuberculosis-associated immune reconstitution inflammatory syndrome is characterized by Toll-like receptor and inflammasome signalling | Not related to immunogenicity, efficacy, and safety of TB/Malaria vaccine candidate |
|  | Isozumi et al., | 2015 | Improved detection of malaria cases in island settings of Vanuatu and Kenya by PCR that targets the Plasmodium mitochondrial cytochrome c oxidase III (cox3) gene | Not related to immunogenicity, efficacy, and safety of TB/Malaria vaccine candidate |
|  | mcNerney et al., | 2015 | Regulatory In Vitro Diagnostics Landscape in Africa: Update on Regional Activities | Not related to immunogenicity, efficacy, and safety of TB/Malaria vaccine candidate |
|  | Kaewseekhao et al., | 2015 | Comparative Proteomics of Activated THP-1 Cells Infected with Mycobacterium tuberculosis Identifies Putative Clearance Biomarkers for Tuberculosis Treatment | Not related to immunogenicity, efficacy, and safety of TB/Malaria vaccine candidate |
|  | Band et al., | 2015 | A novel locus of resistance to severe malaria in a region of ancient balancing selection | Not related to immunogenicity, efficacy, and safety of TB/Malaria vaccine candidate |
|  | Chen et al., | 2015 | An assessment of the supply, programmatic use, and regulatory issues of single low-dose primaquine as a Plasmodium falciparum gametocytocide for sub-Saharan Africa | Not related to immunogenicity, efficacy, and safety of TB/Malaria vaccine candidate |
|  | Erik Wikman-Jorgensen, et al., | 2015 | Implementation challenges of a TB programme in rural northern mozambique: evaluation of 2012-2013 outcomes | Not related to immunogenicity, efficacy, and safety of TB/Malaria vaccine candidate |
|  | Saunders et al., | 2015 | Safety, Tolerability, and Compliance with Long-Term Antimalarial Chemoprophylaxis in American Soldiers in Afghanistan | Not related to immunogenicity, efficacy, and safety of TB/Malaria vaccine candidate |
|  | Datta et al., | 2015 | Anti-vascular endothelial growth factor treatment normalizes tuberculosis granuloma vasculature and improves small molecule delivery | Not related to immunogenicity, efficacy, and safety of TB/Malaria vaccine candidate |
|  | Garcia-Prats et al., | 2015 | Pharmacokinetics and Safety of Ofloxacin in Children with Drug-Resistant Tuberculosis | Not related to immunogenicity, efficacy, and safety of TB/Malaria vaccine candidate |
|  | Ramma et al., | 2015 | Patients' costs associated with seeking and accessing treatment for drug-resistant tuberculosis in South Africa | Not related to immunogenicity, efficacy, and safety of TB/Malaria vaccine candidate |
|  | Zionecker et al., | 2015 | The V Gene Repertoires of Classical and Atypical Memory B Cells in Malaria-Susceptible West African Children | Not related to immunogenicity, efficacy, and safety of TB/Malaria vaccine candidate |
|  | Musoke et al., | 2015 | Promising Perceptions, Divergent Practices and Barriers to Integrated Malaria Prevention in Wakiso District, Uganda: A Mixed Methods Study | Not related to immunogenicity, efficacy, and safety of TB/Malaria vaccine candidate |
|  | Umanah et al., | 2015 | Predictors of cure among HIV co-infected multidrug-resistant TB patients at Sizwe Tropical Disease Hospital Johannesburg, South Africa | Not related to immunogenicity, efficacy, and safety of TB/Malaria vaccine candidate |
|  | Reddy et al., | 2015 | Patterns of Lymph Node Pathology; Fine Needle Aspiration Biopsy as an Evaluation Tool for Lymphadenopathy: A Retrospective Descriptive Study Conducted at the Largest Hospital in Africa | Not related to immunogenicity, efficacy, and safety of TB/Malaria vaccine candidate |
|  | Huang et al., | 2015 | A Single Mutation in K13 Predominates in Southern China and is Associated with Delayed Clearance of Plasmodium falciparum Following Artemisinin Treatment | Not related to immunogenicity, efficacy, and safety of TB/Malaria vaccine candidate |
|  | Goovaerts et al., | 2015 | Lower Pre-Treatment T Cell Activation in Early- and Late-Onset Tuberculosis-Associated Immune Reconstitution Inflammatory Syndrome | Not related to immunogenicity, efficacy, and safety of TB/Malaria vaccine candidate |
|  | Mcllleron et al., | 2015 | Special Populations and Pharmacogenetic Issues in Tuberculosis Drug Development and Clinical Research | Not related to immunogenicity, efficacy, and safety of TB/Malaria vaccine candidate |
|  | Tyagi et al., | 2015 | Clofazimine shortens the duration of the first-line treatment regimen for experimental chemotherapy of tuberculosis | Not related to immunogenicity, efficacy, and safety of TB/Malaria vaccine candidate |
|  | Kukula et al., | 2015 | Feasibility and cost of using mobile phones for capturing drug safety information in peri-urban settlement in Ghana: a prospective cohort study of patients with uncomplicated malaria | Not related to immunogenicity, efficacy, and safety of TB/Malaria vaccine candidate |
|  | Cummings et al., | 2015 | Inverting the pyramid: increasing awareness of mycobacterial sepsis in sub-Saharan Africa | Not related to immunogenicity, efficacy, and safety of TB/Malaria vaccine candidate |
|  | Ndour et al., | 2015 | Plasmodium falciparum Clearance Is Rapid and Pitting Independent in Immune Malian Children Treated with Artesunate for Malaria | Not related to immunogenicity, efficacy, and safety of TB/Malaria vaccine candidate |
|  | Glunt et al., | 2015 | Long-lasting insecticidal nets no longer effectively kill the highly resistant Anopheles funestus of southern Mozambique | Not related to immunogenicity, efficacy, and safety of TB/Malaria vaccine candidate |
|  | Wilkinson et al., | 2015 | Cytotoxic Mediators in Paradoxical HIV-Tuberculosis Immune Reconstitution Inflammatory Syndrome | Not related to immunogenicity, efficacy, and safety of TB/Malaria vaccine candidate |
|  | Lobo et al., | 2015 | Unexpected diversity of Anopheles species in Eastern Zambia: implications for evaluating vector behavior and interventions using molecular tools | Not related to immunogenicity, efficacy, and safety of TB/Malaria vaccine candidate |
|  | Wotodjo et al., | 2015 | The implication of long-lasting insecticide-treated net use in the resurgence of malaria morbidity in a Senegal malaria endemic village in 2010-2011 | Not related to immunogenicity, efficacy, and safety of TB/Malaria vaccine candidate |
|  | Corbett et al., | 2015 | Involvement of Nod2 in the innate immune response elicited by malarial pigment hemozoin | Not related to immunogenicity, efficacy, and safety of TB/Malaria vaccine candidate |
|  | Mze et al., | 2015 | RDTs as a source of DNA to study Plasmodium falciparum drug resistance in isolates from Senegal and the Comoros Islands |  |
|  | Gumbo et al., | 2015 | Nonclinical Models for Antituberculosis Drug Development: A Landscape Analysis | Not related to immunogenicity, efficacy, and safety of TB/Malaria vaccine candidate |
|  | Lindblade et al., | 2015 | A cohort study of the effectiveness of insecticide-treated bed nets to prevent malaria in an area of moderate pyrethroid resistance, Malawi | Not related to immunogenicity, efficacy, and safety of TB/Malaria vaccine candidate |
|  | Sedda et al., | 2015 | A geostatistical analysis of the association between armed conflicts and Plasmodium falciparum malaria in Africa, 1997-2010 | Not related to immunogenicity, efficacy, and safety of TB/Malaria vaccine candidate |
|  | Lindblade et al., | 2015 | Antiretroviral treatment among co-infected tuberculosis patients in integrated and non-integrated facilities | Not related to immunogenicity, efficacy, and safety of TB/Malaria vaccine candidate |
|  | Mbonye et al., | 2015 | Prevalence of Plasmodium falciparum Resistance Markers to Sulfadoxine-Pyrimethamine among Pregnant Women Receiving Intermittent Preventive Treatment for Malaria in Uganda | Not related to immunogenicity, efficacy, and safety of TB/Malaria vaccine candidate |
|  | ElMaraachli et al., | 2015 | Predicting differential rifamycin resistance in clinical Mycobacterium tuberculosis isolates by specific rpoB mutations | Not related to immunogenicity, efficacy, and safety of TB/Malaria vaccine candidate |
|  | Cairns et al., | 2015 | Seasonality in malaria transmission: implications for case-management with long-acting artemisinin combination therapy in sub-Saharan Africa | Not related to immunogenicity, efficacy, and safety of TB/Malaria vaccine candidate |
|  | Gumbo et al., | 2015 | Correlations Between the Hollow Fiber Model of Tuberculosis and Therapeutic Events in Tuberculosis Patients: Learn and Confirm | Not related to immunogenicity, efficacy, and safety of TB/Malaria vaccine candidate |
|  | O’Neill et al., | 2015 | Foul wind, spirits and witchcraft: illness conceptions and health-seeking behaviour for malaria in the Gambia | Not related to immunogenicity, efficacy, and safety of TB/Malaria vaccine candidate |
|  | Srivastava et al., | 2015 | Rapid Drug Tolerance and Dramatic Sterilizing Effect of Moxifloxacin Monotherapy in a Novel Hollow-Fiber Model of Intracellular Mycobacterium kansasii Disease | Not related to immunogenicity, efficacy, and safety of TB/Malaria vaccine candidate |
|  | Cheriff et al., | 2015 | Is Fc gamma receptor IIA (Fc gamma RIIA) polymorphism associated with clinical malaria and Plasmodium falciparum specific antibody levels in children from Burkina Faso? | Not related to immunogenicity, efficacy, and safety of TB/Malaria vaccine candidate |
|  | Mnzava et al., | 2015 | Implementation of the global plan for insecticide resistance management in malaria vectors: progress, challenges, and the way forward | Not related to immunogenicity, efficacy, and safety of TB/Malaria vaccine candidate |
|  | Davis et al., | 2015 | Impact of Daily Cotrimoxazole on Clinical Malaria and Asymptomatic Parasitemias in HIV-Exposed, Uninfected Infants | Not related to immunogenicity, efficacy, and safety of TB/Malaria vaccine candidate |
|  | Do Sambo et al., | 2015 | Quantitative trait locus analysis of parasite density reveals that HbS gene carriage protects severe malaria patients against Plasmodium falciparum hyperparasitaemia | Not related to immunogenicity, efficacy, and safety of TB/Malaria vaccine candidate |
|  | Ndounga et al., | 2015 | Artesunate-amodiaquine versus artemether-lumefantrine for the treatment of acute uncomplicated malaria in Congolese children under 10 years old living in a suburban area: a randomized study | Not related to immunogenicity, efficacy, and safety of TB/Malaria vaccine candidate |
|  | Bisoff et al., | 2015 | Early hyperreactive malarial splenomegaly and risk factors for evolution into the full-blown syndrome: a single-centre, retrospective, longitudinal study | Not related to immunogenicity, efficacy, and safety of TB/Malaria vaccine candidate |
|  | Zhu et al., | 2015 | Modelling optimum use of attractive toxic sugar bait stations for effective malaria vector control in Africa | Not related to immunogenicity, efficacy, and safety of TB/Malaria vaccine candidate |
|  | Wumba et al., | 2015 | Interactions between malaria and HIV infections in pregnant women: a first report of the magnitude, clinical and laboratory features, and predictive factors in Kinshasa, the Democratic Republic of Congo | Not related to immunogenicity, efficacy, and safety of TB/Malaria vaccine candidate |
|  | Mekonnen et al., | 2015 | Efficacy of artemether-lumefantrine therapy for the treatment of uncomplicated Plasmodium falciparum malaria in Southwestern Ethiopia | Not related to immunogenicity, efficacy, and safety of TB/Malaria vaccine candidate |
|  | Pfeil et al., | 2015 | An Economic Evaluation of the Posttreatment Prophylactic Effect of Dihydroartemisinin-Piperaquine versus Artemether-Lumefantrine for First-Line Treatment of Plasmodium falciparum Malaria across Different Transmission Settings in Africa | Not related to immunogenicity, efficacy, and safety of TB/Malaria vaccine candidate |
|  | Randriamaherijaona, et al., | 2015 | Do holes in long-lasting insecticidal nets compromise their efficacy against pyrethroid resistant Anopheles gambiae and Culex quinquefasciatus? Results from a release-recapture study in experimental huts | Not related to immunogenicity, efficacy, and safety of TB/Malaria vaccine candidate |
|  | Wallis et al., | 2015 | Advancing host-directed therapy for tuberculosis | Not related to immunogenicity, efficacy, and safety of TB/Malaria vaccine candidate |
|  | Kassa et al., | 2016 | The effect of HIV coinfection, HAART and TB treatment on cytokine/chemokine responses to Mycobacterium tuberculosis (Mtb) antigens in active TB patients and latently Mtb infected individuals | Not related to immunogenicity, efficacy, and safety of TB/Malaria vaccine candidate |
|  | Ginsberg et al., | 2016 | TB vaccines in clinical development | Not related to immunogenicity, efficacy, and safety of TB/Malaria vaccine candidate |
|  | Riou et al., | 2016 | Selective reduction of IFN-gamma single positive mycobacteria-specific CD4+T cells in HIV-1 infected individuals with latent tuberculosis infection | Not related to immunogenicity, efficacy, and safety of TB/Malaria vaccine candidate |
|  | Roos et al., | 2016 | Test performance of three serological assays for the detection of Mycobacterium bovis infection in common warthogs (Phacochoerus africanus) | Non-clinical vaccination study |
|  | Spearman et al., | 2016 | A phase 1, randomized, controlled dose-escalation study of EP-1300 polyepitope DNA vaccine against Plasmodium falciparum malaria administered via electroporation | Non-sub-Saharan African vaccination study |
|  | Ritz et al., | 2016 | Comparable CD4 and CD8 T cell responses and cytokine release after at-birth and delayed BCG immunisation in infants born in Australia | Non-sub-Saharan African vaccination study |
|  | Imboumy-Limoukou et al., | 2016 | Immunoglobulin response to the low polymorphic Pf113 antigen in children from Lastoursville, South-East of Gabon | Not related to immunogenicity, efficacy, and safety of TB/Malaria vaccine candidate |
|  | Mvubu et al., | 2016 | Mycobacterium tuberculosis strains exhibit differential and strain-specific molecular signatures in pulmonary epithelial cells | Not related to immunogenicity, efficacy, and safety of TB/Malaria vaccine candidate |
|  | Ntege et al., | 2016 | Identification of Plasmodium falciparum reticulocyte binding protein homologue 5-interacting protein, PfRipr, as a highly conserved blood-stage malaria vaccine candidate | Not related to immunogenicity, efficacy, and safety of TB/Malaria vaccine candidate |
|  | Floridia et al., | 2017 | Tuberculosis Case Finding with Combined Rapid Point-of-Care Assays (Xpert MTB/RIF and Determine TB LAM) in HIV-Positive Individuals Starting Antiretroviral Therapy in Mozambique | Not related to immunogenicity, efficacy, and safety of TB/Malaria vaccine candidate |
|  | Ferrian et al., | 2017 | A combination of baseline plasma immune markers can predict therapeutic response in multidrug resistant tuberculosis | Not related to immunogenicity, efficacy, and safety of TB/Malaria vaccine candidate |
|  | Dlamini et al., | 2017 | Low-Quality Housing Is Associated with Increased Risk of Malaria Infection: A National Population-Based Study from the Low Transmission Setting of Swaziland | Not related to immunogenicity, efficacy, and safety of TB/Malaria vaccine candidate |
|  | Wild et al., | 2017 | Tuberculosis, human rights and ethics considerations along the route of a highly vulnerable migrant from sub-Saharan Africa to Europe | Not related to immunogenicity, efficacy, and safety of TB/Malaria vaccine candidate |
|  | Turner et al., | 2017 | Tuberculosis Infectiousness and Host Susceptibility | Not related to immunogenicity, efficacy, and safety of TB/Malaria vaccine candidate |
|  | Moh et al., | 2017 | Screening for active tuberculosis before isoniazid preventive therapy among HIV-infected West African adults | Not related to immunogenicity, efficacy, and safety of TB/Malaria vaccine candidate |
|  | Raban et al., | 2016 | A randomised controlled trial of high vs low volume initiation and rapid vs slow advancement of milk feeds in infants with birthweights ≤ 1000 g in a resource-limited setting | Not related to immunogenicity, efficacy, and safety of TB/Malaria vaccine candidate |
|  | Franzen et al., | 2016 | Ultrathin bronchoscopy for solitary pulmonary lesions in a region endemic for tuberculosis: A randomised pilot trial | Not related to immunogenicity, efficacy, and safety of TB/Malaria vaccine candidate |
|  | Papas et al., | 2016 | Associations Between the Phosphatidylethanol Alcohol Biomarker and Self-Reported Alcohol Use in a Sample of HIV-Infected Outpatient Drinkers in Western Kenya | Non-TB/malaria study |
|  | Smith et al., | 2016 | The effect of neonatal vitamin A supplementation on morbidity and mortality at 12 months: A randomized trial | Not related to immunogenicity, efficacy, and safety of TB/Malaria vaccine candidate |
|  | Rustagi et al., | 2016 | Implementation and Operational Research: Impact of a Systems Engineering Intervention on PMTCT Service Delivery in Côte d'Ivoire, Kenya, Mozambique: A Cluster Randomized Trial | Not related to immunogenicity, efficacy, and safety of TB/Malaria vaccine candidate |
|  | Kargarfard et al., | 2016 | Efficacy of massage on muscle soreness, perceived recovery, physiological restoration and physical performance in male bodybuilders | Non-TB/malaria study |
|  | Skau et al., | 2016 | A complex behavioural change intervention to reduce the risk of diabetes and prediabetes in the pre-conception period in Malaysia: Study protocol for a randomised controlled trial | Non-TB/malaria study |
|  | Eikelboom et al., | 2016 | Perioperative Aspirin for Prevention of Venous Thromboembolism: The PeriOperative ISchemia Evaluation-2 Trial and a Pooled Analysis of the Randomized Trials | Non-TB/malaria study |
|  | Trevisanuta et al., | 2016 | Is a woolen cap effective in maintaining normothermia in low-birth-weight infants during kangaroo mother care? Study protocol for a randomized controlled trial | Non-TB/malaria study |
|  | Friedland et al., |  | Baseline Predictors of High Adherence to a Coitally Dependent Microbicide Gel Based on an Objective Marker of Use: Findings from the Carraguard Phase 3 Trial | Non-TB/malaria study |
|  | As’adi et al., | 2016 | A Randomized Controlled Trial Comparing Endoscopic Assisted Versus Open Neck Tissue Expander Placement in Reconstruction of Post-Burn Facial Scar Deformities | Non-TB/malaria study |
|  | Musinguzi et al., | 2016 | Comparison of subjective and objective adherence measures for preexposure prophylaxis against HIV infection among serodiscordant couples in East Africa | Non-TB/malaria study |
|  | Johnson et al., | 2016 | Comparison of Adding Treatment of PTSD During and After Shelter Stay to Standard Care in Residents of Battered Women’s Shelters: Results of a Randomized Clinical Trial | Non-TB/malaria study |
|  | Van Reijen et al., | 2016 | Increasing compliance with neuromuscular training to prevent ankle sprain in sport: Does the 'Strengthen your ankle' mobile App make a difference? A randomised controlled trial | Non-TB/malaria study |
|  | Ritz et al., | 2016 | Comparable CD4 and CD8 T cell responses and cytokine release after at-birth and delayed BCG immunisation in infants born in Australia | Non-sub-Saharan African study |
|  | Hertz et al., | 2016 | A study of vaccine-induced immune pressure on breakthrough infections in the Phambili phase 2b HIV-1 vaccine efficacy trial | Non-TB/malaria study |
|  | Spearman et al., | 2016 | A phase 1, randomized, controlled dose-escalation study of EP-1300 polyepitope DNA vaccine against Plasmodium falciparum malaria administered via electroporation | Non-sub-Sahara African study |
|  | Smet et al., | 2016 | Mycobacterium tuberculosis-associated synthetic mycolates differentially exert immune stimulatory adjuvant activity | Not related to immunogenicity, efficacy, and safety of TB/Malaria vaccine candidate |
|  | Maghendji-Nzondo et al., | 2016 | Prevalence of malaria, prevention measures, and main clinical features in febrile children admitted to the Franceville Regional Hospital, Gabon | Not related to immunogenicity, efficacy, and safety of TB/Malaria vaccine candidate |
|  | Fairhurst and Dondorp | 2016 | Artemisinin-Resistant Plasmodium falciparum Malaria | Not related to immunogenicity, efficacy, and safety of TB/Malaria vaccine candidate |
|  | Du Bruyn | 2016 | The Immune Interaction between HIV-1 Infection and Mycobacterium  tuberculosis | Not related to immunogenicity, efficacy, and safety of TB/Malaria vaccine candidate |
|  | Hatherill et al., | 2016 | Clinical Testing of Tuberculosis Vaccine Candidates | Not related to immunogenicity, efficacy, and safety of TB/Malaria vaccine candidate |
|  | Pallitto et al., | 2016 | Testing a counselling intervention in antenatal care for women experiencing partner violence: a study protocol for a randomized controlled trial in Johannesburg, South Africa | Not related to immunogenicity, efficacy, and safety of TB/Malaria vaccine candidate |
|  | Gcebe et al, | 2016 | Comparative Genomics and Proteomic Analysis of Four Non-tuberculous Mycobacterium Species and Mycobacterium tuberculosis Complex: Occurrence of Shared Immunogenic Proteins | Not related to immunogenicity, efficacy, and safety of TB/Malaria vaccine candidate |
|  | Iversen et al., | 2016 | Immunogenicity of the 9-valent HPV Vaccine Using 2-Dose Regimens in Girls and Boys Vs A 3-Dose Regimen in Women | Not related to immunogenicity, efficacy, and safety of TB/Malaria vaccine candidate |
|  | Timol et al., | 2016 | Addressing adolescents’ risk and protective factors related to risky behaviours: Findings from a school-based peer-education evaluation in the Western Cape | Not related to immunogenicity, efficacy, and safety of TB/Malaria vaccine candidate |
|  | Raban et al., | 2016 | A randomised controlled trial of high vs low volume initiation and rapid vs slow advancement of milk feeds in infants with birthweights ≤ 1000 g in a resource-limited setting | Non-TB/malaria study |
|  | Sazawal et al., | 2016 | Efficacy of chlorhexidine application to umbilical cord on neonatal mortality in Pemba, Tanzania: a community-based randomised controlled trial | Non-TB/malaria study |
|  | Muchiri et al., | 2016 | Effect of a nutrition education programme on clinical status and dietary behaviours of adults with type 2 diabetes in a resource-limited setting in South Africa: A randomised controlled trial | Non-TB/malaria study |
|  | Zijenah et al., | 2016 | Comparative performance characteristics of the urine lipoarabinomannan strip test and sputum smear microscopy in hospitalized HIV-infected patients with suspected tuberculosis in Harare, Zimbabwe | Not related to immunogenicity, efficacy, and safety of TB/Malaria vaccine candidate |
|  | Pinder et al., | 2016 | The RooPfs study to assess whether improved housing provides additional protection against clinical malaria over current best practice in The Gambia: Study protocol for a randomized controlled study and ancillary studies | Not related to immunogenicity, efficacy, and safety of TB/Malaria vaccine candidate |
|  | Ugwu et al., | 2016 | Randomized controlled trial comparing 200μg and 400μg sublingual misoprostol for prevention of primary postpartum hemorrhage | Non-TB/malaria study |
|  | Zlotnick et al., | 2016 | Randomized controlled trial to prevent postpartum depression in mothers on public assistance | Non-TB/malaria study |
|  | Lippman et al., | 2016 | Evaluation of short message service and peer navigation to improve engagement in HIV care in South Africa: Study protocol for a three-arm cluster randomized controlled trial | Non-TB/malaria study |
|  | Ter Heide et al., | 2016 | Eye movement desensitisation and reprocessing therapy v. stabilisation as usual for refugees: Randomised controlled trial | Non-TB/malaria study |
|  | Hoff et al., | 2016 | Sensitivity of C-Tb: A novel RD-1-specific skin test for the diagnosis of tuberculosis infection | Not related to immunogenicity, efficacy, and safety of TB/Malaria vaccine candidate |
|  | Ensoli et al., | 2016 | HIV-Tat immunization induces cross-clade neutralizing antibodies and CD4+ T cell increases in antiretroviral-treated South African volunteers: A randomized phase II clinical trial | Non-TB/malaria study |
|  | Rosen et al., | 2016 | Initiating Antiretroviral Therapy for HIV at a Patient’s First Clinic Visit: The RapIT Randomized Controlled Trial | Non-TB/malaria study |
|  | Churchyard et al., | 2016 | Sequential immunization with GP140 boosts immune responses primed by modified vaccinia Ankara or DNA in HIV-uninfected South African participants | Non-TB/malaria vaccination study |
|  | Kajula et al., | 2016 | Vijana Vijiweni II: A cluster-randomized trial to evaluate the efficacy of a microfinance and peer health leadership intervention for HIV and intimate partner violence prevention among social networks of young men in Dar es Salaam Global health | Non-TB/malaria study |
|  | De Villiers et al., | 2016 | Primary school children's nutrition knowledge, self-efficacy, and behavior, after a three-year healthy lifestyle intervention (HealthKick) | Non-TB/malaria study |
|  | Zhou et al., | 2016 | The impact of long-lasting microbial larvicides in reducing malaria transmission and clinical malaria incidence: Study protocol for a cluster randomized controlled trial | Not related to immunogenicity, efficacy, and safety of TB/Malaria vaccine candidate |
|  | Nel et al., | 2016 | Safety, acceptability and adherence of dapivirine vaginal ring in a microbicide clinical trial conducted in multiple countries in sub-Saharan Africa | Non-TB/malaria study |
|  | Hill et al., | 2016 | User and provider acceptability of intermittent screening and treatment and intermittent preventive treatment with dihydroartemisinin-piperaquine to prevent malaria in pregnancy in western Kenya | Not related to immunogenicity, efficacy, and safety of TB/Malaria vaccine candidate |
|  | Madhi et al., | 2016 | Safety and immunogenicity of an investigational maternal trivalent group B streptococcus vaccine in healthy women and their infants: a randomised phase 1b/2 trial | Non-TB/malaria study |
|  | Rhodes et al., | 2016 | Individual-level factors associated with variation in mycobacterial-specific immune response: Gender and previous BCG vaccination status | Not related to immunogenicity, efficacy, and safety of TB/Malaria vaccine candidate |
|  | Lambert et al., | 2016 | A Randomized Controlled Double-Blind Trial of Ciclosporin versus Prednisolone in the Management of Leprosy Patients with New Type 1 Reaction, in Ethiopia | Non-TB/malaria study |
|  | Afrane et al., | 2016 | Evaluation of long-lasting microbial larvicide for malaria vector control in Kenya | Not related to immunogenicity, efficacy, and safety of TB/Malaria vaccine candidate |
|  | Furin et al., | 2016 | Drug-resistant tuberculosis clinical trials: proposed core research definitions in adults | Not related to immunogenicity, efficacy, and safety of TB/Malaria vaccine candidate |
|  | Chang et al., | 2016 | Persistence of Plasmodium falciparum parasitemia after artemisinin combination therapy: evidence from a randomized trial in Uganda | Not related to immunogenicity, efficacy, and safety of TB/Malaria vaccine candidate |
|  | Lortholary et al., | 2016 | Tenofovir DF/emtricitabine and efavirenz combination therapy for HIV infection in patients treated for tuberculosis: the ANRS 129 BKVIR trial | Not related to immunogenicity, efficacy, and safety of TB/Malaria vaccine candidate |
|  | Dinga et al., | 2016 | Differential T-cell responses to a chimeric Plasmodium falciparum antigen; UB05-09, correlates with acquired immunity to malaria | Not related to immunogenicity, efficacy, and safety of TB/Malaria vaccine candidate |
|  | Bertin et al., | 2016 | Proteomic analysis of Plasmodium falciparum parasites from patients with cerebral and uncomplicated malaria | Not related to immunogenicity, efficacy, and safety of TB/Malaria vaccine candidate |
|  | Longwe et al., | 2016 | Delayed acquisition of Plasmodium falciparum antigen-specific CD4(+) T cell responses in HIV-exposed uninfected Malawian children receiving daily cotrimoxazole prophylaxis | Not related to immunogenicity, efficacy, and safety of TB/Malaria vaccine candidate |
|  | Bowker et al., | 2016 | Polymorphisms in the Pattern Recognition Receptor Mincle Gene (CLEC4E) and Association with Tuberculosis | Not related to immunogenicity, efficacy, and safety of TB/Malaria vaccine candidate |
|  | Jabot-Hanin et al., | 2016 | Major Loci on Chromosomes 8q and 3q Control Interferon gamma Production Triggered by Bacillus Calmette-Guerin and 6-kDa Early Secretory Antigen Target, Respectively, in Various Populations | Not related to immunogenicity, efficacy, and safety of TB/Malaria vaccine candidate |
|  | Kefyalew et al., | 2016 | Health worker and policy-maker perspectives on use of intramuscular artesunate for pre-referral and definitive treatment of severe malaria at health posts in Ethiopia | Not related to immunogenicity, efficacy, and safety of TB/Malaria vaccine candidate |
|  | Kasirye et al., | 2016 | Longitudinal effect of CD4 by cotrimoxazole use on malaria incidence among HIV-infected Ugandan adults on antiretroviral therapy: a randomized controlled study | Not related to immunogenicity, efficacy, and safety of TB/Malaria vaccine candidate |
|  | Riou et al., | 2016 | HIV Skews the Lineage-Defining Transcriptional Profile of Mycobacterium tuberculosis-Specific CD4(+) T Cells | Not related to immunogenicity, efficacy, and safety of TB/Malaria vaccine candidate |
|  | Dambuza et al., | 2016 | Persistent p55TNFR expression impairs T cell responses during chronic tuberculosis and promotes reactivation | Not related to immunogenicity, efficacy, and safety of TB/Malaria vaccine candidate |
|  | Ampah et al., | 2016 | Burden and Historical Trend of Buruli Ulcer Prevalence in Selected Communities along the Offin River of Ghana | Non-TB/malaria study |
|  | Mourano et al., | 2016 | Anti-erythrocyte antibodies may contribute to anaemia in Plasmodium vivax malaria by decreasing red blood cell deformability and increasing erythrophagocytosis | Not related to immunogenicity, efficacy, and safety of TB/Malaria vaccine candidate |
|  | Kimani et al., | 2016 | Efficacy and Safety of Azithromycin-Chloroquine versus Sulfadoxine-Pyrimethamine for Intermittent Preventive Treatment of Plasmodium falciparum Malaria Infection in Pregnant Women in Africa: An Open-Label, Randomized Trial | Not related to immunogenicity, efficacy, and safety of TB/Malaria vaccine candidate |
|  | Viana et al., | 2016 | Delayed mortality effects cut the malaria transmission potential of insecticide-resistant mosquitoes | Not related to immunogenicity, efficacy, and safety of TB/Malaria vaccine candidate |
|  | Addai-mensah et al., | 2016 | Acquired immune responses to three malaria vaccine candidates and their relationship to invasion inhibition in two populations naturally exposed to malaria | Not related to immunogenicity, efficacy, and safety of TB/Malaria vaccine candidate |
|  | Diedrich et al., | 2016 | Relationship Between HIV Coinfection, Interleukin 10 Production, and Mycobacterium tuberculosis in Human Lymph Node Granulomas | Not related to immunogenicity, efficacy, and safety of TB/Malaria vaccine candidate |
|  | Dugassa et al., | 2016 | Field evaluation of two novel sampling devices for collecting wild oviposition site seeking malaria vector mosquitoes: OviART gravid traps and squares of electrocuting nets | Not related to immunogenicity, efficacy, and safety of TB/Malaria vaccine candidate |
|  | Leang et al., | 2016 | Efficacy and Safety of Pyronaridine-Artesunate for Treatment of Uncomplicated Plasmodium falciparum Malaria in Western Cambodia | Not related to immunogenicity, efficacy, and safety of TB/Malaria vaccine candidate |
|  | Gabryszewski et al., | 2016 | Evolution of Fitness Cost-Neutral Mutant PfCRT Conferring P. falciparum 4-Aminoquinoline Drug Resistance Is Accompanied by Altered Parasite Metabolism and Digestive Vacuole Physiology | Not related to immunogenicity, efficacy, and safety of TB/Malaria vaccine candidate |
|  | Krause et al., | 2017 | Plasmodium glyceraldehyde-3-phosphate dehydrogenase: A potential malaria diagnostic target | Not related to immunogenicity, efficacy, and safety of TB/Malaria vaccine candidate |
|  | Thompson et al., | 2017 | Host blood RNA signatures predict the outcome of tuberculosis treatment | Not related to immunogenicity, efficacy, and safety of TB/Malaria vaccine candidate |
|  | Bansal et al., | 2017 | Antibodies elicited during natural infection in a predominantly Plasmodium falciparum transmission area cross-react with sexual stage-specific antigen in P-vivax | Not related to immunogenicity, efficacy, and safety of TB/Malaria vaccine candidate |
|  | Gouttebarge et al., | 2017 | Preventing musculoskeletal injuries among recreational adult volleyball players: Design of a randomised prospective controlled trial | Non-TB/malaria study |
|  | Mollel and chilongola | 2017 | Predictors for Mortality among Multidrug-Resistant Tuberculosis Patients in Tanzania | Not related to immunogenicity, efficacy, and safety of TB/Malaria vaccine candidate |
|  | Matangila et al., | 2017 | Efficacy and safety of intermittent preventive treatment in schoolchildren with sulfadoxine/pyrimethamine (SP) and SP plus piperaquine in Democratic Republic of Congo: a randomised controlled trial | Not related to immunogenicity, efficacy, and safety of TB/Malaria vaccine candidate |
|  | Bork et al., | 2017 | Formula-Feeding of HIV-Exposed Uninfected African Children Is Associated with Faster Growth in Length during the First 6 Months of Life in the Kesho Bora Study | Non-TB/malaria study |
|  | Schrieff-Elson et al., | 2017 | Attention-training with children from socioeconomically disadvantaged backgrounds in Cape Town | Non-TB/malaria study |
|  | Madhi et al., | 2017 | Immunization with 10-valent pneumococcal non-typeable Haemophilus influenzae protein D conjugate vaccine (PHiD-CV) according to different schedules in infants in South Africa: a phase III trial | Non-TB/malaria study |
|  | Cornejo-Granados et al., | 2017 | Secretome Prediction of Two M. tuberculosis Clinical Isolates Reveals Their High Antigenic Density and Potential Drug Targets | Not related to immunogenicity, efficacy, and safety of TB/Malaria vaccine candidate |
|  | Riou et al., | 2017 | Analysis of the Phenotype of Mycobacterium tuberculosis-Specific CD4+T Cells to Discriminate Latent from Active Tuberculosis in HIV-Uninfected and HIV-Infected Individuals | Not related to immunogenicity, efficacy, and safety of TB/Malaria vaccine candidate |
|  | Rockwood et al., | 2017 | Mycobacterium tuberculosis induction of heme Oxygenase-1 expression is Dependent on Oxidative stress and reflects Treatment Outcomes | Not related to immunogenicity, efficacy, and safety of TB/Malaria vaccine candidate |
|  | Parsons et al., | 2017 | The Kinetics of the Humoral and Interferon-Gamma Immune Responses to Experimental Mycobacterium bovis Infection in the White Rhinoceros (Ceratotherium simum) | Not related to immunogenicity, efficacy, and safety of TB/Malaria vaccine candidate |
|  | Whittaker et al., | 2017 | Regulatory T Cells and Pro-inflammatory Responses Predominate in children with Tuberculosis | Not related to immunogenicity, efficacy, and safety of TB/Malaria vaccine candidate |
|  | Aguilar et al., | 2017 | Assessment of the Combined Effect of Epstein-Barr Virus and Plasmodium falciparum Infections on Endemic Burkitt Lymphoma Using a Multiplex Serological Approach | Not related to immunogenicity, efficacy, and safety of TB/Malaria vaccine candidate |
|  | Tousif et al., | 2017 | Nanoparticle-Formulated Curcumin Prevents Posttherapeutic Disease Reactivation and Reinfection with Mycobacterium tuberculosis following Isoniazid Therapy | Not related to immunogenicity, efficacy, and safety of TB/Malaria vaccine candidate |
|  | Gcebe et al., | 2017 | Non-tuberculous Mycobacteria in South African Wildlife: Neglected Pathogens and Potential Impediments for Bovine Tuberculosis Diagnosis | Not related to immunogenicity, efficacy, and safety of TB/Malaria vaccine candidate |
|  | Bhattacharya et al., | 2017 | Cellular Architecture of Spinal Granulomas and the Immunological Response in Tuberculosis Patients Coinfected with HIV | Not related to immunogenicity, efficacy, and safety of TB/Malaria vaccine candidate |
|  | Hsu et al., | 2017 | Myeloid and T Cell-Derived TNF Protects against Central Nervous System Tuberculosis | Not related to immunogenicity, efficacy, and safety of TB/Malaria vaccine candidate |
|  | Bonney et al., | 2017 | The efficacy of two activity-based interventions in adolescents with Developmental Coordination Disorder | Non-TB/malaria study |
|  | Chirawurah et al., | 2017 | Antimalarial activity of Malaria Box Compounds against Plasmodium falciparum clinical isolates | Not related to immunogenicity, efficacy, and safety of TB/Malaria vaccine candidate |
|  | Deshpande et al., | 2016 | Thioridazine as Chemotherapy for Mycobacterium avium Complex Diseases | Not related to immunogenicity, efficacy, and safety of TB/Malaria vaccine candidate |
|  | Otienoburu et al., | 2016 | Selection of Plasmodium falciparum pfcrt and pfmdr1 polymorphisms after treatment with artesunate-amodiaquine fixed dose combination or artemether-lumefantrine in Liberia | Not related to immunogenicity, efficacy, and safety of TB/Malaria vaccine candidate |
|  | Parsons et al., | 2016 | Antigen-Specific IP-10 Release Is a Sensitive Biomarker of Mycobacterium bovis Infection in Cattle | Not related to immunogenicity, efficacy, and safety of TB/Malaria vaccine candidate |
|  | Jegede et al., | 2016 | Assessing Acceptability of a Diagnostic and Malaria Treatment Package Delivered by Community Health Workers in Malaria-Endemic Settings of Burkina Faso, Nigeria, and Uganda | Not related to immunogenicity, efficacy, and safety of TB/Malaria vaccine candidate |
|  | Ferro et al., | 2016 | Tigecycline Is Highly Efficacious against Mycobacterium abscessus pulmonary disease | Not related to immunogenicity, efficacy, and safety of TB/Malaria vaccine candidate |
|  | Poirier et al., | 2016 | The hide and seek of Plasmodium vivax in West Africa: report from a large-scale study in Beninese asymptomatic subjects | Not related to immunogenicity, efficacy, and safety of TB/Malaria vaccine candidate |
|  | Verkuijl et al., | 2016 | Protecting Our Front-liners: Occupational Tuberculosis Prevention Through Infection Control Strategies | Not related to immunogenicity, efficacy, and safety of TB/Malaria vaccine candidate |
|  | Hanifa et al., | 2016 | Diagnostic Accuracy of Lateral Flow Urine LAM Assay for TB Screening of Adults with Advanced Immunosuppression Attending Routine HIV Care in South Africa | Non-TB/malaria study |
|  | Niang et al., | 2016 | Patterns of insecticide resistance and knock down resistance (kdr) in malaria vectors An. arabiensis, An. coluzzii and An. gambiae from sympatric areas in Senegal | Not related to immunogenicity, efficacy, and safety of TB/Malaria vaccine candidate |
|  | Aseffa et al., | 2016 | Efficacy and Safety of `Fixed Dose' versus `Loose' Drug Regimens for Treatment of Pulmonary Tuberculosis in Two High TB-Burden African Countries: A Randomized Controlled Trial | Not related to immunogenicity, efficacy, and safety of TB/Malaria vaccine candidate |
|  | Hwysell et al., | 2016 | Undertreated HIV and drug-resistant tuberculosis at a referral hospital in Irkutsk, Siberia | Not related to immunogenicity, efficacy, and safety of TB/Malaria vaccine candidate |
|  | Febir et al., | 2016 | Seeking treatment for uncomplicated malaria: experiences from the Kintampo districts of Ghana | Not related to immunogenicity, efficacy, and safety of TB/Malaria vaccine candidate |
|  | N'Guessan et al., | 2016 | A Chlorfenapyr Mixture Net Interceptor (R) G2 Shows High Efficacy and Wash Durability against Resistant Mosquitoes in West Africa | Not related to immunogenicity, efficacy, and safety of TB/Malaria vaccine candidate |
|  | Brennan et al., | 2016 | Incident tuberculosis in HIV-positive children, adolescents, and adults on antiretroviral therapy in South Africa | Not related to immunogenicity, efficacy, and safety of TB/Malaria vaccine candidate |
|  | Williams et al., | 2016 | Non-falciparum malaria infections in pregnant women in West Africa | Not related to immunogenicity, efficacy, and safety of TB/Malaria vaccine candidate |
|  | Furin et al., | 2016 | Early Bactericidal Activity of AZD5847 in Patients with Pulmonary Tuberculosis | Not related to immunogenicity, efficacy, and safety of TB/Malaria vaccine candidate |
|  | Jaleta et al., | 2016 | Chicken volatiles repel host-seeking malaria mosquitoes | Not related to immunogenicity, efficacy, and safety of TB/Malaria vaccine candidate |
|  | Kanyangarara et al., | 2016 | Reduction in Malaria Incidence following Indoor Residual Spraying with Actellic 300 CS in a Setting with Pyrethroid Resistance: Mutasa District, Zimbabwe | Not related to immunogenicity, efficacy, and safety of TB/Malaria vaccine candidate |
|  | Tudor et al., | 2016 | Occupational Risk Factors for Tuberculosis Among Healthcare Workers in KwaZulu-Natal, South Africa | Not related to immunogenicity, efficacy, and safety of TB/Malaria vaccine candidate |
|  | Goodman et al., | 2016 | Natural products from Zanthoxylum heitzii with potent activity against the malaria parasite | Not related to immunogenicity, efficacy, and safety of TB/Malaria vaccine candidate |
|  | Lagarde and Blaauw | 2017 | Physicians’ responses to financial and social incentives: A medically framed real effort experiment | Non-TB/malaria study |
|  | Lachman et al., | 2017 | Randomized controlled trial of a parenting program to reduce the risk of child maltreatment in South Africa | Non-TB/malaria study |
|  | Atterbury et al., | 2017 | Balance training in individuals with Parkinson's disease: Therapist-supervised vs. home-based exercise programme | Non-TB/malaria study |
|  | Tscholl et al., | 2017 | Prospective randomized study evaluating the effects of PerClot® (Polysaccharide Hemostatic System) application in patients with high bleeding risk undergoing cardiac rhythm device implantation | Non-TB/malaria study |
|  | Chin et al., | 2017 | Rationale and design of a prospective study to assess the effect of left cardiac sympathetic denervation in chronic heart failure | Non-TB/malaria study |
|  | Ojal et al., | 2017 | Pneumococcal conjugate vaccine induced IgG and nasopharyngeal carriage of pneumococci: Hyporesponsiveness and immune correlates of protection for carriage | Non-TB/malaria study |
|  | Dinzouna-Boutamba et al., | 2017 | Determination of multiple-clone infection at allelic dimorphism site of Plasmodium vivax merozoite surface protein-1 in the Republic of Korea by pyrosequencing assay | Not related to immunogenicity, efficacy, and safety of TB/Malaria vaccine candidate |
|  | Wehner et al., | 2017 | Malaria incidence during early childhood in rural Burkina Faso: Analysis of a birth cohort protected with insecticide-treated mosquito nets | Not related to immunogenicity, efficacy, and safety of TB/Malaria vaccine candidate |
|  | Birhanu et al., | 2017 | Relationship between exposure to malaria and haemoglobin level of children 2-9 years old in low malaria transmission settings | Not related to immunogenicity, efficacy, and safety of TB/Malaria vaccine candidate |
|  | Gureje et al., | 2017 | COllaborative Shared care to IMprove Psychosis Outcome (COSIMPO): Study protocol for a randomized controlled trial | Non-TB/malaria study |
|  | Smith et al., | 2017 | A randomized clinical trial comparing cervical dysplasia treatment with cryotherapy vs loop electrosurgical excision procedure in HIV-seropositive women from Johannesburg, South Africa | Not related to immunogenicity, efficacy, and safety of TB/Malaria vaccine candidate |
|  | Realini et al., | 2017 | West Indies Glaucoma Laser Study (WIGLS): 1. 12-Month Efficacy of Selective Laser Trabeculoplasty in Afro-Caribbeans with Glaucoma | Non-TB/malaria study |
|  | Morgan et al., | 2017 | Serotonin transporter gene (SLC6A4) polymorphism and susceptibility to a home-visiting maternal-infant attachment intervention delivered by community health workers in South Africa: Reanalysis of a randomized controlled trial | Non-TB/malaria study |
|  | Van Reijen et al., | 2017 | The “Strengthen your ankle” program to prevent recurrent injuries: A randomized controlled trial aimed at long-term effectiveness | Non-TB/malaria study |
|  | Amoakoh et al., | 2017 | The effect of a clinical decision-making mHealth support system on maternal and neonatal mortality and morbidity in Ghana: Study protocol for a cluster randomized controlled trial | Non-TB/malaria study |
|  | Choko et al., | 2017 | Investigating interventions to increase uptake of HIV testing and linkage into care or prevention for male partners of pregnant women in antenatal clinics in Blantyre, Malawi: Study protocol for a cluster randomised trial | Non-TB/malaria study |
|  | Ekirapa-Kiracho | 2017 | Uganda Newborn Study (UNEST) trial: Community-based maternal and newborn care economic analysis | Non-TB/malaria study |
|  | Grellety, et al., | 2017 | Effects of unconditional cash transfers on the outcome of treatment for severe acute malnutrition (SAM): A cluster-randomised trial in the Democratic Republic of the Congo | Non-TB/malaria study |
|  | Patil et al., | 2017 | Implementation challenges and outcomes of a randomized controlled pilot study of a group prenatal care model in Malawi and Tanzania | Non-TB/malaria study |
|  | Madhombiro et al., | 2017 | A cluster randomised controlled trial protocol of an adapted intervention for alcohol use disorders in people living with HIV and AIDS: Impact on alcohol use, general functional ability, quality of life and adherence to HAART | Non-TB/malaria study |
|  | Houngbe et al., | 2017 | Unconditional cash transfers do not prevent children's undernutrition in themoderate acute malnutrition out (MAM'Out) cluster-randomized controlled trial in rural Burkina Faso | Non-TB/malaria study |
|  | Rotheram-Borus et al., | 2017 | To evaluate if increased supervision and support of South African Government health workers' home visits improves maternal and child outcomes: Study protocol for a randomized control trial | Non-TB/malaria study |
|  | Sabur et al., | 2017 | Diagnosing tuberculosis in hospitalized HIV-infected individuals who cannot produce sputum: Is urine lipoarabinomannan testing the answer? | Non-TB/malaria study |
|  | Dorward et al., | 2017 | Factors associated with poor linkage to HIV care in South Africa: Secondary analysis of data from the Thol'impilo trial | Non-TB/malaria study |
|  | Dirajlal-Fargo et al., | 2017 | Insulin Resistance and Markers of Inflammation in HIV-infected Ugandan Children in the CHAPAS-3 Trial | Non-TB/malaria study |
|  | Carballo-Diéguez et al., | 2017 | High levels of adherence to a rectal microbicide gel and to oral Pre-Exposure Prophylaxis (PrEP) achieved in MTN-017 among men who have sex with men (MSM) and transgender women | Non-TB/malaria study |
|  | Baiocchi et al., | 2017 | A Behavior-Based Intervention That Prevents Sexual Assault: The Results of a Matched-Pairs, Cluster-Randomized Study in Nairobi, Kenya | Non-TB/malaria study |
|  | Wandera et al., | 2017 | Efficacy of a Single, Brief Alcohol Reduction Intervention among Men and Women Living with HIV/AIDS and Using Alcohol in Kampala, Uganda: A Randomized Trial | Non-TB/malaria study |
|  | Adachi et al., | 2017 | Cytomegalovirus Urinary Shedding in HIV-infected Pregnant Women and Congenital Cytomegalovirus Infection | Non-TB/malaria study |
|  | Lawrie et al., | 2017 | Participant recruitment and retention in longitudinal preconception randomized trials: Lessons learnt from the Calcium and Pre-eclampsia (CAP) trial | Non-TB/malaria study |
|  | Adams et al., | 2017 | Maternal and child supplementation with lipid-based nutrient supplements, but not child supplementation alone, decreases self-reported household food insecurity in some settings | Non-TB/malaria study |
|  | Kaljeeet al., | 2017 | A randomized-control trial for the teachers’ diploma programme on psychosocial care, support and protection in Zambian government primary schools | Non-TB/malaria study |
|  | Montgomery et al., | 2017 | Acceptability and use of a dapivirine vaginal ring in a phase III trial | Non-TB/malaria study |
|  | Gasper et al., | 2017 | BCG vaccination induces HIV target cell activation in HIV-exposed infants in a randomized trial | Recheck |
|  | Chimoyi et al., | 2017 | Using surrogate vaccines to assess feasibility and acceptability of future HIV vaccine trials in men: A randomised trial in inner-city Johannesburg, South Africa | Non-TB/malaria study |
|  | Madhi et al., | 2017 | Vaccination with 10-valent pneumococcal conjugate vaccine in infants according to HIV status | Non-TB/malaria study |
|  | Paddick et al., | 2017 | Cognitive stimulation therapy as a sustainable intervention for dementia in sub-Saharan Africa: Feasibility and clinical efficacy using a stepped-wedge design | Non-TB/malaria study |
|  | Daviaud et al., | 2017 | South-Africa (Goodstart III) trial: Community-based maternal and newborn care economic analysis | Non-TB/malaria study |
|  | Price et al., | 2017 | Hepatitis B serological markers and plasma DNA concentrations | Non-TB/malaria study |
|  | Greer et al., | 2017 | Comparison of hepatitis b virus infection in HIV-infected and HIV-uninfected participants enrolled in a multinational clinical trial: HPTN 052 | Non-TB/malaria study |
|  | Lewinsohn et al., | 2017 | Comprehensive definition of human immunodominant CD8 antigens in tuberculosis | Not related to immunogenicity, efficacy, and safety of TB/Malaria vaccine candidate |
|  | Siriez et al., | 2017 | Post-Malaria Neurologic Syndrome: A Rare Pediatric Case Report | Not related to immunogenicity, efficacy, and safety of TB/Malaria vaccine candidate |
|  | Chico et al., | 2017 | Sulfadoxine-Pyrimethamine Exhibits Dose-Response Protection Against Adverse Birth Outcomes Related to Malaria and Sexually Transmitted and Reproductive Tract Infections | Not related to immunogenicity, efficacy, and safety of TB/Malaria vaccine candidate |
|  | Andrews et al., | 2017 | Serial QuantiFERON testing and tuberculosis disease risk among young children: an observational cohort study | Not related to immunogenicity, efficacy, and safety of TB/Malaria vaccine candidate |
|  | Pathmanathan et al., | 2017 | Incidence and predictors of tuberculosis among HIV-infected adults after initiation of antiretroviral therapy in Nigeria, 2004-2012 | Not related to immunogenicity, efficacy, and safety of TB/Malaria vaccine candidate |
|  | Obaldia et al., | 2017 | A Plasmodium vivax Plasmid DNA- and Adenovirus-Vectored Malaria Vaccine Encoding Blood-Stage Antigens AMA1 and MSP1(42) in a Prime/Boost Heterologous Immunization Regimen Partially Protects Aotus Monkeys against Blood-Stage Challenge | Pre-clinical study |
|  | Zhu et al., | 2017 | Is outdoor vector control needed for malaria elimination? An individual-based modelling study | Not related to immunogenicity, efficacy, and safety of TB/Malaria vaccine candidate |
|  | Flick et al., | 2017 | Assessing infection control practices to protect health care workers and patients in Malawi from nosocomial transmission of Mycobacteriumtuberculosis | Not related to immunogenicity, efficacy, and safety of TB/Malaria vaccine candidate |
|  | Amelio et al., | 2017 | Mixed Th1 and Th2 Mycobacterium tuberculosis-specific CD4 T cell responses in patients with active pulmonary tuberculosis from Tanzania | Not related to immunogenicity, efficacy, and safety of TB/Malaria vaccine candidate |
|  | Fenner et al., | 2017 | HIV viral load as an independent risk factor for tuberculosis in South Africa: collaborative analysis of cohort studies | Not related to immunogenicity, efficacy, and safety of TB/Malaria vaccine candidate |
|  |  |  |  |  |
|  | Bunjun et al., | 2017 | Effect of HIV on the Frequency and Number of Mycobacterium tuberculosis-Specific CD4(+) T Cells in Blood and Airways During Latent M-tuberculosis Infection | Not related to immunogenicity, efficacy, and safety of TB/Malaria vaccine candidate |
|  | Lin et al., | 2017 | Signatures of malaria-associated pathology revealed by high-resolution whole-blood transcriptomics in a rodent model of malaria | Not related to immunogenicity, efficacy, and safety of TB/Malaria vaccine candidate |
|  | Kobylinski et al., | 2017 | Ivermectin susceptibility and sporontocidal effect in Greater Mekong Subregion Anopheles | Not related to immunogenicity, efficacy, and safety of TB/Malaria vaccine candidate |
|  | Nambozi et al., | 2017 | Artemisinin-based combination therapy in pregnant women in Zambia: efficacy, safety and risk of recurrent malaria | Not related to immunogenicity, efficacy, and safety of TB/Malaria vaccine candidate |
|  | Radtke et al., | 2017 | Adjuvant and carrier protein-dependent T-cell priming promotes a robust antibody response against the Plasmodium falciparum Pfs25 vaccine candidate | Pre-clinical study |
|  | Janssen et al., | 2017 | Mortality in Severe Human Immunodeficiency Virus-Tuberculosis Associates with Innate Immune Activation and Dysfunction of Monocytes | Not related to immunogenicity, efficacy, and safety of TB/Malaria vaccine candidate |
|  | Miles et al., | 2017 | Genetic diversity of the African malaria vector Anopheles gambiae | Not related to immunogenicity, efficacy, and safety of TB/Malaria vaccine candidate |
|  | Toure et al., | 2017 | Assessment of Efficacy and Safety of Arterolane Maleate-Piperaquine Phosphate Dispersible Tablets in Comparison with Artemether-Lumefantrine Dispersible Tablets in Pediatric Patients with Acute Uncomplicated Plasmodium falciparum Malaria: A Phase 3, Randomized, Multicenter Trial in India and Africa | Not related to immunogenicity, efficacy, and safety of TB/Malaria vaccine candidate |
|  | Eholie et al., | 2017 | Effect of cotrimoxazole prophylaxis on malaria occurrence among HIV-infected adults in West Africa: the MALHIV Study | Non-TB/malaria study |
|  | Eldering et al., | 2017 | Comparative assessment of An. gambiae and An. stephensi mosquitoes to determine transmission-reducing activity of antibodies against P-falciparum sexual stage antigens | Not related to immunogenicity, efficacy, and safety of TB/Malaria vaccine candidate |
|  | Biggs et al., | 2017 | Serology reveals heterogeneity of Plasmodium falciparum transmission in northeastern South Africa: implications for malaria elimination | Not related to immunogenicity, efficacy, and safety of TB/Malaria vaccine candidate |
|  | Balikagala et al., | 2017 | Absence of in vivo selection for K13 mutations after artemether-lumefantrine treatment in Uganda | Not related to immunogenicity, efficacy, and safety of TB/Malaria vaccine candidate |
|  | Tham et al., | 2017 | Plasmodium vivax vaccine research - we've only just begun | Not related to immunogenicity, efficacy, and safety of TB/Malaria vaccine candidate |
|  | Day et al., | 2017 | HIV-1 Infection Is Associated with Depletion and Functional Impairment of Mycobacterium Tuberculosis-Specific CD4 T Cells in Individuals with Latent Tuberculosis Infection | Not related to immunogenicity, efficacy, and safety of TB/Malaria vaccine candidate |
|  | Mogire et al., | 2017 | Target-similarity search using Plasmodium falciparum proteome identifies approved drugs with anti-malarial activity and their possible targets | Not related to immunogenicity, efficacy, and safety of TB/Malaria vaccine candidate |
|  | Dheda et al., | 2017 | The epidemiology, pathogenesis, transmission, diagnosis, and management of multidrug-resistant, extensively drug-resistant, and incurable tuberculosis | Not related to immunogenicity, efficacy, and safety of TB/Malaria vaccine candidate |
|  | Shah et al., | 2017 | A Functional Toll-Interacting Protein Variant Is Associated with Bacillus Calmette-Guerin-Specific Immune Responses and Tuberculosis | Not related to immunogenicity, efficacy, and safety of TB/Malaria vaccine candidate |
|  | Tine et al., | 2017 | Safety and Efficacy of Adding a Single Low Dose of Primaquine to the Treatment of Adult Patients with Plasmodium falciparum Malaria in Senegal, to Reduce Gametocyte Carriage: A Randomized Controlled Trial | Not related to immunogenicity, efficacy, and safety of TB/Malaria vaccine candidate |
|  | Youdom et al., | 2017 | Comparison of anti-malarial drugs efficacy in the treatment of uncomplicated malaria in African children and adults using network meta-analysis | Not related to immunogenicity, efficacy, and safety of TB/Malaria vaccine candidate |
|  | van der Heijden et al., | 2017 | Immune response profiles of calves following vaccination with live BCG and inactivated Mycobacterium bovis vaccine candidates | Non-human study |
|  | Lynch et al., | 2017 | Travel and the emergence of high-level drug resistance in Plasmodium falciparum in southwest Uganda: results from a population-based study | Not related to immunogenicity, efficacy, and safety of TB/Malaria vaccine candidate |
|  | Yewhalaw et al., | 2017 | Determination of the residual efficacy of carbamate and organophosphate insecticides used for indoor residual spraying for malaria control in Ethiopia | Not related to immunogenicity, efficacy, and safety of TB/Malaria vaccine candidate |
|  | Clouse et al., | 2017 | Low implementation of Xpert MTB/RIF among HIV/TBco-infected adults in the International epidemiologic Databases to Evaluate AIDS (IeDEA) program | Not related to immunogenicity, efficacy, and safety of TB/Malaria vaccine candidate |
|  | Clouse et al., | 2017 | Clinic-Based Urinary Lipoarabinomannan as a Biomarker of Clinical Disease Severity and Mortality Among Antiretroviral Therapy-Naive Human Immunodeficiency Virus-Infected Adults in South Africa | Not related to immunogenicity, efficacy, and safety of TB/Malaria vaccine candidate |
|  | Cumming et al., | 2017 | Mycobacterium tuberculosis arrests host cycle at the G(1)/S transition to establish long term infection | Not related to immunogenicity, efficacy, and safety of TB/Malaria vaccine candidate |
|  | Manyangadze et al., | 2017 | Micro-spatial distribution of malaria cases and control strategies at ward level in Gwanda district, Matabeleland South, Zimbabwe | Non-TB/malaria study |
|  | Bei et al., | 2017 | Functional Analysis Reveals Geographical Variation in Inhibitory Immune Responses Against a Polymorphic Malaria Antigen | Not related to immunogenicity, efficacy, and safety of TB/Malaria vaccine candidate |
|  | Hanifa et al., | 2017 | A clinical scoring system to prioritise investigation for tuberculosis among adults attending HIV clinics in South Africa | Not related to immunogenicity, efficacy, and safety of TB/Malaria vaccine candidate |
|  | Batista et al., | 2017 | Semi-field assessment of the BG-Malaria trap for monitoring the African malaria vector, Anopheles arabiensis | Not related to immunogenicity, efficacy, and safety of TB/Malaria vaccine candidate |
|  | Javeed et al., | 2017 | A Functional Toll-Interacting Protein Variant Is Associated with Bacillus Calmette-Guérin–Specific Immune Responses and Tuberculosis | Not related to immunogenicity, efficacy, and safety of TB/Malaria vaccine candidate |
|  | Ademoule et al., | 2017 | Patterns of inflammatory responses and parasite tolerance vary with malaria transmission intensity | Not related to immunogenicity, efficacy, and safety of TB/Malaria vaccine candidate |
|  | Ippolito et al., | 2017 | House Structure Is Associated with Plasmodium falciparum Infection in a Low-Transmission Setting in Southern Zambia | Not related to immunogenicity, efficacy, and safety of TB/Malaria vaccine candidate |
|  | Tetard et al., | 2017 | Heterozygous HbAC but not HbAS is associated with higher newborn birthweight among women with pregnancy-associated malaria | Not related to immunogenicity, efficacy, and safety of TB/Malaria vaccine candidate |
|  | Kurthkoti et al., | 2017 | The Capacity of Mycobacterium tuberculosis To Survive Iron Starvation Might Enable It To Persist in Iron-Deprived Microenvironments of Human Granulomas | Not related to immunogenicity, efficacy, and safety of TB/Malaria vaccine candidate |
|  | Shenoi et al., | 2017 | Integrated Tuberculosis/Human Immunodeficiency Virus Community-Based Case Finding in Rural South Africa: Implications for Tuberculosis Control Efforts | Not related to immunogenicity, efficacy, and safety of TB/Malaria vaccine candidate |
|  | Malotle et al., | 2017 | Occupational tuberculosis in South Africa: are health care workers adequately protected? | Not related to immunogenicity, efficacy, and safety of TB/Malaria vaccine candidate |
|  | Halsey et al., | 2017 | Capacity Development through the US President's Malaria Initiative-Supported Antimalarial Resistance Monitoring in Africa Network | Not related to immunogenicity, efficacy, and safety of TB/Malaria vaccine candidate |
|  | Dowdy et al., | 2017 | Designing and Evaluating Interventions to Halt the Transmission ofTuberculosis | Not related to immunogenicity, efficacy, and safety of TB/Malaria vaccine candidate |
|  | Choi et al., | 2017 | Cost-effectiveness of malaria preventive treatment for HIV-infected pregnant women in sub-Saharan Africa | Not related to immunogenicity, efficacy, and safety of TB/Malaria vaccine candidate |
|  | Goncalves et al., | 2017 | Hemoglobin variants shape the distribution of malaria parasites in human populations and their transmission potential | Not related to immunogenicity, efficacy, and safety of TB/Malaria vaccine candidate |
|  | Stephens et al., | 2017 | Effect of IPTp on Plasmodium falciparum antibody levels among pregnant | Not related to immunogenicity, efficacy, and safety of TB/Malaria vaccine candidate |
|  | Usdin et al., | 2017 | Latent tuberculous screening of recent migrants attending language classes: a cohort study and cost analysis | Not related to immunogenicity, efficacy, and safety of TB/Malaria vaccine candidate |
|  | Russo et al., | 2017 | Molecular evidence of Plasmodium vivax infection in Duffy negative symptomatic individuals from Dschang, West Cameroon | Not related to immunogenicity, efficacy, and safety of TB/Malaria vaccine candidate |
|  | Messenger et al., | 2017 | Insecticide resistance in Anopheles arabiensis from Ethiopia (2012-2016): a nationwide study for insecticide resistance monitoring | Not related to immunogenicity, efficacy, and safety of TB/Malaria vaccine candidate |
|  | Msellemu et al., | 2017 | The underlying reasons for very high levels of bed net use, and higher malaria infection prevalence among bed net users than non-users in the Tanzanian city of Dar es Salaam: a qualitative study | Not related to immunogenicity, efficacy, and safety of TB/Malaria vaccine candidate |
|  | Mbanefo et al., | 2017 | Association of glucose-6-phosphate dehydrogenase deficiency and malaria: a systematic review and meta-analysis | Not related to immunogenicity, efficacy, and safety of TB/Malaria vaccine candidate |
|  | Huang et al., | 2017 | Concomitant nevirapine impacts pharmacokinetic exposure to the antimalarial artemether-lumefantrine in African children | Not related to immunogenicity, efficacy, and safety of TB/Malaria vaccine candidate |
|  | Okell et al., | 2017 | Mapping sulphadoxine-pyrimethamine-resistant Plasmodium falciparum malaria in infected humans and in parasite populations in Africa | Not related to immunogenicity, efficacy, and safety of TB/Malaria vaccine candidate |
|  | Souza et al., | 2017 | Pre-travel malaria chemoprophylaxis counselling in a public travel medicine clinic in Sao Paulo, Brazil | Not related to immunogenicity, efficacy, and safety of TB/Malaria vaccine candidate |
|  | Varo et al., | 2017 | Safety and tolerability of adjunctive rosiglitazone treatment for children with uncomplicated malaria | Not related to immunogenicity, efficacy, and safety of TB/Malaria vaccine candidate |
|  | Benbow et al., | 2017 | Buruli Ulcer: Case Study of a Neglected Tropical Disease | Non-TB/malaria study |
|  | Kulkarni et al., | 2017 | Examination of Antibody Responses as a Measure of Exposure to Malaria in the Indigenous Batwa and Their Non-Indigenous Neighbors in Southwestern Uganda | Not related to immunogenicity, efficacy, and safety of TB/Malaria vaccine candidate |
|  | Warsame et al., | 2017 | Efficacy of artesunate plus sulphadoxine/pyrimethamine and artemether plus lumefantrine and dhfr and dhps mutations in Somalia: evidence for updating the malaria treatment policy | Not related to immunogenicity, efficacy, and safety of TB/Malaria vaccine candidate |
|  | Wotodjo et al., | 2017 | Malaria in Dielmo, a Senegal village: Is its elimination possible after seven years of implementation of long-lasting insecticide-treated nets? | Not related to immunogenicity, efficacy, and safety of TB/Malaria vaccine candidate |
|  | Herzmann et al., | 2017 | Pulmonary immune responses to Mycobacterium tuberculosis in exposed individuals | Not related to immunogenicity, efficacy, and safety of TB/Malaria vaccine candidate |
|  | Dheda et al., | 2017 | Outcomes, infectiousness, and transmission dynamics of patients with extensively drug-resistant tuberculosis and home-discharged patients with programmatically incurable tuberculosis: a prospective cohort study | Not related to immunogenicity, efficacy, and safety of TB/Malaria vaccine candidate |
|  | Lu et al., | 2017 | Return of chloroquine sensitivity to Africa? Surveillance of African Plasmodium falciparum chloroquine resistance through malaria imported to China | Not related to immunogenicity, efficacy, and safety of TB/Malaria vaccine candidate |
|  | Srivastava et al., | 2017 | Linezolid Dose That Maximizes Sterilizing Effect While Minimizing Toxicity and Resistance Emergence for Tuberculosis | Not related to immunogenicity, efficacy, and safety of TB/Malaria vaccine candidate |
|  | Bastos et al., | 2017 | An updated systematic review and meta-analysis for treatment of multidrug-resistant tuberculosis | Not related to immunogenicity, efficacy, and safety of TB/Malaria vaccine candidate |
|  | Idris et al., | 2017 | Naturally acquired antibody response to Plasmodium falciparum describes heterogeneity in transmission on islands in Lake Victoria | Not related to immunogenicity, efficacy, and safety of TB/Malaria vaccine candidate |
|  | Plucinski et al., | 2017 | Efficacy of artemether-lumefantrine, artesunate-amodiaquine, and dihydroartemisinin-piperaquine for treatment of uncomplicated Plasmodium falciparum malaria in Angola, 2015 | Not related to immunogenicity, efficacy, and safety of TB/Malaria vaccine candidate |
|  | Kassegne et al., | 2017 | Study roadmap for high-throughput development of easy to use and affordable biomarkers as diagnostics for tropical diseases: a focus on malaria and schistosomiasis | Not related to immunogenicity, efficacy, and safety of TB/Malaria vaccine candidate |
|  | Bruxvoort et al., | 2017 | The Impact of Introducing Malaria Rapid Diagnostic Tests on Fever Case Management: A Synthesis of Ten Studies from the ACT Consortium | Not related to immunogenicity, efficacy, and safety of TB/Malaria vaccine candidate |
|  | Strickland et al., | 2017 | Characterization of Mycobacterium tuberculosis-Specific Cells Using MHC Class II Tetramers Reveals Phenotypic Differences Related to HIV Infection and Tuberculosis Disease | Not related to immunogenicity, efficacy, and safety of TB/Malaria vaccine candidate |
|  | O’Hara et al., | 2017 | Infection control and tuberculosis in health care workers: an assessment of 28 hospitals in South Africa | Not related to immunogenicity, efficacy, and safety of TB/Malaria vaccine candidate |
|  | Gengenbacher et al., | 2017 | NOS2-deficient mice with hypoxic necrotizing lung lesions predict outcomes of tuberculosis chemotherapy in humans | Not related to immunogenicity, efficacy, and safety of TB/Malaria vaccine candidate |
|  | Yerramilli et al., | 2017 | The location of Australian Buruli ulcer lesions-Implications for unravelling disease transmission | Non-TB/malaria study |
|  | Kumar et al., | 2017 | Mycobacterium abscessus L, D-Transpeptidases Are Susceptible to Inactivation by Carbapenems and Cephalosporins but Not Penicillins | Non-TB/malaria study |
|  | Roh et al., | 2017 | Protective Effect of Indoor Residual Spraying of Insecticide on Preterm Birth Among Pregnant Women with HIV Infection in Uganda: A Secondary Data Analysis | Not related to immunogenicity, efficacy, and safety of TB/Malaria vaccine candidate |
|  | Parent et al., | 2017 | Participatory theatre and tuberculosis: a feasibility study with South African health care workers | Not related to immunogenicity, efficacy, and safety of TB/Malaria vaccine candidate |
|  | Bayili et al., | 2017 | Evaluation of efficacy of Interceptor (R) G2, a long-lasting insecticide net coated with a mixture of chlorfenapyr and alpha-cypermethrin, against pyrethroid resistant Anopheles gambiae s.l. in Burkina Faso | Not related to immunogenicity, efficacy, and safety of TB/Malaria vaccine candidate |
|  | Tocko-Marabena et al., | 2017 | Genetic diversity of Bemisia tabaci species colonizing cassava in Central African Republic characterized by analysis of cytochrome c oxidase subunit I | Non-TB/malaria study |
|  | Ogoma et al., | 2017 | A low technology emanator treated with the volatile pyrethroid transfluthrin confers long term protection against outdoor biting vectors of lymphatic filariasis, arboviruses and malaria | Not related to immunogenicity, efficacy, and safety of TB/Malaria vaccine candidate |
|  | Mohapatra et al., | 2017 | HIV-malaria interactions in North-East India: A prospective cohort study | Non-TB/malaria study |
|  | Ngondi et al., | 2017 | Surveillance for sulfadoxine-pyrimethamine resistant malaria parasites in the Lake and Southern Zones, Tanzania, using pooling and next-generation sequencing | Not related to immunogenicity, efficacy, and safety of TB/Malaria vaccine candidate |
|  | Masalu et al., | 2017 | Efficacy and user acceptability of transfluthrin-treated sisal and hessian decorations for protecting against mosquito bites in outdoor bars | Not related to immunogenicity, efficacy, and safety of TB/Malaria vaccine candidate |
|  | Newton et al., | 2017 | Do anti-malarials in Africa meet quality standards? The market penetration of non-quality-assured artemisinin combination therapy in eight African countries | Not related to immunogenicity, efficacy, and safety of TB/Malaria vaccine candidate |
|  | Kozycki et al., | 2017 | False-negative malaria rapid diagnostic tests in Rwanda: impact of Plasmodium falciparum isolates lacking hrp2 and declining malaria transmission | Not related to immunogenicity, efficacy, and safety of TB/Malaria vaccine candidate |
|  | Fox et al., | 2017 | Group 5 drugs for multidrug-resistant tuberculosis: individual patient data meta-analysis | Not related to immunogenicity, efficacy, and safety of TB/Malaria vaccine candidate |
|  | Hershey et al., | 2017 | Malaria Control Interventions Contributed to Declines in Malaria Parasitemia, Severe Anemia, and All-Cause Mortality in Children Lesst han 5 Years of Age in Malawi, 2000-2010 | Not related to immunogenicity, efficacy, and safety of TB/Malaria vaccine candidate |
|  | Osman et al., | 2017 | Excellent Treatment Outcomes in Children Treated for Tuberculosis Under Routine Operational Conditions in Cape Town, South Africa | Not related to immunogenicity, efficacy, and safety of TB/Malaria vaccine candidate |
|  | Tiwari et al., | 2017 | Fast and efficient detection of tuberculosis antigens using liposome encapsulated secretory proteins of Mycobacterium tuberculosis | Not related to immunogenicity, efficacy, and safety of TB/Malaria vaccine candidate |
|  | Loy et al., | 2017 | Out of Africa: origins and evolution of the human malaria parasites Plasmodium falciparum and Plasmodium vivax | Not related to immunogenicity, efficacy, and safety of TB/Malaria vaccine candidate |
|  | Jemmott et al., | 2018 | Effect of a Behavioral Intervention on Perpetrating and Experiencing Forced Sex among South African Adolescents: A Secondary Analysis of a Cluster Randomized Trial | Not related to immunogenicity, efficacy, and safety of TB/Malaria vaccine candidate |
|  | Chidimatembue et al., | 2021 | Molecular surveillance for polymorphisms associated with artemisinin-based combination therapy resistance in Plasmodium falciparum isolates collected in Mozambique, 2018 | Not related to immunogenicity, efficacy, and safety of TB/Malaria vaccine candidate |
|  | Kimenyi et al., | 2022 | Maintenance of high temporal Plasmodium falciparum genetic diversity and complexity of infection in asymptomatic and symptomatic infections in Kilifi, Kenya from 2007 to 2018 | Not related to immunogenicity, efficacy, and safety of TB/Malaria vaccine candidate |
|  | Mabweazara et al., | 2018 | A six-week contextualised physical activity intervention for women living with HIV and AIDS of low socioeconomic status: a pilot study | Non-TB/malaria study |
|  | Galárraga et al., | 2018 | The empower nudge lottery to increase dual protection use: A proof-of-concept randomised pilot trial in South Africa | Non-TB/malaria study |
|  | Cockcroft et al., | 2018 | Access of choice-disabled young women in Botswana to government structural support programmes: a cross-sectional study | Non-TB/malaria study |
|  | Pacheco et al., | 2020 | Changes in the frequencies of Plasmodium falciparum dhps and dhfr drug-resistant mutations in children from Western Kenya from 2005 to 2018: the rise of Pfdhps S436H | Not related to immunogenicity, efficacy, and safety of TB/Malaria vaccine candidate |
|  | Speizer et al., | 2018 | Changing sex risk behaviors, gender norms, and relationship dynamics among couples in Cape Town, South Africa: Efficacy of an intervention on the dyad | Non-TB/malaria study |
|  | Cutland et al., | 2018 | Immunogenicity and safety of one or two doses of the quadrivalent meningococcal vaccine MenACWY-TT given alone or with the 13-valent pneumococcal conjugate vaccine in toddlers: A phase III, open-label, randomised study | Non-TB/malaria study |
|  | Van reijen et al., | 2018 | Preventing recurrent ankle sprains: Is the use of an App more cost-effective than a printed Booklet? Results of a RCT | Non-TB/malaria study |
|  | Mugwanya et al., | 2018 | Scale up of PrEP integrated in public health HIV care clinics: A protocol for a stepped-wedge cluster-randomized rollout in Kenya | Non-TB/malaria study |
|  | Lion et al., | 2018 | The effect of the "Follow in my Green Food Steps" programme on cooking behaviours for improved iron intake: A quasi-experimental randomized community study | Non-TB/malaria study |
|  | van der Kop et al., | 2018 | Effect of an interactive text-messaging service on patient retention during the first year of HIV care in Kenya (WelTel Retain): an open-label, randomised parallel-group study | Non-TB/malaria study |
|  | Burke et al., | 2018 | Effect of self-administration versus provider-administered injection of subcutaneous depot medroxyprogesterone acetate on continuation rates in Malawi: a randomised controlled trial | Non-TB/malaria study |
|  | Hespanhol et al., | 2018 | Effectiveness of online tailored advice to prevent running-related injuries and promote preventive behaviour in Dutch trail runners: a pragmatic randomised controlled trial | Non-TB/malaria study |
|  | Källander et al., | 2018 | Universal versus conditional day 3 follow-up for children with non-severe unclassified fever at the community level in Ethiopia: A cluster-randomised non-inferiority trial | Non-TB/malaria study |
|  | Floyd et al., | 2018 | Towards 90-90: Findings after two years of the HPTN 071 (PopART) cluster-randomized trial of a universal testing-and-treatment intervention in Zambia | Non-TB/malaria study |
|  | Wernette et al., | 2018 | A Pilot Randomized Controlled Trial of a Computer-Delivered Brief Intervention for Substance Use and Risky Sex during Pregnancy | Non-TB/malaria study |
|  | Mwangi et al., | 2018 | Effectiveness of peer support to increase uptake of retinal examination for diabetic retinopathy: Study protocol for the DURE pragmatic cluster randomized clinical trial in Kirinyaga, Kenya | Non-TB/malaria study |
|  | Schmedes et al., | 2021 | Plasmodium falciparum kelch 13 Mutations, 9 Countries in Africa 2014-2018 | Not related to immunogenicity, efficacy, and safety of TB/Malaria vaccine candidate |
|  | Chung et al., | 2020 | Scaling up malaria elimination management and leadership: a pilot in three provinces in Zimbabwe, 2016-2018 | Not related to immunogenicity, efficacy, and safety of TB/Malaria vaccine candidate |
|  | Mpande et al., | 2021 | Antigen-Specific T-Cell Activation Distinguishes between Recent and Remote Tuberculosis Infection | Not related to immunogenicity, efficacy, and safety of TB/Malaria vaccine candidate |
|  | Ronit et al., | 2018 | Serum Albumin as a Prognostic Marker for Serious Non-AIDS Endpoints in the Strategic Timing of Antiretroviral Treatment (START) Study | Non-TB/malaria study |
|  | Mallewa et al., | 2018 | Effect of ready-to-use supplementary food on mortality in severely immunocompromised HIV-infected individuals in Africa initiating antiretroviral therapy (REALITY): an open-label, parallel-group, randomised controlled trial | Non-TB/malaria study |
|  | Van der Straten | 2018 | The tablets, ring, injections as options (TRIO) study: What young African women chose and used for future HIV and pregnancy prevention | Non-TB/malaria study |
|  | Evbuomwan et al., | 2018 | A prospective study comparing 99m Tc-MIBI and 99m Tc-MDP with 99m Tc-DTPA for lung ventilation scintigraphy in pulmonary thromboembolism | Non-TB/malaria study |
|  | Goldstein et al., | 2018 | Doctors' perceptions of the impact of upfront point-of-care testing in the emergency department | Non-TB/malaria study |
|  | Boateng et al., | 2018 | Improving Blood Retinol Concentrations with Complementary Foods Fortified with Moringa oleifera Leaf Powder - A Pilot Study | Non-TB/malaria study |
|  | Urayeneza et al., | 2018 | Increasing evidence-based interventions in patients with acute infections in a resource-limited setting: a before-and-after feasibility trial in Gitwe, Rwanda | Non-TB/malaria study |
|  | Negussie et al., | 2018 | Lymphoedema management to prevent acute dermatolymphangioadenitis in podoconiosis in northern Ethiopia (GoLBeT): a pragmatic randomised controlled trial | Non-TB/malaria study |
|  | Zelellw et al., | 2018 | Level of partograph utilization and its associated factors among obstetric caregivers at public health facilities in East Gojam Zone, Northwest Ethiopia | Non-TB/malaria study |
|  | Kilburn et al., | 2018 | Conditional cash transfers and the reduction in partner violence for young women: An investigation of causal pathways using evidence from a randomized experiment in South Africa (HPTN 068): An | Non-TB/malaria study |
|  | Knox etal., | 2018 | Six hours of task-oriented training optimizes walking competency post stroke: a randomized controlled trial in the public health-care system of South Africa | Non-TB/malaria study |
|  | Anders et al., | 2018 | Cluster-randomized test-negative design trials: A novel and efficient method to assess the efficacy of community-level dengue interventions | Non-TB/malaria study |
|  | Cavallin et al., | 2018 | Thermal effect of a woolen cap in low-birth-weight infants during kangaroo care | Non-TB/malaria study |
|  | Aggerbeck et al., | 2018 | C-Tb skin test to diagnose Mycobacterium tuberculosis infection in children and HIV-infected adults: A phase 3 trial | Not related to immunogenicity, efficacy, and safety of TB/Malaria vaccine candidate |
|  | Hendriksz et al., | 2018 | Impact of long-term elosulfase alfa on activities of daily living in patients with Morquio A syndrome in an open-label, multi-center, phase 3 extension study | Non-TB/malaria study |
|  | Delany-Moretlwe et al., | 2018 | Tenofovir 1% vaginal gel for prevention of HIV-1 infection in women in South Africa (FACTS-001): a phase 3, randomised, double-blind, placebo-controlled trial | Non-TB/malaria study |
|  | Sarfo et al., | 2018 | PINGS (Phone-based intervention under nurse guidance after stroke) interim results of a pilot randomized controlled trial | Non-TB/malaria study |
|  | Maharaj and Yakasai | 2018 | Does a Rehabilitation Program of Aerobic and Progressive Resisted Exercises Influence HIV-Induced Distal Neuropathic Pain? | Non-TB/malaria study |
|  | Ssenyonga et al., | 2018 | Reducing violence against children by implementing the preventative intervention Interaction Competencies with Children for Teachers (ICC-T): Study protocol for a cluster randomized controlled trial in Southwestern Uganda | Non-TB/malaria study |
|  | Brown et al., | 2018 | Mitigating intimate partner violence among South African women testing HIV positive during mobile counseling and testing | Non-TB/malaria study |
|  | Herrero et al., | 2018 | Can supportive parenting protect against school delay amongst violence-exposed adolescents in South Africa? | Non-TB/malaria study |
|  | Rada et al., | 2018 | Concordance of three alternative gestational age assessments for pregnant women from four African countries: A secondary analysis of the MIPPAD trial | Non-TB/malaria study |
|  | Klop et al., | 2018 | Learning new words from an interactive electronic storybook intervention | Non-TB/malaria study |
|  | Heeren et al., | 2018 | Health-Promotion Intervention Increases Self-Reported Physical Activity in Sub-Saharan African University Students: A Randomized Controlled Pilot Study | Non-TB/malaria study |
|  | Kon et al., | 2018 | A multilayer biomaterial for osteochondral regeneration shows superiority vs microfractures for the treatment of osteochondral lesions in a multicentre randomized trial at 2 years | Non-TB/malaria study |
|  | Joag et al., | 2018 | Ex vivo HIV entry into blood CD4+ T cells does not predict heterosexual HIV acquisition in women | Non-TB/malaria study |
|  | Bekker et al., | 2018 | Subtype C ALVAC-HIV and bivalent subtype C gp120/MF59 HIV-1 vaccine in low-risk, HIV-uninfected, South African adults: a phase 1/2 trial | Non-TB/malaria study |
|  | Nel et al., | 2018 | Safety of a silicone elastomer vaginal ring as potential microbicide delivery method in African women: A Phase 1 randomized trial | Non-TB/malaria study |
|  | Corten et al., | 2018 | Assisted autogenic drainage in infants and young children hospitalized with uncomplicated pneumonia, a pilot study | Non-TB/malaria study |
|  | Clark et al., | 2018 | Labial tissue rejuvenation and sexual function improvement using a novel noninvasive focused monopolar radio frequency device | Non-TB/malaria study |
|  | Makanani et al., | 2018 | Pregnancy and infant outcomes among women using the dapivirine vaginal ring in early pregnancy | Non-TB/malaria study |
|  | Tiono et al., | 2018 | Efficacy of Olyset Duo, a bednet containing pyriproxyfen and permethrin, versus a permethrin-only net against clinical malaria in an area with highly pyrethroid-resistant vectors in rural Burkina Faso: a cluster-randomised controlled trial | Not related to immunogenicity, efficacy, and safety of TB/Malaria vaccine candidate |
|  | Muthengi and Austrian | 2018 | Cluster randomized evaluation of the Nia Project: Study protocol | Non-TB/malaria study |
|  | Johansen et al., | 2018 | A comparative evaluation of PDQ-Evidence | Non-TB/malaria study |
|  | Adachi et al., | 2018 | Congenital Cytomegalovirus and HIV Perinatal Transmission | Non-TB/malaria study |
|  | Olotu et al., | 2018 | Advancing global health through development and clinical trials partnerships: A randomized, placebo-controlled, double-blind assessment of safety, tolerability, and immunogenicity of pfspz vaccine for malaria in healthy equatoguinean men | Non-TB/malaria study |
|  | Chen et al., | 2018 | Safety and immunogenicity of a pentavalent meningococcal conjugate vaccine containing serogroups A, C, Y, W, and X in healthy adults: a phase 1, single-centre, double-blind, randomised, controlled study | Non-TB/malaria study |
|  | Brown et al., | 2018 | Whey protein hydrolysate supplementation accelerates recovery from exercise-induced muscle damage in females | Non-TB/malaria study |
|  | Lambrano et al., | 2019 | Preliminary Evaluation of the Safety and Immunogenicity of an Antimalarial Vaccine Candidate Modified Peptide (IMPIPS) Mixture in a Murine Model | Pre-clinical study |
|  | Opoku and Nyanor et al., | 2019 | Qualitative and Quantitative Microbiological Studies of Paediatric Artemether-Lumefantrine Dry Powders and Paracetamol Syrups Obtained from Selected Drug Stores in Accra, Ghana | Non-TB/malaria study |
|  | Kirinyet | 2019 | An Assessment of Malaria Parasite Density among HIV/AIDS-Subjects at Different Levels of CD4 T-Cells Prior to Antimalarial Therapy at Chulaimbo Sub-County Hospital, Western Kenya | Non-TB/malaria study |
|  | Lee et al., | 2019 | Comparison of Major Adverse Cardiac Events between Instantaneous Wave-Free Ratio and Fractional Flow Reserve-Guided Strategy in Patients with or Without Type 2 Diabetes: A Secondary Analysis of a Randomized Clinical Trial | Non-TB/malaria study |
|  | Byrnes et al., | 2019 | Within trial cost-utility analysis of disease management program for patients hospitalized with atrial fibrillation: results from the SAFETY trial | Non-TB/malaria study |
|  | Beksinska, et al., | 2019 | Functional performance study of an adapted design of the woman’s condom: a crossover, noninferiority, randomized clinical trial | Non-TB/malaria study |
|  | Sugarman et al., | 2019 | Preventive Misconception and Risk Behaviors in a Multinational HIV Prevention Trial | Non-TB/malaria study |
|  | Kiravu et al., | 2019 | Bacille Calmette-Guerin Vaccine Strain Modulates the Ontogeny of Both Mycobacterial-Specific and Heterologous T Cell Immunity to Vaccination in Infants | Randomisation not clear |
|  | Mouton et al., | 2019 | Comprehensive Characterization of the Attenuated Double Auxotroph Mycobacterium tuberculosis Delta leuD Delta panCD as an Alternative to H37Rv | Not related to immunogenicity, efficacy, and safety of TB/Malaria vaccine candidate |
|  | Frimpong et al., | 2019 | Phenotypic Evidence of T Cell Exhaustion and Senescence During Symptomatic Plasmodium falciparum Malaria | Not related to immunogenicity, efficacy, and safety of TB/Malaria vaccine candidate |
|  | Kivisi et al., | 2019 | Exploring Plasmodium falciparum Var Gene Expression to Assess Host Selection Pressure on Parasites During Infancy | Not related to immunogenicity, efficacy, and safety of TB/Malaria vaccine candidate |
|  | Tebruegge | 2019 | Mycobacteria-Specific Mono- and Polyfunctional CD4+T Cell Profiles in Children with Latent and Active Tuberculosis: Prospective Proof-of-Concept Study | Not related to immunogenicity, efficacy, and safety of TB/Malaria vaccine candidate |
|  | Villa et al., | 2019 | Tuberculosis among asylum seekers in Milan, Italy: epidemiological analysis and evaluation of interventions | Not related to immunogenicity, efficacy, and safety of TB/Malaria vaccine candidate and non-sub-Saharan Africa study |
|  | Nagaoka et al., | 2019 | The N-Terminal Region of Plasmodium falciparum MSP10 Is a Target of Protective Antibodies in Malaria and Is Important for PfGAMA/PfMSP10 Interaction | Not related to immunogenicity, efficacy, and safety of TB/Malaria vaccine candidate |
|  | Ca et al., | 2019 | Experimental Evidence for Limited in vivo Virulence of Mycobacterium africanum | Not related to immunogenicity, efficacy, and safety of TB/Malaria vaccine candidate |
|  | von Groote-Bidlingmaier et al., | 2019 | Efficacy and safety of delamanid in combination with an optimised background regimen for treatment of multidrug-resistant tuberculosis: a multicentre, randomised, double-blind, placebo-controlled, parallel group phase 3 trial | Not related to immunogenicity, efficacy, and safety of TB/Malaria vaccine candidate |
|  | Chileshe et al., | 2019 | An interferon-gamma release assay for the diagnosis of the Mycobacterium bovis infection in white rhinoceros (Ceratotherium simum) | Not related to immunogenicity, efficacy, and safety of TB/Malaria vaccine candidate |
|  | Vinhaes et al., | 2019 | Changes in inflammatory protein and lipid mediator profiles persist after antitubercular treatment of pulmonary and extrapulmonary tuberculosis: A prospective cohort study | Not related to immunogenicity, efficacy, and safety of TB/Malaria vaccine candidate |
|  | Mashabela et al., | 2019 | Mycobacterium tuberculosis Metabolism | Not related to immunogenicity, efficacy, and safety of TB/Malaria vaccine candidate |
|  | der Westhuizen et al., | 2019 | The high-quality health system `revolution': Re-imagining tuberculosis infection prevention and control | Not related to immunogenicity, efficacy, and safety of TB/Malaria vaccine candidate |
|  | Ciccacci et al., | 2019 | Plasma levels of CRP, neopterin and IP-10 in HIV-infected individuals with and without pulmonary tuberculosis | Not related to immunogenicity, efficacy, and safety of TB/Malaria vaccine candidate |
|  | Kendall et al., | 2019 | Phonomotor versus semantic feature analysis treatment for anomia in 58 persons with aphasia: A randomized controlled trial | Non-TB/malaria study |
|  | Chiliza et al., | 2019 | Immunoscreening of the M-tuberculosis F15/LAM4/KZN secretome library against TB patients' sera identifies unique active- and latent-TB specific biomarkers | Not related to immunogenicity, efficacy, and safety of TB/Malaria vaccine candidate |
|  | Thanapongpichat et al., | 2019 | Genetic Diversity of Plasmodium vivax in Clinical Isolates from Southern Thailand using PvMSP1, PvMSP3 (PvMSP3 alpha, PvMSP3 beta) Genes and Eight Microsatellite Markers | Not related to immunogenicity, efficacy, and safety of TB/Malaria vaccine candidate and non-sub-Saharan African study |
|  | Miller et al., | 2019 | Serological reactivity to MPB83 and CFP10/ESAT-6 antigens in three suid hosts of Mycobacterium bovis infection | Not related to immunogenicity, efficacy, and safety of TB/Malaria vaccine candidate |
|  | Jo et al., | 2019 | Resistance training during a 12-week protein supplemented VLCD treatment enhances weight-loss outcomes in obese patients | Non-TB/malaria study |
|  | Brown et al., | 2019 | Montmorency tart cherry (Prunus cerasus L.) supplementation accelerates recovery from exercise-induced muscle damage in females | Non-TB/malaria study |
|  | Blagrove et al., | 2019 | Efficacy of depth jumps to elicit a post-activation performance enhancement in junior endurance runners | Non-TB/malaria study |
|  | Zlotnick et al., | 2019 | A randomized controlled trial of a computer-based brief intervention for victimized perinatal women seeking mental health treatment | Non-TB/malaria study |
|  | Van’T et al., | 2019 | Using photo stories to support doctor-patient communication: Evaluating a communicative health literacy intervention for older adults | Non-TB/malaria study |
|  | Gopalan et al., | 2019 | Use of financial incentives and text message feedback to increase healthy food purchases in a grocery store cash back program: A randomized controlled trial | Non-TB/malaria study |
|  | Ahmed et al., | 2019 | HIV incidence among women using intramuscular depot medroxyprogesterone acetate, a copper intrauterine device, or a levonorgestrel implant for contraception: a randomised, multicentre, open-label trial | Non-TB/malaria study |
|  | Sarnquist et al., | 2019 | A protocol for a cluster-randomized controlled trial testing an empowerment intervention to prevent sexual assault in upper primary school adolescents in the informal settlements of Nairobi, Kenya | Non-TB/malaria study |
|  | Minnaar et al., | 2019 | The effect of modulated electro-hyperthermia on local disease control in HIV-positive and -negative cervical cancer women in South Africa: Early results from a phase III randomised controlled trial | Non-TB/malaria study |
|  | Wright et al., | 2019 | Effect of A Sun Protection Intervention on the Immune Response to Measles Booster Vaccination in Infants in Rural South Africa | Non-TB/malaria study |
|  | Sabben et al., | 2019 | A Smartphone Game to Prevent HIV Among Young Africans (Tumaini): Assessing Intervention and Study Acceptability Among Adolescents and Their Parents in a Randomized Controlled Trial. | Non-TB/malaria study |
|  | Magai et al., | 2019 | A randomized control trial of phototherapy and 20% albumin versus phototherapy and saline in Kilifi, Kenya | Non-TB/malaria study |
|  | McCollum et al., | 2019 | Bubble continuous positive airway pressure for children with high-risk conditions and severe pneumonia in Malawi: an open label, randomised, controlled trial | Non-TB/malaria study |
|  | Darbes et al., | 2019 | Results of a couples-based randomized controlled trial aimed to increase testing for HIV | Non-TB/malaria study |
|  | Ageru et al., | 2019 | Anemia and its associated factors among adult people living with human immunodeficiency virus at Wolaita Sodo University teaching referral hospital | Non-TB/malaria study |
|  | Mbabazi-Kabachelor et al., | 2019 | Infection risk for bactiseal universal shunts versus chhabra shunts in ugandan infants: A randomized controlled trial | Non-TB/malaria study |
|  | Harding et al., | 2019 | Improving psychological outcomes for orphans living with HIV in Tanzania through a novel intervention to improve resilience: findings from a pilot RCT | Non-TB/malaria study |
|  | Al-Hweish et al., | 2019 | Tidal peritoneal dialysis versus ultrafiltration in type 1 cardiorenal syndrome: A prospective randomized study | Non-TB/malaria study |
|  | Naidoo et al., | 2019 | Does the use of adjunct urine lipopolysaccharide lipoarabinomannan in HIV-infected hospitalized patients reduce the utilization of healthcare resources? A post hoc analysis of the LAM multi-country randomized controlled trial | Non-TB/malaria study |
|  | Botek et al., | 2019 | Hydrogen Rich Water Improved Ventilatory, Perceptual and Lactate Responses to Exercise | Non-TB/malaria study |
|  | Aggerbeck et al., | 2019 | Interaction between C-Tb and PPD given concomitantly in a split-body randomised controlled trial |  |
|  | Bonney et al., | 2019 | Benefits of Activity-Based Interventions among Female Adolescents Who Are Overweight and Obese | Non-TB/malaria study |
|  | Montgomery et al., | 2019 | Social harms in female-initiated HIV prevention method research: State of the evidence | Non-TB/malaria study |
|  | Zulaika et al., | 2019 | Menstrual cups and cash transfer to reduce sexual and reproductive harm and school dropout in adolescent schoolgirls: Study protocol of a cluster-randomised controlled trial in western Kenya | Non-TB/malaria study |
|  | Church et al., | 2019 | Neonatal Vitamin A supplementation and immune responses to oral polio vaccine in Zimbabwean infants | Non-TB/malaria study |
|  | Robertson et al., | 2019 | Human Immunodeficiency Virus Type 1 and Tuberculosis Coinfection in Multinational, Resource-limited Settings: Increased neurological dysfunction | Not related to immunogenicity, efficacy, and safety of TB/Malaria vaccine candidate |
|  | Gray et al., | 2019 | Immune correlates of the Thai RV144 HIV vaccine regimen in South Africa | Non-TB/malaria study |
|  | van de Hoef et al., | 2019 | Does a bounding exercise program prevent hamstring injuries in adult male soccer players? – A cluster-RCT | Non-TB/malaria study |
|  | Matrevi et al., | 2019 | Plasmodium falciparum Kelch Propeller Polymorphisms in Clinical Isolates from Ghana from 2007 to 2016 | Not related to immunogenicity, efficacy, and safety of TB/Malaria vaccine candidate |
|  | Lyon s | 2019 | Modeling and Simulation of Pretomanid Pharmacodynamics in Pulmonary Tuberculosis Patients | Not related to immunogenicity, efficacy, and safety of TB/Malaria vaccine candidate |
|  | Rodo et al., | 2019 | A comparison of antigen-specific T cell responses induced by six novels tuberculosis vaccine candidates | Not related to immunogenicity, efficacy, and safety of TB/Malaria vaccine candidate |
|  | Steiner-Monard et al., | 2019 | The Candidate Blood-stage Malaria Vaccine P27A Induces a Robust Humoral Response in a Fast Track to the Field Phase 1 Trial in Exposed and Nonexposed Volunteers | Unclear randomisation process |
|  | Akinyotu et al., | 2019 | A randomized controlled trial of azithromycin and sulphadoxine-pyrimethamine as prophylaxis against malaria in pregnancy among human immunodeficiency virus-positive women | Not related to immunogenicity, efficacy, and safety of TB/Malaria vaccine candidate |
|  | Zhang et al., | 2019 | Immunogenicity analysis of genetically conserved segments in Plasmodium ovale merozoite surface protein-8 | Not related to immunogenicity, efficacy, and safety of TB/Malaria vaccine candidate |
|  | He et al., | 2019 | Antibody responses to Plasmodium vivax Duffy binding and Erythrocyte binding proteins predict risk of infection and are associated with protection from clinical Malaria | Not related to immunogenicity, efficacy, and safety of TB/Malaria vaccine candidate |
|  | Raman et al., | 2019 | Safety and tolerability of single low-dose primaquine in a low-intensity transmission area in South Africa: an open label, randomized controlled trial | Not related to immunogenicity, efficacy, and safety of TB/Malaria vaccine candidate |
|  | Wasserman et al., | 2019 | Linezolid Pharmacokinetics in South African Patients with Drug-Resistant Tuberculosis and a High Prevalence of HIV Coinfection | Not related to immunogenicity, efficacy, and safety of TB/Malaria vaccine candidate |
|  | Tweed et al., | 2019 | Toxicity related to standard TB therapy for pulmonary tuberculosis and treatment outcomes in the REMoxTB study according to HIV status | Not related to immunogenicity, efficacy, and safety of TB/Malaria vaccine candidate |
|  | Silva et al., | 2019 | Plasmodium falciparum K13 expression associated with parasite clearance during artemisinin-based combination therapy | Not related to immunogenicity, efficacy, and safety of TB/Malaria vaccine candidate |
|  | Venkatesh et al., | 2019 | Hospital-derived antibody profiles of malaria patients in Southwest India | Not related to immunogenicity, efficacy, and safety of TB/Malaria vaccine candidate |
|  | Sanou et al., | 2019 | Evaluation of mosquito electrocuting traps as a safe alternative to the human landing catch for measuring human exposure to malaria vectors in Burkina Faso | Not related to immunogenicity, efficacy, and safety of TB/Malaria vaccine candidate |
|  | Huangfu et al., | 2019 | Point of care HbA(1c) level for diabetes mellitus management and its accuracy among tuberculosis patients: a study in four countries | Non-TB/malaria study |
|  | Harries et al., | 2019 | Challenges and opportunities to prevent tuberculosis in people living with HIV in low-income countries | Not related to immunogenicity, efficacy, and safety of TB/Malaria vaccine candidate |
|  | Von Seidlein | 2019 | The Advanced Development Pathway of the RTS, S/AS01 Vaccine | Not related to immunogenicity, efficacy, and safety of TB/Malaria vaccine candidate |
|  | Davies et al., | 2019 | Effectiveness of 24-h mobile reporting tool during a malaria outbreak in Mpumalanga Province, South Africa | Not related to immunogenicity, efficacy, and safety of TB/Malaria vaccine candidate |
|  | Ghansah et al., | 2019 | Targeted Next Generation Sequencing for malaria research in Africa: current status and outlook | Not related to immunogenicity, efficacy, and safety of TB/Malaria vaccine candidate |
|  | Silveira-Mattos et al., | 2019 | Differential expression of CXCR3 and CCR6 on CD4(+) T-lymphocytes with distinct memory phenotypes characterizes tuberculosis-associated immune reconstitution inflammatory syndrome | Not related to immunogenicity, efficacy, and safety of TB/Malaria vaccine candidate |
|  | Goovaerts et al., | 2019 | Increased KLRG1 and PD-1 expression on CD8 T lymphocytes in TB-IRIS | Not related to immunogenicity, efficacy, and safety of TB/Malaria vaccine candidate |
|  | Tanner et al., | 2019 | In Vitro Efficacies, ADME, and Pharmacokinetic Properties of Phenoxazine Derivatives Active against Mycobacterium tuberculosis | Not related to immunogenicity, efficacy, and safety of TB/Malaria vaccine candidate |
|  | Sherrard-Smith et al., | 2019 | Mosquito feeding behavior and how it influences residual malaria transmission across Africa | Not related to immunogenicity, efficacy, and safety of TB/Malaria vaccine candidate |
|  | Hamid et al., | 2019 | Absence of K13 gene mutations among artesunate/sulfadoxine-pyrimethamine treatment failures of Sudanese Plasmodium falciparum isolates from Damazin, southeast Sudan | Not related to immunogenicity, efficacy, and safety of TB/Malaria vaccine candidate |
|  | Adolfi et al., | 2019 | Functional genetic validation of key genes conferring insecticide resistance in the major African malaria vector, Anopheles gambiae | Not related to immunogenicity, efficacy, and safety of TB/Malaria vaccine candidate |
|  | Selvaraj et al., | 2019 | Reducing malaria burden and accelerating elimination with long-lasting systemic insecticides: a modelling study of three potential use cases | Not related to immunogenicity, efficacy, and safety of TB/Malaria vaccine candidate |
|  | Huestis et al., | 2019 | Windborne long-distance migration of malaria mosquitoes in the Sahel | Not related to immunogenicity, efficacy, and safety of TB/Malaria vaccine candidate |
|  | Salvatore et al., | 2019 | Projecting the impact of variable MDR-TB transmission efficiency on long-term epidemic trends in South Africa and Vietnam | Not related to immunogenicity, efficacy, and safety of TB/Malaria vaccine candidate |
|  | Yang et al., | 2019 | Molecular investigation of the Pfmdr1 gene of Plasmodium falciparum isolates in Henan Province imported from Africa | Not related to immunogenicity, efficacy, and safety of TB/Malaria vaccine candidate |
|  | Mmbando et al., | 2019 | Evaluation of a push-pull system consisting of transfluthrin-treated eave ribbons and odour-baited traps for control of indoor- and outdoor-biting malaria vectors | Not related to immunogenicity, efficacy, and safety of TB/Malaria vaccine candidate |
|  | Ishengoma et al., | 2019 | Deployment and utilization of next-generation sequencing of Plasmodiumfalciparum to guide anti-malarial drug policy decisions in sub-Saharan Africa: opportunities and challenges | Not related to immunogenicity, efficacy, and safety of TB/Malaria vaccine candidate |
|  | Pacheco et al., | 2019 | Evolution and Genetic Diversity of the k13 Gene Associated with Artemisinin Delayed Parasite Clearance in Plasmodium falciparum | Not related to immunogenicity, efficacy, and safety of TB/Malaria vaccine candidate |
|  | Asua et al., | 2019 | Changing Molecular Markers of Antimalarial Drug Sensitivity across Uganda | Not related to immunogenicity, efficacy, and safety of TB/Malaria vaccine candidate |
|  | Elliot et al., | 2019 | Synergy and timing: a concurrent mass medical campaign predicted to augment indoor residual spraying for malaria | Not related to immunogenicity, efficacy, and safety of TB/Malaria vaccine candidate |
|  | Pett et al., | 2019 | CYP2D6 Polymorphisms and the Safety and Gametocytocidal Activity of Single-Dose Primaquine for Plasmodium falciparum | Not related to immunogenicity, efficacy, and safety of TB/Malaria vaccine candidate |
|  | Deshpande et al., | 2019 | Once-a-week tigecycline for the treatment of drug-resistant TB | Not related to immunogenicity, efficacy, and safety of TB/Malaria vaccine candidate |
|  | Verzier et al., | 2019 | Plasmodium knowlesi as a model system for characterising Plasmodium vivax drug resistance candidate genes | Not related to immunogenicity, efficacy, and safety of TB/Malaria vaccine candidate |
|  | Monroe et al., | 2019 | Human behaviour and residual malaria transmission in Zanzibar: findings from in-depth interviews and direct observation of community events | Not related to immunogenicity, efficacy, and safety of TB/Malaria vaccine candidate |
|  | Egyir et al., | 2019 | Susceptibility of Anopheles Mosquito to Agricultural Insecticides in the Adansi North District, Ghana | Not related to immunogenicity, efficacy, and safety of TB/Malaria vaccine candidate |
|  | Burke et al., | 2019 | Anopheles parensis contributes to residual malaria transmission in South Africa | Not related to immunogenicity, efficacy, and safety of TB/Malaria vaccine candidate |
|  | Raman et al., | 2019 | Absence of kelch13 artemisinin resistance markers but strong selection for lumefantrine-tolerance molecular markers following 18 years of artemisinin-based combination therapy use in Mpumalanga Province, South Africa (2001-2018) | Not related to immunogenicity, efficacy, and safety of TB/Malaria vaccine candidate |
|  | Rowhlwink et al., | 2019 | Tuberculous meningitis in children is characterized by compartmentalized immune responses and neural excitotoxicity | Not related to immunogenicity, efficacy, and safety of TB/Malaria vaccine candidate |
|  | Portugaliza et al., | 2019 | Examining community perceptions of malaria to inform elimination efforts in Southern Mozambique: a qualitative study | Not related to immunogenicity, efficacy, and safety of TB/Malaria vaccine candidate |
|  | Diouf et al., | 2019 | Association of high Plasmodium falciparum parasite densities with polyclonal microscopic infections in asymptomatic children from Toubacouta, Senegal | Not related to immunogenicity, efficacy, and safety of TB/Malaria vaccine candidate |
|  | Thawnashom et al., | 2019 | Validation of Plasmodium vivax centromere and promoter activities using Plasmodium yoelii | Not related to immunogenicity, efficacy, and safety of TB/Malaria vaccine candidate |
|  | Mazigo et al., | 2019 | Rice farmers' perceptions and acceptability in the use of a combination of biolarvicide (Bacillus thuringiensis var. israeliensis) and fertilizers application for malaria control and increase rice productivity in a rural district of central Tanzania | Non-TB/malaria study |
|  | Yoon et al., | 2019 | Yield and Efficiency of Novel Intensified Tuberculosis Case-Finding Algorithms for People Living with HIV | Non-TB/malaria study |
|  | Murungi et al., | 2019 | Serological Profiling for Malaria Surveillance Using a Standard ELISA  Protocol | Not related to immunogenicity, efficacy, and safety of TB/Malaria vaccine candidate |
|  | Amambua-Ngwa et al., | 2019 | Major subpopulations of Plasmodium falciparum in sub-Saharan Africa | Not related to immunogenicity, efficacy, and safety of TB/Malaria vaccine candidate |
[truncated: 142,741 more chars]
