# Supplementary material for: A systematic review on malaria and Tuberculosis (TB) vaccine challenges in sub-Saharan African clinical trials
Source: PLoS One. 2025 Jan 24;20(1):e0317233. doi: 10.1371/journal.pone.0317233 (PMC11760592; doi:10.1371/journal.pone.0317233)
Supplement: S2 Table — (DOCX) [file pone.0317233.s002.docx]

S2 Table: Eligible studies, extracted data, extractors, period of data extraction, and reasons for inclusion.

| Author | Year | Country | Vaccine | Control | Phase | No of doses | Participants | Main Findings | References | Data extractor | Period of data extraction | Eligibility reasons |
| --- | --- | --- | --- | --- | --- | --- | --- | --- | --- | --- | --- | --- |
| Tuberculosis (TB) | | | | | | | | | | | | |
| Churchyard et al., | 2015 | South Africa | AERAS- 402/A D35. TB-S | Placebo | II | 2 | Adults | Safe, CD4^+^, CD8^+^, antibodies | (27) | MH & AN | 1^st^ Sept-31^st^ Dec 2023 | Sub-Saharan study, clinical trial, publication year, immunogenicity, efficacy, or safety vaccination study, randomized study |
| Ndiaye et al., | 2015 | South Africa & Senegal | MVA 85A | Placebo | II | 2 | Adults | Safe, no protection, CD8^+^, CD4^+^ | (24) | MH & AN | 1^st^ Sept-31^st^ Dec 2023 | Sub-Saharan study, clinical trial, publication year, immunogenicity, efficacy, or safety vaccination study, randomized study |
| Meeren et al., | 2018 | Zambia, Kenya, South Africa | M72/ AS10 E | Placebo | II | 2 | Adults | 54% VE, safe | (32) | MH & AN | 1^st^ Sept-31^st^ Dec 2023 | Sub-Saharan study, clinical trial, publication year, immunogenicity, efficacy, or safety vaccination study, randomized study |
| Tait et al., | 2019 | Kenya, South Africa, & Zambia | M72/ AS01 E | Placebo | II | 2 | Adults | Safe, high CD4^+^, 49.7% VE, IgG | (38) | MH & AN | 1^st^ Sept-31^st^ Dec 2023 | Sub-Saharan study, clinical trial, publication year, immunogenicity, efficacy, or safety vaccination study, randomized study |
| Tameris et al., | 2019 | South Africa | MTBA V | BCG | I & II | 3 | Infants and adults | Higher CD4^+^, safe | (11) | MH & AN | 1^st^ Sept-31^st^ Dec 2023 | Sub-Saharan study, clinical trial, publication year, immunogenicity, efficacy, or safety vaccination study, randomized study |
| Walsh et al., | 2016 | Kenya | AERA S-402 | Placebo | I | 2 | Adults | Safe, modest CD4^+^ & CD8^+^ | (29) | MH & AN | 1^st^ Sept-31^st^ Dec 2023 | Sub-Saharan study, clinical trial, publication year, immunogenicity, efficacy, or safety vaccination study, randomized study |
| Tchakoute et al., | 2014 | South Africa | BCG | BCG | II | 1 | Infants | Higher CD4^+^ in delayed BCG | (39) | MH & AN | 1^st^ Sept-31^st^ Dec 2023 | Sub-Saharan study, clinical trial, publication year, immunogenicity, efficacy, or safety vaccination study, randomized study |
| Tameris et al., | 2015 | South Africa | AERA S-402 | Placebo | II | 3 | Infants | Safe, low CD4^+^ and CD8^+^ | (40) | MH & AN | 1^st^ Sept-31^st^ Dec 2023 | Sub-Saharan study, clinical trial, publication year, immunogenicity, efficacy, or safety vaccination study, randomized study |
| Penn-Nicholson et al., | 2018 | South Africa | ID93+  GLA- SE) | Placebo | I | 2 | Adults | Th1, IgG, safe | (41) | MH & AN | 1^st^ Sept-31^st^ Dec 2023 | Sub-Saharan study, clinical trial, publication year, immunogenicity, efficacy, or safety vaccination study, randomized study |
| Nell et al., | 2014 | South Africa | RUTI | Placebo | II | 2 | Adults | Safe, T-and humoral responses | (34) | MH & AN | 1^st^ Sept-31^st^ Dec 2023 | Sub-Saharan study, clinical trial, publication year, immunogenicity, efficacy, or safety vaccination study, randomized study |
| Lutwama et al., | 2014 | Uganda | BCG | Delayed | - | 1 | Infants | Higher CD4^+^ & CD8^+^ in early BCG | (42) | MH & AN | 1^st^ Sept-31^st^ Dec 2023 | Sub-Saharan study, clinical trial, publication year, immunogenicity, efficacy, or safety vaccination study, randomized study |
| Idoko et al., | 2014 | Gambia | M72/ AS01 | Meningitis vaccine | II | 2 | Infants | Safe, higher CD4^+^ & IgG | (31) | MH & AN | 1^st^ Sept-31^st^ Dec 2023 | Sub-Saharan study, clinical trial, publication year, immunogenicity, efficacy, or safety vaccination study, randomized study |
| Nemes et al., | 2018 | South Africa | H4: IC  31& r BCG | Placebo | II | 2 | Adolescent s | Safe, higher CD4^+^, low VE | (43) | MH & AN | 1^st^ Sept-31^st^ Dec 2023 | Sub-Saharan study, clinical trial, publication year, immunogenicity, efficacy, or safety vaccination study, randomized study |
| Suliman et al., | 2016 | South Africa | BCG | Non-vaccinated | I | 1 | Adults | Higher CD4, CD8, γδ T & NK cells | (44) | MH & AN | 1^st^ Sept-31^st^ Dec 2023 | Sub-Saharan study, clinical trial, publication year, immunogenicity, efficacy, or safety vaccination study, randomized study |
| Loxton et al., | 2017 | South Africa | VPM1 002 | BCG | II | 1 | Infants | Safe, IL-17 secreting CD8^+^ | (36) | MH & AN | 1^st^ Sept-31^st^ Dec 2023 | Sub-Saharan study, clinical trial, publication year, immunogenicity, efficacy, or safety vaccination study, randomized study |
| Tameris et al, | 2013 | South Africa | MVA 85A | Placebo | IIb | 1 | Infants | Safe, low CD4^+^, low VE | (45) | MH & AN | 1^st^ Sept-31^st^ Dec 2023 | Sub-Saharan study, clinical trial, publication year, immunogenicity, efficacy, or safety vaccination study, randomized study |
| Bekker et al., | 2020 | South Africa | H4: IC 31, H56: I C31 & BCG | Placebo | 1b | 2 | Adolescent s | Safe, higher CD4^+^, low CD8^+^ | (30) | MH & AN | 1^st^ Sept-31^st^ Dec 2023 | Sub-Saharan study, clinical trial, publication year, immunogenicity, efficacy, or safety vaccination study, randomized study |
| Odutola et al., | 2012 | Gambia | MVA 85A | EPI | I | 1 | Infants | Safe, IFN-γ | (25) | MH & AN | 1^st^ Sept-31^st^ Dec 2023 | Sub-Saharan study, clinical trial, publication year, immunogenicity, efficacy, or safety vaccination study, randomized study |
| Geldenhuys et al., | 2015 | South Africa | BCG | - | I & II | 1 | Infants and adults | Safe, CD4^+^, CD8^+^ | (46) | MH & AN | 1^st^ Sept-31^st^ Dec 2023 | Sub-Saharan study, clinical trial, publication year, immunogenicity, efficacy, or safety vaccination study, randomized study |
| Hesseling et al., | 2015 | South Africa | BCG | - | II | 1 | Infants | IFN-γ | (35) | MH & AN | 1^st^ Sept-31^st^ Dec 2023 | Sub-Saharan study, clinical trial, publication year, immunogenicity, efficacy, or safety vaccination study, randomized study |
| Kagina et al., | 2014 | South Africa | AERA S-402 | Placebo | I | 2 | Infants | Safe, High CD4^+^, low CD8^+^ | (28) | MH & AN | 1^st^ Sept-31^st^ Dec 2023 | Sub-Saharan study, clinical trial, publication year, immunogenicity, efficacy, or safety vaccination study, randomized study |
| Hatherill et al., | 2014 | South Africa | BCG | - | I | 1 | Adults | Safe | (47) | MH & AN | 1^st^ Sept-31^st^ Dec 2023 | Sub-Saharan study, clinical trial, publication year, immunogenicity, efficacy, or safety vaccination study, randomized study |
| Malaria | | | | | | | | | | | | |
| Bell et al., | 2022 | Ghana, Malawi, and Gabon | RTS, S/AS01/AS0 1 | Placebo | III | 3 | Children | Waning VE due to transmission intensity | (10) | MH & AN | 1^st^ Sept-31^st^ Dec 2023 | Sub-Saharan study, clinical trial, publication year, immunogenicity, efficacy, or safety vaccination study, randomized study |
| Agnandji et al., | 2014 | 7 Sub Saharan countries | RTS, S/AS01/AS0 1E | Meningococcol or rabies | III | 3 | Infants & children | Safe, partial VE | (48) | MH & AN | 1^st^ Sept-31^st^ Dec 2023 | Sub-Saharan study, clinical trial, publication year, immunogenicity, efficacy, or safety vaccination study, randomized study |
| Kimani et al., | 2014 | Kenya and Gambia | Chad 63  &MV A ME- TRAP | Placebo | Ib | 2 | Adults | CD4^+^, CD8^+^ | (13) | MH & AN |  | Sub-Saharan study, clinical trial, publication year, immunogenicity, efficacy, or safety vaccination study, randomized study |
| Partnership | 2012 | 7 Sub Saharan countries | RTS, S/AS01 & EPI | Meningococcol vaccine | III | 3 | Infants | Safe, partial VE, antibodies | (17) | MH & AN | 1^st^ Sept-31^st^ Dec 2023 | Sub-Saharan study, clinical trial, publication year, immunogenicity, efficacy, or safety vaccination study, randomized study |
| Dassah et al., | 2021 | Ghana, Uganda, Burkina Faso, and Gabon | GMZ2 | Rabies | IIb | 3 | Children | Safe, low VE | (15) | MH & AN | 1^st^ Sept-31^st^ Dec 2023 | Sub-Saharan study, clinical trial, publication year, immunogenicity, efficacy, or safety vaccination study, randomized study |
| Otieno et al., | 2020 | 7 Sub- Sahara countries | RTS, S/AS01/AS0 1 | Meningococcol or rabies vaccine | III | 3 | Infants and children | Safe, antibodies | (49) | MH & AN | 1^st^ Sept-31^st^ Dec 2023 | Sub-Saharan study, clinical trial, publication year, immunogenicity, efficacy, or safety vaccination study, randomized study |
| Bejon et al., | 2013 | 7 Sub Saharan countries | RTS, S/AS01A | Placebo/comparator vaccine | II | 3 | NA | Variable VE | (50) | MH & AN | 1^st^ Sept-31^st^ Dec 2023 | Sub-Saharan study, clinical trial, publication year, immunogenicity, efficacy, or safety vaccination study, randomized study |
| Oneko et al., | 2021 | Kenya | PfSPZ | Placebo | I & II | 1 | Infants | Low VE, Safe, undetectable T immune cells | (51) | MH & AN | 1^st^ Sept-31^st^ Dec 2023 | Sub-Saharan study, clinical trial, publication year, immunogenicity, efficacy, or safety vaccination study, randomized study |
| Bell et al., | 2020 | Malawi | RTS, S/AS01/AS0 1 | Meningo coccol or rabies | III | 4 | Infants and children | Vegetation cover affects VE | (52) | MH & AN | 1^st^ Sept-31^st^ Dec 2023 | Sub-Saharan study, clinical trial, publication year, immunogenicity, efficacy, or safety vaccination study, randomized study |
| Ouédraogo et al., | 2013 | Burkina Faso | Ad35. CS.01 | Placebo | Ib | 3 | Adults | Moderate IgG and neutralizing antibodies, safe | (12) | MH & AN | 1^st^ Sept-31^st^ Dec 2023 | Sub-Saharan study, clinical trial, publication year, immunogenicity, efficacy, or safety vaccination study, randomized study |
| Dejon-Agobe et al., | 2019 | Gabon | GMZ2 | Rabies vaccine | NA | 3 | Adults | Safe, higher IgG, modest VE | (16) | MH & AN | 1^st^ Sept-31^st^ Dec 2023 | Sub-Saharan study, clinical trial, publication year, immunogenicity, efficacy, or safety vaccination study, randomized study |
| Dobaño et al., | 2019 | Tanzania, Burki Faso, and Ghana | RTS, S/AS01/AS0 1E | Comparator vaccine | III | 3 | Infants and children | Higher IgG associated with VE | (53) | MH & AN | 1^st^ Sept-31^st^ Dec 2023 | Sub-Saharan study, clinical trial, publication year, immunogenicity, efficacy, or safety vaccination study, randomized study |
| Mendoza et al., | 2019 | 7 Sub Sahar an Africa | RTS, S/AS01 | Menongococcol or rabies | III | 4 | Infants and children | Increased febrile convulsions | (54) | MH & AN | 1^st^ Sept-31^st^ Dec 2023 | Sub-Saharan study, clinical trial, publication year, immunogenicity, efficacy, or safety vaccination study, randomized study |
| Datoo et al., | 2021 | Burkina Faso | R21/MM | Rabies vaccine | IIb | 3 | Children | Safe, anti-NANP IgG, 77% VE | (55) | MH & AN | 1^st^ Sept-31^st^ Dec 2023 | Sub-Saharan study, clinical trial, publication year, immunogenicity, efficacy, or safety vaccination study, randomized study |
| Berry et al., | 2019 | Mali | FMP2.1/ AS02A | Rabies vaccine | II | 3 | Children | High IgG1, IgG2, IgG3, IgG4, no VE | (56) | MH & AN | 1^st^ Sept-31^st^ Dec 2023 | Sub-Saharan study, clinical trial, publication year, immunogenicity, efficacy, or safety vaccination study, randomized study |
| Sirima et al., | 2017 | Burkina Faso | (PfAMA1-DiCo)- GLA SE | Placebo | Ia/Ib | 3 | Adults | IgG, Th1/Th2, safe | (14) | MH & AN | 1^st^ Sept-31^st^ Dec 2023 | Sub-Saharan study, clinical trial, publication year, immunogenicity, efficacy, or safety vaccination study, randomized study |
| Moncunill et al., | 2017 | Africa | RTS, S/AS01E | Rabies vaccine | III | 3 | Children | Polyfunctional CD4^+^ cells | (18) | MH & AN | 1^st^ Sept-31^st^ Dec 2023 | Sub-Saharan study, clinical trial, publication year, immunogenicity, efficacy, or safety vaccination study, randomized study |
| Han et al., | 2017 | Malawi | RTS, S/AS01 | Meningococcal & rabies vaccines | III | 3-4 | Infants and children | Seasonal precipitation has no effect on VE | (57) | MH & AN | 1^st^ Sept-31^st^ Dec 2023 | Sub-Saharan study, clinical trial, publication year, immunogenicity, efficacy, or safety vaccination study, randomized study |
| Sissoko et al., | 2017 | Mali | PfSPZ | Placebo | I | 5 | Adults | Safe, significant protection | (22) | MH & AN | 1^st^ Sept-31^st^ Dec 2023 | Sub-Saharan study, clinical trial, publication year, immunogenicity, efficacy, or safety vaccination study, randomized study |
| Mensah et al., | 2016 | Senegal | ChAd  63 & MVA ME-TRAP | Rabies vaccine | II | 2 | Male adults | T-cells, no VE, safe | (58) | MH & AN | 1^st^ Sept-31^st^ Dec 2023 | Sub-Saharan study, clinical trial, publication year, immunogenicity, efficacy, or safety vaccination study, randomized study |
| RTS, S partnership | 2015 | 7 Sub Saharan countries | RTS, S/AS01/AS0 1 | Comparator vaccine | III | 3 | Infants and children | Safe, significant VE, antibodies | (20) | MH & AN | 1^st^ Sept-31^st^ Dec 2023 | Sub-Saharan study, clinical trial, publication year, immunogenicity, efficacy, or safety vaccination study, randomized study |
| Thera et al., | 2016 | Mali | PfAMA1- FVO | Tetanus vaccine | I | 3 | 18-55 men and women | Safe, non-durable IgG | (59) | MH & AN | 1^st^ Sept-31^st^ Dec 2023 | Sub-Saharan study, clinical trial, publication year, immunogenicity, efficacy, or safety vaccination study, randomized study |
| Ubillos et al., | 2018 | Ghana and Mozambique | RTS, S/AS01E | NA | III | 3 | Infants and children | Protective IgG (1, 2, 3, &4) & IgM, | (60) | MH & AN | 1^st^ Sept-31^st^ Dec 2023 | Sub-Saharan study, clinical trial, publication year, immunogenicity, efficacy, or safety vaccination study, randomized study |
| Neafsey et al., | 2015 | 7 Sub- Sahara Africa | RTS, S/AS01 | Meningococcal vaccine | III | 3 | Infants and children | 50.3% VE, genetic mismatch reduces protection | (61) | MH & AN | 1^st^ Sept-31^st^ Dec 2023 | Sub-Saharan study, clinical trial, publication year, immunogenicity, efficacy, or safety vaccination study, randomized study |
| Gyaase et al., | 2021 | Ghana | RTS, S/AS01 | Meningococcal vaccine | III | 3 | Children | VE depended on SES | (62) | MH & AN | 1^st^ Sept-31^st^ Dec 2023 | Sub-Saharan study, clinical trial, publication year, immunogenicity, efficacy, or safety vaccination study, randomized study |
| Jongo et al., | 2018 | Tanzania | PfSPZ | Placebo | I | 5 | Men adults | Safe, antibodies, high CD4^+^, low CD8^+^, durable VE | (21) | MH & AN | 1^st^ Sept-31^st^ Dec 2023 | Sub-Saharan study, clinical trial, publication year, immunogenicity, efficacy, or safety vaccination study, randomized study |
| Chandramohan et al., | 2021 | Burkina Faso | RTS, S/AS01_E_ | NA | III | 3 | Children | VE comparable to chemoprevention, safe | (63) | MH & AN | 1^st^ Sept-31^st^ Dec 2023 | Sub-Saharan study, clinical trial, publication year, immunogenicity, efficacy, or safety vaccination study, randomized study |
| Shekalaghe et al., | 2014 | Tanzania | PfSPZ | Placebo | I | 2 | Adult males | Safe | (23) | MH & AN | 1^st^ Sept-31^st^ Dec 2023 | Sub-Saharan study, clinical trial, publication year, immunogenicity, efficacy, or safety vaccination study, randomized study |
